# Total Synthesis of Njaoamine C by Concurrent Macrocycle Formation

**DOI:** 10.1021/jacs.3c08410

**Published:** 2023-09-21

**Authors:** Thomas Varlet, Sören Portmann, Alois Fürstner

**Affiliations:** Max-Planck-Institut für Kohlenforschung, 45470 Mülheim/Ruhr, Germany

## Abstract

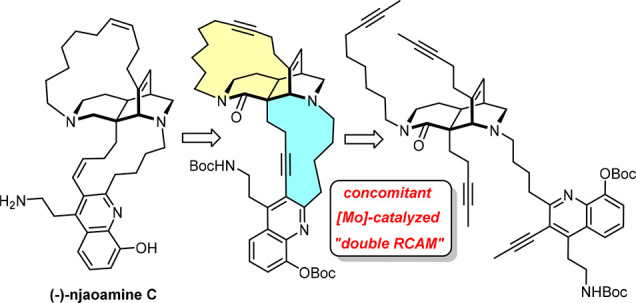

In conceptual terms, the first total synthesis of the cytotoxic marine natural product njaoamine C differs from all known approaches toward related alkaloids of the manzamine superfamily in that both macrocyclic rings enveloping the diazatricyclic core are concomitantly formed; this goal was reached by double ring closing alkyne metathesis (dRCAM). The success of this maneuver does not merely reflect a favorable preorientation of the four alkyne chains that need to be concatenated in the proper pairwise manner but is also the outcome of dynamic covalent chemistry involving error correction by the chosen “canopy” molybdenum alkylidyne catalyst. The end game downstream of dRCAM capitalizes on the striking chemoselectivity of palladium-catalyzed hydrostannation, which selects for (hetero)arylalkynes even in the presence of sterically much more accessible dialkylalkynes or alkenes; for this preference, the method complements the classical repertoire of hydrometalation and semireduction reactions.

The final attempt made by Baldwin and co-workers to emulate the proposed biosynthesis of keramaphidin B (*rac*-**1**) as the parent member of the manzamine alkaloid estate^1−3^ consisted of an intermolecular hetero-Diels–Alder reaction of the dihydropyridinium salt **4** to access tetraene **5**, which was then exposed to Grubbs catalyst **9** in the hope of enforcing the formation of both enveloping macrocycles in one pot (Scheme 1).^4^ This venturous plan was met with little success in that no more than 1–2% of **1** was obtained together with 10–20% of the monocyclized compound **6**, even though the ring closing metathesis (RCM) reaction was performed under very high dilution.^4^ The corresponding bis-hydrochloride salt **5**·2HCl was also tested to rule out that catalyst deactivation by the free amines was liable, but the yield of **1** was even lower in this case. Attempts to close the missing 13-membered ring by resubjecting **6** (or a derived salt) to **9** or **10** were to no avail either, resulting mainly in decomposition of the substrate.^4^

**Scheme 1:**
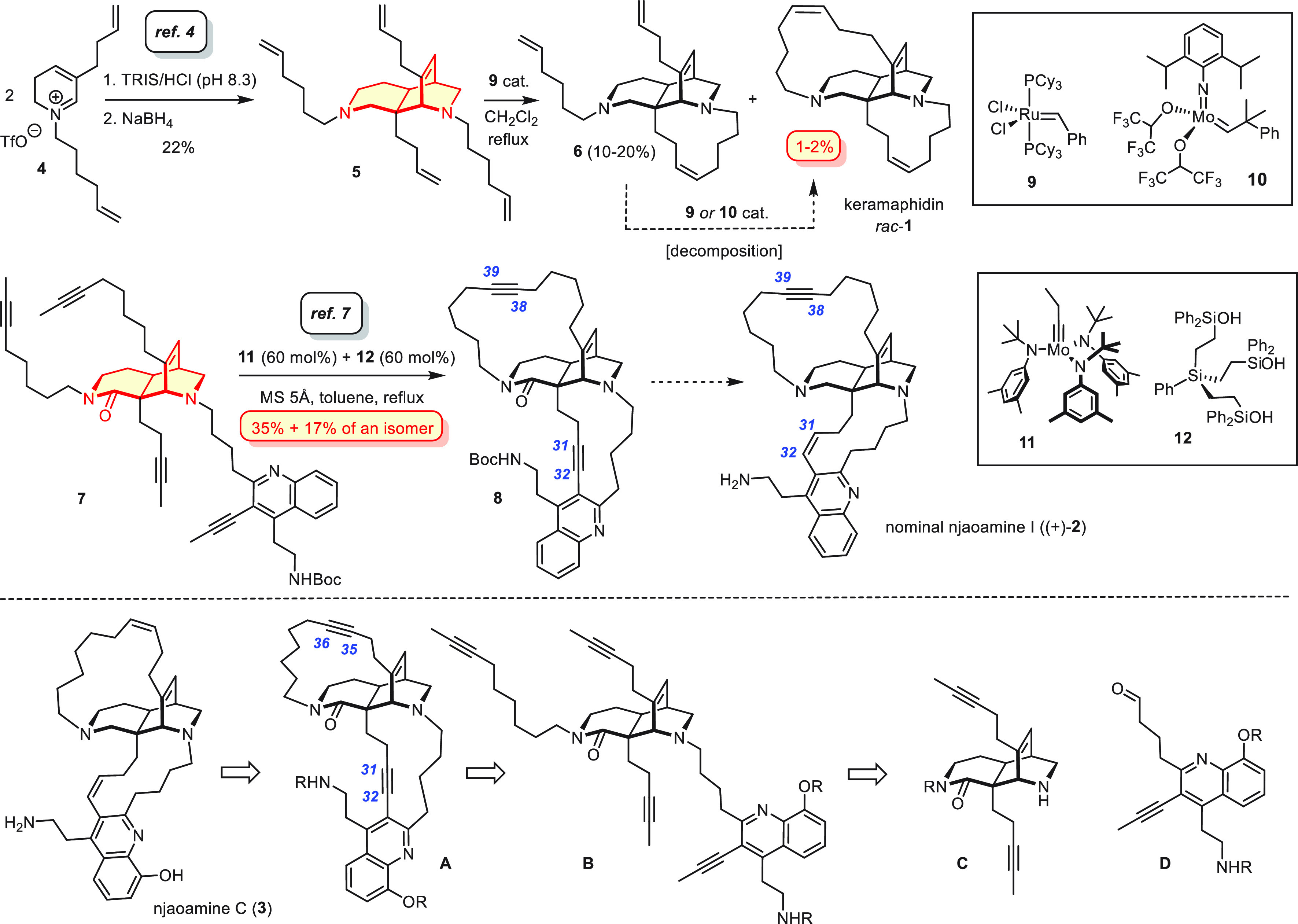
Prior Art (Top) and Retrosynthetic Analysis of Njaoamine C (Bottom)

Prompted by this poor outcome, we had deliberately based our initial foray toward this family of polycyclic marine alkaloids on a strategy involving two consecutive macrocyclization events. In the end, different variants of this blueprint turned out to be successful, allowing us to reach keramaphidin B ((+)-**1**), ingenamine A, nominal and actual xestocyclamine A, *epi*-tetrahydrohalicyclamine B, and nominal njaoamine I ((+)-**2**)^5^ (the structure of this natural product was found to be wrong and had to be corrected during our endeavor).^6−8^ Somewhat surprisingly, even sequences involving two consecutive metathesis events proved viable and high yielding (ring closing alkyne metathesis (RCAM)/RCM or RCAM/RCAM).^7^ The fact that no ene/yne or yne/yne crossover was observed in these cases piqued our curiosity and led us to reconsider the possibility of concurrent formation of both signature macrocycles.^9,10^ Indeed, tetra-yne **7** afforded product **8** in 35% yield when treated with an alkyne metathesis catalyst generated in situ from **11** and **12**, despite the presence of two basic sites in **7** that might quench the activity of the high-valent molybdenum alkylidyne.^7,11^ If one considers that the cores of tetra-ene **5** and tetra-yne **7** feature, in geometric terms, the same 1,4-etheno-bridged 2,7-diazadecaline scaffold, this outcome represents a significant advance over the Baldwin precedent,^4^ even though a substantial amount (ca. 17%) of a second isomer of unknown constitution was also formed.^7^ The price to pay was the high catalyst loading (60 mol %), which caused problems during product isolation. In the end, however, this lead finding was not pursued any further, as we saw at the time no good way to elaborate **8** into nominal njaoamine I (**2**), which would mandate selective semireduction of the sterically very hindered and deactivated C31–C32 triple bond flanking the quinoline while keeping the exposed C38–C39 alkyne inscribed into the 17-membered ring intact.^7^

This selectivity issue downstream of the double ring closing alkyne metathesis (dRCAM) event might vanish if one were to chase the sister compound njaoamine C (**3**) comprising a smaller A-ring, which is an equipotent cytotoxic agent of unknown absolute configuration derived from a *Reniera* sponge collected off the Tanzania coastline.^12^ In this case, 2-fold Lindlar-type reduction^13^ of a diyne of type **A** formed by dRCAM from tetrayne **B** should pave the way to this unconquered bioactive target of considerable architectural splendor. For this tantalizing outlook, it seemed worthwhile to study the projected dRCAM in more detail, in the hope of improving its efficiency by suppressing isomer formation and optimizing the catalyst loading.^14−16^ The latter goal, however, is arguably nontrivial because njaoamine C (**3**) comprises a *hydroxyquinoline* ring (instead of the ordinary quinoline in **2**), which is a powerful chelating ligand for most transition metal reagents and catalysts. As outlined below, we ultimately managed to meet these criteria and were able to complete the first total synthesis of (−)-**3**; as a gratifying spin-off, even a practical solution was found for the chemoselectivity issue that had previously compelled us not to pursue the dRCAM strategy en route to nominal njaoamine I (**2**).

For the assembly of an appropriate tetra-yne substrate for the envisaged dRCAM strategy, it sufficed to adapt the blueprint underlying our previous studies (Scheme 2). Specifically, compounds **14** and **16** were prepared on a multigram scale from commercial **13** and **15**, respectively, by minor adaptation of the published procedures (for details, see the Supporting Information).^7^ When treated with *t*BuOLi in tetrahydrofuran (THF) at low temperature, these building blocks engage in an exquisitely selective Michael/Michael reaction cascade to afford the tricyclic product **17** in 66% yield on a gram scale after reintroduction of the −NBoc group prior to workup; it is the C23-OTBS group (njaoamine numbering) in **16** that relays stereochemical information onto the five newly formed chiral centers. The elaboration of **17** to **19** also followed our prior work;^7^ as expected, it proved straightforward, high yielding, and scalable. The C23-OH group, which had been quintessential for the assembly process, was then removed by conversion into a chloromethylsulfonate followed by base-induced elimination;^17,18^ the resulting cyclic enamide **20** was best reduced with NaBH_3_CN in neat formic acid to give **21** in high overall yield.^19,20^

**Scheme 2:**
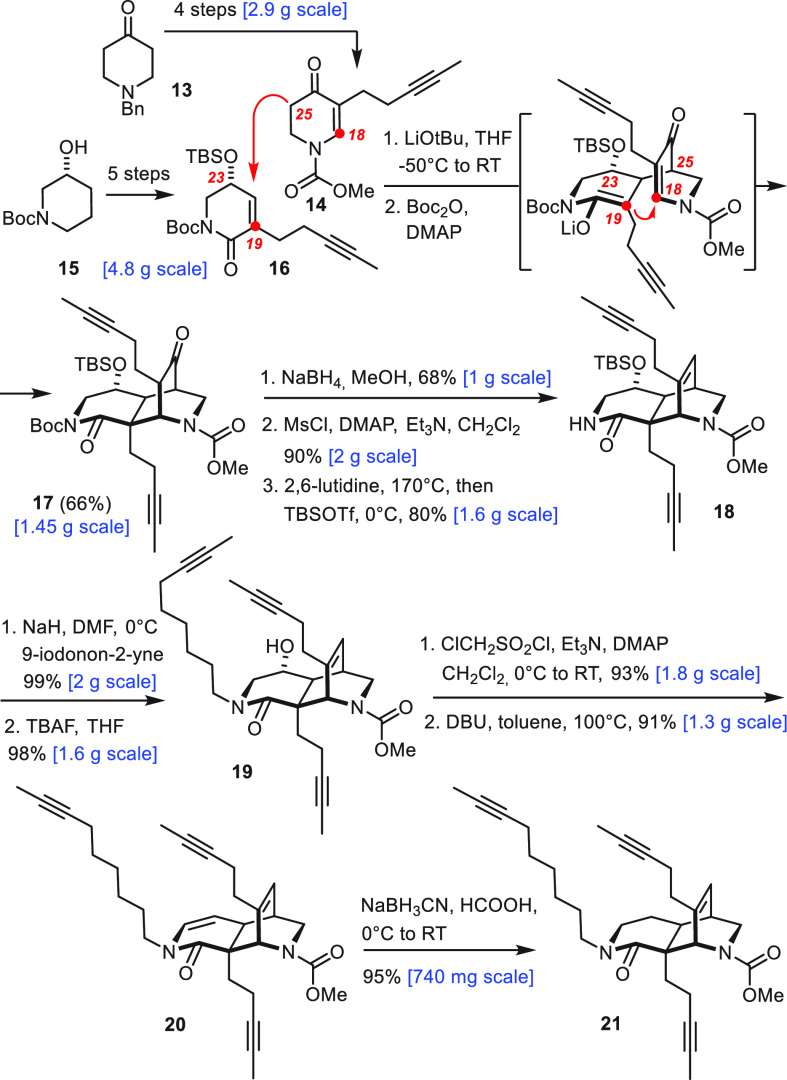
Diazatricyclic Core The scales shown in this and the following schemes refer to the single largest batch.

We had originally planned to access the required hydroxyquinoline building block by regioselective C–H borylation/oxidation of the quinoline fragment **22** previously used en route to njaoamine I.^7^ This shortcut had been inspired by a literature report describing iridium-catalyzed C8-selective C–H borylations of quinolines with the aid of a silica-supported phosphine ligand.^21^ When applied to **22**, however, alcohol **23** formed by reduction of the ketone was the only discernible product in the crude mixture (Scheme 3).^22^ Therefore we pursued a de novo synthesis starting from commercial 7-benzyloxyindole **24**. This compound was readily elaborated into the tryptamine derivative **26** without the need to purify any intermediate compound by flash chromatography.^23^ Oxidative ring cleavage with NaIO_4_ followed by N-deformylation with HCl/MeOH afforded **27**, which reacted with **28** in a Dieckmann-type condensation to give hydroxyquinoline **29** in good yield.^24^ The derived triflate **30**(25) was cross coupled with the alkylborane generated from alkene **31** and 9-H-9-BBN; while optimizing this step, NaOAc was identified as a particularly effective (though apparently underutilized) promoter for the alkyl-Suzuki–Miyaura reaction.^26^ The benzyl ether in **32** inherited from the commercial substrate had to be swapped for a Boc-group^27^ prior to installation of the nonterminal alkyne, which was accomplished in almost quantitative yield by enol triflate formation/elimination at low temperature.^28^ Routine protecting group management followed by oxidation of the primary alcohol then furnished aldehyde **34** in readiness for attachment to the core.^29^

**Scheme 3:**
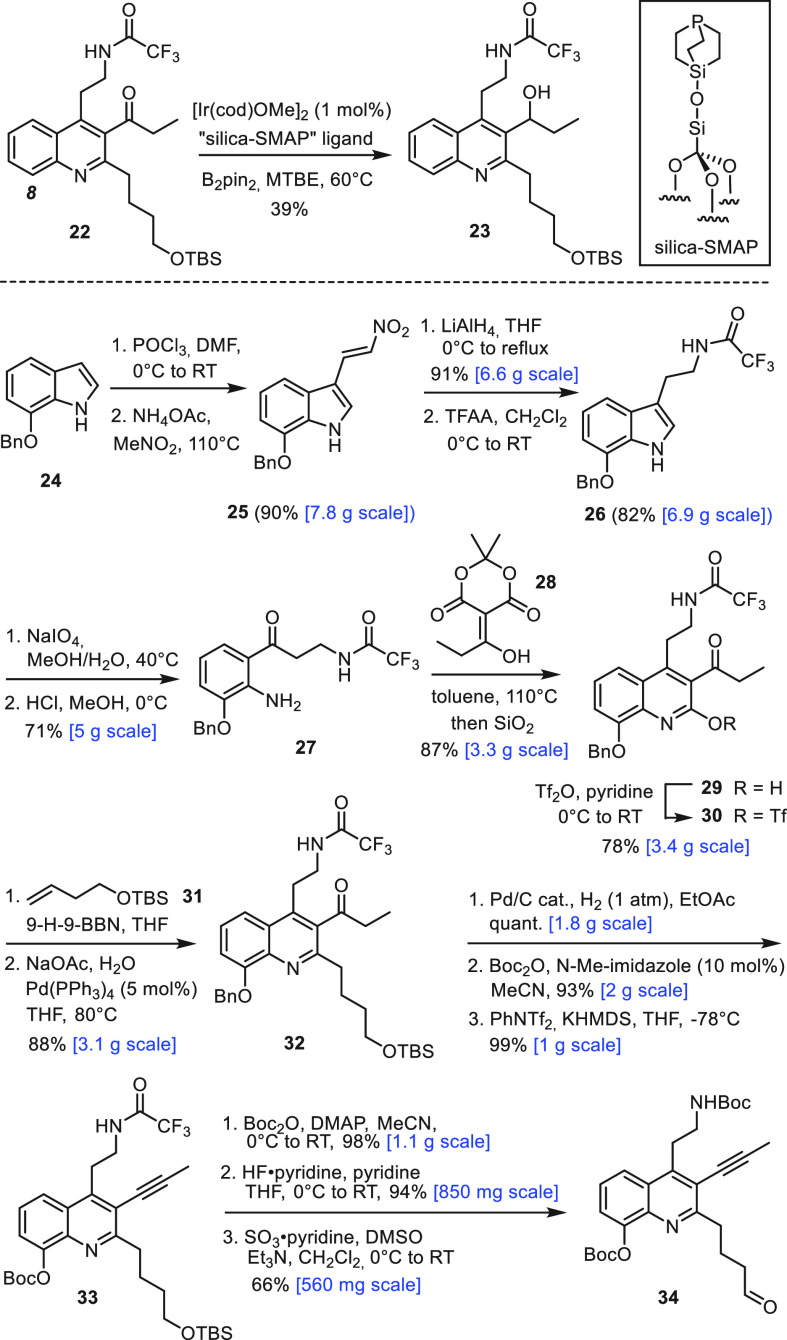
Hydroxyquinoline Segment

The methyl carbamate of **21** was then cleaved off with L-Selectride, and the resulting amine subjected to reductive amination with **34** to give tetra-yne **35** in well reproducible 58% yield (Scheme 4).^30^ With copious material in hand, the stage was set for the decisive dRCAM step. Rather than employing the two-component system **11**/**12**,^11^ we resorted to the “canopy catalyst” series for alkyne metathesis, as they are structurally well-defined entities of exquisite performance for reasons that are well understood by now.^31−36^ Complex **40a** as the lead member combines high reactivity with a remarkable tolerance toward functional groups, including numerous basic sites.^31,37^ Indeed, **40a** converted tetra-yne **35** by dRCAM into diyne **36** as the only detectable product (^1^H NMR) in ≤1 h of reaction time. After brief optimization, we settled on the use of 20 mol % of **40a** in toluene (2 mM) at 60 °C^38^ in the presence of MS 5 Å as a 2-butyne sequestering agent;^39,40^ under these conditions, **36** was isolated in 91% yield. Prior to workup, however, one must make sure that the catalyst is completely removed by filtration through a plug of silica; otherwise, the product (partly) decomposes upon evaporation of the solvent. Cycloalkyne formation is hence reversible, and ring opening is obviously so facile that it can ruin the outcome.

**Scheme 4:**
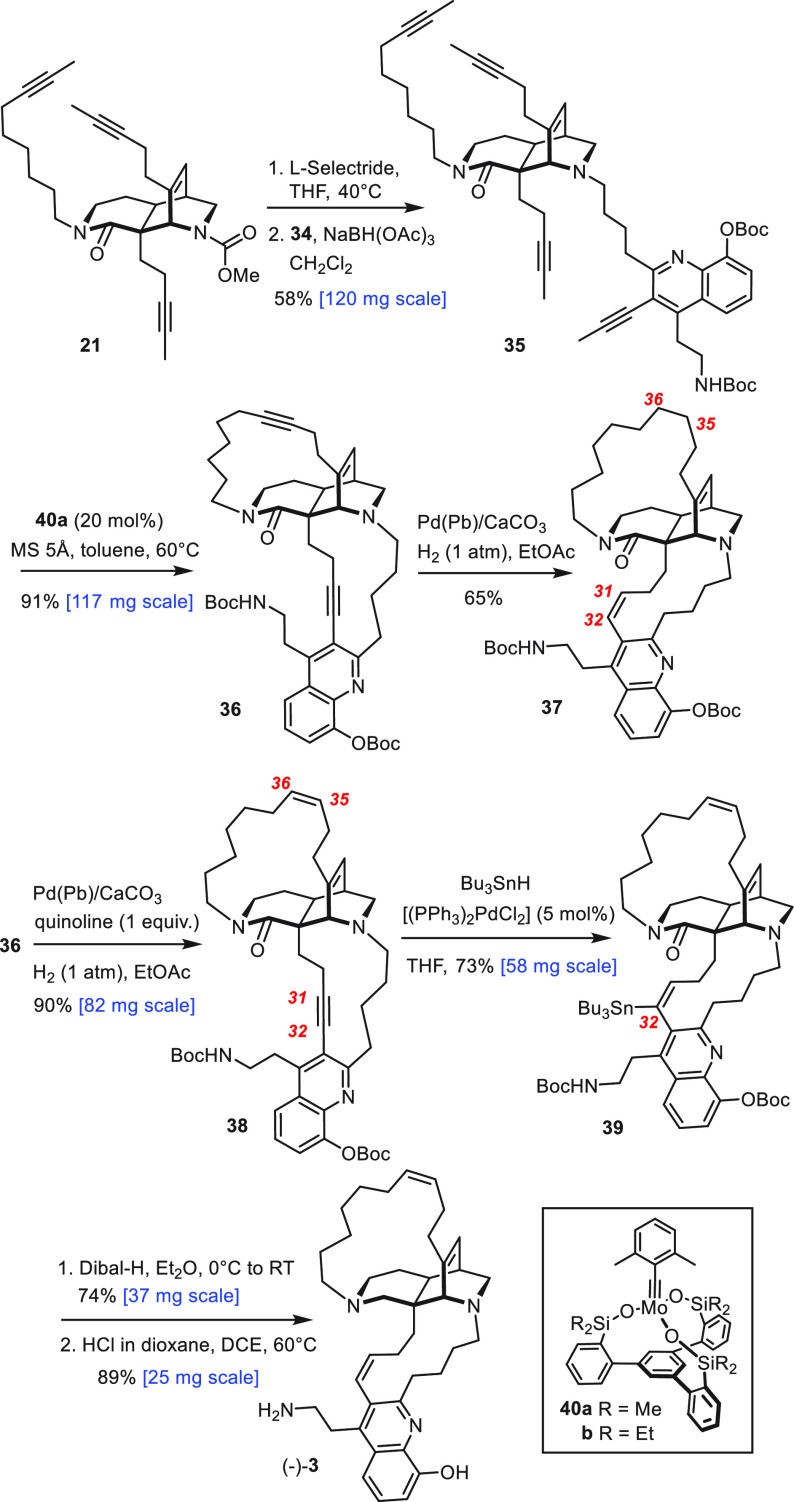
Completion of the Total Synthesis

Interestingly, the use of **40a** was mandatory; even the only slightly more encumbered sibling **40b**(41) proved much less adequate in that it furnished a mixture, of which the desired product **36** is only a minor constituent (≈ 10%). LC-MS and ^1^H NMR indicate the presence of several monomeric compounds with only one macrocycle closed; other components are dimeric in nature (for details, see the Supporting Information). Details apart, this product distribution implies that tetra-yne **35** does not transform straight away into **36**; rather, it is first engaged in more random inter- as well as intramolecular alkyne metathesis reactions. Because of the lower activity of **40b**, the initial product distribution does not evolve much with time, whereas **40a** is capable of reopening cycloalkynes once formed and also likely able to cleave dimeric species. Indeed, when **40a** (30 mol %) was added to the mixture, the desired product **36** accumulated and could be isolated in 58% yield.^42^ These observations imply that the selective formation of **36** is not solely reflecting a favorable geometric preorientation of the four alkyne side chains branching off the tricyclic core of **35**, since other ways of connecting the termini are possible. For the successful formation of **36**, dynamic covalent chemistry (DCC) must come into play,^43^ in that the catalyst corrects initial mistakes by scrambling of the mixture and hence gives the target compound the chance to accumulate. While DCC employing alkyne metathesis has gained prominence in material science,^14,44^ the current example is arguably the first advanced incarnation in the realm of natural product total synthesis.^45^

The ease of the dRCAM reaction stood in sharp contrast to the difficulties with the supposedly trivial semireduction of diyne **36**.^13^ Despite considerable experimentation, we were incapable of reducing both triple bonds concomitantly to the corresponding *Z*-alkenes. The shielded C31–C32 alkyne invariably became reduced only after the exposed C35–C36 triple bond had been fully saturated to furnish product **37**; otherwise, it remained untouched to give enyne **38**. Confronted with this impasse, several alternative strategies were contemplated.^46^ In the end, we opted for metal-catalyzed hydrostannation, which is common for alkynes but infrequent for alkenes devoid of steering substituents.^47,48^ Indeed, only the shielded triple bond of **38** was engaged in a palladium-catalyzed hydrostannation, whereas the exposed olefin remained untouched. Although this chemoselectivity pattern is not unprecedented,^48,49^ the outcome is striking if one considers that palladium-catalyzed hydrogenation saturates the alkene before the alkyne starts to react. Compound **39** was formed essentially as a single alkenylstannane regioisomer.^50^ The amide group of **39** was reduced to the corresponding amine with Dibal-H in Et_2_O; under these conditions, the carbonate group was also cleaved off of the hydroxyquinoline, but the heterocyclic ring itself remained untouched. Subsequent treatment with HCl in 1,4-dioxane at 60 °C entailed concomitant protodestannation^51^ and cleavage of the second Boc group. The spectroscopic data of synthetic njaoamine C (−)-**3** thus formed were in excellent agreement with those reported in the literature, but the [α]_D_ is of opposite sign;^12^ our sample hence represents the enantiomer of the natural product (for details, see the Supporting Information).

The “reversed” chemoselectivity profile of the palladium-catalyzed hydrostannation manifested in the selective formation of **39** carries even further. An early literature report had shown that 4-octyne remained largely unchanged under conditions where PhC≡CMe reacted well.^49,52^ This somewhat hidden information on what seems to be differential reactivity of substantial measure worked in our favor (Scheme 5). Specifically, the remaining sample of tetra-yne **7** from our previous study on njaoamine I was resubjected to dRCAM with **40a** (25 mol %) as the catalyst to furnish product **8** in a significantly improved yield of 59%.^7^ Gratifyingly, it was the shielded triple bond branching off the quinoline ring that had resisted all previous attempts at selective manipulation, which succumbed to palladium-catalyzed hydrostannation, whereas the exposed dialkylalkyne within the 17-membered ring remained unchanged. Subsequent protodestannation of **41** gave **42** in an unoptimized 30% yield, which potentially connects to njaoamine I (**2**) by amide reduction.^53^

**Scheme 5:**
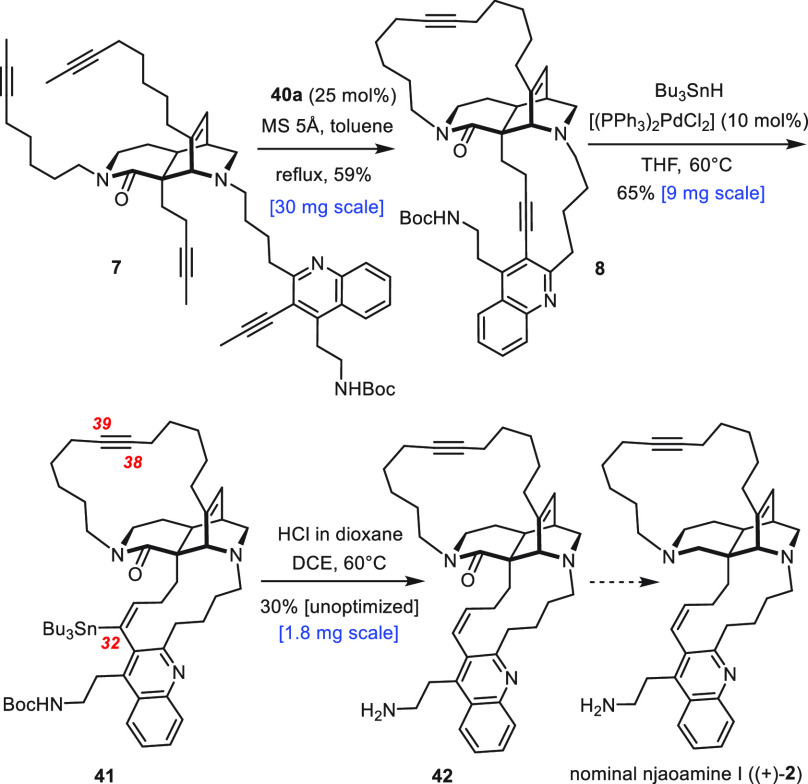
Route to Nominal Njaoamine I Revisited

These case studies allowed us to conclude that concomitant dRCAM is an emanation of dynamic covalent chemistry, which in turn is the fruit of ever more powerful alkyne metathesis catalysts; it constitutes a conceptually new strategy for the synthesis of polycyclic targets, even if of low overall symmetry. It gains its full potential when combined with chemoselective downstream functionalization reactions, which allow one or the other triple bond of a diyne to be addressed at will. Ongoing work in our group explores further possibilities along these lines.

## Supporting Information Available

Experimental Section including characterization data (PDF)
NMR spectra of new compounds (PDF)
