# Supplementary material for: Total Synthesis of Njaoamine C by Concurrent Macrocycle Formation
Source: J Am Chem Soc. 2023 Sep 21;145(39):21197–202. doi: 10.1021/jacs.3c08410 (PMC10557140; doi:10.1021/jacs.3c08410)
Supplement: Supplementary file 1 — ja3c08410_si_001.pdf [file ja3c08410_si_001.pdf]

SUPPORTING INFORMATION

Total Synthesis of Njaoamine C by Concurrent Macrocycle Formation

Thomas Varlet, Sören Portmann, and Alois Fürstner*

Max-Planck-Institut für Kohlenforschung, 45470 Mülheim/Ruhr, Germany

E-Mail: fuerstner@kofo.mpg.de

Table of Contents

General Information......S2

Synthesis of the Tricyclic Core......S3

Synthesis of the Quinoline Core......S13

Assembly and Concurrent Macrocyclization by dRCAM......S20

Completion of the Total Synthesis of Njaoamine C......S23

The dRCAM Route to Nominal Njaoamine I Revisited......S34

References......S36

General Information

Unless stated otherwise, all reactions were carried out in flame-dried glassware using anhydrous solvents under argon atmosphere.

The solvents were purified by distillation over the indicated drying agents and were transferred under argon: THF, Et_2O (Mg/anthracene); acetonitrile, 2,6-lutidine, CH_2Cl_2, 1,2-DCE, nitromethane (CaH_2); toluene (Na/K alloy); MeOH (Mg, stored over MS 3 Å). DMSO, DMF, Et_3N, pentane and pyridine were dried by an adsorption solvent purification system based on molecular sieves.

Thin layer chromatography (TLC): Macherey-Nagel pre-coated plates (POLYGRAM®SIL/UV254). Detection was achieved under UV-light (254 nm) and by staining with either acidic *p*-anisaldehyde, cerium ammonium molybdenate, or basic $KMnO_4$ solution. Flash chromatography: Merck silica gel 60 (40–63 µm) with predistilled or HPLC grade solvents.

NMR: spectra were recorded on Bruker AV 400, AV 500, AVIII 600 or AVneo 600 spectrometers in the indicated solvents; chemical shifts (δ) are given in ppm relative to TMS, coupling constants (*J*) in Hz. All spectra were recorded at 25 °C. The solvent signals were used as references and the chemical shifts converted to the TMS scale ($CDCl_3$: δ_C = 77.16 ppm; residual $CHCl_3$ in $CDCl_3$: δ_H = 7.26 ppm; CD_3OD: δ_C = 49.00 ppm, residual CD_2HOD in CD_3OD: δ_H = 3.31 ppm; CD_2Cl_2: δ_C = 53.84 ppm, residual $CDHCl_2$: δ_H = 5.32 ppm; [D_5]-pyridine: δ_C = 123.50 ppm, residual C_5D_4HN: δ_H = 7.19 ppm). Multiplicities are indicated by the following abbreviations: s: singlet, d: doublet, t: triplet, q: quartet, hept: heptet, m: multiplet, br. s: broad singlet. ^{13}C NMR spectra were recorded in ^{1}H-decoupled manner and the values of the chemical shifts are rounded to one decimal point. Signal assignments were established using HSQC, HMBC, COSY, NOESY and other 2D experiments.

IR: Spectra were recorded on an Alpha Platinum ATR instrument (Bruker), wave numbers ($\tilde{\nu}$) in cm^{-1}.

MS (ESI-MS): Finnigan MAT 8200 (70 eV), ESI-MS: ESQ3000 (Bruker), accurate mass determinations: Bruker APEX III FTMS (7 T magnet) or Mat 95 (Finnigan).

Optical rotations ($[\alpha]_D^{20}$) were measured with an A-Krüss Optronic Model P8000-t polarimeter at a wavelength of 589 nm.

Molecular sieve (5 Å) was activated at 150 °C for 24 h under high vacuum (1×10^{-3} mbar) and stored under argon.

Unless stated otherwise, commercially available compounds (Alfa Aesar, Aldrich, TCI, Strem Chemicals, ChemPUR) were used as received. The following compounds were prepared according to the cited literature: 5-iodopent-2-yne,[1] and molybdenum alkylidyne complexes **40a**[2] and **40b**.[3]

Synthesis of the Tricyclic Core

tert-Butyl (*R*)-5-((*tert*-butyldimethylsilyl)oxy)-2-oxopiperidine-1-carboxylate (S1).[4] ***Step 1***: 4-Dimethyl-aminopyridine (0.55 eq., 54.7 mmol, 6.68 g) and triethylamine (13.9 mL, 99.4 mmol) were added to a solution of (*R*)-1-(*tert*-butoxycarbonyl)-3-hydroxypiperidine (**15**) (20.0 g, 99.4 mmol) in CH_2Cl_2 (200 mL) at room temperature. After 5 min, *tert*-butyldimethylsilyl chloride (16.4 g, 109.3 mmol) was added. The mixture was stirred for 16 h before it was poured into ice-cold water (100 mL). The resulting mixture was extracted with CH_2Cl_2 (3 x 100 mL), the combined organic layers were washed with brine (50 mL), dried over anhydrous Na_2SO_4 and filtered. After removing the solvent under reduced pressure, the residue was purified by flash chromatography on silica gel (hexanes/EtOAc, 20:1) to provide the desired product as a colorless oil.

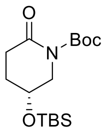

Step 2: $RuO_2{\bullet}xH_2O$ (832 mg, 6.25 mmol) was added to a solution of the obtained oil in EtOAc/H_2O (1.28 L, 3:1), followed by $NaIO_4$ (100.0 g, 469 mmol). The mixture was stirred for 1.5 h before being filtered through a pad of Celite. The filtrate was washed with brine (100 mL) and dried over anhydrous Na_2SO_4. After filtration and evaporation of the solvent under reduced pressure, the residue was purified by flash chromatography on silica gel (hexanes/EtOAc, 20:1) to provide title compound as a colorless oil (20.2 g, 59%). $[\alpha]_D^{20}$ = −6.1 (c = 1.6, $CHCl_3$); ^{1}H NMR (400 MHz, $CDCl_3$): δ = 4.14 (ddd, *J* = 8.6, 4.7, 2.8 Hz, 1H), 3.70 – 3.57 (m, 2H), 2.70 (ddd, *J* = 17.1, 9.1, 6.7 Hz, 1H), 2.42 (dt, *J* = 17.2, 6.2 Hz, 1H), 1.95 (dddd, *J* = 13.2, 9.1, 6.4, 3.9 Hz, 1H), 1.86 – 1.77 (m, 1H), 1.51 (s, 9H), 0.87 (s, 9H), 0.07 (s, 6H) ppm; ^{13}C NMR (101 MHz, $CDCl_3$): δ = 171.1, 152.6, 83.0, 64.6, 52.6, 31.2, 29.1, 28.1 (3C), 25.8 (3C), 18.1, −4.7, −4.8 ppm; IR (film) $\tilde{\nu}$ = 2930, 2857, 1772, 1295, 1249, 1147, 883, 775 cm^{-1}; HRMS (ESI): *m/z:* calcd. for $C_{16}H_{32}NO_4Si$ [M+H$^+$]: 330.20951, found: 330.20940.
The data are consistent with those reported in the literature.[4]

The optical purity of (*R*)-**S1** (> 99% *ee*) was determined by chiral HPLC on a chiral stationary phase (Chiralpak IC-3, 3 µm, 150 mm, 4.6 mm i.D.).

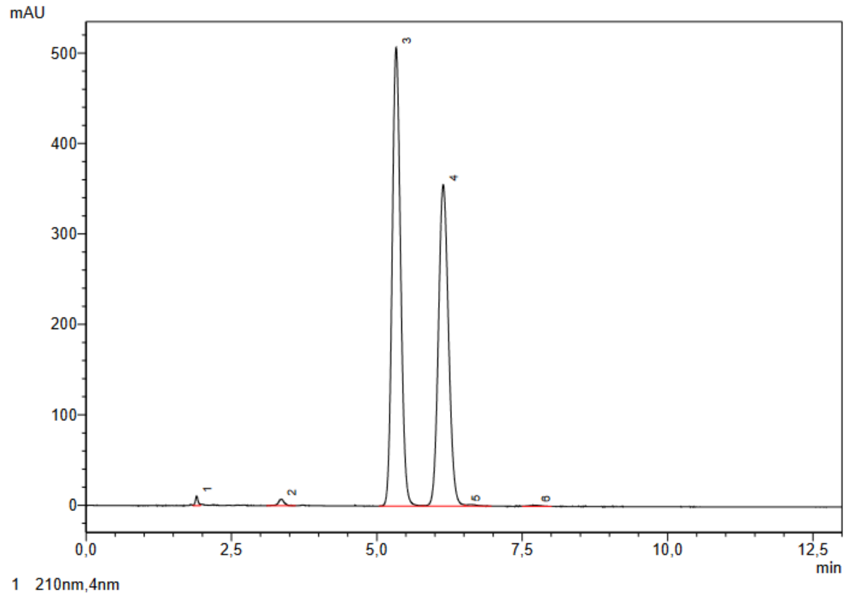

1 210nm,4nm

PDA Ch1 210nm

Peak #	Ret. Time	Area %	Name
1	1,90	0,37	
2	3,36	0,56	
3	5,33	53,69	1. Enantiomer
4	6,14	44,99	2. Enantiomer
5	6,52	0,22	
6	7,73	0,18	
Total		100,00	

VAT-VA-023-01
150 mm Chiralpak IC-3, 3 µm, 4.6 mm i.D.
n-Heptan / 2-Propanol = 80:20
1.0 mL/min / 298 K / 9.1 MPa

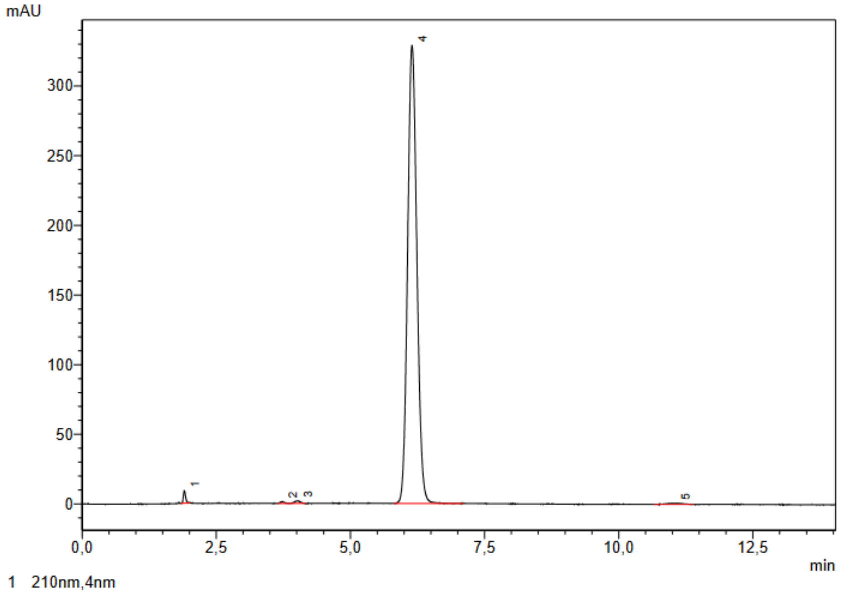

1 210nm,4nm

PDA Ch1 210nm

Peak #	Ret. Time	Area %	Name
1	1,90	0,65	
2	3,73	0,17	
3	4,01	0,35	
4	6,15	98,45	1. Enantiomer > 99 % ee
5	11,05	0,38	
Total		100,00	

3-Allyl 1-(*tert*-butyl) (5*R*)-5-((*tert*-butyldimethylsilyl)oxy)-2-oxopiperidine-1,3-dicarboxylate (S2).[5] LiHMDS (1 M in THF, 102 mL, 101.7 mmol) was slowly added to a solution of **S1** (11.55 g, 35.05 mmol) in THF (75 ml) at –78 °C (dry ice/acetone bath). Once the addition was complete, the mixture was stirred for 1 h at –78 °C before allyl chloroformate (3.9 mL, 36.80 mmol) was added dropwise. After stirring for additional 30 min at –78 °C, the reaction was quenched with sat. aq. NH_4Cl (10 mL). The resulting mixture was extracted with EtOAc (3 x 200 mL), and the combined organic layers were washed with brine (100 mL), dried over anhydrous Na_2SO_4 and filtered. After evaporation of the solvent under reduced pressure, the residue was purified by flash chromatography on silica gel (hexanes/EtOAc, 5:1 to 3:1) to provide the title compound as a yellow oil (13.37 g, 32.32 mmol, 92%). $[\alpha]_D^{20}$ = –14.3 (c = 0.2, $CHCl_3$); ^{1}H NMR (400 MHz, $CDCl_3$) *mixture of diastereomers*: δ = 5.99 – 5.87 (m, 1H), 5.36 (dp, *J* = 17.2, 1.5 Hz, 1H), 5.25 (ddt, *J* = 10.5, 2.2, 1.2 Hz, 1H), 4.75 – 4.62 (m, 2H), 4.26 (p, *J* = 3.9 Hz, 0.7H, major), 4.17 – 4.10 (m, 0.3H, minor), 3.84 – 3.56 (m, 2.7H), 3.46 (dd, *J* = 10.0, 7.3 Hz, 0.3H, minor), 2.37 – 2.19 (m, 1.3H), 2.09 (dddd, *J* = 13.6, 6.4, 4.6, 1.6 Hz, 0.7H), 1.52 (d, *J* = 1.8 Hz, 9H), 0.87 (d, *J* = 1.6 Hz, 9H), 0.08 (d, *J* = 2.6 Hz, 6H) ppm; ^{13}C NMR (101 MHz, $CDCl_3$) *mixture of diastereomers*: δ = 169.8, 168.9, 167.0, 152.7, 152.4, 131.80, 131.78, 118.84, 118.76, 83.7, 83.6, 66.3, 64.5, 63.4, 52.4, 51.5, 49.7, 48.2, 33.5, 33.0, 28.1, 25.8, 18.1, –4.6, –4.72, –4.77, –4.79 ppm; IR (film) $\tilde{\nu}$ = 2954, 2932, 2889, 2858, 1776, 1746, 1721, 1471, 1462, 1368, 1297, 1254, 1148, 838, 779 cm^{-1}; HRMS (ESI): *m/z*: calcd. for $C_{20}H_{35}NO_6SiNa$ $[M+Na]^+$: 436.21278, found: 436.21259.

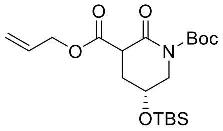

The data are consistent with those reported in the literature.[5]

3-Allyl 1-(*tert*-butyl) (5*R*)-5-((*tert*-butyldimethylsilyl)oxy)-2-oxo-3-(pent-3-yn-1-yl)piperidine-1,3-dicarboxylate (S3).[5] Cs_2CO_3 (11.08 g, 34.00 mmol) was added to a solution of **S2** (7.03 g, 17.00 mmol) and 5-iodo-2-pentyne (6.60 g, 34.00 mmol)[1] in DMF (34 mL) at room temperature. The mixture was stirred for 12 h before the reaction was quenched with sat. aq. NH_4Cl (10 mL). The resulting solution was extracted with EtOAc (3 x 50 mL). The combined organic layers were dried over anhydrous Na_2SO_4, filtered, and concentrated under reduced pressure. The residue was purified by flash chromatography on silica gel (hexanes/EtOAc, 10:1) to afford title compound as a colorless oil (7.86 g, 96%). $[\alpha]_D^{20}$ = —10.2 (c = 1.8, $CHCl_3$); ^{1}H NMR (400 MHz, $CDCl_3$) *mixture of diastereomers*: δ = 5.99–5.73 (m, 1H), 5.42–5.27 (m, 1H), 5.25–5.16 (m, 1H), 4.68–4.52 (m, 2H), 4.20–4.04 (m, 1H), 3.83–3.69 (m, 1H), 3.51–3.37 (m, 1H), 2.68–2.41 (m, 1H), 2.31–1.96 (m, 4H), 1.80–1.66 (m, 4H), 1.49 (s, 9H), 0.89–0.79 (m, 9H), 0.09–0.03 (m, 6H) ppm; ^{13}C NMR (101 MHz, $CDCl_3$) *mixture of diastereomers*: δ = 171.2, 171.1, 169.5, 169.0, 152.8, 152.7, 131.5, 131.3, 119.1, 118.5, 83.3, 83.2, 78.2, 78.1, 76.39, 76.35, 66.4, 66.2, 64.1, 63.9, 55.4, 54.6, 51.3, 51.0, 38.9, 38.8, 36.0, 35.6, 28.1, 25.81, 25.75, 18.2, 18.0, 14.8, 14.6, 3.6, 3.5, -4.69, -4.71, -4.83, -4.85 ppm; IR (film) $\tilde{\nu}$ = 2926, 2856, 1717, 1376, 1300, 1254, 1147, 1092, 836, 777 cm^{-1}; HRMS (ESI): *m/z:* calcd. for $C_{25}H_{41}NO_6SiNa$ $[M+Na^+]$: 502.2595, found: 502.2597.

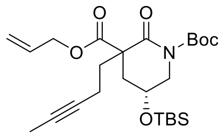

The data are consistent with those reported in the literature.[5]

tert-Butyl (*R*)-3-((*tert*-butyldimethylsilyl)oxy)-6-oxo-5-(pent-3-yn-1-yl)-3,6-dihydropyridine-1(2H)-carboxylate

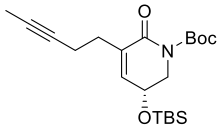

(16).[5] $Pd_2(dba)_3$•$CHCl_3$ (0.820 mmol, 848 mg, 6 mol%) was added to a solution of **S3** (6.34 g, 13.22 mmol) in acetonitrile (60 mL) at room temperature. The mixture was stirred at 80 °C (oil bath) for 30 min before it was cooled to room temperature and filtered through a pad of Celite. After removing the solvent under reduced pressure, the residue was purified by flash chromatography on silica gel (hexanes/CH_2Cl_2, 1:1 to 1:4) to remove the benzylidene-acetone; the eluent was then changed to hexanes/*tert*-butyl methyl ether (4:1) to afford title compound as a colorless oil (4.82 g, 92%). $[\alpha]_D^{20}$ = 56.3 (c = 0.9, $CHCl_3$); ^{1}H NMR (400 MHz, $CDCl_3$) δ = 6.51–6.48 (m, 1H), 4.50–4.44 (m, 1H), 3.91 (ddd, *J* = 12.8, 4.9, 1.2 Hz, 1H), 3.64 (dd, *J* = 12.8, 8.2 Hz, 1H), 2.57–2.36 (m, 2H), 2.32 (ddddd, *J* = 6.3, 5.3, 3.8, 2.6, 1.0 Hz, 2H), 1.75 (t, *J* = 2.5 Hz, 3H), 1.54 (s, 9H), 0.89 (s, 9H), 0.11 (s, 6H) ppm; ^{13}C NMR (101 MHz, $CDCl_3$) δ = 163.8, 152.9, 142.3, 134.2, 83.2, 78.3, 77.4, 64.1, 50.9, 30.1, 28.2 (3C), 25.8 (3C), 18.2, 18.1, 3.6, –4.55, –4.59 ppm; IR (film) $\tilde{\nu}$ = 2930, 2857, 1715, 1368, 1301, 1255, 1093, 837, 778 cm^{-1}; HRMS (ESI): *m/z:* calcd. for $C_{21}H_{35}NO_4SiNa$ [*M*+Na$^+$]: 416.22276, found: 416.22272.

The data are consistent with those reported in the literature.[5]

Allyl 1-benzyl-4-hydroxy-1,2,5,6-tetrahydropyridine-3-carboxylate (S4).[5] A Schlenk-flask was charged with NaH (4.2 g, 175.02 mmol) before THF (87 mL) was added. The suspension was cooled to 0 °C using an ice bath and 1-benzyl-4-piperidone (**13**) (13.0 mL, 70.13 mmol) was added dropwise as a solution in THF (30 mL). Next, the reaction mixture was warmed to room temperature before diallylcarbonate (15.0 mL, 104.6 mmol) was added and the resulting mixture was stirred at room temperature for 18 h. Sat. aq. NH_4Cl (30 mL) was carefully added, the aqueous layer was diluted with H_2O (50 mL) and extracted with EtOAc (3 x 150 mL). The combined organic layers were washed with brine (20 mL), dried over anhydrous $MgSO_4$ and concentrated under reduced pressure. The residue was purified by flash chromatography on silica gel (hexanes/EtOAc 5:1), furnishing the title compound as a colorless oil (7.02 g, 37%).

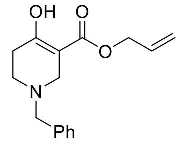

^{1}H NMR (400 MHz, $CDCl_3$, *mixture of the keto-ester and enol form*): δ = 11.93 (s, 0.7H), 7.37 – 7.27 (m, 5H), 5.91 (dddt, *J* = 17.2, 10.4, 9.1, 5.6 Hz, 1H), 5.38 – 5.19 (m, 2H), 4.73 – 4.56 (m, 2H), 3.65 (d, *J* = 2.5 Hz, 2H), 3.50 (ddd, *J* = 7.9, 5.0, 1.3 Hz, 0.25H), 3.24 (t, *J* = 1.8 Hz, 1.5H), 3.08 (ddd, *J* = 11.6, 7.8, 1.2 Hz, 0.25H), 2.96 (ddd, *J* = 11.7, 5.0, 1.7 Hz, 0.25H), 2.84 (dddd, *J* = 11.7, 6.3, 5.6, 1.7 Hz, 0.25H), 2.75 (dddd, *J* = 11.3, 8.1, 4.8, 1.2 Hz, 0.25H), 2.64 – 2.50 (m, 2H), 2.41 (td, *J* = 5.9, 3.1 Hz, 1.5H) ppm; ^{13}C NMR (101 MHz, $CDCl_3$, *mixture of the keto-ester and enol form*): δ = 204.1, 170.9, 170.7, 168.7, 137.9, 132.2, 131.8, 129.2, 129.0, 128.6, 128.5, 127.6, 127.4, 118.8, 118.2, 96.8, 66.0, 64.9, 62.2, 61.7, 56.7, 55.3, 53.2, 50.1, 48.6, 40.9, 29.6 ppm; IR (film): $\tilde{\nu}$ = 3063, 3028, 2935, 2808, 2764, 1743, 1720, 1664, 1622, 1495, 1453, 1418, 1403, 1367, 1350, 1302, 1285, 1233, 1212, 1193, 1168, 1126, 1078, 1052, 1028, 994, 972, 934, 815, 742, 699 cm^{-1}; HRMS (ESI): *m*/*z* calcd. for $C_{16}H_{20}NO_3$ [M+H$^+$]: 274.14377, found: 274.14376.

The data are consistent with the data reported in the literature.[5]

Allyl 1-benzyl-4-oxo-3-(pent-3-yn-1-yl)piperidine-3-carboxylate (S5).[5] 5-Iodopent-2-yne (8.14 g, 41.96 mmol)[1] and Cs_2CO_3 (14.22 g, 43.64 mmol) were added in three portions (1:1:0.5) to a solution of compound **S4** (4.59 g, 16.80 mmol) in DMF (17 mL) at room temperature (the second and third portion were added 30 min and 1 h after the first portion). The mixture was stirred for 3 h before the reaction was quenched with sat. aq. NH_4Cl (15 mL). The aqueous layer was extracted with EtOAc (3 x 250 mL). The combined organic layers were washed with brine (30 mL), dried over anhydrous $MgSO_4$, and concentrated under reduced pressure. The residue was purified by flash chromatography on silica gel (hexanes/EtOAc 5:1) to furnish the title compound as a colorless oil (4.97 g, 87%). ^{1}H NMR (400 MHz, $CDCl_3$): δ = 7.29 – 7.20 (m, 5H), 5.82 (ddt, *J* = 17.2, 10.4, 5.8 Hz, 1H), 5.32 – 5.14 (m, 2H), 4.58 (qdt, *J* = 13.1, 5.8, 1.4 Hz, 2H), 3.52 (d, *J* = 1.9 Hz, 2H), 3.36 (dd, *J* = 11.6, 2.6 Hz, 1H), 2.92 (dtd, *J* = 12.8, 5.9, 3.5 Hz, 1H), 2.79 (ddd, *J* = 16.0, 12.3, 6.6 Hz, 1H), 2.39 – 2.28 (m, 2H), 2.28 – 2.12 (m, 2H), 2.04 – 1.86 (m, 2H), 1.76 – 1.68 (m, 1H), 1.67 (t, *J* = 2.4 Hz, 3H) ppm; ^{13}C NMR (101 MHz, $CDCl_3$): δ = 205.9, 171.0, 137.9, 131.7, 129.0 (2C), 128.5 (2C), 127.5, 119.1, 78.5, 76.0, 66.1, 62.0, 61.3, 60.9, 53.5, 40.6, 31.9, 14.7, 3.6 ppm; IR (film): $\tilde{\nu}$ = 3028, 2957, 2919, 2807, 1717, 1649, 1495, 1453, 1423, 1348, 1316, 1227, 1186, 1121, 1076, 1059, 1029, 1000, 971, 936, 742, 699 cm^{-1}; HRMS (ESI): *m/z* calcd. for $C_{21}H_{26}NO_3$ [M+H$^+$]: 340.19072, found: 340.19053.

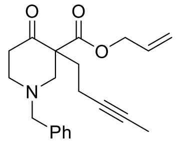

The data are consistent with those reported in the literature.[5]

3-Allyl 1-methyl 4-oxo-3-(pent-3-yn-1-yl)piperidine-1,3-dicarboxylate (S6).[5] Methyl chloroformiate (6.0 mL, 77.65 mmol) was added to a solution of compound **S5** (5.27 g, 15.53 mmol) in toluene (25 mL). The mixture was stirred at 100 °C (oil bath) for 14 h before it was cooled to room temperature and concentrated under reduced pressure. The residue was purified by flash chromatography on silica gel (hexanes/EtOAc, 3:1 to 2:1) to furnish the title compound as a yellow oil (4.70 g, 99%). ^{1}H NMR (400 MHz, $CDCl_3$): δ = 5.86 (ddt, *J* = 16.5, 9.9, 5.8 Hz, 1H), 5.36 – 5.20 (m, 2H), 4.65 – 4.50 (m, 3H), 4.27 – 3.93 (br. s, 1H), 3.73 (s, 3H), 3.39 (br. s, 1H), 3.22 (d, *J* = 13.7 Hz, 1H), 2.68 (ddd, *J* = 14.1, 9.8, 6.3 Hz, 1H), 2.48 (dt, *J* = 14.7, 4.7 Hz, 1H), 2.28 – 2.02 (m, 3H), 1.86 (br. s, 1H), 1.76 – 1.68 (m, 3H) ppm; ^{13}C NMR (101 MHz, $CDCl_3$): δ = 204.0, 169.6, 155.8, 131.3, 119.4, 77.9, 76.6, 66.4, 60.8, 53.2, 50.3, 43.8, 39.7, 31.3, 14.5, 3.6 ppm; IR (film): $\tilde{\nu}$ = 2956, 2920, 2860, 1699, 1650, 1447, 1474, 1413, 1375, 1264, 1238, 1220, 1189, 1130, 1067, 1028, 995, 935, 876, 767, 528 cm^{-1}; HRMS (ESI): *m/z* calcd. for $C_{16}H_{21}NO_5Na$ [M+Na$^+$]: 330.13119, found: 330.13101.

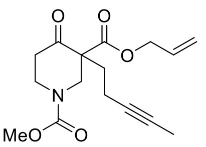

The data are consistent with those reported in the literature.[5]

Methyl 4-oxo-5-(pent-3-yn-1-yl)-3,4-dihydropyridine-1(2*H*)-carboxylate (14).[5] $Pd_2(dba)_3$•$CHCl_3$ (793 mg, 0.77 mmol, 5 mol%) was added to a solution of compound **S6** (4.70 g, 15.31 mmol) in acetonitrile (60 mL). The mixture was stirred at 80 °C (oil bath) for 30 min before it was filtered through a plug of Celite, which was carefully washed with *tert*-butyl methyl ether. The filtrate was concentrated under reduced pressure and the residue was purified by flash chromatography on silica (hexane/EtOAc 3:1 to 1:1) to furnish the title compound as a white solid (2.93 g, 87%). M.p. = 69.8 – 70.5 °C; ^{1}H NMR (400 MHz, $CDCl_3$): δ = 7.77 (br. s, 1H), 3.98 (t, *J* = 7.3 Hz, 2H), 3.85 (s, 3H), 2.57 – 2.51 (m, 2H), 2.35 – 2.28 (m, 2H), 2.24 (dddd, *J* = 7.7, 6.1, 2.9, 2.1 Hz, 2H), 1.74 (t, *J* = 2.5 Hz, 3H) ppm; ^{13}C NMR

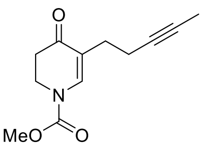

(101 MHz, $CDCl_3$): δ = 193.0, 153.5, 141.0, 117.5, 78.6, 76.7, 54.1, 42.7, 35.9, 27.1, 18.9, 3.5 ppm; IR (film): $\tilde{\nu}$ = 2956, 2919, 2857, 1722, 1662, 1615, 1440, 1399, 1369, 1322, 1300, 1245, 1204, 1174, 1122, 1077, 1049, 1017, 969, 927, 909, 868, 767, 666, 512, 484, 438 cm^{-1}; HRMS (ESI): *m/z* calcd. for $C_{12}H_{15}NO_3Na$ $[M+Na^+]$: 244.09441, found: 244.09442.
The data are consistent with those reported in the literature.[5]

Compound 17. A solution of LiO*t*Bu (384 mg, 4.80 mmol) in THF (9 mL) was added dropwise to a solution of compound **14** (1.02 g, 4.6 mmol) in THF (15 mL) at –50 °C. The resulting red solution was stirred for 10 min before a solution of compound **16** (1.51 g, 3.84 mmol) in THF (8 mL) was added dropwise. The mixture was warmed to room temperature over the course of 5 h and stirring was continued for a further 16 h. Next, 4-dimethyl-aminopyridine (703 mg, 5.75 mmol) was added, followed by di-*tert*-butyl dicarbonate (1.26 g, 5.75 mmol). The mixture was stirred for 1 h at room temperature before sat. aq. NH_4Cl solution (15 mL) was carefully added to quench the reaction. The aqueous layer was extracted with EtOAc (3 x 150 mL) and the combined organic layers were washed with brine (30 mL), dried over anhydrous Na_2SO_4, filtered and concentrated under reduced pressure. The residue was purified by flash chromatography on silica gel (hexanes/EtOAc 10:1 to 8:2) to afford the title compound as a pale yellow gum (1.45 g, 66%). $[\alpha]_D^{20}$ = 51.8 (c = 0.2, $CHCl_3$); ^{1}H NMR (400 MHz, $CDCl_3$) *mixture of rotamers*: δ = 4.63 (dd, *J* = 44.1, 1.9 Hz, 1H), 4.14 – 4.08 (m, 1H), 3.76 (d, *J* = 10.3 Hz, 3H), 3.60 (ddd, *J* = 12.4, 10.0, 2.7 Hz, 1H), 3.49 (ddd, *J* = 14.2, 11.9, 2.5 Hz, 1H), 3.33 – 3.16 (m, 2H), 2.71 (dq, *J* = 24.2, 2.5 Hz, 1H), 2.44 – 2.20 (m, 4H), 2.11 – 1.82 (m, 5H), 1.79 – 1.72 (m, 6H), 1.51 (d, *J* = 1.4 Hz, 10H), 0.88 (s, 9H), 0.14 – 0.07 (m, 6H) ppm; ^{13}C NMR (101 MHz, $CDCl_3$) *mixture of rotamers*: δ = 213.0, 212.4, 170.3, 170.2, 156.5, 156.4, 151.5, 151.4, 84.13, 84.07, 78.1, 78.0, 77.8, 77.6, 76.7, 76.5, 76.2, 69.0, 68.9, 53.5, 53.2, 53.0, 52.0, 51.9, 51.6, 50.4, 50.1, 48.6, 45.8, 45.6, 45.30, 45.28, 40.3, 39.9, 28.4, 28.3, 28.11, 28.10, 25.8, 17.9, 16.8, 16.7, 14.4, 3.74, 3.70, 3.67, 3.6, –4.37, –4.39, –4.65, –4.66 ppm; IR (film) $\tilde{\nu}$ = 2954, 2929, 2858, 1772, 1703, 1448, 1389, 1293, 1255, 1153, 1123, 838, 751 cm^{-1}; HRMS (ESI): *m/z*: calcd. for $C_{33}H_{50}N_2O_7SiNa$ $[M+Na]^+$: 637.32795, found: 637.32768.

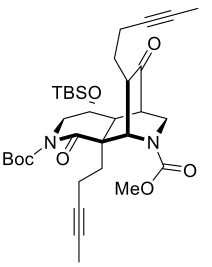

Compound S7. $NaBH_4$ (360 mg, 9.52 mmol) was added in portions to a solution of **S6** (1.45 g, 2.36 mmol) in MeOH (16 mL) at 0 °C (ice bath). The mixture was stirred at this temperature for 1 h until completion of the reaction was reached (TLC). The reaction was quenched with sat. aq. NH_4Cl (30 mL) and the mixture was extracted with EtOAc (3 x 50 mL). The combined organic layers were washed with brine, dried over anhydrous Na_2SO_4, filtered and concentrated under reduced pressure. The residue was purified by flash chromatography on silica gel (pentane/*tert*-butyl methyl ether, 8:2 to 7:3) to afford the title compound as a white foam (992 mg, 68%). $[\alpha]_D^{20}$ = 75.5 (c = 0.3, $CHCl_3$); ^{1}H NMR (400 MHz, $CDCl_3$) *mixture of rotamers*: δ = 4.47 (tdd, *J* = 10.6, 4.1, 2.8 Hz, 1H), 4.16 (dd, *J* = 40.2, 0.9 Hz, 1H), 4.12 – 4.05 (m, 1H), 3.70 (d, *J* = 2.4 Hz, 3H), 3.67 – 3.60 (m, 1H), 3.31 (ddd, *J* = 19.2, 11.4, 3.0 Hz, 1H), 3.16 – 3.05 (m, 2H), 2.36 – 2.10 (m, 5H), 2.04 – 1.92 (m, 1H), 1.79 – 1.58 (m, 11H), 1.53 – 1.44 (m, 10H), 0.87 (s, 9H), 0.09 (d, *J* = 3.4 Hz, 6H) ppm; ^{13}C NMR (101 MHz, $CDCl_3$) *mixture of rotamers*: δ = 171.80, 171.77,

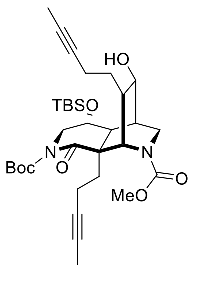

156.8, 156.7, 151.8, 151.6, 83.53, 83.47, 79.2, 79.0, 78.5, 78.0, 76.6, 76.5, 76.0, 75.7, 75.6, 75.4, 67.85, 67.81, 52.9, 52.8, 52.6, 52.4, 52.2, 52.0, 51.9, 51.5, 49.92, 49.89, 48.3, 46.4, 46.2, 40.2, 39.9, 34.4, 34.3, 32.5, 32.4, 28.13, 28.12, 25.90, 25.89, 18.0, 16.6, 16.5, 13.92, 13.89, 3.63, 3.57, 3.55, −4.31, −4.32, −4.5 ppm; IR (film) $\tilde{\nu}$ = 3502, 2928, 2885, 2857, 1766, 1701, 1680, 1449, 1392, 1253, 1117, 1069, 837, 777 cm^{-1}; HRMS (ESI): *m/z*: calcd. for $C_{33}H_{52}N_2O_7SiNa$ $[M+Na]^+$: 639.34360, found: 639.34359.

Compound S8. Triethylamine (1.4 mL, 10.04 mmol), 4-dimethyl-aminopyridine (918 mg, 3.19 mmol) and methanesulfonyl chloride (620 µL, 8.01 mmol) were successively added to a solution of compound **S7** in CH_2Cl_2 (13 mL) at 0 °C (ice bath). After 5 min, the ice bath was removed and the mixture was stirred at room temperature for 2 h before sat. aq. $NaHCO_3$ (10 mL) was added. The aqueous phase was extracted with EtOAc (3 x 50 mL) and the combined organic layers were washed with brine, dried over anhydrous Na_2SO_4, filtered and concentrated under reduced pressure. The residue was purified by flash chromatography on silica gel (hexanes/EtOAc, 9:1 to 6:4) to afford the title compound as a white foam (2.0 g, 90%). $[\alpha]_D^{20}$ = 28.7 (c = 0.5, $CHCl_3$); ^1H NMR (400 MHz, $CDCl_3$) *mixture of rotamers*: δ = 4.49 – 4.40 (m, 1H), 4.30 (dd, *J* = 41.7, 1.4 Hz, 1H), 4.23 – 4.16 (m, 2H), 3.74 (d, *J* = 9.2 Hz, 3H), 3.39 (ddd, *J* = 11.1, 8.5, 2.6 Hz, 1H), 3.29 – 3.14 (m, 2H), 3.04 (d, *J* = 4.8 Hz, 3H), 2.63 – 2.55 (m, 1H), 2.45 – 2.13 (m, 3H), 2.11 – 1.94 (m, 2H), 1.91 – 1.79 (m, 2H), 1.77 – 1.67 (m, 8H), 1.63 – 1.49 (m, 10H), 0.88 (s, 9H), 0.16 – 0.13 (m, 6H) ppm; ^{13}C NMR (101 MHz, $CDCl_3$) *mixture of rotamers*: δ = 170.9, 170.8, 156.6, 151.4, 151.3, 84.6, 84.4, 83.9, 83.8, 78.6, 78.3, 78.2, 77.7, 76.5, 76.4, 76.3, 76.0, 67.91, 67.86, 53.2, 53.1, 51.9, 51.8, 51.7, 51.6, 50.3, 49.9, 49.3, 48.1, 43.0, 42.9, 40.1, 39.7, 39.0, 38.9, 34.2, 33.9, 31.9, 28.2, 28.1, 25.9, 18.1, 16.24, 16.17, 14.05, 14.02, 3.69, 3.68, 3.66, 3.58, −4.11, −4.12, −4.35, −4.36 ppm; IR (film) $\tilde{\nu}$ = 2953, 2930, 2857, 1771, 1701, 1448, 1387, 1366, 1338, 1294, 1255, 1175, 1154, 1120, 837, 778 cm^{-1}; HRMS (ESI): *m/z*: calcd. for $C_{34}H_{54}N_2O_9SSiNa$ $[M+Na]^+$: 717.32115, found: 717.32153.

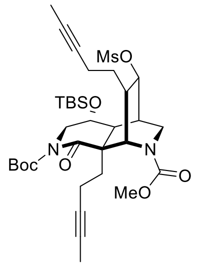

Compound 18. In a flame-dried pressure Schlenk flask, mesylate **S8** (2.85 g, 4.10 mmol) was dissolved in dry 2,6-lutidine (20 mL) and the resulting solution was heated to 170 °C (sand bath temperature) for 5 days. The mixture was cooled to room temperature before it was diluted with CH_2Cl_2 (10 mL). The mixture was then cooled to 0 °C (ice bath) before TBSOTf (4.7 mL, 20.47 mmol) was added. After 5 min, the ice bath was removed and stirring continued at room temperature for 3 h. Sat. aq. $NaHCO_3$ (30 mL) was added at 0 °C, followed, after 5 min, by careful addition of HCl (2 M, 125 mL). After vigorous stirring for 15 min, the mixture was extracted with EtOAc (3 x 100 mL). The combined organic layers were washed with sat. aq. $NaHCO_3$ (50 mL) and dried over anhydrous Na_2SO_4. After filtration and concentration under reduced pressure, the residue was purified by flash chromatography on silica gel (CH_2Cl_2/*tert*-butyl methyl ether, 98:2 to 8:2) to afford the title compound as a white foam (1.63 g, 80%). $[\alpha]_D^{20}$ = 34.7 (c = 0.5, $CHCl_3$); ^1H NMR (400 MHz, $CDCl_3$) *mixture of rotamers*: δ = 6.38 (dd, *J* = 21.8, 6.3 Hz, 1H), 5.93 (dd, *J* = 10.3, 6.6 Hz, 1H), 4.77 (dd, *J* = 45.8, 1.6 Hz, 1H), 3.69 (d, *J* = 17.2 Hz, 3H), 3.38 – 3.29 (m, 1H), 3.20 – 2.91 (m, 4H), 2.82 – 2.69 (m, 1H), 2.45 – 2.09 (m, 6H), 1.92 – 1.79 (m, 2H), 1.75 – 1.66 (m, 6H), 1.65 – 1.60 (m, 1H), 0.88 (s, 9H), 0.07 (d, *J* = 16.5 Hz, 6H) ppm; ^{13}C NMR (101 MHz, $CDCl_3$) *mixture of rotamers*:

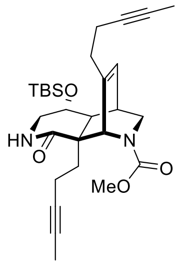

δ = 173.1, 173.0, 156.2, 156.1, 146.5, 145.7, 125.8, 125.2, 78.9, 78.49, 78.46, 78.1, 76.0, 75.8, 75.6, 75.5, 70.8, 70.7, 54.1, 52.6, 52.5, 52.4, 52.2, 51.3, 51.2, 47.2, 47.0, 45.59, 45.57, 39.9, 39.7, 33.7, 33.4, 33.2, 33.1, 25.7, 25.6, 17.9, 16.9, 16.8, 14.1, 3.52, 3.45, 3.44, 3.40, –4.31, –4.33, –4.78, –4.80 ppm; IR (film) $\tilde{\nu}$ = 3204, 2953, 2929, 2894, 2858, 1702, 1667, 1448, 1388, 1258, 1122, 871, 838, 776 cm^{-1}; HRMS (ESI): *m/z*: calcd. for $C_{28}H_{42}N_2O_4SiNa$ $[M+Na]^+$: 521.28061, found: 521.28044.

9-Iodonon-2-yne (S9). In a flame-dried round bottom flask, PPh_3 (3.10 g, 11.81 mmol) was added to CH_2Cl_2 (30 mL), followed by imidazole (1.00 g 14.76 mmol) and iodine (3.75 g, 14.76 mmol). The mixture was cooled to 0 °C using an ice bath before a solution of oct-7-yn-1-ol (1.38 g, 9.84 mmol) in CH_2Cl_2 (10 mL) was added dropwise. The ice bath was removed and stirring continued at room temperature in the dark (the flask was wrapped with aluminum foil) for 2 h. sat. aq. $Na_2S_2O_3$ (40 mL) was added and the mixture was extracted with pentane (3 x 100 mL). The combined organic layers were washed with brine, dried over anhydrous Na_2SO_4, filtered and concentrated under reduced pressure. The residue was purified by flash chromatography on silica gel (pentane/Et_2O, 9:1) to afford the title compound as a colorless oil (2.02 g, 82%). ^1H NMR (400 MHz, $CDCl_3$) δ = 3.18 (t, *J* = 7.0 Hz, 2H), 2.16 – 2.08 (m, 2H), 1.87 – 1.79 (m, 2H), 1.77 (t, *J* = 2.5 Hz, 3H), 1.51 – 1.36 (m, 6H) ppm; ^{13}C NMR (101 MHz, $CDCl_3$) δ = 79.2, 75.7, 33.5, 30.2, 28.9, 27.9, 18.8, 7.2, 3.6 ppm; IR (film) $\tilde{\nu}$ = 2931, 2855, 1458, 1432, 1246, 1198, 1164, 729 cm^{-1}; HRMS (EI): *m/z*: calcd. for $C_9H_{15}I$ $[M]^+$: 250.02129, found: 250.02128.

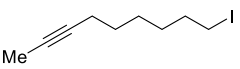

Compound S10. NaH (550 mg, 22.92 mmol) was added in one portion to a solution of amide **18** (1.63 g, 3.27 mmol) and alkyl iodide **S9** (1.69 g, 6.77 mmol) in DMF (13.0 mL) at 0 °C (ice bath). After stirring at this temperature for 1 h, sat. aq. NH_4Cl (20 mL) was carefully added. The mixture was extracted with EtOAc (3 x 10 mL). The combined organic layers were washed with brine (50 mL), dried over anhydrous Na_2SO_4, filtered, and concentrated under reduced pressure. The residue was purified by flash chromatography on silica gel (hexanes/*tert*-butyl methyl ether, 95:5 to 7:3) to afford the title compound as a colorless oil (2.02 g, 99%). $[\alpha]_D^{20}$ = 43.4 (c = 0.5, $CHCl_3$); ^1H NMR (400 MHz, $CDCl_3$) *mixture of rotamers*: δ = 5.94 – 5.87 (m, 1H), 4.84 (dd, *J* = 45.8, 1.6 Hz, 1H), 3.69 (d, *J* = 19.3 Hz, 3H), 3.41 – 3.19 (m, 3H), 3.19 – 3.07 (m, 2H), 3.05 – 2.89 (m, 2H), 2.78 – 2.69 (m, 1H), 2.45 – 1.99 (m, 8H), 1.92 – 1.64 (m, 11H), 1.61 – 1.55 (m, 1H), 1.52 – 1.30 (m, 6H), 1.25 – 1.14 (m, 2H), 0.89 (s, 9H), 0.08 (d, *J* = 12.4 Hz, 6H) ppm; ^{13}C NMR (101 MHz, $CDCl_3$) *mixture of rotamers*: δ = 170.04, 170.02, 156.4, 156.3, 147.0, 146.2, 125.5, 124.9, 79.31, 79.28, 78.8, 78.7, 78.3, 76.1, 75.7, 75.60, 75.57, 75.56, 75.42, 70.8, 70.7, 54.7, 52.9, 52.67, 52.65, 52.5, 52.0, 51.9, 51.1, 47.62, 47.58, 47.3, 47.1, 40.2, 40.0, 33.8, 33.5, 33.2, 33.1, 29.1, 28.74, 28.70, 27.62, 27.59, 26.4, 26.3, 25.80, 25.76, 18.8, 18.0, 17.0, 16.9, 14.2, 3.65, 3.59, 3.57, 3.55, 3.52, –4.17, –4.19, –4.6 ppm; IR (film) $\tilde{\nu}$ = 2929, 2857, 1702, 1646, 1447, 1389, 1257, 1123, 1102, 1088, 871, 838, 777 cm^{-1}; HRMS (ESI): *m/z*: calcd. for $C_{37}H_{56}N_2O_4SiNa$ $[M+Na]^+$: 643.39016, found: 643.39006.

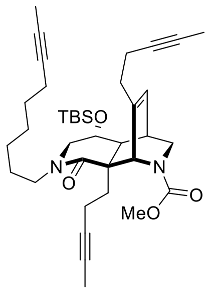

Compound 19. TBAF (1.0 M in THF, 6.5 mL, 6.5 mmol) was added dropwise to a solution of compound **S10** (2.02 g, 3.25 mmol) in THF (13 mL). The mixture was stirred (approximately 1 h) at room temperature until TLC indicated complete conversion. Sat. aq. NH_4Cl (10 mL) was added, the aqueous phase was extracted with EtOAc (3 x 30 mL), and the combined organic layers were washed with brine (40 mL), dried over anhydrous Na_2SO_4, filtered and concentrated under reduced pressure. The residue was purified by flash chromatography on silica gel (hexanes/EtOAc, 7:3 to 3:7) to afford the title compound as a colorless oil (1.62 g, 98 %).

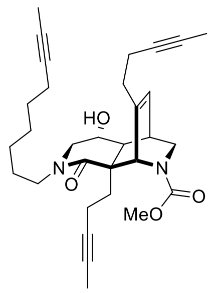

$[\alpha]_D^{20}$ = 87.6 (c = 0.5, CHC l3); ^{1}H NMR (400 MHz, $CDCl_3$) *mixture of rotamers*: δ = 5.99 – 5.92 (m, 1H), 4.83 (dd, *J* = 42.9, 1.6 Hz, 1H), 3.70 (d, *J* = 19.6 Hz, 3H), 3.44 – 3.35 (m, 2H), 3.32 – 3.24 (m, 1H), 3.22 – 3.15 (m, 1H), 3.15 – 3.06 (m, 2H), 3.04 – 2.85 (m, 2H), 2.46 – 2.21 (m, 6H), 2.15 – 2.04 (m, 3H), 1.96 – 1.82 (m, 1H), 1.81 – 1.67 (m, 10H), 1.60 (dd, *J* = 8.9, 2.1 Hz, 1H), 1.50 – 1.29 (m, 6H), 1.26 – 1.16 (m, 2H) ppm; ^{13}C NMR (101 MHz, $CDCl_3$) *mixture of rotamers*: δ = 170.04, 170.01, 156.4, 146.8, 146.0, 125.6, 125.0, 79.33, 79.31, 79.2, 78.8, 78.6, 78.3, 76.2, 75.9, 75.8, 75.7, 75.6, 69.9, 69.8, 54.0, 53.9, 53.1, 52.9, 52.8, 52.6, 52.1, 52.0, 51.1, 47.73, 47.70, 47.4, 47.1, 39.9, 39.7, 33.9, 33.6, 33.3, 33.2, 29.1, 28.71, 28.67, 27.53, 27.50, 26.38, 26.36, 18.8, 17.0, 16.9, 14.3, 3.7, 3.6, 3.54, 3.51 ppm; IR (film) $\tilde{\nu}$ = 3041, 2920, 2857, 1700, 1678, 1642, 1617, 1449, 1392, 1114, 767 cm^{-1}; HRMS (ESI): *m/z*: calcd. for $C_{31}H_{42}N_2O_4Na$ $[M+Na]^+$: 529.30368, found: 529.30402.

Compound S11. In a flame-dried two-necked round bottom flask, alcohol **19** (1.62 g, 3.20 mmol) was dissolved in CH_2Cl_2 (16 mL). The solution was cooled to 0 °C (ice bath) and triethylamine (1.34 mL, 9.61 mmol) was added, followed by 4-dimethyl-aminopyridine (391 mg, 3.20 mmol). Next, chloromethanesulfonyl chloride (6.4 mL, 581 µL) was added dropwise. The mixture was stirred for 30 min at 0 °C before it was stirred at room temperature for another 4 h. The solution was then cooled to 0 °C and the reaction quenched with sat. aq. $NaHCO_3$ (50 mL). The aqueous phase was extracted with EtOAc (4 x 150 mL). The combined organic layers were washed with brine and dried over anhydrous Na_2SO_4, filtered and concentrated under reduced pressure. The orange residue was purified by flash chromatography on silica gel (hexanes/EtOAc, 9:1 to 6:4) to afford the title compound as a pale yellow oil (1.84 g, 93%). $[\alpha]_D^{20}$ = 55.7 (c = 0.5, $CHCl_3$); ^{1}H NMR (400 MHz, $CDCl_3$) *mixture of rotamers*: δ = 6.02 (t, *J* = 7.7 Hz, 1H), 4.87 (dd, *J* = 47.9, 1.6 Hz, 1H), 4.72 – 4.61 (m, 2H), 4.43 (tt, *J* = 9.9, 5.1 Hz, 1H), 3.70 (d, *J* = 18.2 Hz, 3H), 3.58 – 3.44 (m, 2H), 3.42 – 3.34 (m, 1H), 3.22 – 3.10 (m, 2H), 3.08 – 2.86 (m, 2H), 2.44 – 2.20 (m, 5H), 2.15 – 2.04 (m, 3H), 1.98 – 1.86 (m, 2H), 1.82 – 1.64 (m, 10H), 1.49 – 1.31 (m, 6H), 1.25 – 1.17 (m, 2H) ppm; ^{13}C NMR (101 MHz, $CDCl_3$) *mixture of rotamers*: δ = 169.0, 156.25, 156.20, 147.6, 146.8, 125.2, 124.6, 80.2, 79.9, 79.24, 79.22, 78.8, 78.4, 78.3, 78.0, 76.4, 76.3, 76.04, 76.00, 75.6, 54.14, 54.11, 53.2, 53.0, 52.8, 52.7, 52.5, 52.4, 51.0, 48.25, 48.22, 47.89, 47.86, 46.6, 46.4, 40.0, 39.8, 33.6, 33.3, 33.1, 33.0, 29.8, 29.0, 28.61, 28.58, 27.5, 27.4, 26.32, 26.30, 18.7, 16.8, 16.7, 14.3, 3.61, 3.57, 3.55, 3.48, 3.4 ppm; IR (film) $\tilde{\nu}$ = 3005, 2922,2857, 1693, 1644, 1448, 1388, 1350, 1260, 1179, 1113, 945, 889, 765 cm^{-1}; HRMS (ESI): *m/z*: calcd. for $C_{32}H_{43}ClN_2O_6SNa$ $[M+Na]^+$: 641.24226, found: 641.24185.

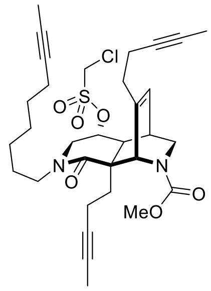

Compound 20. A flame-dried pressure Schlenk flask was charged with a solution of compound **S11** (1.74 g, 2.81 mmol) in toluene (6.0 mL). 1,8-Diazabicyclo[5.4.0]undec-7-ene (12 mL) was added and the solution was stirred at 100 °C (sand bath) until completion of the reaction was reached as monitored by TLC (ca. 3 h). The mixture was allowed to cool to room temperature before distilled water (50 mL) was added. The aqueous phase was extracted with EtOAc (3 x 150 mL), and the combined organic layers were washed with brine, dried over anhydrous Na_2SO_4, filtered and concentrated under reduced pressure. The residue was purified by flash chromatography on silica gel (hexanes/EtOAc, 9:1 to 7:3) to afford the title compound as a colorless oil (1.32 g, 91%). $[\alpha]_D^{20}$ = 39.3 (c = 0.5, $CHCl_3$); ^{1}H NMR (400 MHz, $CDCl_3$) *mixture of rotamers*: δ = 6.06 – 6.00 (m, 1H), 5.70 (dd, *J* = 8.1, 1.3 Hz, 1H), 4.94 – 4.74 (m, 2H), 3.69 (d, *J* = 16.1 Hz, 3H), 3.50 – 3.37 (m, 1H), 3.23 – 3.16 (m, 1H), 3.14 – 3.04 (m, 1H), 2.82 (ddd, *J* = 24.6, 10.0, 2.4 Hz, 1H), 2.66 – 2.56 (m, 1H), 2.32 – 2.04 (m, 10H), 1.75 (t, *J* = 2.5 Hz, 3H), 1.74 – 1.70 (m, 3H), 1.70 – 1.66 (m, 3H), 1.65 – 1.57 (m, 1H), 1.50 – 1.29 (m, 6H), 1.26 – 1.16 (m, 2H) ppm; ^{13}C NMR (101 MHz, $CDCl_3$) *mixture of rotamers*: δ = 168.7, 156.6, 156.5, 146.0, 145.3, 127.9, 127.8, 126.9, 126.4, 106.9, 106.7, 79.25, 79.23, 78.7, 78.5, 78.4, 78.1, 76.2, 75.84, 75.78, 75.76, 75.6, 55.9, 55.6, 53.4, 53.3, 52.7, 52.5, 48.2, 48.1, 47.3, 47.2, 44.0, 43.9, 41.1, 37.8, 37.5, 33.6, 33.5, 29.0, 28.59, 28.58, 28.34, 28.31, 26.32, 26.31, 18.7, 17.0, 16.9, 15.1, 3.58, 3.57, 3.51, 3.48 ppm; IR (film) $\tilde{\nu}$ = 2920, 2858, 1699, 1646, 1448, 1416, 1391, 1341, 1279, 1258, 1191, 1156, 1106, 766, 703 cm^{-1}; HRMS (ESI): *m/z*: calcd. for $C_{31}H_{40}N_2O_3Na$ $[M+Na]^+$: 511.29311, found: 511.29331.

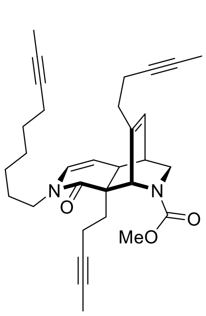

Compound 21. $NaBH_3CN$ (504 mg, 8.0 mmol) was carefully added in three portions to neat formic acid (8 mL) at 0 °C (ice bath). After 5 min, when gas evolution had ceased, a solution of enamide **20** (783 mg, 1.60 mmol) in formic acid (8 mL) was added dropwise at such a rate as to maintain the temperature at 0 °C. After 5 min, the mixture was allowed to reach room temperature. After completion of the reaction (4 h, monitored by TLC), the solution was carefully poured into an ice-cold solution of NaOH (2 M, 150 mL). The pH was adjusted to ≈12 by adding more of NaOH (2 M) before the aqueous phase was extracted with CH_2Cl_2 (3 x 150 mL). The combined organic layers were washed with distilled water (200 mL) and brine (200 mL), before they were dried over anhydrous Na_2SO_4, filtered and concentrated under reduced pressure. The residue was purified by flash chromatography on silica gel (hexanes/EtOAc 9:1 to 7:3) to afford the title compound as a colorless oil (743 mg, 95%). $[\alpha]_D^{20}$ = 77.9 (c = 0.7, $CHCl_3$); ^{1}H NMR (600 MHz, $CDCl_3$) *mixture of rotamers*: δ = 5.93 (dd, *J* = 9.8, 6.5 Hz, 1H), 4.79 (dd, *J* = 61.2, 1.6 Hz, 1H), 3.70 (d, *J* = 27.2 Hz, 3H), 3.39 (dddd, *J* = 13.3, 8.6, 6.7, 1.9 Hz, 1H), 3.28 – 3.17 (m, 2H), 3.15 – 3.02 (m, 2H), 2.91 (ddd, *J* = 47.2, 10.1, 2.6 Hz, 1H), 2.52 (ddq, *J* = 25.7, 6.7, 2.2 Hz, 1H), 2.40 – 2.19 (m, 5H), 2.13 – 2.03 (m, 3H), 2.02 – 1.88 (m, 2H), 1.80 (ddd, *J* = 9.0, 6.8, 1.9 Hz, 1H), 1.76 (t, *J* = 2.5 Hz, 3H), 1.74 (t, *J* = 2.5 Hz, 3H), 1.70 (dt, *J* = 6.9, 2.3 Hz, 3H), 1.67 – 1.61 (m, 1H), 1.49 – 1.31 (m, 7H), 1.28 – 1.17 (m, 2H) ppm; ^{13}C NMR (151 MHz, $CDCl_3$) *mixture of rotamers*: δ = 170.53, 170.52, 156.5, 156.4, 145.8, 145.1, 125.5, 124.9, 79.37, 79.35, 79.0, 78.7, 78.4, 76.1, 75.7, 75.60, 75.56, 75.55, 75.4, 53.95, 53.92, 52.7, 52.5, 52.1, 52.0, 48.1, 48.0, 47.92, 47.85, 45.3, 45.19, 45.18, 45.1, 39.7, 39.6, 37.4, 37.0, 33.3, 33.2, 30.0, 29.9, 29.1, 28.75, 28.72, 27.52, 27.51, 26.53, 26.49, 18.8, 16.94, 16.88, 14.6, 14.5, 3.7, 3.60, 3.55, 3.52 ppm; IR (film) $\tilde{\nu}$ = 2920, 2857,

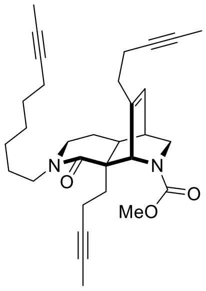

1697, 1632, 1446, 1390, 1337, 1282, 1110, 767 cm^{-1}; HRMS (ESI): *m/z*: calcd. for $C_{31}H_{42}N_2O_3Na$ [M+Na]$^+$: 513.30876, found: 513.30902.

Synthesis of the Quinoline Core

Compound 23. A flame-dried pressure Schlenk flask was charged under Ar with $[Ir(cod)OMe]_2$ (0.5 µmol, 0.3 mg, 1 mol%), Silica-SMAP ligand (1.0 µmol, 14.3 mg, 2 mol%),[6] B_2Pin_2 (14 mg, 0.055 mmol) and degassed (3 freeze-pump-thaw cycles) *tert*-butyl methyl ether (250 µL). A degassed solution of quinoline **22** (25.5 mg, 0.05 mmol)[5] in *tert*-butyl methyl ether (250 µL, 3 freeze-pump-thaw cycles) was then added. The Schlenk flask was closed and the solution stirred at 60 °C (oil bath) overnight (16 h). After this time, all volatile materials were removed under reduced pressure and the residue was purified by flash chromatography on silica gel (hexanes/EtOAc, 9:1 to 6:4) to afford the title compound as a colorless oil (10 mg, 39%). A second fraction contained unreacted starting material **22** (8.2 mg, 32%). Analytical and spectroscopic data of compound **23**: ^{1}H NMR (400 MHz, $CDCl_3$): δ = 8.21 (s, 1H), 8.02 (ddd, *J* = 8.3, 4.9, 1.1 Hz, 2H), 7.66 (ddd, *J* = 8.4, 6.9, 1.3 Hz, 1H), 7.53 (ddd, *J* = 8.3, 6.8, 1.4 Hz, 1H), 5.35 (dt, *J* = 9.1, 4.6 Hz, 1H), 3.84 – 3.63 (m, 5H), 3.61 – 3.56 (m, 1H), 3.16 – 2.95 (m, 3H), 2.05 (ddq, *J* = 14.5, 9.0, 7.3 Hz, 1H), 1.92 – 1.76 (m, 3H), 1.72 – 1.62 (m, 2H), 1.06 (t, *J* = 7.4 Hz, 3H), 0.89 (s, 9H), 0.05 (s, 6H) ppm; ^{13}C NMR (101 MHz, $CDCl_3$): δ = 160.3, 157.5 (q, $^2J_{C-F}$ = 36.4 Hz), 147.1, 142.4, 133.1, 129.9, 129.4, 126.7, 126.6, 123.4, 116.0 (q, $^1J_{C-F}$ = 288.9 Hz), 73.1, 63.1, 40.8, 37.5, 32.9, 31.9, 26.9, 26.3, 26.1 (3C), 18.5, 11.4, −5.1, −5.2 ppm; ^{19}F NMR (282 MHz, $CDCl_3$) δ = −75.9 ppm; IR (film) $\tilde{\nu}$ = 3249, 3076, 2954, 2930, 2884, 2857, 1705, 1563, 1463, 1254, 1210, 1182, 1161, 1099, 836, 761 cm^{-1}; MS (ESI): *m/z:* 513 [M+H]$^+$; HRMS (ESI): *m/z*: calcd. for $C_{26}H_{40}F_3N_2O_3Si$ [M+H]$^+$: 513.27577, found: 513.27548.

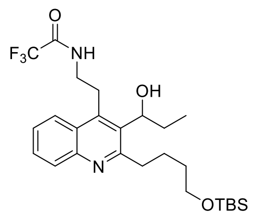

Compound S12. ***Step 1:*** A flame-dried pressure Schlenk flask was charged under Ar with $[Ir(cod)OMe]_2$ (0.7 mg, 2 mol%, 1.0 µmol), 4,4'-di-*tert*-butyl-bipyridine (0.54 mg, 2.0 µmol, 4 mol%), B_2Pin_2 (25.4 mg, 0.1 mmol), and degassed (3 freeze-pump-thaw cycles) *tert*-butyl methyl ether (500 µL). A degassed solution of quinoline **22** (25.5 mg, 0.05 mmol)[5] in *tert*-butyl methyl ether (500 µL, 3 freeze-pump-thaw cycles) was then added. The Schlenk flask was closed and the solution stirred at 60 °C overnight (16 h). After this time, all volatile materials were removed under reduced pressure and the residue was directly engaged in the next step.

Step 2: The residue was dissolved in THF (250 µL) and distilled water (250 µL). $NaIO_4$ (23.0 mg, 0.15 mmol) was then added and the resulting suspension was vigorously stirred under air at room temperature. After 1 h, distilled water (5 mL) was added and the aqueous phase was extracted with EtOAc (3 x 5 mL). The combined organic layers were dried over anhydrous Na_2SO_4. The solution was filtered and concentrated under reduced pressure. The residue was purified by flash chromatography on silica gel (Hexanes/EtOAc 9:1 to 6:4) to afford the title compound as a yellow oil (16 mg, 30.3 µmol, 61%). ^{1}H NMR (600 MHz, $CDCl_3$): δ = 8.30 (br.s, 1H), 7.96 (d, *J* = 9.0 Hz, 1H), 7.85 (s, 1H), 7.37 (dd, *J* = 9.0, 2.5 Hz, 1H), 7.34 (d, *J* = 2.6

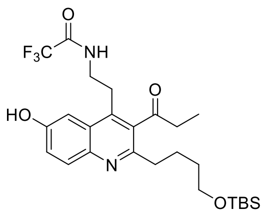

Hz, 1H), 3.60 (t, *J* = 6.4 Hz, 2H), 3.57 (q, *J* = 5.6 Hz, 2H), 3.00 (s, 2H), 2.88 – 2.72 (m, 4H), 1.81 (p, *J* = 7.8 Hz, 2H), 1.55 (dt, *J* = 13.9, 6.5 Hz, 2H), 1.25 (t, *J* = 7.2 Hz, 3H), 0.86 (s, 9H), 0.01 (s, 6H) ppm; ^{13}C NMR (151 MHz, $CDCl_3$): δ = 211.8, 158.2 (q, $^{2}J_{C\text{-}F}$ = 37.4 Hz), 155.7 (d, $^{3}J_{C\text{-}F}$ = 2.5 Hz), 153.7, 143.1, 137.5, 135.9, 131.3, 126.0, 123.0, 115.8 (q, $^{1}J_{C\text{-}F}$ = 287.3 Hz), 105.4, 62.9, 39.8, 39.3, 36.7, 32.8, 29.8, 28.2, 26.5, 26.1 (3C), 18.4, 8.1, –5.2 ppm; ^{19}F NMR (565 MHz, $CDCl_3$) δ = –75.9 ppm; IR (film) $\tilde{\nu}$ = 3305, 3088, 2953, 2929, 2857, 1703, 1620, 1565, 1501, 1461, 1210, 1182, 1161, 1102, 835, 776 cm^{-1}; MS (ESI): *m/z:* 527 [M+H]$^+$; HRMS (ESI): *m/z*: calcd. for $C_{26}H_{38}F_3N_2O_4Si$ [M+H]$^+$: 527.25431, found: 527.25475.

Compound 26. The synthesis of steps 1 to 3 followed a literature-reported procedure.[7]

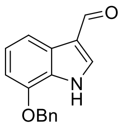

Step 1: $POCl_3$ (2.87 mL, 30.8 mmol) was added dropwise to DMF (30 mL) at 0 °C (ice bath). The resulting mixture was stirred for 5 min at 0 °C before a solution of 7-(benzyloxy)-indole **24** (6.54 g, 29.3 mmol) in DMF (25 mL) was added dropwise. The resulting orange mixture was allowed to come to room temperature and stirring continued in the dark (the flask was covered with aluminum foil) for 4.5 h. After cooling back to 0 °C, aq. KOH solution (5.5 g in 20 mL of distilled water) was added to the mixture, resulting in a change of color from orange to yellow. The mixture was stirred at reflux temperature (oil bath) for 5 min. After cooling to room temperature, the mixture was diluted with EtOAc (300 mL) and the organic layer was washed with water (250 mL) and brine (250 mL). The solvent was removed under reduced pressure to yield aldehyde **S13** as a red solid which was used without any further purification in the following step (7.06 g).

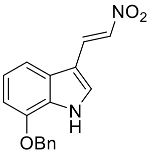

Step 2: Aldehyde **S13** (7.06 g, 28.1 mmol) was dissolved in freshly distilled nitromethane (150 mL) at room temperature. Ammonium acetate (2.40 g, 31.0 mmol) was added and the resulting mixture was stirred at reflux temperature (oil bath) for 3 h. The solution was allowed to cool to room temperature before it was diluted with CH_2Cl_2 (250 mL). The organic layer was washed with distilled water (3 x 100 mL) and brine (200 mL), dried over anhydrous Na_2SO_4, filtered and concentrated under reduced pressure to yield nitroalkene **S14** as an orange solid which was used without any further purification in the following step (7.75 g).

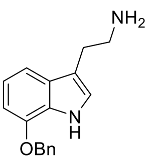

Step 3: $LiAlH_4$ (5.83 g, 153.6 mmol) was suspended in THF (150 mL) at 0 °C (ice bath). A solution of nitroalkene **S14** (7.52 g, 25.6 mmol) in THF (120 mL) was added dropwise to the suspension at such a rate as to keep the reaction temperature at 0 °C. Once the addition was complete, the ice bath was removed and the solution was allowed to warm to room temperature before it was heated to 60 °C (oil bath) for 4.5 h. The mixture was cooled to room temperature and then to 0 °C. Sat. aq. solution of Rochelle salt (100 mL) was carefully added to quench the reaction. The mixture was diluted with EtOAc (150 mL) and the organic layer was separated. The aqueous phase was extracted with EtOAc (2 × 150 mL), the combined organic layers were washed with brine (250 mL), dried over anhydrous Na_2SO_4, filtered and concentrated under reduced pressure to afford amine **S15** as an off-white solid material which was used without further purification in the next step (6.19 g).

Step 4: Amine **S15** (6.19 g, 23.3 mmol) was dissolved in CH_2Cl_2 (140 mL) and the solution was cooled to 0 °C (ice bath) before trifluroacetic acid anhydride (TFAA, 4.86 mL, 34.9 mmol) was added dropwise. Stirring was continued at 0 °C for 2 h. The mixture was then poured into distilled water (50 mL), the aqueous phase was extracted with CH_2Cl_2 (3 × 200 mL), and the combined organic layers were dried over anhydrous Na_2SO_4, filtered and concentrated under reduced pressure. The residue was loaded onto silica to give a solid dry deposit, which was then purified by flash chromatography on silica gel (hexanes/EtOAc, 9:1 to 7:3) to afford the title compound **26** as a brown solid (6.91 g, 65% over four steps). Mp: 135.4 -137.1 °C; ^{1}H NMR (400 MHz, $CDCl_3$): δ 8.34 (s, 1H), 7.52 – 7.45 (m, 2H), 7.45 – 7.34 (m, 3H), 7.21 (d, *J* = 8.0 Hz, 1H), 7.07 (t, *J* = 7.9 Hz, 1H), 7.01 (d, *J* = 2.3 Hz, 1H), 6.77 (d, *J* = 7.7 Hz, 1H), 6.37 (s, 1H), 5.22 (s, 2H), 3.68 (q, *J* = 6.4 Hz, 2H), 3.04 (t, *J* = 6.6 Hz, 2H) ppm; ^{13}C NMR (101 MHz, $CDCl_3$): δ 157.3 (q, $^{2}J_{C\text{-}F}$ = 36.2 Hz), 145.7, 137.0, 128.8 (2C), 128.5, 128.3, 128.0 (2C), 127.4, 122.0, 120.4, 116.0 (q, $^{1}J_{C\text{-}F}$ = 288.4 Hz), 112.3, 111.5, 103.7, 70.4, 40.3, 25.0 ppm; ^{19}F NMR (565 MHz, $CDCl_3$): δ –76.00 ppm; IR (film) $\tilde{\nu}$ = 3426, 3312, 1696, 1372, 1159, 1002, 692, 511 cm^{-1}; HRMS (EI): m/z: calcd. for $C_{19}H_{17}F_3N_2O_2$ [M^+]: 362.12366, found: 362.12412.

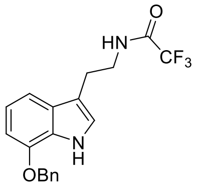

Compound 27. ***Step 1:*** $NaIO_4$ (41.2 g, 192.7 mmol) was dissolved in distilled water (300 mL) and the solution was cooled to 0 °C (ice bath). A solution of indole **26** (7.0 g, 19.2 mmol) in MeOH (600 mL) was added dropwise under vigorous stirring while keeping the reaction temperature at 0 °C. The mixture was warmed to room temperature and then heated to 40 °C (oil bath) for 24 h. After this time, the mixture was cooled to room temperature before it was poured into distilled water (600 mL). The aqueous phase was extracted with CH_2Cl_2 (3 × 500 mL). The combined organic layers were washed with brine (300 mL), dried over anhydrous Na_2SO_4, filtered and concentrated under reduced pressure to afford crude formamide **S16**, which was directly used in the following step.

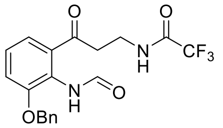

Step 2: Concentrated aq. HCl (37% *w/w*, 0.89 mL) was added to a solution of compound **S16** in MeOH (250 mL) at 0 °C (ice bath) and the resulting mixture was stirred for 6 h at this temperature. An aq. solution of K_2CO_3 (1 M, 25.7 mL) was carefully added until the pH of the solution was ≈6. The mixture was poured into distilled water (150 mL) and the aqueous phase was extracted with CH_2Cl_2 (3 × 250 mL) and then EtOAc (1 × 150 mL). The combined organic layers were dried over anhydrous Na_2SO_4, filtered, and concentrated under reduced pressure. The crude product was purified by flash chromatography (hexanes/EtOAc, 9:1 to 7:3) to obtain title compound **27** as a yellow solid material (4.97 g, 71 % over 2 steps). Mp: 50.1 – 51.0 °C; ^{1}H NMR (600 MHz, $CDCl_3$): δ 7.44 – 7.39 (m, 4H), 7.36 (m, 1H), 7.29 (dd, *J* = 8.4, 1.2 Hz, 1H), 7.20 (s, 1H), 6.93 (dd, *J* = 7.8, 1.2 Hz, 1H), 6.70 (br. s, 2H), 6.57 (dd, *J* = 8.4, 7.7 Hz, 1H), 5.11 (s, 2H), 3.77 (m, 2H), 3.26 (m, 2H) ppm; ^{13}C NMR (151 MHz, $CDCl_3$): δ 200.7 , 157.2 (q, $^{2}J_{C\text{-}F}$ = 36.2 Hz), 146.6, 142.3, 136.5, 128.9 (2C), 128.5, 127.8 (2C), 122.6, 116.7, 116.0 (q, $^{1}J_{C\text{-}F}$ = 288.4 Hz), 115.0, 114.6, 70.9, 38.0, 35.1 ppm; ^{19}F NMR (565 MHz, $CDCl_3$): δ –76.09 ppm; IR (film) $\tilde{\nu}$ = 3294, 2960, 2919, 1682, 1480,1148,1010,696 cm^{-1}; HRMS (ESIneg): *m/z*: calcd. for $C_{18}H_{16}F_3N_2O_3$ [M-H]$^-$: 365.11185, found: 365.11164.

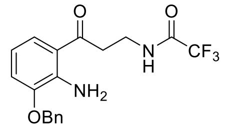

Compound 28. A solution of Meldrum's acid (4.41 g, 30.6 mmol) in CH_2Cl_2 (40 mL) was cooled to 0 °C (ice bath).

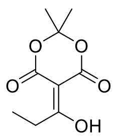

Pyridine (5.0 mL, 61.2 mmol) was added, followed by a dropwise addition of propionic acid (2.90 mL, 33.7 mmol). The solution was then allowed to reach room temperature. After 1 h, aq. HCl (2 M, 10 mL) was added, and the organic layer was separated. The aqueous phase was extracted with CH_2Cl_2 (3 × 50 mL). The combined organic layers were washed with distilled water (50 mL) and brine (50 mL) before being dried over anhydrous $MgSO_4$, filtered, and concentrated under reduced pressure. The title compound was obtained as a red solid material, which was used without further purification (3.40 g, 56%). ^{1}H NMR (400 MHz, $CDCl_3$): δ 15.39 (s, 1H), 3.09 (q, *J* = 7.4 Hz, 2H), 1.72 (s, 6H), 1.24 (t, *J* = 7.4 Hz, 3H) ppm. The data are consistent with those reported in the literature.[8]

Compound 29. Compound **28** (1.67 g, 8.34 mmol) was added to a solution of aniline **27** (2.10 g, 5.62 mmol) in

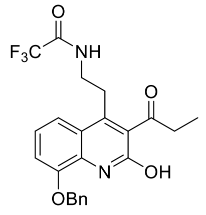

toluene (20 mL) at room temperature. The mixture was then stirred at reflux temperature (oil bath) for 3 h before being allowed to cool to room temperature. Silica was added to the mixture before removing the solvent under reduced pressure in order to obtain a dry solid deposit. This adsorbate was deposed on silica gel, which was wetted with toluene and left for 24 h. After this time, the product was eluted (hexanes/acetone, 3:0.1 to 0:1) to afford the title compound as a yellow solid (2.19 g, 87%). Mp: 61.5-62.6 °C (decomp.); ^{1}H NMR (600 MHz, MeOD): δ 7.63 (dd, *J* = 7.6, 1.8 Hz, 1H), 7.51 (m, 2H), 7.39 (m, 2H), 7.33 (tt, *J* = 7.3, 1.3 Hz, 1H), 7.26 (dd, *J* = 8.0, 1.8 Hz, 1H), 7.24 (t, *J* = 8.1 Hz, 1H), 5.35 (s, 2H), 3.57 (dd, *J* = 8.5, 6.6 Hz, 2H), 3.01 (m, 2H), 2.91 (q, *J* = 7.2 Hz, 2H), 1.17 (t, *J* = 7.2 Hz, 3H) ppm (*the labile protons from the amide and alcohol are not seen due to exchange with MeOD*); ^{13}C NMR (151 MHz, MeOD): δ 208.0, 161.6, 159.3 (q, $^2J_{C\text{-}F}$ = 37.8 Hz), 147.1, 146.6, 137.8, 135.2, 130.0, 129.7 (2C), 129.4, 128.9 (2C), 124.2, 121.0, 118.4, 117.4 (q, $^1J_{C\text{-}F}$ = 286.9 Hz), 114.3, 72.0, 40.9, 37.9, 30.1, 7.9 ppm; ^{19}F NMR (565 MHz, MeOD): δ –77.45 ppm; IR (film) $\tilde{\nu}$ = 3251, 1636, 1455, 1151, 692 cm^{-1}; HRMS (ESI): *m/z*: calcd. for $C_{23}H_{21}F_3N_2O_4Na$ [M+Na$^+$]: 469.13456, found: 469.13472.

Compound 30. A solution of quinoline **29** (3.30 g, 7.4 mmol) in pyridine (40 mL) was cooled to 0 °C (ice bath)

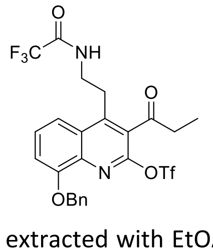

before Tf_2O (2.5 mL, 14.9 mmol) was added dropwise. The mixture was stirred for 10 min at 0 °C before the ice bath was removed and stirring was continued for 1.75 h [*Note*: the reaction time is an important parameter to limit the formation of the corresponding vinyl triflate side-product, which is difficult to separate from the desired product]. The solution was then poured into a sat. aq. solution of $NaHCO_3$ (150 mL). The aqueous phase was extracted with EtOAc (3 × 200 mL) and the combined organic layers were poured into aq. HCl (0.1 M, 240 mL). The layers were separated, and the aqueous phase was further extracted with CH_2Cl_2 (100 mL). The combined organic layers were carefully neutralized with sat. aq. $NaHCO_3$ solution (150 mL) and then washed with distilled water (150 mL) and brine (150 mL); they were dried over anhydrous Na_2SO_4, filtered and concentrated under reduced pressure. Purification of the residue by flash chromatography on silica gel (CH_2Cl_2/MeOH, 100:0 to 97:3) yielded the title compound as a light-yellow solid (2.21 g, 78%). Mp: 42.3 – 44.5 °C; ^{1}H NMR (600 MHz, $CDCl_3$): δ 7.75 (dd, *J* = 8.6, 1.0 Hz, 1H), 7.66 (t, *J* = 4.9 Hz, 1H), 7.63 (dd, *J* = 8.6, 7.8 Hz, 1H), 7.55 (m, 2H), 7.41 (m, 2H), 7.34 (m, 1H), 7.28 (dd, *J* = 7.9, 1.0 Hz, 1H), 5.34 (s, 2H), 3.75 (td, *J* = 6.7, 4.9 Hz, 2H), 3.25 (t, *J* = 6.8 Hz, 2H), 2.96 (q, *J* =

7.2 Hz, 2H), 1.28 (t, J = 7.2 Hz, 3H) ppm; ^{13}C NMR (151 MHz, $CDCl_3$): δ 205.2, 158.0 (q, $^{2}J_{C\text{-}F}$ = 37.8 Hz), 155.1, 148.7, 147.6, 138.0, 136.4, 129.4, 128.7 (2C), 128.2, 127.8, 127.3 (2C), 126.5, 118.3 (q, $^{1}J_{C\text{-}F}$ = 466.6 Hz), 116.2, 116.3 (q, $^{1}J_{C\text{-}F}$ = 431.9 Hz), 113.5, 71.4, 40.2, 38.5, 28.9, 8.1 ppm; ^{19}F NMR (565 MHz, $CDCl_3$): δ −71.9, −76.0 ppm; IR (film) $\tilde{\nu}$ = 3302, 2922, 1705, 1419, 1174, 999, 695 cm^{-1}; HRMS (ESI): *m/z*: calcd. for $C_{24}H_{19}F_6N_2O_6S$ [M-H]$^-$: 577.08736, found: 577.08765.

(But-3-en-1-yloxy) (*tert*-butyl) dimethyl silane (31). 3-Butenol (6.88 mL, 80 mmol) was dissolved in CH_2Cl_2 (220

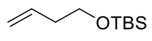

mL) and the solution cooled to 0 °C (ice bath) before imidazole (13.61 g, 200 mmol) and TBDMSCl (13.26 g, 88 mmol) were successively added. The mixture was stirred for 1 h at 0 °C before the reaction was quenched with sat. aq. NH_4Cl (4 mL). The aqueous phase was extracted with CH_2Cl_2 (3 × 60 mL). The combined organic layers were dried over anhydrous Na_2SO_4 and filtered. The solvent was evaporated under reduced pressure to give the title compound as a colorless oil, which was used in the next step without further purification (7.87 g, 53%). ^{1}H NMR (400 MHz, $CDCl_3$): δ 5.85 – 5.71 (m, 1H), 5.08 – 4.94 (m, 2H), 3.63 (t, J = 6.8 Hz, 2H), 2.29 – 2.18 (m, 2H), 0.90 – 0.85 (m, 9H), 0.03 (s, 6H). The data are consistent with those reported in the literature.[9]

Compound 32. In a flame-dried pressure Schlenk flask, a solution of 9-H-9-BBN dimer (858 mg, 3.51 mmol) in

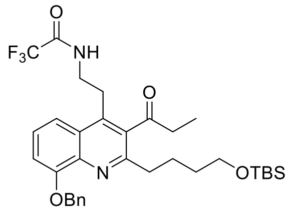

THF (30 mL) was added to neat (but-3-en-1-yloxy)(*tert*-butyl)dimethylsilane **31** (1.61 g, 8.67 mmol), and the resulting mixture was stirred under Ar overnight (16 h) at room temperature. NaOAc (950 mg, 11.58 mmol) was added, followed by distilled water (5.2 mL) and a solution of quinoline triflate **30** (3.35 g, 5.78 mmol) in THF (30 mL). Finally, $Pd(PPh_3)_4$ (340 mg, 0.29 mmol, 5 mol%) was added and a gentle stream of Ar was bubbled through the resulting solution for 10 min at room temperature. The pressure Schlenk flask was then sealed and the mixture stirred at 80 °C (oil bath) for 1 h. After reaching ambient temperature, distilled water was added (20 mL), followed by *tert*-butyl methyl ether (20 mL). The biphasic mixture was extracted with *tert*-butyl methyl ether (3 x 40 mL). The combined organic layers were washed with brine (100 mL) and dried over anhydrous Na_2SO_4, filtered, and concentrated under reduced pressure. The residue was purified by flash chromatography on silica gel (toluene/EtOAc, 100:0 to 95:5) to afford the title compound as a white waxy solid (3.13 g, 88%). ^{1}H NMR (600 MHz, $CDCl_3$): δ = 7.69 (br. s, 1H), 7.59 (dd, J = 8.6, 1.1 Hz, 1H), 7.53 (ddq, J = 7.6, 1.3, 0.7 Hz, 2H), 7.44 (dd, J = 8.5, 7.7 Hz, 1H), 7.41 – 7.35 (m, 2H), 7.34 – 7.28 (m, 1H), 7.11 (dd, J = 7.8, 1.1 Hz, 1H), 5.43 (s, 2H), 3.71 – 3.65 (m, 2H), 3.63 (t, J = 6.4 Hz, 2H), 3.13 (br. s, 2H), 2.91 (t, J = 7.8 Hz, 2H), 2.84 (q, J = 7.8 Hz, 2H), 1.95 – 1.87 (m, 2H), 1.64 – 1.58 (m, 2H), 1.28 (t, J = 7.2 Hz, 3H), 0.88 (s, 9H), 0.03 (s, 6H) ppm; ^{13}C NMR (151 MHz, $CDCl_3$): δ = 211.9, 157.8 (q, $^{2}J_{C\text{-}F}$ = 37.2 Hz), 155.5, 154.9, 140.5, 138.8, 137.2, 136.0, 128.7 (2C), 127.9, 127.3, 127.1 (2C), 125.9, 115.9 (q, $^{1}J_{C\text{-}F}$ = 287.9 Hz), 115.7, 111.9, 71.3, 63.0, 40.1, 39.3, 37.3, 32.7, 28.3, 26.1 (3C), 26.0, 18.5, 8.2, −5.2 (2C) ppm; ^{19}F NMR (565 MHz, $CDCl_3$): δ = −75.9 ppm; IR (film) $\tilde{\nu}$ = 3300, 2928, 2857, 1703, 1563, 1462, 1208, 1159, 835, 731 cm^{-1}; HRMS (ESI): *m/z*: calcd. for $C_{33}H_{44}F_3N_2O_4Si$ [M+H]$^+$: 617.30170, found: 617.30095.

Compound S17. Quinoline **32** (1.40 g, 2.27 mmol) was dissolved in EtOAc (20 mL) and Pd/C (10% *w/w,* 242 mg) was added. A balloon filled with H_2 gas was attached to the flask via a needle through a rubber cap and H_2 was bubbled through the suspenion for 10 min. The mixture was stirred for 1 h under H_2 atm (1 bar) before it was filtered through a plug of Celite. The filtrate was evaporated and the residue purified by flash chromatography on silica gel (hexanes/EtOAc, 10:0 to 7:3) to afford the title compound as a pale yellow oil (1.20 g, quant.). ^{1}H NMR (400 MHz, $CDCl_3$): δ = 8.38 (br. s, 1H), 7.55 (s, 1H), 7.53 – 7.45 (m, 2H), 7.21 (dd, *J* = 4.9, 3.8 Hz, 1H), 3.77 – 3.61 (m, 4H), 3.14 (br. s, 2H), 2.90 – 2.78 (m, 4H), 1.99 – 1.86 (m, 2H), 1.65 – 1.54 (m, 2H), 1.28 (t, *J* = 7.2 Hz, 3H), 0.89 (s, 9H), 0.05 (s, 6H) ppm; ^{13}C NMR (101 MHz, $CDCl_3$): δ = 211.1, 157.8 (q, $^{2}J_{C\text{-}F}$ = 37.3 Hz), 154.5, 152.5, 140.1, 137.4, 136.4, 128.6, 125.1, 115.8 (q, $^{1}J_{C\text{-}F}$ = 288.7 Hz), 113.8, 111.2, 62.8, 40.1, 39.3, 36.2, 32.5, 28.4, 26.1 (3C), 25.5, 18.5, 8.1, –5.2 (2C) ppm; ^{19}F NMR (282 MHz, $CDCl_3$): δ = –75.9 ppm; IR (film) $\tilde{\nu}$ = 3324, 2929, 2858, 1705, 1568, 1497, 1467, 1250, 1209, 1178, 1104, 836, 776 cm^{-1}; HRMS (ESI): *m/z*: calcd. for $C_{26}H_{38}F_3N_2O_4Si$ $[M+H]^+$: 527.25475, found: 527.25510.

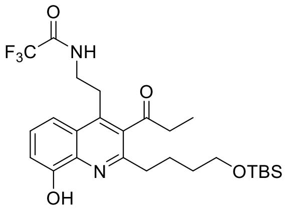

Compound S18. Quinoline **S17** (3.49 mmol, 1.84 g) was dissolved in acetonitrile (35 mL). The solution was cooled to 0 °C (ice bath) before di-*tert*-butyldicarbonate (800 µL, 3.66 mmol) was added, followed by 1-methylimidazole (28 µL, 0.35 mmol). The solution was allowed to come to room temperature and stirring was continued until TLC showed complete conversion of the substrate (ca. 5 h). Sat. aq. NH_4Cl solution (20 mL) was added and the mixture was extracted with EtOAc (3 x 20 mL). The combined organic layers were washed with brine (50 mL), dried over anhydrous Na_2SO_4, filtered and concentrated under reduced pressure. The residue was purified by flash chromatography on silica gel (hexanes/EtOAc, 10:0 to 8:2) to afford the title compound as a pale yellow oil, which slowly solidified upon standing (2.05 g, 93%). M.p.: 114.5 – 115.5 °C; ^{1}H NMR (400 MHz, $CDCl_3$): δ = 7.94 (dd, *J* = 7.7, 2.1 Hz, 1H), 7.66 (t, *J* = 5.0 Hz, 1H), 7.59 – 7.47 (m, 2H), 3.66 – 3.54 (m, 4H), 3.10 (br. s, 2H), 2.86 – 2.73 (m, 4H), 1.94 – 1.80 (m, 2H), 1.65 – 1.52 (m, 11H), 1.25 (t, *J* = 7.2 Hz, 3H), 0.88 (s, 9H), 0.03 (s, 6H) ppm; ^{13}C NMR (101 MHz, $CDCl_3$): δ = 211.0, 157.9 (q, $^{2}J_{C\text{-}F}$ = 37.5 Hz), 156.7, 152.1, 147.7, 140.9, 139.1, 136.3, 126.8, 126.0, 121.8, 121.7, 115.8 (q, $^{1}J_{C\text{-}F}$ = 288.7 Hz), 83.6, 63.1, 62.9, 40.2, 39.2, 37.0, 32.8, 27.8 (3C), 26.0 (3C), 25.3, 18.4, 8.0, –5.2 (2C) ppm; ^{19}F NMR (282 MHz, $CDCl_3$): δ = –75.9 ppm; IR (film) $\tilde{\nu}$ = 3325, 2932, 2858, 1760, 1705, 1556, 1463, 1370, 1248, 1150, 836, 775 cm^{-1}; HRMS (ESI): *m/z*: calcd. for $C_{31}H_{45}F_3N_2O_6SiNa$ $[M+Na]^+$: 648.28912, found: 649.28932.

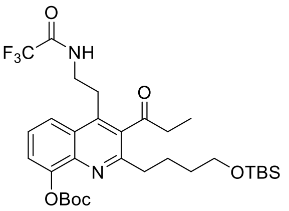

Compound 33. $PhNTf_2$ (1.2 eq., 1.94 mmol, 694 mg) was added to a solution of quinoline **S18** (1.01 g, 1.62 mmol) in THF (32 mL) at –78 °C (dry ice/acetone bath). After 5 min, a solution of KHMDS (1.94 g, 9.71 mmol) in THF (10 mL) was slowly added at such a rate as to keep the temperature below –75 °C (internal thermometer) to prevent any decomposition. Once the addition was complete, stirring was continued for 30 min at –78 °C before $PhNTf_2$ (173 mg, 0.49 mmol) was added in one portion. The solution was stirred for a further 15 min at –78 °C before it was directly poured into an ice-cold sat. aq. $NaHCO_3$ solution (20 mL). The resulting mixture was extracted with EtOAc (3 x 20 mL). The combined organic layers were washed with brine

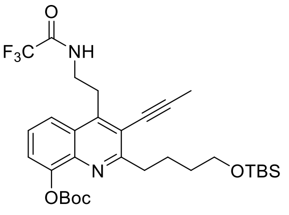

(50 mL) and dried over anhydrous Na_2SO_4, filtered and concentrated under reduced pressure. The residue was purified by flash chromatography on silica gel (hexanes/EtOAc, 10:0 to 7:3) to afford the title compound as a pale yellow oil (974 mg, 99%). ^{1}H NMR (400 MHz, $CDCl_3$): δ = 7.81 (dd, *J* = 8.1, 1.7 Hz, 1H), 7.51 – 7.41 (m, 2H), 6.66 (t, *J* = 6.1 Hz, 1H), 3.70 – 3.61 (m, 4H), 3.54 (t, *J* = 6.7 Hz, 2H), 3.16 – 3.08 (m, 2H), 2.18 (s, 3H), 1.94 – 1.82 (m, 2H), 1.71 – 1.60 (m, 2H), 1.60 (s, 9H), 0.89 (s, 9H), 0.05 (s, 6H) ppm; ^{13}C NMR (101 MHz, $CDCl_3$): δ = 163.2, 157.6 (q, $^2J_{C\text{-}F}$ = 37.3 Hz), 152.2, 147.8, 145.4, 139.2, 126.4, 126.2, 121.22, 121.17, 119.0, 115.9 (q, $^1J_{C\text{-}F}$ = 289.0 Hz), 97.1, 83.5, 75.7, 63.4, 40.0, 37.9, 33.0, 29.5, 27.9 (3C), 26.1 (3C), 24.8, 18.5, 4.7, –5.1 (2C) ppm; ^{19}F NMR (282 MHz, $CDCl_3$): δ = –76.0 ppm; IR (film) $\tilde{\nu}$ = 3333, 2954, 2931, 2859, 1763, 1721, 1278, 1250, 1211, 1153, 1102, 837, 775 cm^{-1}; HRMS (ESI): *m/z*: calcd. for $C_{31}H_{43}F_3N_2O_5SiNa$ $[M+Na]^+$: 631.27855, found: 631.27828.

Compound S19. A solution of quinoline **33** (1.12 mg, 1.84 mmol) in acetonitrile (18 mL) was cooled to 0 °C (ice bath) before di-*tert*-butyldicarbonate (445 µL, 1.94 mmol) was added followed by 4-dimethyl-aminopyridine (225 mg, 1.84 mmol). The mixture was warmed to room temperature and stirring was continued for 1.5 h. Methanol (20 mL) was then added followed by sat. aq. Na_2CO_3 (16.0 mL). The resulting suspension was vigorously stirred for 1 h before sat. aq. NH_4Cl (20 mL) was added. The mixture was extracted with EtOAc (3 x 20 mL), the combined organic layers were washed with brine (50 mL) and dried over anhydrous Na_2SO_4, filtered and concentrated under reduced pressure. The residue was purified by flash chromatography on silica gel (hexanes/EtOAc, 10:0 to 7:3) to afford the title compound as a pale yellow oil (1.10 g, 98%). ^{1}H NMR (400 MHz, $CDCl_3$): δ = 7.97 – 7.91 (m, 1H), 7.50 – 7.38 (m, 2H), 4.67 (br. s, 1H), 3.66 (t, *J* = 6.8 Hz, 2H), 3.50 (br. s, 4H), 3.16 – 3.08 (m, 2H), 2.19 (s, 3H), 1.93 – 1.83 (m, 2H), 1.70 – 1.63 (m, 2H), 1.60 (s, 9H), 1.44 (s, 9H), 0.89 (s, 9H), 0.05 (s, 6H) ppm; ^{13}C NMR (101 MHz, $CDCl_3$): δ = 163.0, 156.1, 152.2, 147.5, 147.1, 139.0, 127.2, 125.8, 122.1, 120.8, 118.6, 96.3, 83.3, 79.4, 75.9, 63.4, 40.5, 37.8, 33.1, 31.2, 28.6 (3C), 27.9 (3C), 26.1 (3C), 24.8, 18.5, 4.9, –5.1 (2C) ppm; IR (film) $\tilde{\nu}$ = 3348, 2954, 2930, 2858, 1762, 1711, 1509, 1470, 1367, 1275, 1248, 1152, 1102, 837, 775 cm^{-1}; HRMS (ESI): *m/z*: calcd. for $C_{34}H_{53}N_2O_6SiNa$ $[M+H]^+$: 613.36674, found: 613.36638.

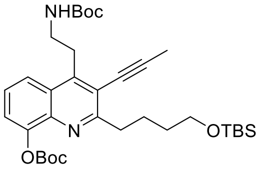

Compound S20. Quinoline **S19** (1.10 g, 1.81 mmol) was dissolved in THF (3.5 mL) in a Teflon flask. The solution was cooled to 0 °C (ice bath) before pyridine (2.3 mL) was added, followed by HF•Py (70/30 *w/w*, 2.3 mL, 17.9 mmol). The solution was stirred at 0 °C for 3.5 h before it was poured into a sat. aq. $NaHCO_3$ solution (20 mL). The mixture was extracted with EtOAc (3 x 20 mL), and the combined organic layers were washed with brine (50 mL) and dried over anhydrous Na_2SO_4, filtered and concentrated under reduced pressure. The residue was purified by flash chromatography on silica gel (hexanes/acetone, 9:1 to 6:4) to afford the title compound as a pale yellow oil (849 mg, 94%). ^{1}H NMR (400 MHz, $CDCl_3$): δ = 7.95 (d, *J* = 8.1 Hz, 1H), 7.51 – 7.39 (m, 2H), 4.68 (br. s, 1H), 3.65 (t, *J* = 6.5 Hz, 2H), 3.46 (br. s, 4H), 3.16 (t, *J* = 7.5 Hz, 2H), 2.20 (s, 3H), 1.96 (p, *J* = 7.5 Hz, 2H), 1.80 – 1.64 (m, 3H), 1.60 (s, 9H), 1.43 (s, 9H) ppm; ^{13}C NMR (101 MHz, $CDCl_3$): δ = 162.8, 156.1, 152.2, 147.44, 147.37, 139.0, 127.2, 125.9, 122.2, 121.0, 118.7, 96.6, 83.5, 79.4, 75.8, 62.7, 40.5, 37.3, 32.7, 31.3, 28.6 (3C), 27.9 (3C), 24.1, 5.0 ppm; IR (film) $\tilde{\nu}$ = 3333, 2977, 2932, 1761, 1695, 1368, 1276, 1248, 1151 cm^{-1}; HRMS (ESI): *m/z*: calcd. for $C_{28}H_{39}N_2O_6$ $[M+H]^+$: 499.28026, found: 499.28043.

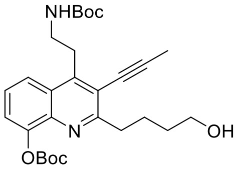

Compound 34. A solution of quinoline **S20** (849 mg, 1.70 mmol) in CH_2Cl_2 (17 mL) was cooled to 0 °C (ice bath) before triethylamine (1.40 mL, 10.2 mmol) was added followed by DMSO (600 µL, 8.5 mmol) and pyridine•SO_3 complex (1.08 g, 6.80 mmol). The resulting solution was stirred under Ar for 10 min at 0 °C and at room temperature for another 3 h. Sat. aq. $NaHCO_3$ solution (10 mL) was added and the resulting mixture was extracted with EtOAc (3 x 10 mL). The combined organic layers were washed with brine (20 mL) and dried over anhydrous Na_2SO_4, filtered and concentrated under reduced pressure. The residue was purified by flash chromatography on silica gel (hexanes/acetone, 10:0 to 7:3) to afford the title compound as a white solid (558 mg, 66%). M.p.: 141.0 – 142.0 °C; ^{1}H NMR (600 MHz, $CDCl_3$): δ = 9.79 (t, *J* = 1.7 Hz, 1H), 7.96 (d, *J* = 8.4 Hz, 1H), 7.47 (t, *J* = 7.9 Hz, 1H), 7.43 (dd, *J* = 7.5, 1.4 Hz, 1H), 4.71 (s, 1H), 3.46 (d, *J* = 3.1 Hz, 4H), 3.16 (t, *J* = 7.4 Hz, 2H), 2.57 (td, *J* = 7.3, 1.7 Hz, 2H), 2.23 (p, *J* = 7.3 Hz, 2H), 2.20 (s, 3H), 1.60 (s, 9H), 1.43 (s, 9H) ppm; ^{13}C NMR (151 MHz, $CDCl_3$): δ = 202.7, 161.7, 156.1, 152.2, 147.4 (2C), 139.0, 127.3, 126.0, 122.2, 121.1, 118.7, 96.9, 83.5, 79.4, 75.7, 43.5, 40.5, 36.6, 31.2, 28.6 (3C), 27.9 (3C), 20.5, 4.9 ppm; IR (film) $\tilde{\nu}$ = 3408, 2978, 2934, 1760, 1712, 1368, 1276, 1247, 1151 cm^{-1}; HRMS (ESI): *m/z*: calcd. for $C_{28}H_{37}N_2O_6$ $[M+H]^+$: 497.26461, found: 497.26469.

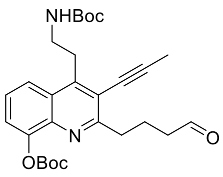

Assembly and Concurrent Macrocyclization by dRCAM

Compound 35. ***Step 1:*** L-Selectride (1.0 M solution in THF, 1.11 mmol, 1.11 mL) was added under Ar to a solution of compound **21** (109 mg, 0.22 mmol) in THF (1.5 mL), and the resulting mixture was stirred at 40 °C (bath temperature) for 24 h under Ar. The solution was then cooled to 0 °C (ice bath) and the reaction was carefully quenched with MeOH (5 mL) and distilled water (5 mL). The ice bath was removed and the solution was vigorously stirred for 15 min before it was filtered through an amino cartridge (Bond Elut-NH_2, Agilent, 500 mg, 3 mL, 40 µm, pre-equilibrated with MeOH, distilled water and a mixture of MeOH/water 8:2, 3 column-volumes each), using MeOH/water 8:2 as the eluent. The methanol was removed under reduced pressure and the resulting aqueous phase was extracted with EtOAc (3 x 20 mL). The combined organic layers were washed with brine (40 mL), dried over anhydrous Na_2SO_4, filtered and concentrated under reduced pressure to afford a pale yellow oil, which was used in the next step without further purification.

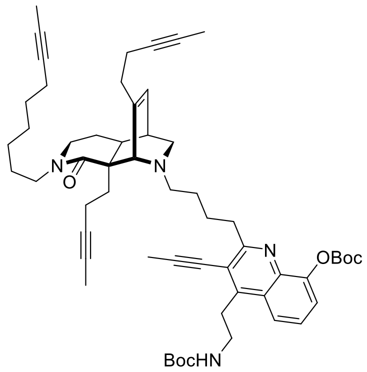

Step 2: Aldehyde **34** (132 mg, 0.27 mmol) was added in one portion to a solution of the crude residue obtained in Step 1 in CH_2Cl_2 (3 mL). The solution turned bright yellow while it was stirred for 15 min under Ar at room temperature. $NaBH(OAc)_3$ (64 mg, 0.29 mmol) was then added and stirring was continued for 1.5 h at room temperature under Ar. After completion of the reaction, sat. aq. $NaHCO_3$ (3 mL) was added to quench the reaction. The resulting solution was transferred into a separatory funnel and the aqueous phase was extracted with EtOAc (3 x 10 mL). The combined organic layers were washed with brine (20 mL), dried over anhydrous Na_2SO_4, filtered and concentrated under reduced

pressure to afford a pale yellow foam which was purified by preparative HPLC (Nucleodur C18 HTEC 10 µM, 250 mm x 40 mm, CH_3CN/H_2O 95:05, 75 mL/min, λ = 260 nm, t = 19.6 min) to provide the title compound as an off-white foam (117 mg, 58%). $[\alpha]_D^{20}$ = −19.4 (c = 0.95, $CHCl_3$); ^1H NMR (600 MHz, CD_2Cl_2): δ = 7.98 (d, *J* = 8.5 Hz, 1H), 7.47 (dd, *J* = 8.5, 7.4 Hz, 1H), 7.40 (dd, *J* = 7.4, 1.2 Hz, 1H), 5.91 (dd, *J* = 6.6, 1.8 Hz, 1H), 4.86 – 4.77 (m, 1H), 3.50 – 3.42 (m, 4H), 3.39 – 3.32 (m, 2H), 3.17 – 3.14 (m, 1H), 3.13 – 3.10 (m, 2H), 3.09 – 3.07 (m, 1H), 3.06 – 3.03 (m, 1H), 3.03 – 3.00 (m, 1H), 2.50 (ddd, *J* = 11.5, 9.2, 6.8 Hz, 1H), 2.27 (dq, *J* = 6.1, 2.0 Hz, 1H), 2.25 – 2.20 (m, 5H), 2.20 – 2.13 (m, 4H), 2.09 (dtt, *J* = 6.7, 4.1, 2.1 Hz, 4H), 1.90 – 1.85 (m, 1H), 1.84 – 1.77 (m, 3H), 1.75 (t, *J* = 2.6 Hz, 3H), 1.72 (t, *J* = 2.3 Hz, 3H), 1.71 (d, *J* = 2.6 Hz, 1H), 1.69 (d, *J* = 2.3 Hz, 1H), 1.66 (t, *J* = 2.5 Hz, 3H), 1.58 (s, 9H), 1.54 – 1.49 (m, 1H), 1.48 – 1.44 (m, 2H), 1.42 (s, 11H), 1.39 – 1.31 (m, 4H), 1.25 – 1.20 (m, 2H) ppm; ^{13}C NMR (151 MHz, CD_2Cl_2): δ = 172.0, 163.5, 156.2, 152.2, 147.9, 147.6, 143.3, 139.3, 127.4, 125.8, 123.8, 122.4, 120.9, 119.0, 97.0, 83.4, 79.9, 79.5, 79.4, 79.3, 76.0, 75.7, 75.5, 75.1, 62.9, 58.4, 55.7, 52.5, 47.9, 45.6, 44.6, 40.8, 39.6, 38.4, 38.2, 35.0, 31.5, 30.0, 29.5, 29.15, 29.06, 28.5 (3C), 27.9 (3C), 27.8, 26.8, 26.6, 19.0, 16.7, 15.0, 4.9, 3.54, 3.51, 3.50 ppm; IR (film) $\tilde{\nu}$ = 3331, 2931, 2858, 1760, 1707, 1633, 1490, 1454, 1367, 1275, 1248, 1151, 774 cm^{-1}; HRMS (ESI): *m/z*: calcd. for $C_{57}H_{77}N_4O_6$ $[M+H]^+$: 913.58376, found: 913.58385.

Compound 36. A flame-dried two-necked round bottom flask was charged with flame-dried powdered 5 Å molecular sieves (2 g) under Ar. Toluene (64 mL) was added and the resulting suspension was degassed by bubbling a gentle stream of Ar through it for 30 min. The suspension was then heated to 60 °C (oil bath) for 30 min before tetra-yne **35** (117 mg, 0.13 mmol) was added at this temperature as a solution in toluene (2.0 mL). Next, a solution of catalyst **40a** (19 mg, 0.026 mmol, 20 mol%) in toluene (1.0 mL) was added dropwise over the course of 10 min. The resulting yellow suspension was stirred for 1 h at 60 °C before it was allowed to come to room temperature. The suspension was filtered through a pad of silica, which was carefully washed with EtOAc (3 x 30 mL). The combined filtrates were concentrated under reduced pressure and the residue purified by flash chromatography on silica gel (pentane/*tert*-butyl methyl ether, 8:2 to 4:6) to afford the title compound as a white film (94 mg, 91%). $[\alpha]_D^{20}$ = 20.1 (c = 0.45, CH_2Cl_2); ^1H NMR (400 MHz, CD_2Cl_2): δ = 7.98 (d, *J* = 8.5 Hz, 1H), 7.47 (t, *J* = 8.0 Hz, 1H), 7.39 (dd, *J* = 7.5, 1.3 Hz, 1H), 6.06 (dd, *J* = 6.8, 2.2 Hz, 1H), 5.14 (s, 1H), 4.17 (ddd, *J* = 13.6, 7.5, 5.8 Hz, 1H), 3.73 (m, 1H), 3.54 – 3.32 (m, 5H), 3.17 (td, *J* = 12.5, 1.9 Hz, 1H), 3.08 (dt, *J* = 12.7, 3.6 Hz, 1H), 3.03 – 2.89 (m, 2H), 2.82 – 2.67 (m, 2H), 2.63 – 2.47 (m, 4H), 2.40 – 2.26 (m, 4H), 2.23 – 2.01 (m, 4H), 1.95 (ddd, *J* = 14.4, 10.0, 5.0 Hz, 1H), 1.91 – 1.81 (m, 1H), 1.72 (dd, *J* = 9.5, 2.6 Hz, 1H), 1.66 – 1.54 (m, 13H), 1.53 – 1.44 (m, 4H), 1.43 (s, 9H), 1.38 – 1.23 (m, 5H) ppm; ^{13}C NMR (101 MHz, CD_2Cl_2): δ = 172.3, 164.6, 156.3, 152.2, 148.0, 147.1, 143.2, 139.2, 127.5, 125.8, 122.3, 121.8, 120.7, 119.1, 103.5, 83.4, 80.9, 80.1, 79.2, 76.3, 61.0, 55.6, 54.4, 52.6, 47.9, 46.3, 43.6, 41.0, 39.1, 37.4, 37.3, 34.3, 31.5, 30.7, 29.2, 28.6 (3C), 28.01, 27.97, 27.78 (3C), 27.75, 25.5, 25.4, 18.1, 15.1, 13.9 ppm; IR (film) $\tilde{\nu}$ = 3338, 2926, 2853, 1760, 1705, 1630, 1490, 1367, 1275, 1247, 1150, 753 cm^{-1}; HRMS (ESI): *m/z*: calcd. for $C_{49}H_{65}N_4O_6$ $[M+H]^+$: 805.48986, found: 805.48971.

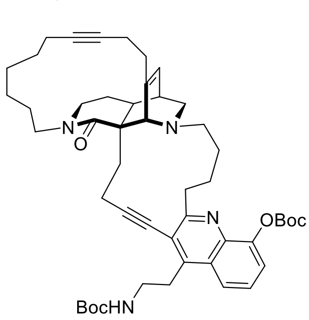

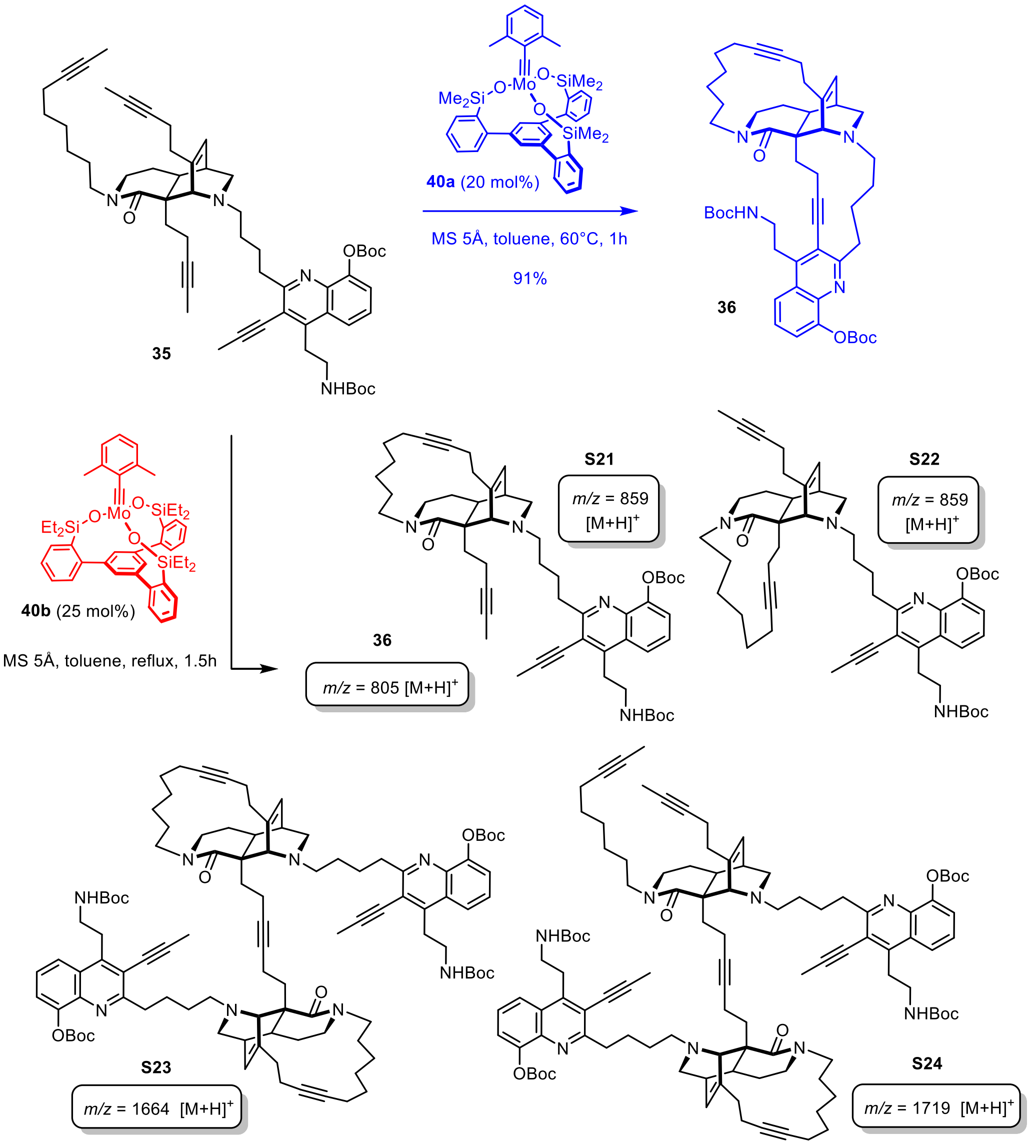

Scheme S1. Comparison of the performance of two homologous "canopy" catalysts differing only in the size of the lateral substituents on the silicon linkers.

Whereas complex **40a** furnished diyne **36** as the only product of the dRCAM reaction, the less active catalyst **40b** led to a rather complex mixture. Product **36** could be unambiguously identified as a minor component of this mixture (≈ 10%, MS); the other structure assignments shown in Scheme S1 are tentative as the compounds could not be separated and therefore no pure samples were available for unambiguous structure elucidation. However, the following observations and conclusions are deemed relevant:

- Two different compounds of the same mass (*m/z* = 859) make up for ≈ 60% (MS) of the crude mixture; this mass corresponds to products in which only one macrocycle has been closed. As the ^{1}H NMR spectrum shows strong signals for the methyl group of the propynyl substituent on the hydroxyquinoline ring, which has a characteristic shift (δ_H 2.23 ppm (s)) that is distinct from the shift of the methyl caps on the other alkynes (δ_H 1.75, 1.72, 1.66 ppm (t each)), we conclude that this moiety is untouched in the main components of the crude material. This conclusion is well in line with numerous earlier reactivity

data, which show that methyl-capped alkyl-alkynes are usually (much) more reactive than propynylated heteroaryl-alkynes in alkyne metathesis reactions of all sorts.[2,10,11]

- Under the premise that the propynylated quinoline moiety is intact, the two compounds of identical mass (*m/z* = 859) can only be **S21** and **S22**. One must hence conclude that the alkyne chains branching off the tricyclic core of substrate **35** can be connected in different ways and that RCAM is non-selective in the first place.
- If different ways of concatenation are possible on geometric grounds and initially occur with similar ease, then the selective formation of product **36** with the aid of the more active catalyst **40a** must involve "error correction", in that the initial product mixture is scrambled until the most favorable product has accumulated.
- This notion was supported by a control experiment, in which **40a** (30 mol%) was added to the mixture initially formed by **40b** (30 mol%). When the resulting solution was stirred at 110°C for 60 min, the initially complex product distribution converged to the desired product **36**, which could be isolated in pure form by HPLC in 58% yield. The lower yield is mainly caused by difficulties in the separation of the large amount of ligands present in the crude material as a result of the high combined catalyst loading of 60%.
- The notion that the initial RCAM events are unselective is further supported by the detection of dimeric species in the reaction mixture. One of them (**S23**, *m/z* = 1664, ≈ 8%, MS) is derived from the monomers with one macrocycle closed (*m/z* = 859), the other one (**S24**, *m/z* =1719, ≈ 4%, MS) seems to be formed by cross alkyne metathesis of **35** with one of these monocylic monomers **S21** or **S22**. As no dimers are present in the crude reaction mixture obtained with **40a**, this more active catalyst is likely capable of reconverting them into cyclic monomers. Related "depolymerization" reactions have previously been implied in the formation of macrocyclic rings.[12]

Completion of the Total Synthesis of Njaoamine C

Compound 37. Lindlar catalyst (5 mol% Pd on $CaCO_3$, poisoned with Pb; 9.3 mg, 4.3 µmol) was added to a solution of diyne **36** (7 mg, 8.7 µmol) in EtOAc (2 mL). The resulting suspension was purged with H_2 gas for 1 min and then vigorously stirred for 2 h under H_2 (1 atm) at room temperature. The suspension was filtered through a plug of Celite, which was carefully rinsed with EtOAc. The solvent was removed under reduced pressure and the residue was purified by flash chromatography on silica gel (pentane/*tert*-butyl methyl ether, 8:2 to 6:4) to afford the title compound as a white film (4.6 mg, 65%).

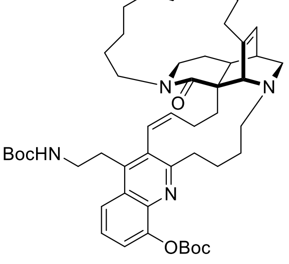

$[\alpha]_D^{20}$ = –37.1 (c = 0.34, CH_2Cl_2); ^1H NMR (600 MHz, CD_2Cl_2): δ = 8.03 (dd, *J* = 8.5, 1.2 Hz, 1H), 7.50 (t, *J* = 8.0 Hz, 1H), 7.40 (dd, *J* = 7.5, 1.2 Hz, 1H), 6.41 (d, *J* = 11.0 Hz, 1H), 5.84 (t, *J* = 10.0 Hz, 1H), 5.78 (dd, *J* = 6.7, 2.0 Hz, 1H), 4.73 (s, 1H), 4.11 (t, *J* = 12.1 Hz, 1H), 3.38 – 3.31 (m, 1H), 3.30 – 3.17 (m, 4H), 3.16 – 3.07 (m, 2H), 3.04 (dt, *J* = 12.4, 3.6 Hz, 1H), 2.99 (d, *J* = 8.1 Hz, 1H), 2.86 (td, *J* = 13.3, 4.0 Hz, 1H), 2.42 (dt, *J* = 13.7, 4.4

Hz, 1H), 2.37 – 2.24 (m, 2H), 2.22 – 2.14 (m, 3H), 1.95 (t, *J* = 12.1 Hz, 1H), 1.88 – 1.80 (m, 2H), 1.70 – 1.60 (m, 4H), 1.59 – 1.52 (m, 10H), 1.52 – 1.47 (m, 3H), 1.47 – 1.45 (m, 1H), 1.44 – 1.40 (m, 9H), 1.40 – 1.35 (m, 1H), 1.36 – 1.28 (m, 4H), 1.24 – 1.17 (m, 4H), 1.15 – 1.05 (m, 5H), 1.05 – 0.96 (m, 2H) ppm; ^{13}C NMR (151 MHz, CD_2Cl_2): δ = 172.3, 160.8, 156.1, 152.2, 148.1, 145.6, 143.3, 140.2, 136.1, 131.5, 128.0, 125.4, 124.4, 122.8, 121.6, 120.1, 83.1, 79.2, 57.4, 56.3, 55.9, 52.9, 47.4, 44.9, 43.0, 40.8, 39.0, 37.7, 37.4, 34.0, 31.3, 29.7, 28.6 (3C), 27.9 (3C), 27.7, 27.3, 27.2, 27.0, 26.8, 26.6, 26.0, 25.5, 25.4, 25.0, 23.6 ppm; IR (film) $\tilde{\nu}$ = 2977, 2928, 2857, 1762, 1710, 1640, 1488, 1367, 1272, 1245, 1151 cm^{-1}; HRMS (ESI): *m/z*: calcd. for $C_{49}H_{71}N_4O_6$ [M+H]$^+$: 811.53681, found: 811.53683.

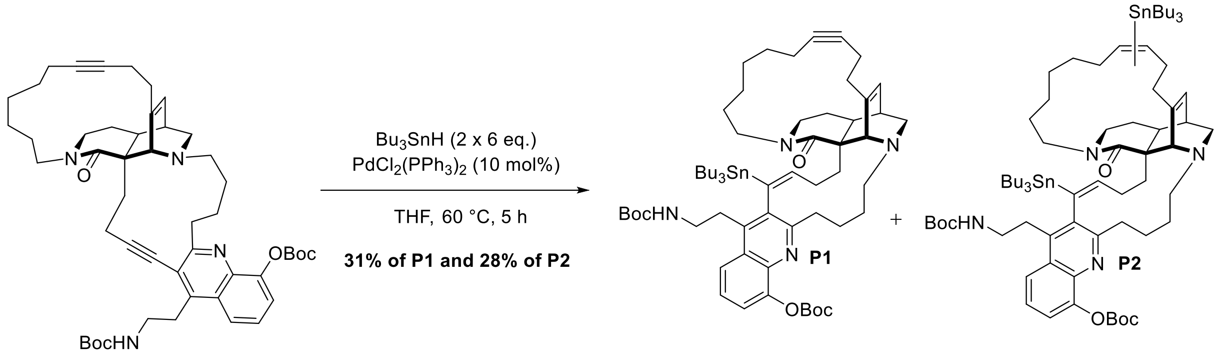

Scheme S2. Exploration of an alternative endgame based on double hydrostannylation. Whereas the palladium-catalyzed hydrostannylation of the triple bond attached to the hydroxyquinoline moiety proceeded smoothly, the dialkylalkyne unit reacted sluggishly despite the use of a very large excess of Bu_3SnH (12 eq., added in 2 portions), the high catalyst loading (10 mol% of Pd) and the rather forcing conditions (THF, reflux). For the poor product ratio and the difficulties in separating the compounds, this approach was not pursued any further.

Compound 38. Diyne **36** (81.6 mg, 0.1 mmol) was dissolved in EtOAc (5 mL). Quinoline (13 µL, 0.11 mmol) was added, followed by Lindlar catalyst (5 mol% Pd on $CaCO_3$, poisoned with Pb; 43 mg, 20 µmol). The suspension was cooled to 0 °C (ice bath) and purged with H_2 gas for 5 min before it was vigorously stirred under H_2 (1 atm) for 55 min at 0 °C. The suspension was then filtered through a plug of Celite, which was carefully rinsed with EtOAc. The solvent was removed under reduced pressure and the residue was purified by flash chromatography on silica gel (pentane/*tert*-butyl methyl ether, 9:1 to 4:6) to afford the title compound as a white film (73.6 mg, 90%). $[\alpha]_D^{20}$ = −68.6 (c = 0.7, CH_2Cl_2); ^{1}H NMR (400 MHz, CD_2Cl_2): δ = 7.98 (d, *J* = 8.5 Hz, 1H), 7.47 (t, *J* = 8.0 Hz, 1H), 7.39 (dd, *J* = 7.5, 1.3 Hz, 1H), 5.93 (dd, *J* = 6.6, 2.0 Hz, 1H), 5.51 (td, *J* = 10.3, 4.8 Hz, 1H), 5.43 (td, *J* = 9.8, 5.5 Hz, 1H), 5.09 (s, 1H), 4.08 (ddd, *J* = 13.5, 7.9, 5.7 Hz, 1H), 3.76 (s, 1H), 3.42 (m, 5H), 3.21 (ddd, *J* = 13.5, 11.4, 2.5 Hz, 1H), 3.15 – 3.09 (m, 1H), 3.05 (dt, *J* = 18.1, 4.0 Hz, 1H), 2.95 (dd, *J* = 9.5, 2.0 Hz, 1H), 2.82 – 2.70 (m, 2H), 2.53 (dt, *J* = 13.7, 4.9 Hz, 2H), 2.47 – 2.27 (m, 4H), 2.24 – 2.10 (m, 4H), 2.03 – 1.91 (m, 2H), 1.91 – 1.79 (m, 2H), 1.77 (dd, *J* = 9.5, 2.6 Hz, 1H), 1.56 (s, 13H), 1.53 – 1.46 (m, 4H), 1.42 (s, 9H), 1.36 – 1.26 (m, 5H) ppm; ^{13}C NMR (101 MHz, CD_2Cl_2): δ = 172.3, 164.7, 156.3, 152.2, 148.0, 147.0, 145.8, 139.2, 130.6, 129.7, 127.5, 125.8, 122.3, 121.8, 120.6, 119.1, 103.7, 83.4, 79.2, 76.2, 62.1, 55.7, 54.6, 52.3, 47.5, 47.4, 45.0, 41.0, 39.1, 37.7, 37.4, 35.9, 31.5, 30.8, 29.1, 28.6 (3C), 27.9,

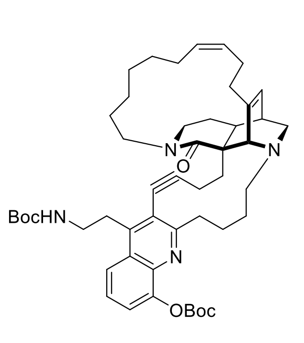

27.8 (3C), 27.4, 27.3, 25.7, 25.4, 25.3, 24.4, 14.3 ppm; IR (film) $\tilde{\nu}$ = 3336, 2927, 2854, 1761, 1708, 1630, 1489, 1367, 1273, 1246, 1149, 873, 735 cm^{-1}; HRMS (ESI): *m/z*: calcd. for $C_{49}H_{67}N_4O_6$ $[M+H]^+$: 807.50551, found: 807.50585.

Compound 39. In a flame-dried Schlenk flask, compound **38** (58 mg, 72 µmol) was dissolved in THF (1.5 mL).

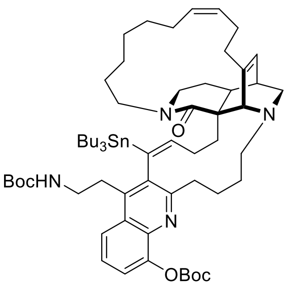

$Pd(PPh_3)_2Cl_2$ (2.5 mg, 3.6 µmol, 5 mol%) was added followed by a dropwise addition of Bu_3SnH (29 µL, 88 µmol) under Ar. The initially yellow solution immediately took a dark brown coloration. The Schlenk flask was closed and the mixture was stirred for 30 min. At this point, a second portion of Bu_3SnH (29 µL, 88 µmol) was added dropwise. After stirring for another 30 min, the solution was transferred to a round-bottom flask and the solvent was removed under reduced pressure. The residue was immediately loaded onto silica gel and the product eluded with pentane/*tert*-butyl methyl ether (95:5 to 7:3) to give the title compound as a white foam (58.3 mg, 73%). $[\alpha]_D^{20}$ = –56.1 (c = 1.0, CH_2Cl_2); ^1H NMR (600 MHz, CD_2Cl_2): δ = 8.06 (d, *J* = 8.5 Hz, 1H), 7.47 (t, *J* = 8.0 Hz, 1H), 7.34 (dd, *J* = 7.5, 1.2 Hz, 1H), 5.90 – 5.85 (m, 2H), 5.40 – 5.34 (m, 1H), 5.34 – 5.29 (m, 1H), 5.00 (br. s, 1H), 4.00 (ddd, *J* = 13.7, 8.1, 5.8 Hz, 1H), 3.37 – 3.28 (m, 2H), 3.24 – 3.12 (m, 3H), 3.08 – 2.99 (m, 4H), 2.87 (td, *J* = 12.8, 5.0 Hz, 1H), 2.42 – 2.32 (m, 2H), 2.26 – 2.12 (m, 4H), 2.06 (qd, *J* = 9.2, 4.7 Hz, 1H), 2.00 – 1.89 (m, 4H), 1.88 – 1.83 (m, 2H), 1.81 – 1.69 (m, 3H), 1.66 (td, *J* = 12.9, 6.5 Hz, 1H), 1.60 – 1.53 (m, 10H), 1.49 – 1.33 (m, 19H), 1.28 – 1.06 (m, 14H), 0.90 – 0.77 (m, 15H) ppm; ^{13}C NMR (151 MHz, CD_2Cl_2): δ = 172.1, 159.1, 156.4, 152.1, 148.0, 145.7, 145.2, 141.2, 139.29, 139.27, 137.3, 130.2, 129.8, 128.4, 125.1, 122.80, 122.76, 119.3, 83.0, 79.2, 57.3, 56.33, 56.30, 52.5, 47.3, 47.0, 45.0, 41.1, 39.4, 37.9, 37.8, 36.2, 31.1, 30.6, 29.3 (3C), 29.0, 28.6 (3C), 28.5, 27.9 (3C), 27.8 (3C), 27.4, 27.2, 26.9, 25.9, 25.6, 25.2, 24.5, 13.8 (3C), 11.3 (3C) ppm; ^{119}Sn NMR (149 MHz, CD_2Cl_2): δ = –42.2 ppm; IR (film) $\tilde{\nu}$ = 2927, 2856, 1763, 1715, 1619, 1487, 1457, 1367, 1270, 1247, 1152 cm^{-1}; HRMS (ESI): *m/z*: calcd. for $C_{61}H_{95}N_4O_6Sn$ $[M+H]^+$: 1099.62884, found: 1099.62681.

Compound S25. In a flame-dried Schlenk flask, a solution of compound **39** (37 mg, 33.7 µmol) in Et_2O (2 mL) was

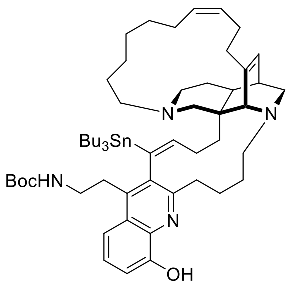

cooled to 0 °C (ice bath) under Ar. DIBAL-H (1.0 M solution in hexanes, 400 µL, 0.40 mmol) was added dropwise at this temperature. The Schlenk flask was closed and stirring was continued for 14 h at ambient temperature. For work up, the solution was cooled to 0 °C, *tert*-butyl methyl ether (1 mL) was added, followed by distilled water (0.5 mL) and a NaOH solution (2 M, 1.0 mL). The resulting biphasic mixture was vigorously stirred under air for 1 h before it was transferred to a separatory funnel. The aqueous phase was extracted with *tert*-butyl methyl ether (3 x 5 mL). The combined organic layers were washed with distilled water (10 mL) and brine (10 mL) and dried over anhydrous Na_2SO_4 before they were filtered and concentrated under reduced pressure. The residue was purified by flash chromatography on silica gel neutralized with triethylamine (pentane/*tert*-butyl methyl ether + 2% Et_3N, 98:2 to 8:2) to afford the title compound as a colorless film (24.7 mg, 74%). $[\alpha]_D^{20}$ = –116.0 (c = 1.0, MeOH); ^1H NMR (600 MHz, CD_2Cl_2): δ = 8.43 (br. s, 1H), 7.66 (d, *J* = 8.4 Hz, 1H), 7.40 (t, *J* = 8.1 Hz, 1H), 7.01 (dd, *J* = 7.5, 1.1

Hz, 1H), 5.95 (dd, J = 9.8, 1.9 Hz, $J_{Sn\text{-}H}$ = 30.4 Hz, 1H), 5.83 (d, J = 6.3 Hz, 1H), 5.36 (td, J = 10.6, 4.3 Hz, 1H), 5.30 – 5.22 (m, 1H), 4.75 (br. s, 1H), 3.38 – 3.17 (m, 3H), 3.11 – 2.97 (m, 3H), 2.87 (td, J = 12.9, 4.6 Hz, 1H), 2.64 – 2.53 (m, 1H), 2.50 – 2.42 (m, 2H), 2.37 (s, 1H), 2.35 – 2.19 (m, 4H), 2.17 – 2.10 (m, 2H), 2.08 – 1.92 (m, 5H), 1.93 – 1.82 (m, 2H), 1.77 (dd, J = 8.8, 2.5 Hz, 1H), 1.75 – 1.69 (m, 1H), 1.65 (dt, J = 13.6, 10.7 Hz, 1H), 1.61 – 1.58 (m, 1H), 1.57 – 1.55 (m, 1H), 1.55 – 1.34 (m, 17H), 1.32 – 1.17 (m, 15H), 1.15 – 1.05 (m, 3H), 0.88 (dd, J = 9.8, 7.3 Hz, 6H), 0.83 (t, J = 7.3 Hz, 9H); ^{13}C NMR (151 MHz, CD_2Cl_2): δ = 157.1, 156.2, 152.6, 146.1, 144.2, 140.6, 140.0, 138.1, 136.1, 130.4, 129.8, 127.4, 126.8, 121.3, 114.8, 108.0, 79.3, 58.9, 58.3, 57.5, 56.5, 53.3, 51.1, 44.9, 43.5, 40.8, 39.2, 39.1, 38.5, 38.2, 30.7, 30.1, 29.3 (3C), 28.6 (3C), 28.0, 27.8 (3C), 27.7, 27.0, 26.9, 26.7, 26.4, 26.2, 26.0, 24.8, 13.8 (3C), 11.4 (3C) ppm; the ^{119}Sn could not be detected in the 1D Spectra, it was assigned *via* ^{119}Sn-^{1}H HMBC (400 MHz, CD_2Cl_2): ^{119}Sn: δ = –44.5 ppm; IR (film) $\tilde{\nu}$ = 3367, 2925, 2854, 1709, 1492,1465, 1365, 1251, 1170, 960, 752, 614, 525 cm^{-1}; HRMS (ESI): *m/z*: calcd. for $C_{56}H_{89}N_4O_3Sn$ $[M+H]^+$: 985.59511, found: 985.59662.

***ent*-Njaoamine C Trifluoroacetate Salt ((–)-3·TFA)**. In a pressure Schlenk flask, alkenyl stannane **S25** (24.7 mg, 25.1 µmol) was dissolved in 1,2-dichloroethane (DCE, 2.5 mL). HCl (4 M in 1,4-dioxane, 630 µL, 2.5 mmol) was added, the Schlenk flask was closed and the resulting yellow solution was stirred at 60 °C (bath temperature) for 8 h, while a yellow suspension was formed. The solvent was removed by sparging the reaction with Ar, the remaining solid was dried under high vacuum for 30 min. The solid was then dissolved in MeOH and the solution passed through an amino cartridge (Bond Elut-NH_2, Agilent, 500 mg, 3 mL, 40 µm, pre-equilibrated with MeOH, distilled water and MeOH, 3 column-volumes each), using MeOH as the eluent. The solvent was removed under reduced pressure to afford the free amine, which was purified by preparative HPLC (150 mm YMC Triart C18 5 µm, 20.0 mm i.D., MeOH/0.1% TFA in H_2O = 40:60 isocratic over 20 min, then to MeOH/0.1% TFA in H_2O = 90:10 over 10 min, 15 mL/min, λ = 260 nm, t = 14.49 min). Evaporation of the product-containing fractions afforded the trifluoroacetate salt of Njaoamine C as a pale yellow powder (15.8 mg, 89%).

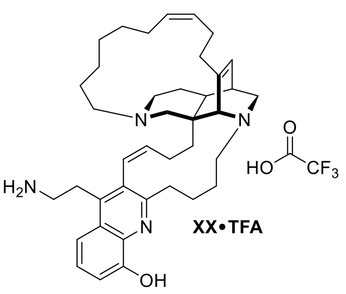

TFA salt of *ent*-Njaoamine C: $[\alpha]_D^{20}$ = –82.3 (c = 0.6, MeOH) [lit.:[13] $[\alpha]_D^{25}$ = +65.7 (c = 0.6, MeOH)]; **free base**: $[\alpha]_D^{20}$ = –102.3 (c = 0.6, MeOH); for the ^{1}H NMR, ^{13}C NMR and ^{19}F NMR data, see Table S1; IR (neat) $\tilde{\nu}$ = 3450, 2930, 1668, 1531, 1402, 1174, 1125 cm^{-1}; HRMS (ESI): *m/z*: calcd. for $C_{39}H_{55}N_4O$ $[M+H]^+$: 595.43704, found: 595.43731.

Table S1. **NMR Assignment and structure confirmation of the TFA salt of synthetic *ent*-Njaoamine C ((–)-3·TFA).** The complete assignment of synthetic **(–)-3·TFA** was carried out with a sample containing 3.3 mg of the material dissolved in 250 µL of [D5]-pyridine in a 3-mm tube. All measurements were performed on a Bruker Avance III 600 spectrometer equipped with a cryogenically cooled 5 mm TCI probehead using a classical set of 1D (^{1}H, ^{13}C) and 2D (^{13}C-HSQC, HMBC, COSY, TOCSY, ROESY and ^{15}N-HMBC) experiments. Numbering Scheme as shown in the Insert.

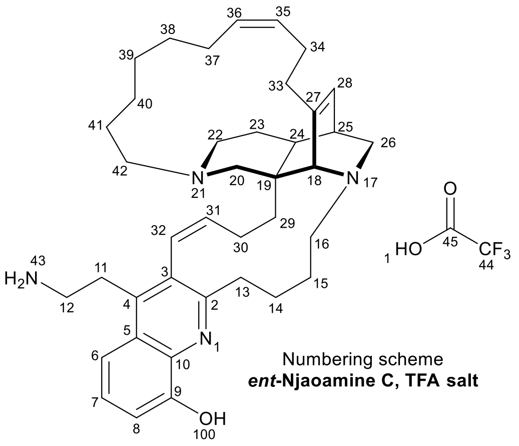

Atom	δ (ppm)	*J* (Hz)	COSY	HSQC	HMBC	NOESY
**1 N**	−91.86					
**2 C**	158.01				13a, 13b	
**3 C**	131.48				11a, 11b, 13b, 31, 32	
**4 C**	142.12				6, 11a, 11b, 32	
**5 C**	126.96				6, 7, 11a, 11b	
**6 C**	114.52			6	8	
**H**	7.6	8.40(7), 1.40(8)	7	6	4, 5, 8, 10	7, 11a, 11b, 12a, 12b
**7 C**	127.5			7		
**H**	7.3	8.40(6)	6, 8	7	5, 9	6
**8 C**	110.39			8	6	
**H**	7.26	1.40(6)	7	8	6, 9, 10	
**9 C***	153.97				7, 8	
**10 C**	137.6				6, 8	
**11 C**	28.35			11a, 11b	12a, 12b	
**Ha**	3.79	12.50, 5.40	11b, 12a, 12b	11	3, 4, 5, 12	6, 11b
**Hb**	3.57		11a	11	3, 4, 5, 12	6, 11a, 32
**12 C**	39.28			12a, 12b	11a, 11b	
**Ha**	3.58		11a, 12b	12	11	6
**Hb**	3.48	12.60, 5.90	11a, 12a	12	11	6
**13 C**	38.28			13a, 13b		
**Ha**	3.15	13.20, 4.40	13b	13	2, 14, 15	13b, 32
**Hb**	2.94	12.90, 4.90	13a, 14a, 14b	13	2, 3, 14, 15	13a
**14 C**	25.76			14a, 14b	13a, 13b, 15, 16a	
**Ha**	2.33		13b, 14b, 15	14		14b
**Hb**	1.5		13b, 14a, 15	14		14a
**15 C**	26.8			15	13a, 13b, 16b	
**H2**	1.31		14a, 14b, 16a, 16b	15	14, 16	18
**16 C**	56.24			16a, 16b	15, 18, 26b	
**Ha**	2.4		15, 16b	16	14, 26	16b, 18
**Hb**	1.97		15, 16a	16	15, 26	16a
**17 N**	−353.76					

**18 C**	56.85			18	24, 26a, 28	
**H**	2.65		28	18	16, 19, 20, 24, 25, 26, 27, 28, 29, 33	15, 16a, 20a, 29b, 33b
**19 C**	43.71				18, 20a, 20b, 23a, 24, 29a	
**20 C**	49.7			20a, 20b	18, 29a, 42a	
**Ha**	3.43	12.50	20b	20	19, 22, 24, 29	18, 20b
**Hb**	1.92		20a	20	19, 29, 42	20a
**21 N**						
**22 C**	49.5			22a, 22b	20a, 23b	
**Ha**	3.55		22b, 23b	22	24, 42	22b
**Hb**	2.99	13.10, 2.90	22a, 23a	22	42	22a
**23 C**	24			23a, 23b	24	
**Ha**	1.56		22b, 23b, 24	23	19	23b, 24, 25
**Hb**	1.08		22a, 23a	23	22, 24	23a
**24 C**	41.49			24	18, 20a, 22a, 23b, 25, 26a, 26b	
**H**	1.14		23a	24	18, 19, 23, 25, 28, 29	23a, 25, 26a
**25 C**	36.75			25	18, 24, 26a, 26b, 28, 33a	
**H**	2.08		26a, 26b, 28	25	24, 27, 28	23a, 24, 26a, 26b, 28
**26 C**	56.79			26a, 26b	16a, 16b, 18, 28	
**Ha**	3.03	9.30, 2.30	25, 26b	26	18, 24, 25, 28	24, 25, 26b
**Hb**	1.77		25, 26a	26	16, 24, 25, 28	25, 26a
**27 C**	143.71				18, 25, 33a, 34b	
**28 C**	122.42			28	18, 24, 25, 26a, 26b, 33a	
**H**	5.83	6.50	18, 25	28	18, 25, 26	25, 33a, 33b, 34a, 34b
**29 C**	36.56			29a, 29b	18, 20a, 20b, 24, 31	
**Ha**	2.22		30a, 30b	29	19, 20, 30, 31	30a, 30b
**Hb**	1.88		30b	29	30, 31	18, 29a
**30 C**	23.47			30a, 30b	29a, 29b, 31, 32	
**Ha**	2.74		29a, 30b	30	31	31
**Hb**	1.88		29a, 29b, 30a, 31	30	32	31
**31 C**	136.64			31	29a, 29b, 30a	
**H**	5.99	11.10(32)	30b, 32	31	3, 29, 30	30a, 30b, 32
**32 C**	124.5			32	30b	
**H**	6.41	11.10(31), 2.50	31	32	3, 4, 30	11b, 13a, 31
**33 C**	36.87			33a, 33b	18, 34b	
**Ha**	1.9		34a	33	25, 27, 28	28, 33b
**Hb**	1.2		34b	33		18, 28, 33a, 34b
**34 C**	24.4			34a, 34b	35, 36	
**Ha**	2.03		33a, 34b, 35	34	35, 36	28
**Hb**	1.9		33b, 34a	34	27, 33, 35, 36	28, 33b, 35, 36
**35 C**	129.62			35	34a, 34b, 37a, 37b	
**H**	5.34		34a	35	34, 37	34b, 37a, 37b

**36 C**	129.79			36	34a, 34b, 37a, 37b	
**H**	5.32		37b	36	34, 37	34b, 37a, 37b
**37 C**	26.65			37a, 37b	35, 36	
**Ha**	1.93		37b, 38a	37	35, 36	35, 36, 37b
**Hb**	1.53		36, 37a, 38b	37	35, 36, 38	35, 36, 37a
**38 C**	27.73			38a, 38b	37b, 39, 40b	
**Ha**	0.95		37a	38		38b
**Hb**	0.76		37b	38		38a
**39 C**	23.73			39	41a	
**H2**	1.2		40b	39	38, 40, 42	
**40 C**	25.35			40a, 40b	39, 41a	
**Ha**	1.13		40b	40	41	
**Hb**	1.06		39, 40a	40	38, 41	
**41 C**	19.69			41a, 41b	40a, 40b, 42a	
**Ha**	1.78		41b, 42a, 42b	41	39, 40, 42	41b
**Hb**	1.6	12.40, 3.50	41a, 42a, 42b	41		41a
**42 C**	57.49			42a, 42b	20b, 22a, 22b, 39, 41	
**Ha**	3.11	12.10, 5.80	41a, 41b, 42b	42	20, 41	42b
**Hb**	2.84		41a, 41b, 42a	42		42a
**43 N***						
**44 C**	118.31	298.1				
**45 C**	162.01	34.0				
**F3**	−74.21					

*C9-OH, δ_C = 153.97 ppm and $–NH_2$ 43 : the labile protons are not visible due to exchange with the NMR solvent.

Table S2. Comparison of the ^{13}C and ^{1}H chemical shifts of the isolated natural product Njaoamine C (as TFA salt)[13] and synthetic *ent*-Njaoamine C ((–)-3·TFA).

	Njaoamine C		Synthetic 3			
	δC	δH	δC	δH	|ΔδC|	|ΔδH|
**2 C**	158.1		158.01		0.09	
**3 C**	131.5		131.48		0.02	
**4 C**	142.2		142.12		0.08	
**5 C**	127		126.96		0.04	
**6 C**	114.6		114.52		0.08	
**H**		7.60		7.60		0.00
**7 C**	127.6		127.5		0.10	
**H**		7.30		7.30		0.00
**8 C**	110.5		110.39		0.11	
**H**		7.28		7.26		0.02
**9 C**	154		153.97		0.03	
**10 C**	137.6		137.6		0.00	
**11 C**	28.4		28.35		0.05	
**Ha**		3.82		3.79		0.03
**Hb**		3.61		3.57		0.04
**12 C**	39.3		39.28		0.02	
**Ha**		3.54		3.58		0.04
**Hb**		3.46		3.48		0.02
**13 C**	38.3		38.28		0.02	
**Ha**		3.14		3.15		0.01
**Hb**		2.98		2.94		0.04
**14 C**	25.8		25.76		0.04	
**Ha**		2.35		2.33		0.02
**Hb**		1.53		1.50		0.03
**15 C**	26.8		26.8		0.00	
**H2**		1.30		1.31		0.01
**16 C**	56.3		56.24		0.06	
**Ha**		2.42		2.40		0.02
**Hb**		1.98		1.97		0.01
**18 C**	56.8		56.85		0.05	
**H**		2.66		2.65		0.01
**19 C**	43.8		43.71		0.09	
**20 C**	49.7		49.7		0.00	
**Ha**		3.46		3.43		0.03
**Hb**		1.96		1.92		0.04
**22 C**	49.5		49.5		0.00	
**Ha**		3.59		3.55		0.04
**Hb**		3.05		2.99		0.06
**23 C**	24		24		0.00	
**Ha**		1.60		1.56		0.04
**Hb**		1.12		1.08		0.04
**24 C**	41.5		41.49		0.01	
**H**		1.16		1.14		0.02
**25 C**	36.8		36.75		0.05	
**H**		2.10		2.08		0.02
**26 C**	56.8		56.79		0.01	
**Ha**		3.06		3.03		0.03
**Hb**		1.78		1.77		0.01
**27 C**	143.8		143.71		0.09	
**28 C**	122.5		122.42		0.08	
**H**		5.82		5.83		0.01
**29 C**	36.6		36.56		0.04	
**Ha**		2.24		2.22		0.02
**Hb**		1.90		1.88		0.02
**30 C**	23.5		23.47		0.03	
**Ha**		2.76		2.74		0.02
**Hb**		1.93		1.88		0.05

**31 C**	136.7		136.64		0.06	
**H**		5.98		5.99		0.01
**32 C**	124.5		124.5		0.00	
**H**		6.43		6.41		0.02
**33 C**	36.9		36.87		0.03	
**Ha**		1.90		1.90		0.00
**Hb**		1.22		1.20		0.02
**34 C**	24.4		24.4		0.00	
**Ha**		2.04		2.03		0.01
**Hb**		1.92		1.90		0.02
**35 C[a]**	129.8		129.62		0.18	
**H**		5.34		5.34		0.00
**36 C[a]**	129.7		129.79		0.09	
**H**		5.34		5.32		0.02
**37 C**	26.7		26.65		0.05	
**Ha**		1.95		1.93		0.02
**Hb**		1.55		1.53		0.02
**38 C**	27.8		27.73		0.07	
**Ha**		0.94		0.95		0.01
**Hb**		0.76		0.76		0.00
**39 C[b]**	23.8		23.73		0.07	
**H2**		1.50; 1.20		1.20		0.00
**40 C**	25.4		25.35		0.05	
**Ha**		1.10		1.13		0.03
**Hb**		1.10		1.06		0.04
**41 C**	19.7		19.69		0.01	
**Ha**		1.80		1.78		0.02
**Hb**		1.60		1.60		0.00
**42 C**	57.5		57.49		0.01	
**Ha**		3.13		3.11		0.02
**Hb**		2.88		2.84		0.04

Comments

(1) A noticeable difference occurs only at C35 and C36 (olefin in the upper ring). These chemical shifts are close and the ^{1}H signals overlap. Our spectra do not allow us to differentiate them with certainty. For this reason, the assignment might be interchanged although it is not clear if this should be done in our or in the original assignment made by the isolation team.[13]

(2) Another aspect concerns the methylene group at position 39. Reyes *et al*. report two different chemical shifts (1.5 and 1.2 ppm) for the diastereotopic protons of the CH_2 group,[13] whereas we clearly see the signals of both protons (Ha and Hb) overlapping at 1.2 ppm. With lower resolution in the HSQC, it could be that Reyes *et al*. interpreted the cross-peak at 1.5 ppm as overlapping with that of C23-H23a (24 ppm and 1.6 ppm).

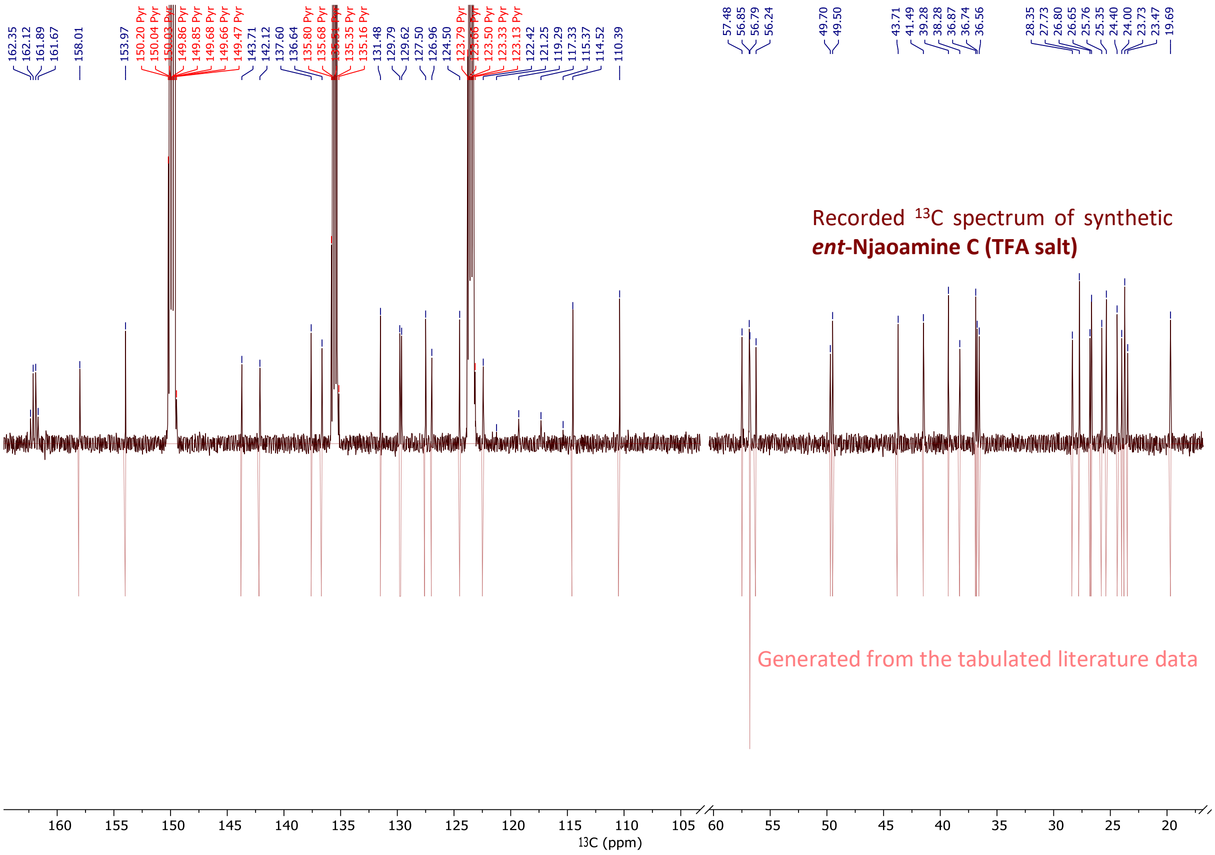

Figure S1. Visual comparison of the ^{13}C NMR data of authentic Njaoamine C (TFA salt, down) with synthetic *ent*-Njaoamine C ((–)-**3·TFA**, up); for the sake of comparison, the spectrum was referenced to [D_5]-pyridine as used in the isolation paper (δ_c = 123.50 ppm). Note that the shown spectrum (down) was genereated (MestReNova) by converting the tabulated ^{13}C NMR data of the natural product into a formal spectrum.[13] The intensity of the lines is arbitrarily set to be identical for all signals, for a tabular survey of the exact numbers, see Table S2.

The *cis*-configuration of the olefin C35-C36 was assigned, as in the isolation paper, based on the ^{13}C chemical shifts of the allylic C34 and C37 (24.4 and 26.7 ppm, respectively). This is in agreement with the observation that for the ircinals and the haliclamines series of compounds, *cis*-alkenes have allylic carbons at δ < 27 ppm whereas *trans*-alkenes have allylic carbons at δ > 30 ppm.[14] We have also simulated the line shape of the olefinic signals at 5.34 ppm (corresponding to protons H35-H36) using a six-1H-spin-system. Using the parameters shown in Table S3, an excellent fit was obtained for a coupling constant of $J_{H35-H36}$ = 11.03 Hz, which clearly indicates a *cis*-olefin (Figure S2).

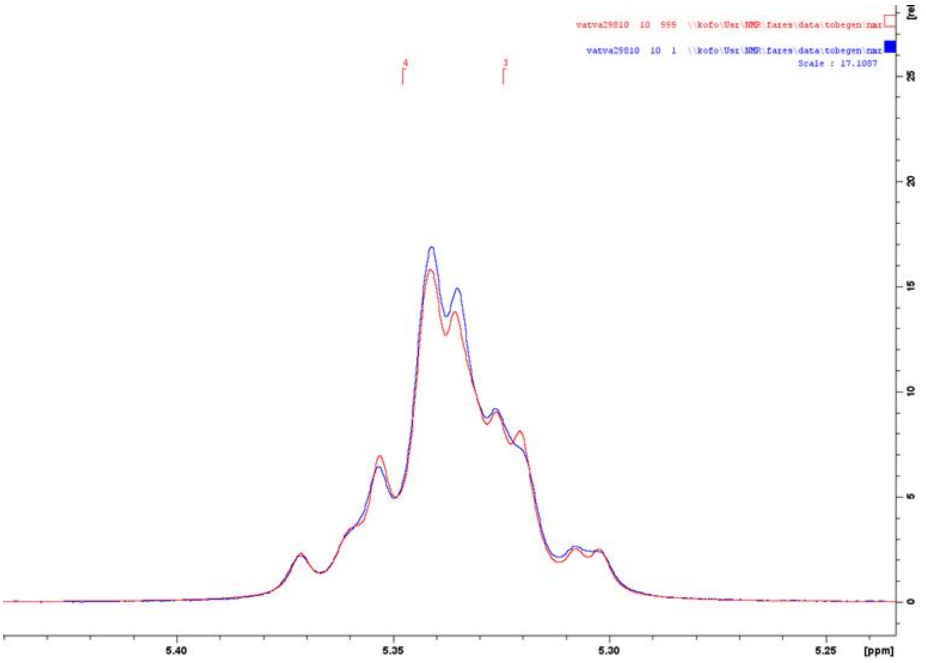

Figure S2. Simulation of the line shape for the olefinic signals at 5.34 ppm for a *cis*-olefin ($^{1}J_{H35\text{-}H36}$ = 11.03 Hz). The blue curve is the experimental signal while the red curve is the simulated signal.

When the coupling constant is forced to a "*trans*-olefin value" ($^{1}J_{H35\text{-}H36}$ = 15 Hz), the fit is less convincing (Figure S3).

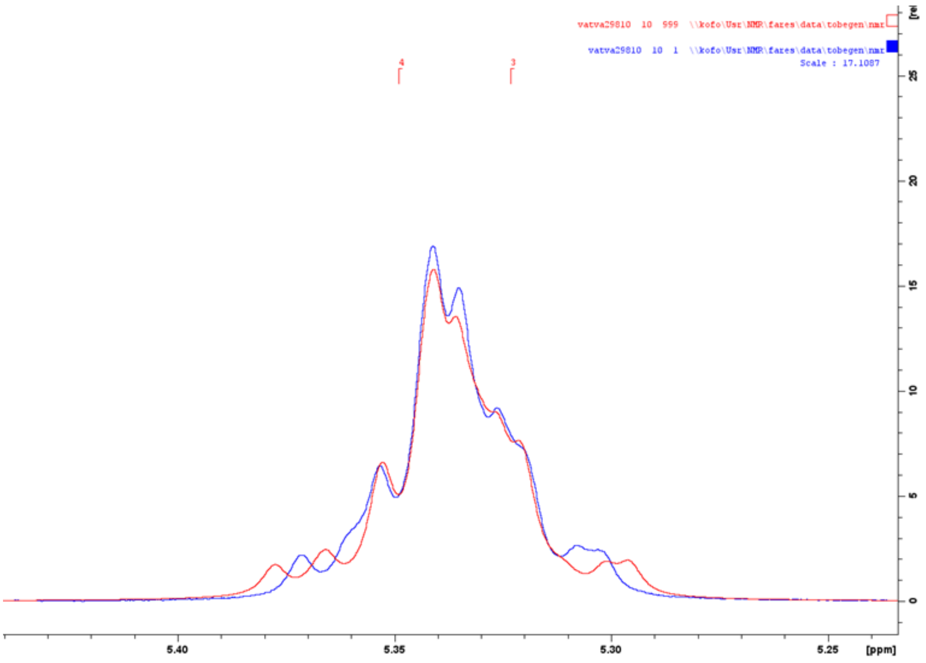

Figure S3. Simulation of the line shape for the olefinic signals at 5.34 ppm for a *trans*-olefin ($^{1}J_{H35\text{-}H36}$ = 15 Hz). The blue curve is the experimental signal while the red curve is the simulated signal.

Table S3. Start parameters defining the calculated spectrum. The simulation was carried out with the program DAISY with Topspin 3.6.

Nuc. ID.	ISO-Value	Shift (ppm)	Frequency (Hz)
1	1 H	2.0400	1224.4488
2	1 H	1.9200	1152.4224
3	1 H	5.3247	3195.9648
4	1 H	5.3477	3209.8227
5	1 H	1.9500	1170.4290
6	1 H	1.5000	900.3300

Couplings		Scalar	Dipolar
1	2	-14.3000	0.0000
1	3	3.6036	0.0000
1	4	0.0000	0.0000
1	5	0.0000	0.0000
1	6	0.0000	0.0000
2	3	9.8631	0.0000
2	4	0.0000	0.0000
2	5	0.0000	0.0000
2	6	0.0000	0.0000
3	4	11.0341	0.0000
3	5	0.0000	0.0000
3	6	0.0000	0.0000
4	5	7.9844	0.0000
4	6	6.5697	0.0000
5	6	-14.3000	0.0000

The dRCAM Route to Nominal Njaoamine I Revisited

Compound 41. ***Step 1:*** A flame-dried two-necked round bottom flask was charged with flame-dried powdered 5 Å molecular sieves (600 mg) under Ar. Toluene (20 mL) was added and the resulting suspension was degassed by bubbling Ar through it for 30 min and was then heated to 110 °C (oil bath) for 30 min. At this temperature, a solution of tetra-yne **7** (30 mg, 0.036 mmol) in toluene (1.0 mL) was added. Next, a solution of catalyst **40a** (6.7 mg, 0.009 mmol) in toluene (1.0 mL) was added dropwise over 10 min. Stirring of the resulting yellow suspension was continued for 1 h at 110 °C before the mixture was cooled to room temperature and filtered through a pad of silica, which was carefully rinsed with EtOAc (3 x 20 mL). The combined filtrates were concentrated under reduced pressure and the residue was purified by flash chromatography on silica gel (pentane/*tert*-butyl methyl ether, 8:2 to 4:6) to afford product **8** as a white film (15.4 mg, 59%). The data are consistent with the data reported in the literature.[5]

Step 2: In a flame-dried flask, diyne **8** (9 mg, 12.6 µmol) was dissolved in THF (500 µL). $Pd(PPh_3)_2Cl_2$ (10 mol %, 1.5 µmol, 1 mg) was added under Ar, followed by a dropwise addition of a solution of Bu_3SnH (5 µL, 18.8 µmol) in THF (100 µL). After stirring for 30 min at room temperature, a second portion of Bu_3SnH (5 µL, 18.8 µmol) in THF (100 µL) was added dropwise and stirring was continued for an additional 30 min at room temperature. The solution was then concentrated under reduced pressure and the residue was purified by flash chromatography on silica gel (pentane/*tert*-butyl methyl ether with 2% of Et_3N, 98:2 to 8:2) to give the title compound as a brown film (8.2 mg, 65%). $[\alpha]_D^{20}$ = −16.5 (c = 0.8, CH_2Cl_2); ^{1}H NMR (600 MHz, CD_2Cl_2): δ = 8.16 (d, *J* = 8.4 Hz, 1H), 7.92 (d, *J* = 8.3 Hz, 1H), 7.59 (ddd, *J* = 8.3, 6.8, 1.3 Hz, 1H), 7.51 (t, *J* = 7.6 Hz, 1H), 5.89 (dd, *J* = 10.5, 2.0 Hz, 1H), 5.79 (dd, *J* = 6.8, 1.9 Hz, 1H), 4.95 (s, 1H), 3.98 – 3.90 (m, 1H), 3.41 – 3.24 (m, 2H), 3.23 – 3.15 (m, 2H), 3.13 (d, *J* = 1.6 Hz, 1H), 3.08 – 2.98 (m, 4H), 2.86 (td, *J* = 12.9, 4.9 Hz, 1H), 2.44 – 2.37 (m, 2H), 2.29 – 2.23 (m, 1H), 2.23 – 2.18 (m, 1H), 2.17 (dq, *J* = 6.6, 2.2 Hz, 1H), 2.14 – 2.07 (m, 4H), 2.05 (dddd, *J* = 17.7, 8.8, 6.8, 2.1 Hz, 1H), 1.97 (td, *J* = 12.6, 3.4 Hz, 1H), 1.93 – 1.88 (m, 1H), 1.88 – 1.82 (m, 1H), 1.81 – 1.76 (m, 1H), 1.74 (dd, *J* = 8.7, 2.3 Hz, 1H), 1.72 – 1.67 (m, 1H), 1.66 – 1.61 (m, 1H), 1.56 – 1.52 (m, 2H), 1.51 – 1.37 (m, 21H), 1.37 – 1.28 (m, 5H), 1.24 – 1.20 (m, 8H), 1.18 – 1.09 (m, 2H), 0.89 – 0.82 (m, 6H), 0.80 (t, *J* = 7.3 Hz, 9H) ppm; ^{13}C NMR (151 MHz, CD_2Cl_2): δ = 172.0, 159.2, 156.3, 146.5, 145.3, 145.1, 141.5, 139.1, 136.6, 129.7, 127.8, 127.1, 125.9, 124.7, 122.1, 81.3, 79.8, 79.2, 57.4, 56.1, 55.3, 52.7, 47.5, 47.4, 44.1, 41.1, 39.5, 37.8, 37.7, 36.4, 31.2, 30.4, 29.4, 29.3 (3C), 28.6 (3C), 28.5, 28.2, 27.8 (3C), 27.7, 27.6, 27.4, 27.1, 26.2, 24.6, 19.1, 18.2, 13.8 (3C), 11.3 (3C) ppm; ^{119}Sn NMR (149 MHz, CD_2Cl_2): δ = −43.1 ppm; IR (film) $\tilde{\nu}$ = 2956, 2928, 2853, 1713, 1644, 1624, 1490, 1455, 1426, 1365, 1259, 1170, 1029 cm^{-1}; HRMS (ESI): *m/z*: calcd. for $C_{58}H_{88}N_4O_3SnNa$ $[M+Na]^+$: 1031.57706, found: 1031.57741.

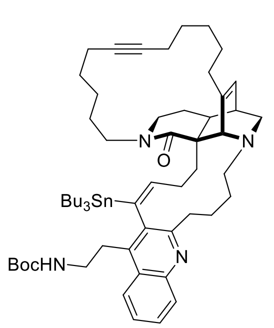

Compound 42·TFA. In a pressure Schlenk tube, alkenyl stannane **41** (8.2 mg, 8.1 µmol) was dissolved in 1,2-dichloroethane (DCE, 800 µL). HCl (4 M in 1,4-dioxane, 200 µL, 0.81 mmol) was added, the Schlenk flask was closed, and the resulting yellow solution was stirred at 60 °C for 8 h. The solvent was then removed by sparging the mixture with Ar. The residue was dried under high vacuum for 30 min before it was dissolved in MeOH and passed through an amino cartridge (Bond Elut-NH_2, Agilent, 500 mg, 3 mL, 40 µm, pre-equilibrated with MeOH, distilled water and MeOH, 3 column volumes each), eluting with MeOH. The combined product-containing fractions were evaporated under reduced pressure to afford the free amine, which was purified by preparative HPLC (150 mm YMC Triart C18 5 µm, 20.0 mm i.D., MeOH/0.1% TFA in H_2O = 60:40 isocratic over 20 min, then to MeOH/0.1% TFA in H_2O = 90:10 over 10 min, 15 mL/min, λ = 260 nm, t = 4.3 min) to give the trifluoroacetate salt of **42** as an off-white powder (1.8 mg, 30%). $[\alpha]_D^{20}$ = +38.6 (c = 0.12, MeOH); ^{1}H NMR (600 MHz, [D$_5$]-pyridine): δ = 8.25 (d, *J* = 8.3 Hz, 1H), 8.17 (d, *J* = 8.4 Hz, 1H), 7.57 (d, *J* = 7.2 Hz, 1H) 7.37 (t, *J* = 7.6 Hz, 1H), 6.44 (dd, *J* = 10.8, 2.5 Hz, 1H), 5.84 (t, *J* = 11.2 Hz, 1H), 5.76 (dd, *J* = 6.5, 2.0 Hz, 1H), 4.10 – 4.00 (m, 1H), 3.87 – 3.80 (m, 1H), 3.76 (td, *J* = 12.7, 5.2 Hz, 1H), 3.69 – 3.57 (m, 2H), 3.32 (s, 1H), 3.26 (dt, *J* = 13.3, 4.1 Hz, 1H), 3.15 – 3.07 (m, 2H), 3.04 (d, *J* = 9.0 Hz, 1H), 2.94 (dt, *J* = 12.5, 3.7 Hz, 1H), 2.49 – 2.34 (m, 3H), 2.25 – 2.11 (m, 6H), 2.10 – 2.04 (m, 2H), 2.04 – 1.97 (m,

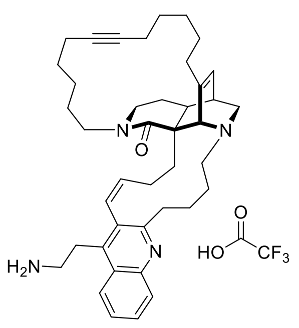

1H), 1.89 (ddd, J = 21.7, 11.2, 5.6 Hz, 1H), 1.73 (d, J = 7.9 Hz, 1H), 1.70 – 1.65 (m, 1H), 1.63 – 1.46 (m, 6H), 1.45 – 1.20 (m, 11H), 1.17 – 1.11 (m, 1H), 1.02 (qd, J = 12.4, 3.9 Hz, 1H) ppm; ^{13}C NMR (151 MHz, [D$_5$]-pyridine): δ = 171.7, 162.1 (q, $^2J_{C-F}$ = 33.5 Hz, from TFA), 160.7, 147.4, 145.1, 141.1, 136.8, 130.8, 130.2, 128.9, 126.5, 126.1, 124.2, 124.1, 122.0, 81.3, 80.0, 57.1, 56.1, 55.6, 52.3, 47.13, 47.09, 43.6, 39.3, 38.7, 37.5, 36.9, 36.2, 31.0, 29.1, 28.0, 27.7, 27.6, 27.3, 27.2, 27.0, 25.6 (2C), 24.5, 19.1, 18.1 ppm; IR (film) $\tilde{\nu}$ = 3413, 2930, 2858, 1674, 1492, 1429, 1372, 1200, 1131, 834, 800, 721 cm^{-1}; HRMS (ESI): *m/z*: calcd. for $C_{41}H_{55}N_4O_1$ [M+H]$^+$: 619.43704, found: 619.43754.

References

1. Hickmann, V.; Alcarazo, M.; Fürstner, A., Protecting-Group-Free and Catalysis-Based Total Synthesis of the Ecklonialactones. *J. Am. Chem. Soc.* **2010,** *132* (32), 11042-11044.
2. Hillenbrand, J.; Leutzsch, M.; Yiannakas, E.; Gordon, C. P.; Wille, C.; Nöthling, N.; Copéret, C.; Fürstner, A., "Canopy Catalysts" for Alkyne Metathesis: Molybdenum Alkylidyne Complexes with a Tripodal Ligand Framework. *J. Am. Chem. Soc.* **2020,** *142* (25), 11279-11294.
3. Hillenbrand, J.; Korber, J. N.; Leutzsch, M.; Nöthling, N.; Fürstner, A., Canopy Catalysts for Alkyne Metathesis: Investigations into a Bimolecular Decomposition Pathway and the Stability of the Podand Cap. *Chem. Eur. J.* **2021,** *27* (56), 14025-14033.
4. Meng, Z.; Fürstner, A., Total Synthesis Provides Strong Evidence: Xestocyclamine A is the Enantiomer of Ingenamine. *J. Am. Chem. Soc.* **2020,** *142* (27), 11703-11708.
5. Meng, Z.; Spohr, S. M.; Tobegen, S.; Farès, C.; Fürstner, A., A Unified Approach to Polycyclic Alkaloids of the Ingenamine Estate: Total Syntheses of Keramaphidin B, Ingenamine, and Nominal Njaoamine I. *J. Am. Chem. Soc.* **2021,** *143* (35), 14402-14414.
6. Konishi, S.; Kawamorita, S.; Iwai, T.; Steel, P. G.; Marder, T. B.; Sawamura, M., Site-Selective C-H Borylation of Quinolines at the C8 Position Catalyzed by a Silica-Supported Phosphane–Iridium System. *Chem. Asian J.* **2014,** *9* (2), 434-438.
7. Ueda, H.; Satoh, H.; Matsumoto, K.; Sugimoto, K.; Fukuyama, T.; Tokuyama, H., Total Synthesis of (+)-Haplophytine. *Angew. Chem. Int. Ed.* **2009,** *48* (41), 7600-7603.
8. Trost, B. M.; Papillon, J. P. N.; Nussbaumer, T., Ru-Catalyzed Alkene–Alkyne Coupling. Total Synthesis of Amphidinolide P. *J. Am. Chem. Soc.* **2005,** *127* (50), 17921-17937.
9. Flowers, C. L.; Vogel, P., Short Diastereoselective Synthesis of the C1–C13 (AB Spiroacetal) and C17–C28 Fragments (CD Spiroacetal) of Spongistatin 1 and 2 through Double Chain-Elongation Reactions. *Chem. Eur. J.* **2010,** *16* (47), 14074-14082.
10. Fürstner, A., The Ascent of Alkyne Metathesis to Strategy-Level Status. *J. Am. Chem. Soc.* **2021,** *143* (38), 15538-15555.
11. Fürstner, A., Alkyne Metathesis on the Rise. *Angew. Chem. Int. Ed.* **2013,** *52* (10), 2794-2819.
12. Lehr, K.; Fürstner, A., An efficient route to the musk odorant (R,Z)-5-muscenone via base-metal-catalysis. *Tetrahedron* **2012,** *68* (37), 7695-7700.
13. Reyes, F.; Fernández, R.; Urda, C.; Francesch, A.; Bueno, S.; de Eguilior, C.; Cuevas, C., Njaoamines A–F, new cytotoxic polycyclic alkaloids from the haplosclerid sponge Reniera sp. *Tetrahedron* **2007,** *63* (11), 2432-2438.
14. Kong, F.; Andersen, R. J.; Allen, T. M., Madangamine A, a novel cytotoxic alkaloid from the marine sponge Xestospongia ingens. *J. Am. Chem. Soc.* **1994,** *116* (13), 6007-6008.
