# Supplementary material for: Total Synthesis of Njaoamine C by Concurrent Macrocycle Formation
Source: J Am Chem Soc. 2023 Sep 21;145(39):21197–202. doi: 10.1021/jacs.3c08410 (PMC10557140; doi:10.1021/jacs.3c08410)

SUPPORTING INFORMATION

Total Synthesis of Njaoamine C by Concurrent Macrocycle Formation

Thomas Varlet, Sören Portmann, and Alois Fürstner*

Max-Planck-Institut für Kohlenforschung, 45470 Mülheim/Ruhr, Germany
E-Mail: fuerstner@kofo.mpg.de

Copies of NMR Spectra

Compound S1

^{1}H ($CDCl_3$, 400 MHz)

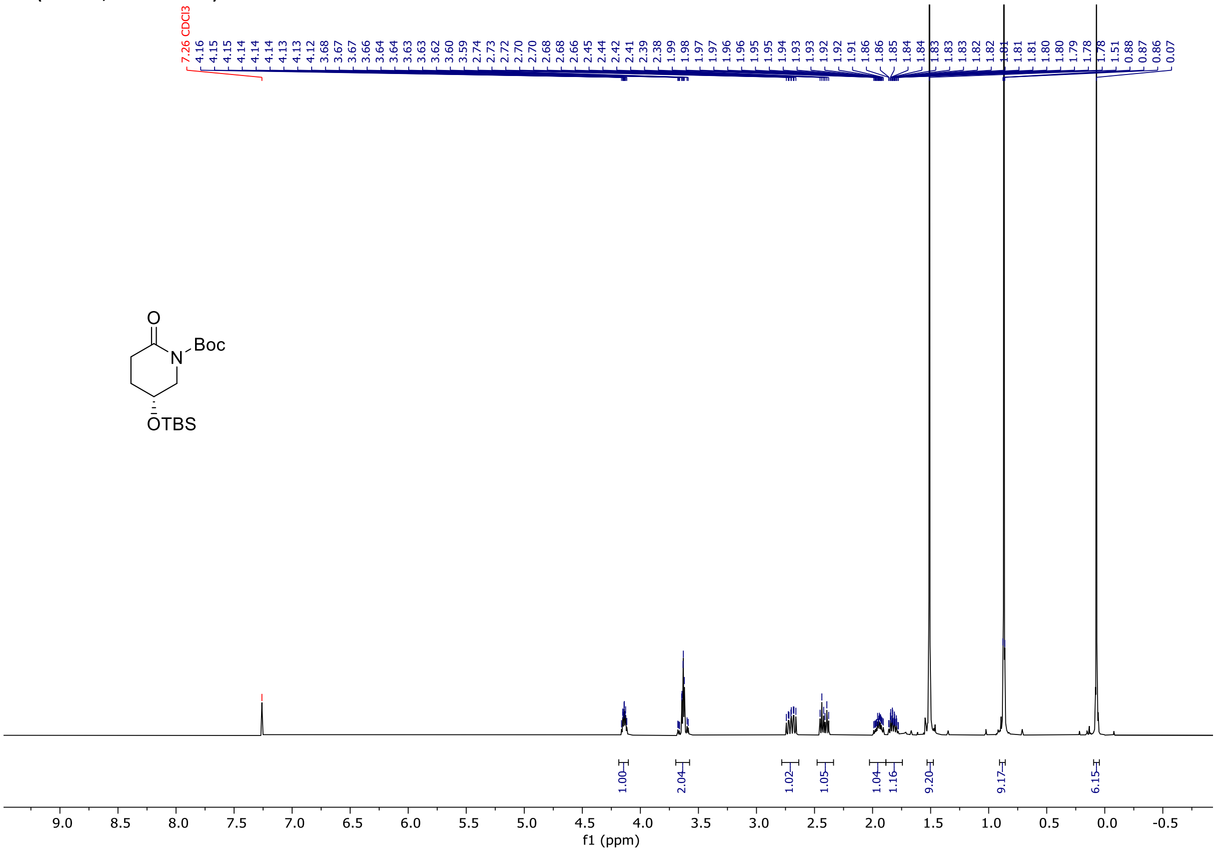

^{13}C ($CDCl_3$, 101 MHz)

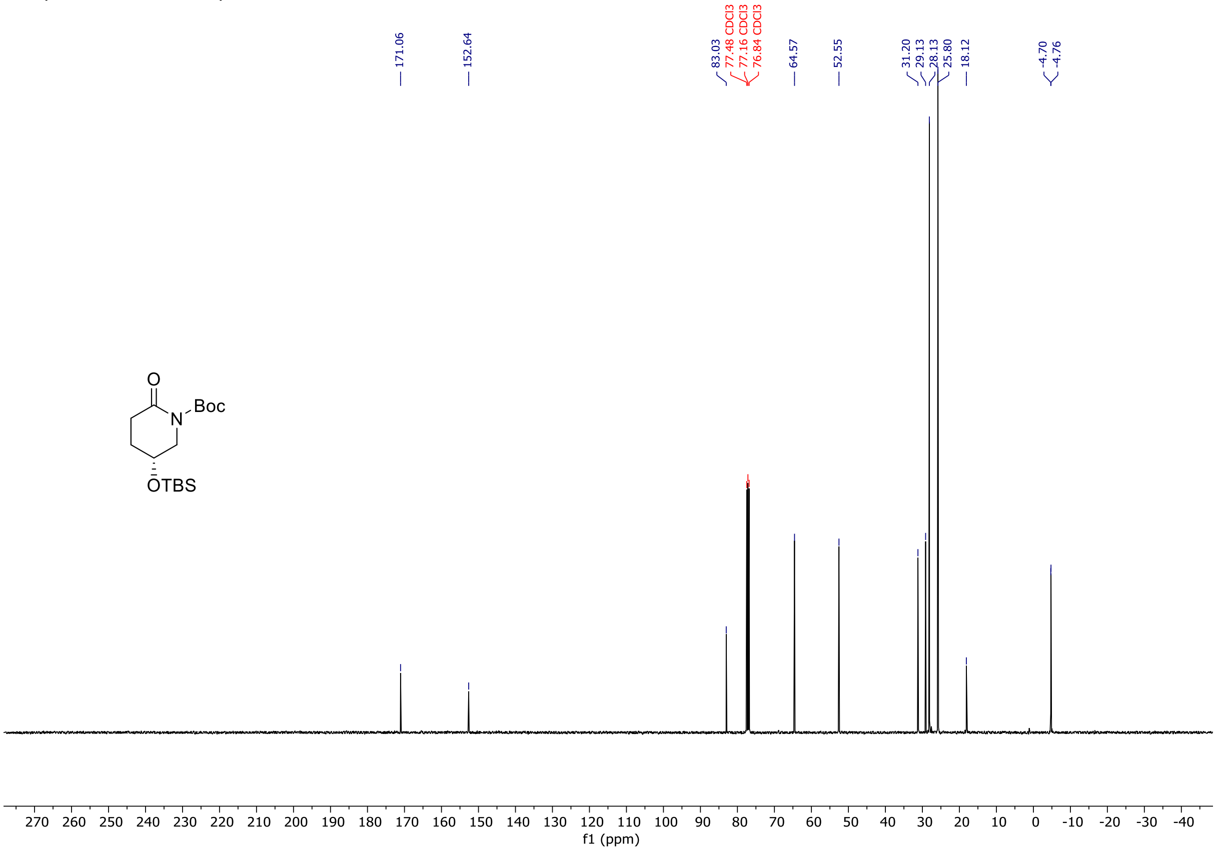

Compound S2

^{1}H ($CDCl_3$, 400 MHz)

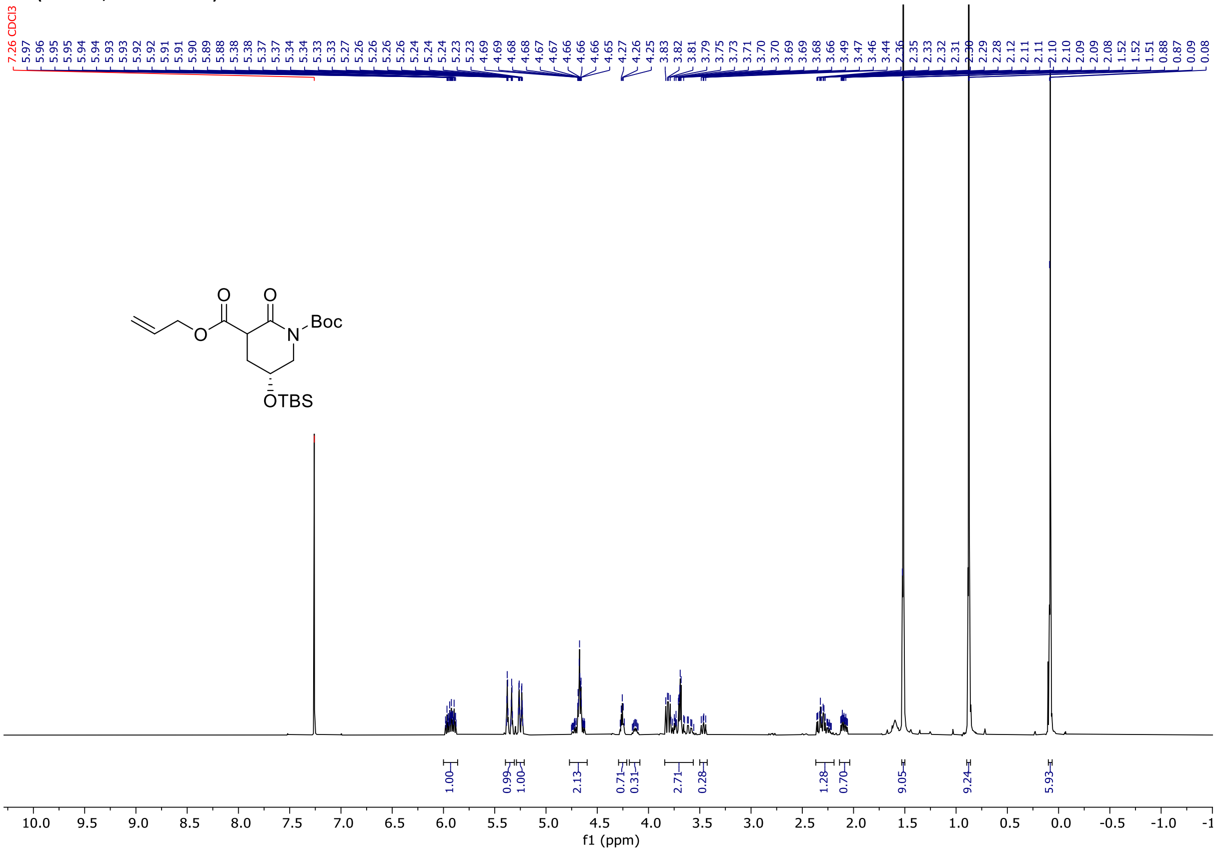

^{13}C ($CDCl_3$, 101 MHz)

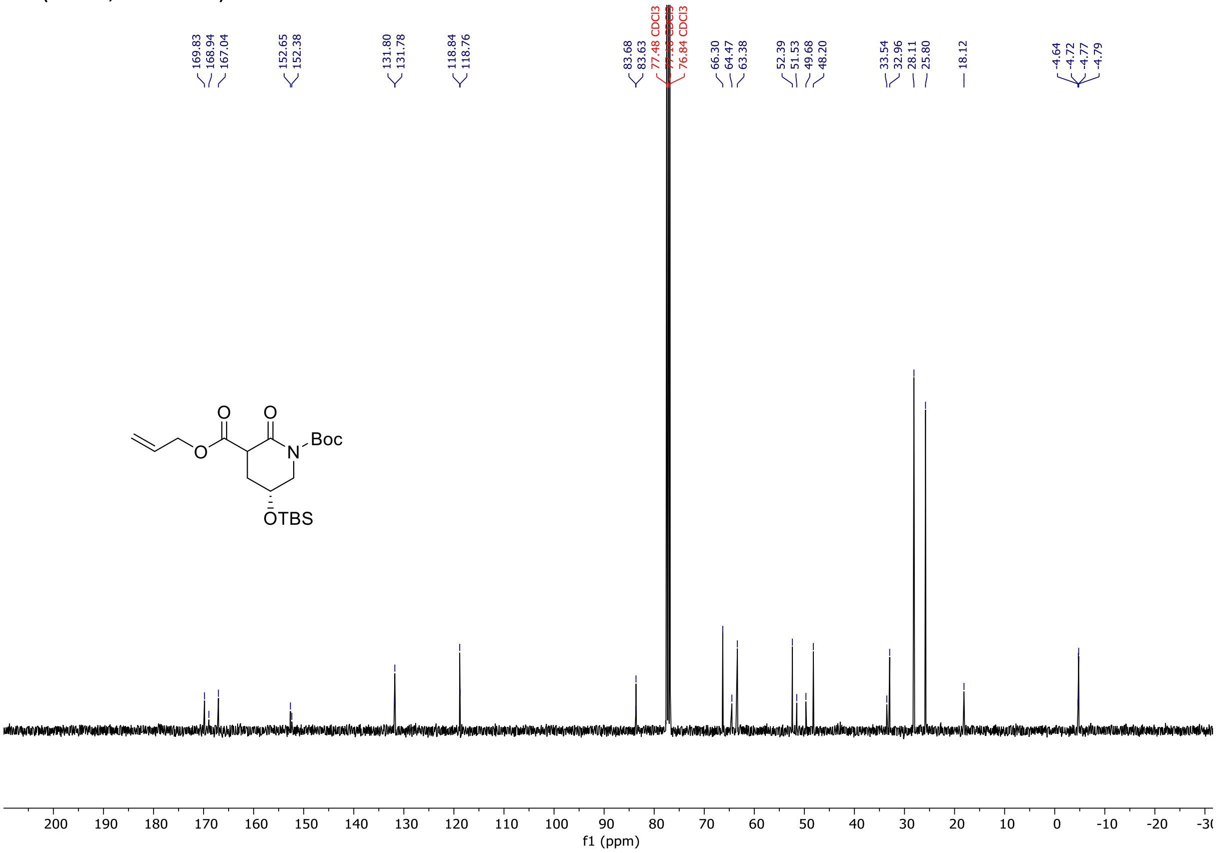

Compound S3

^{1}H ($CDCl_3$, 400 MHz)

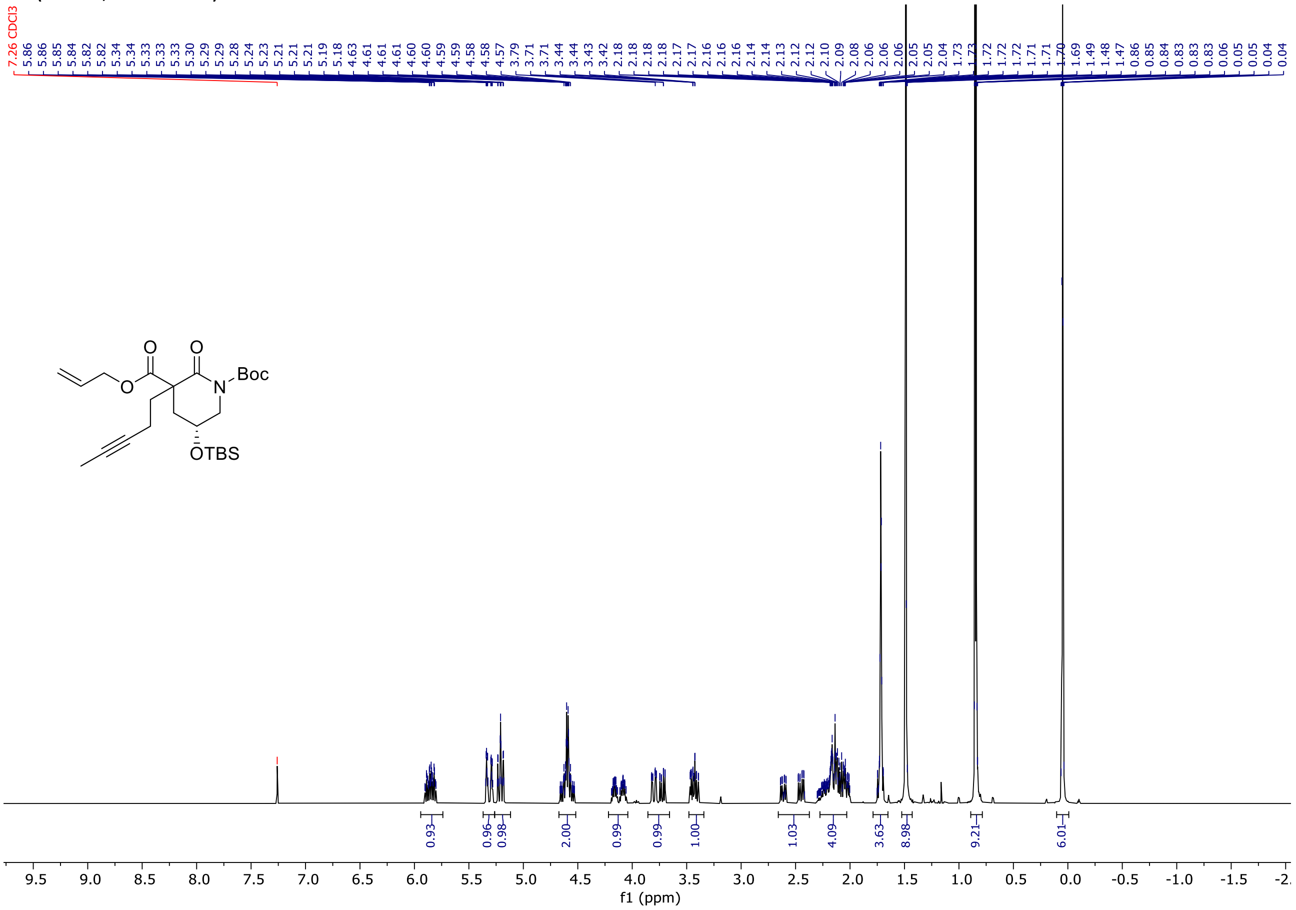

^{13}C ($CDCl_3$, 101 MHz)

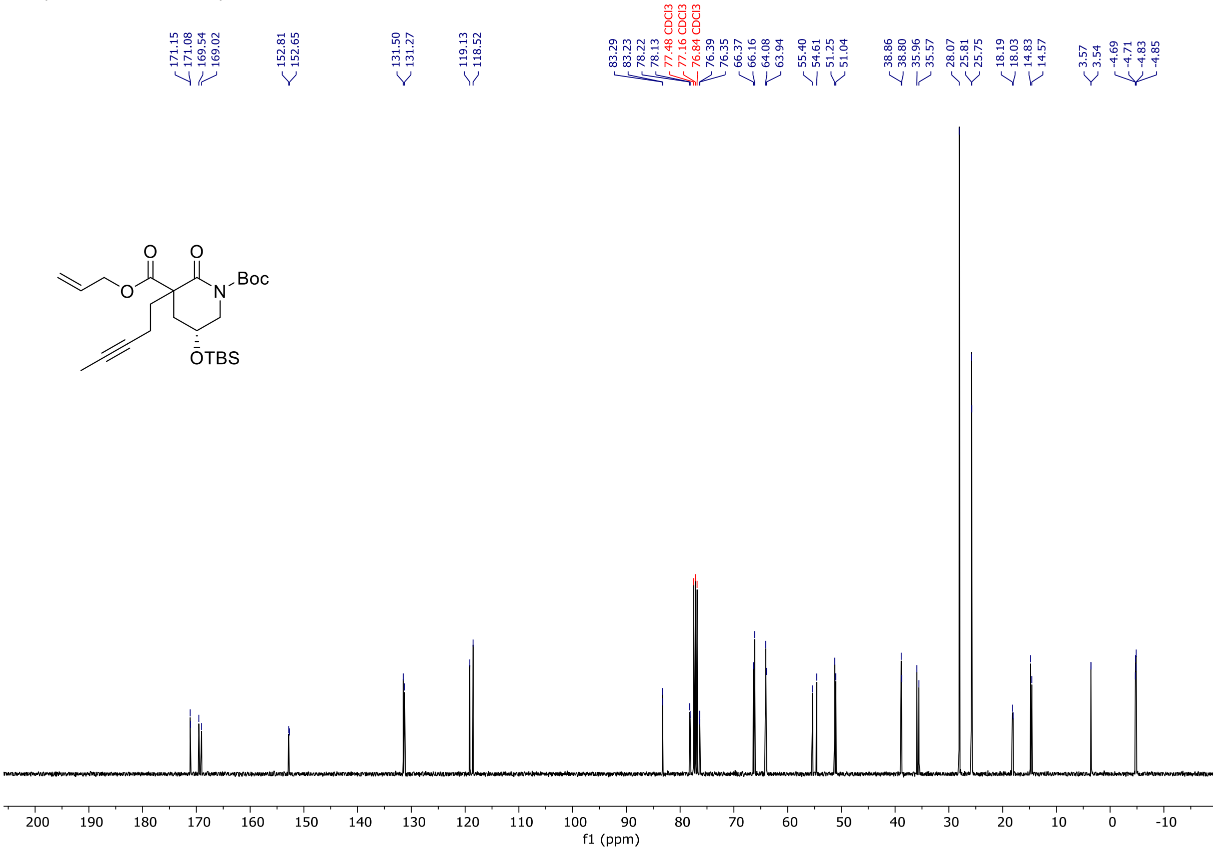

Compound 16

^{1}H ($CDCl_3$, 400 MHz)

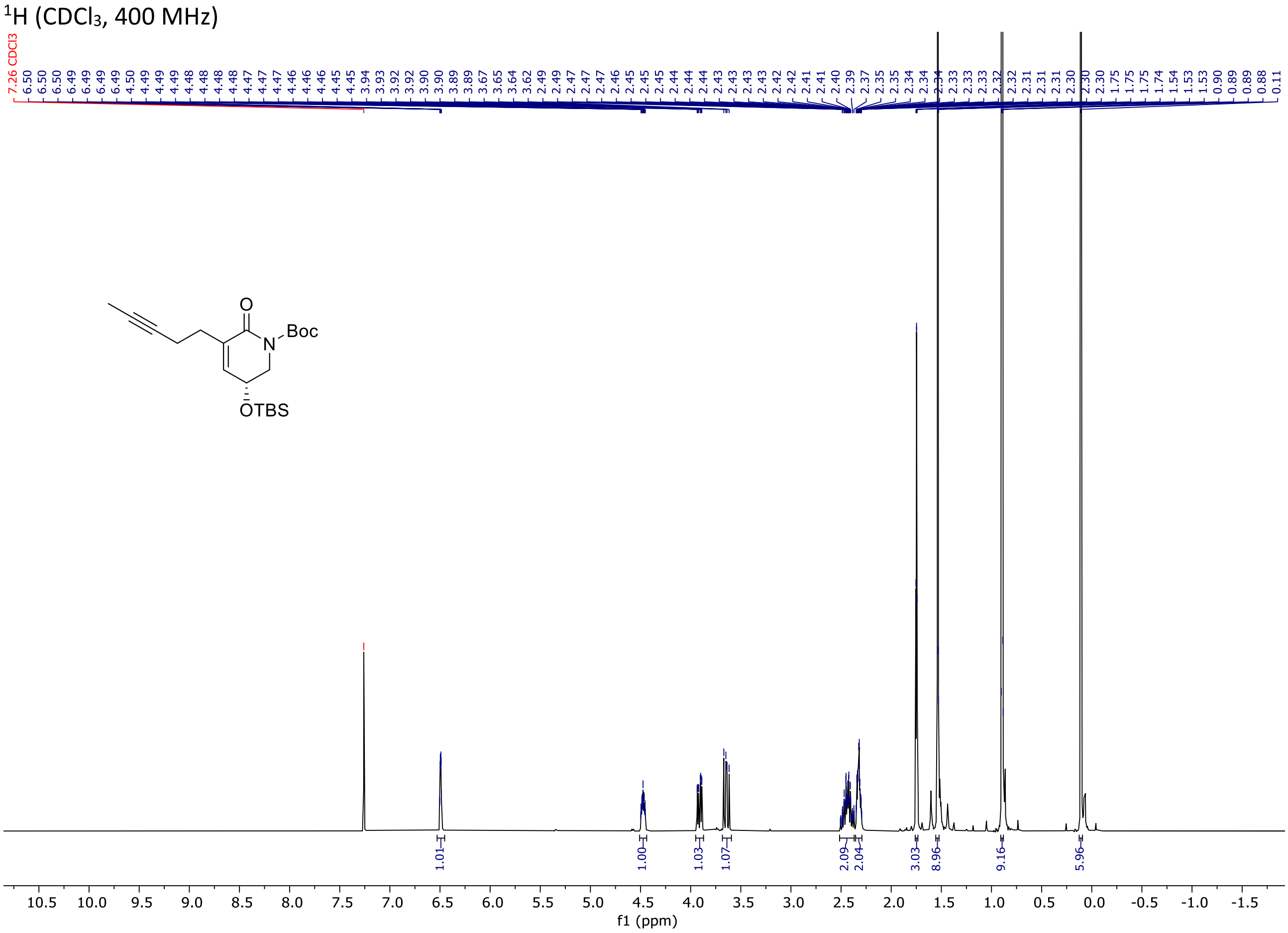

^{13}C ($CDCl_3$, 101 MHz)

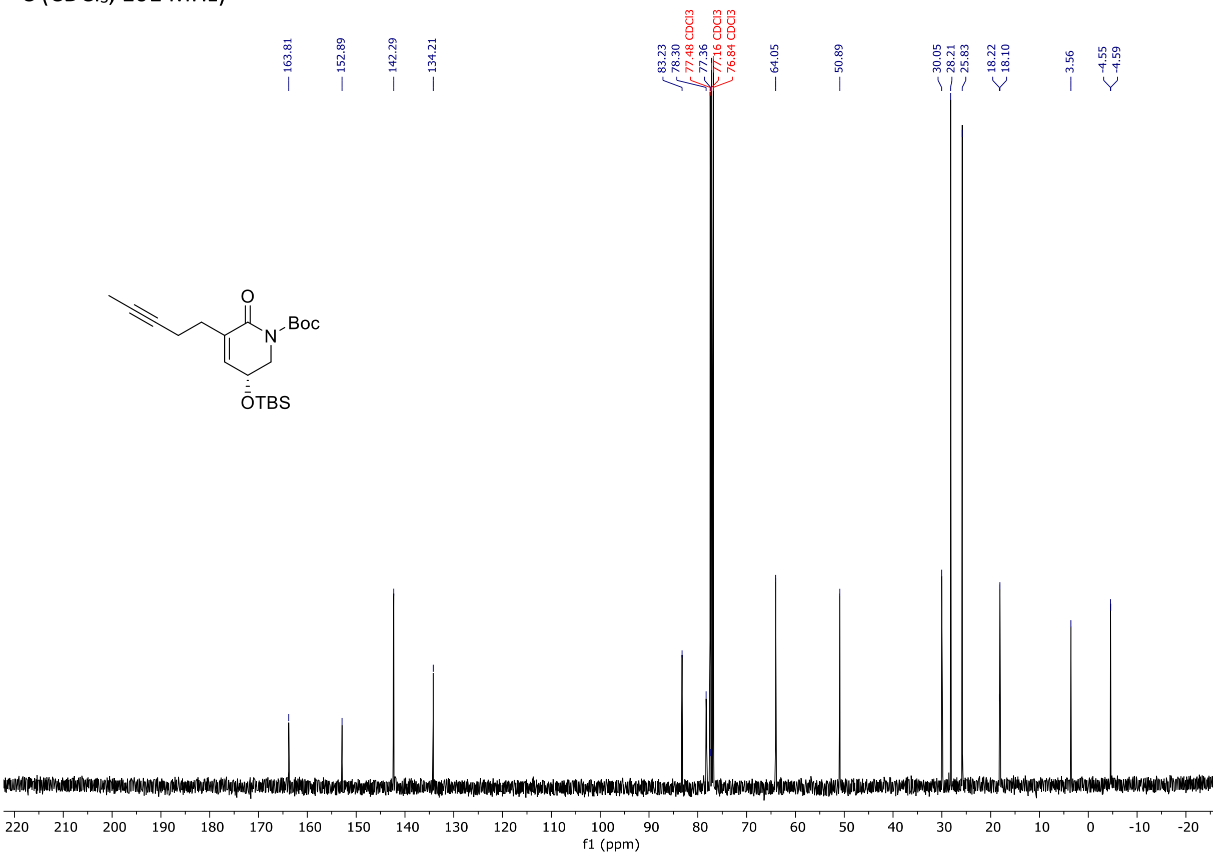

Compound S4

^{1}H ($CDCl_3$, 400 MHz)

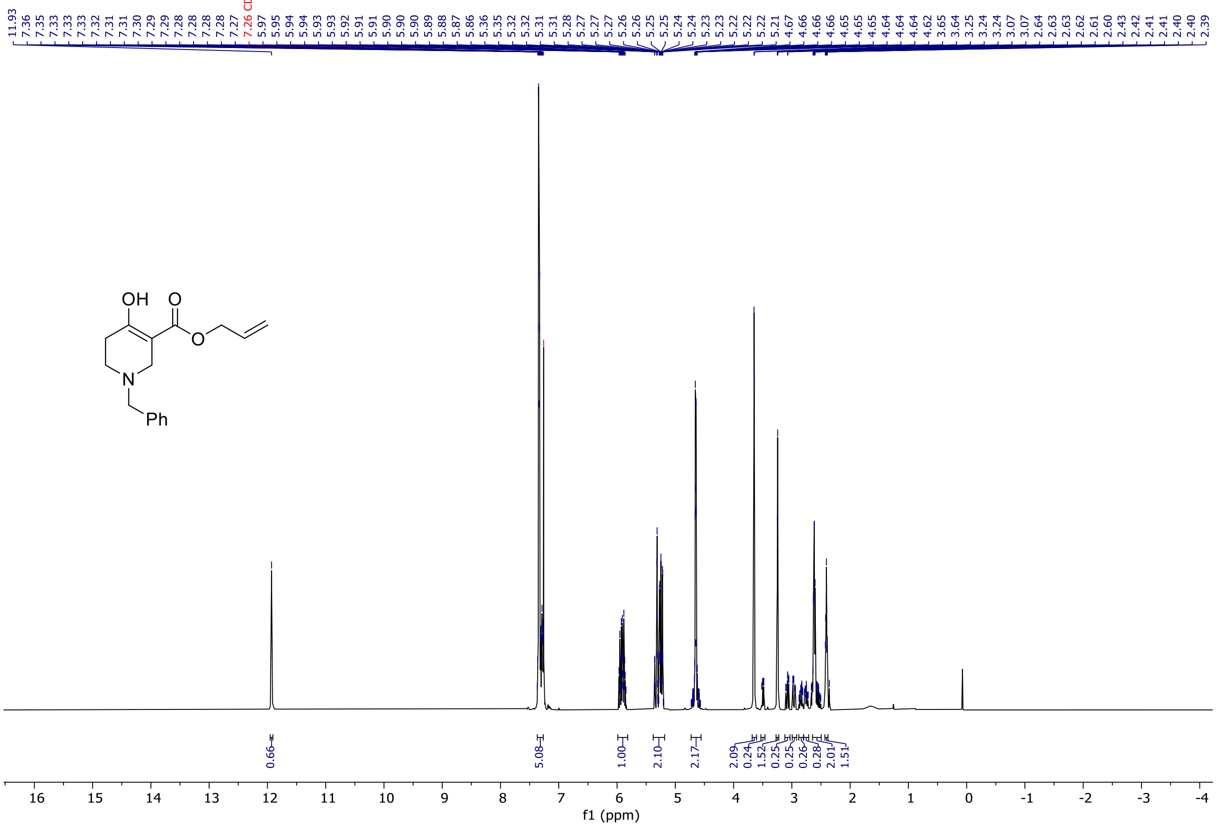

^{13}C ($CDCl_3$, 101 MHz)

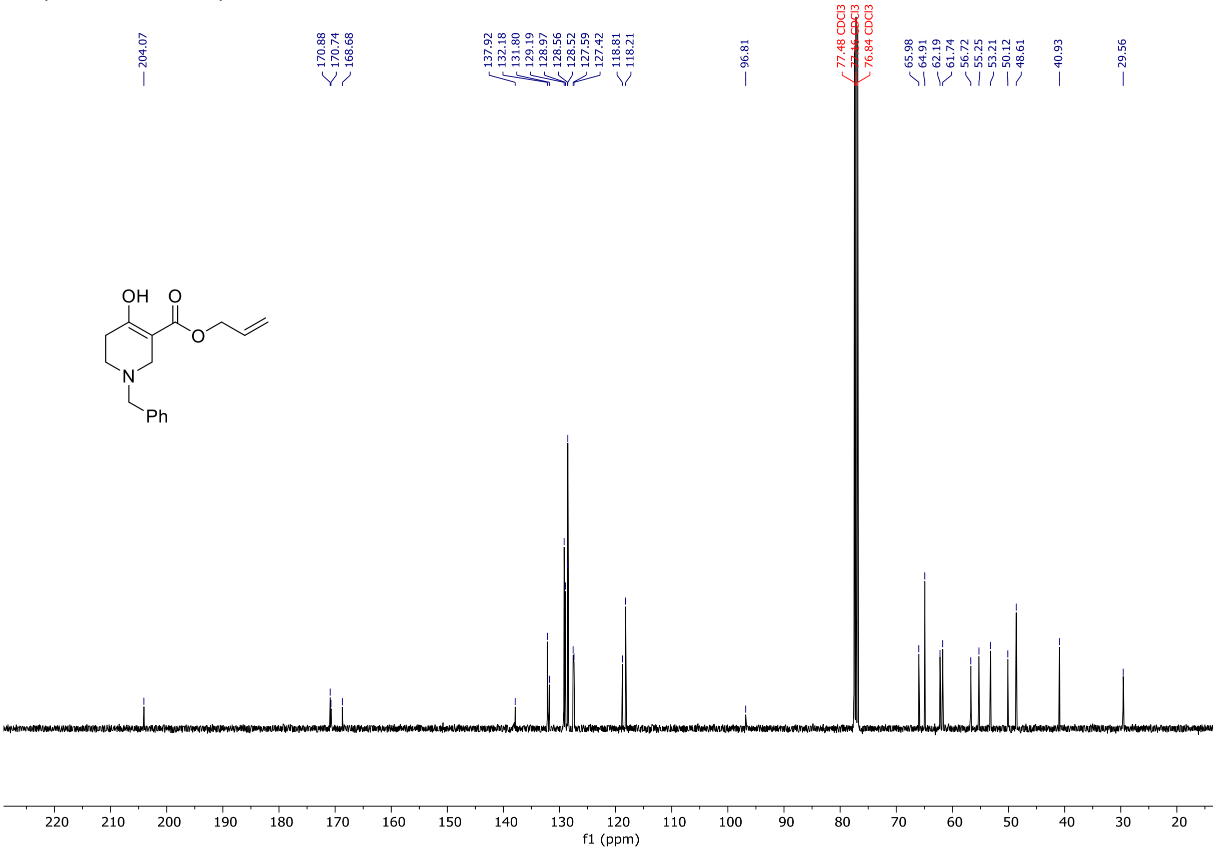

Compound S5

^{1}H ($CDCl_3$, 400 MHz)

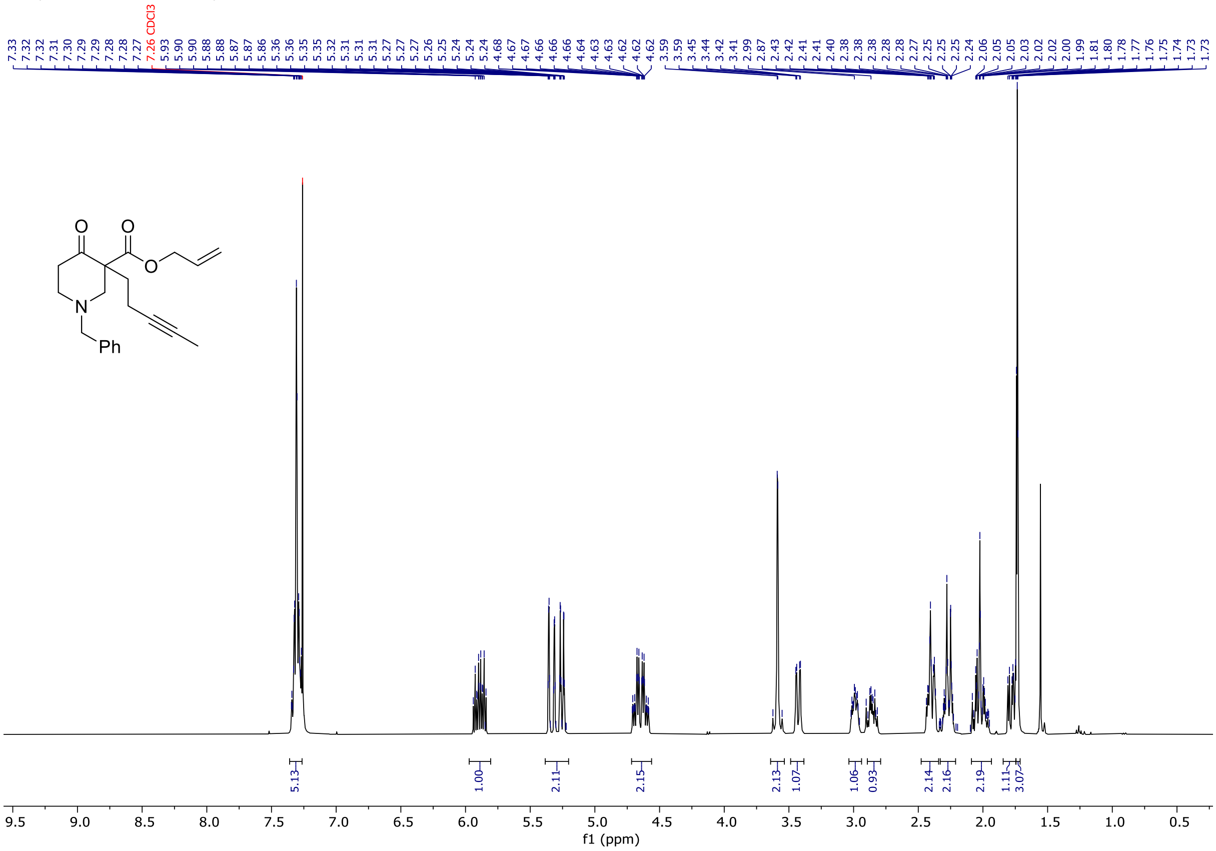

^{13}C ($CDCl_3$, 101 MHz)

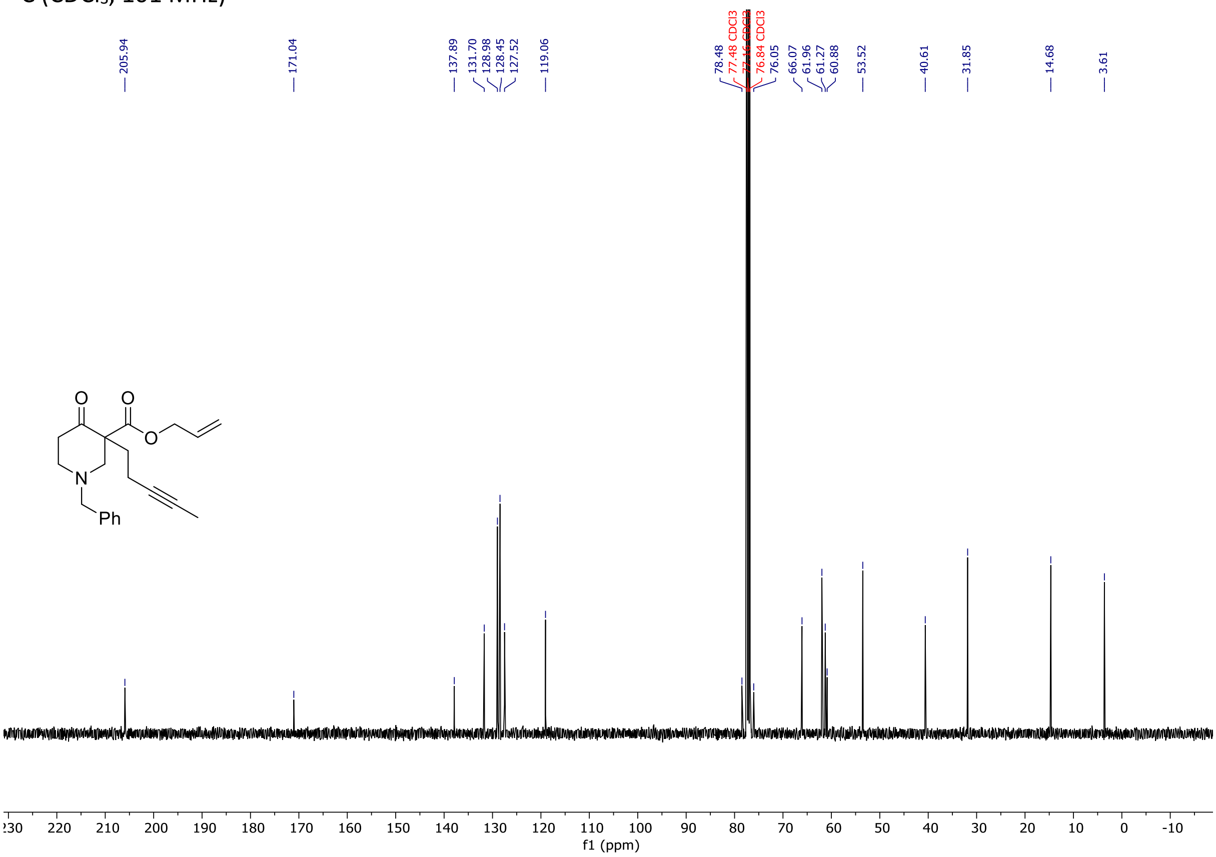

Compound S6

^{1}H ($CDCl_3$, 400 MHz)

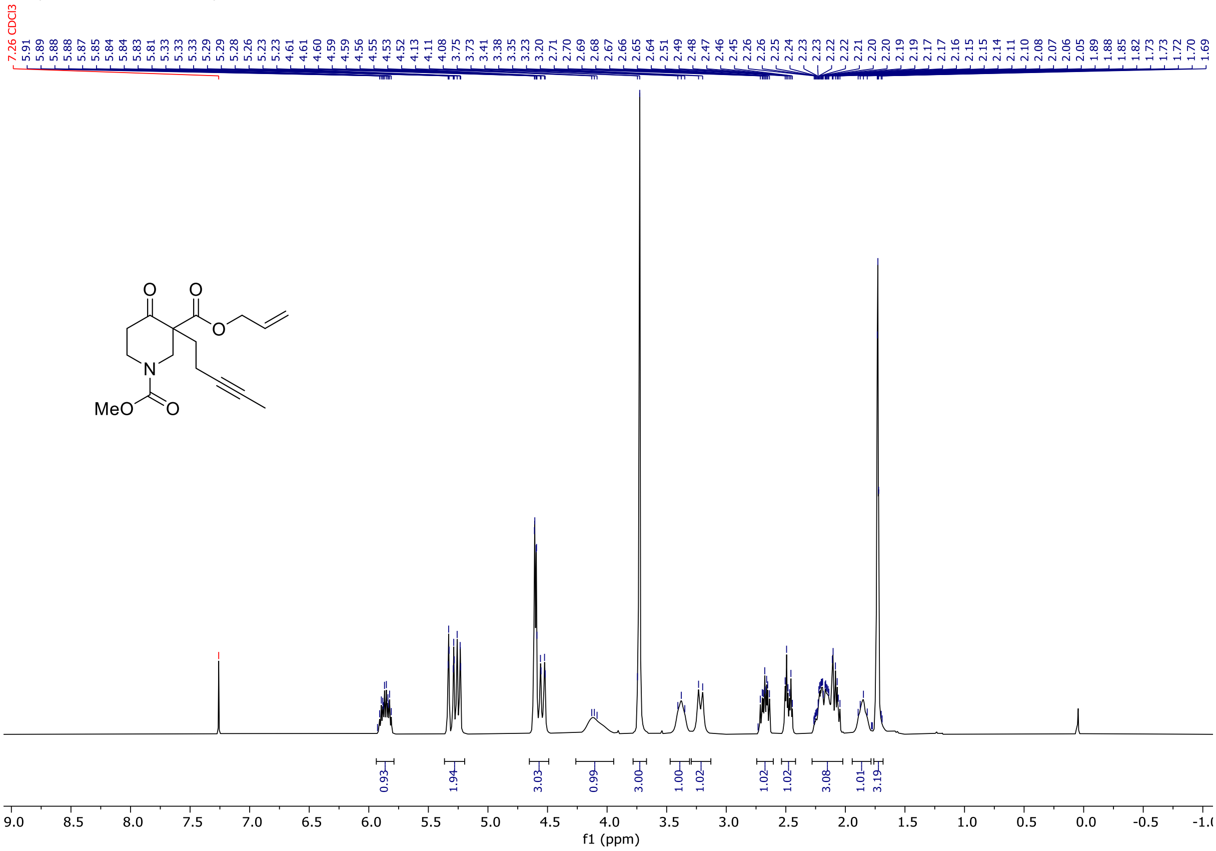

^{13}C ($CDCl_3$, 101 MHz)

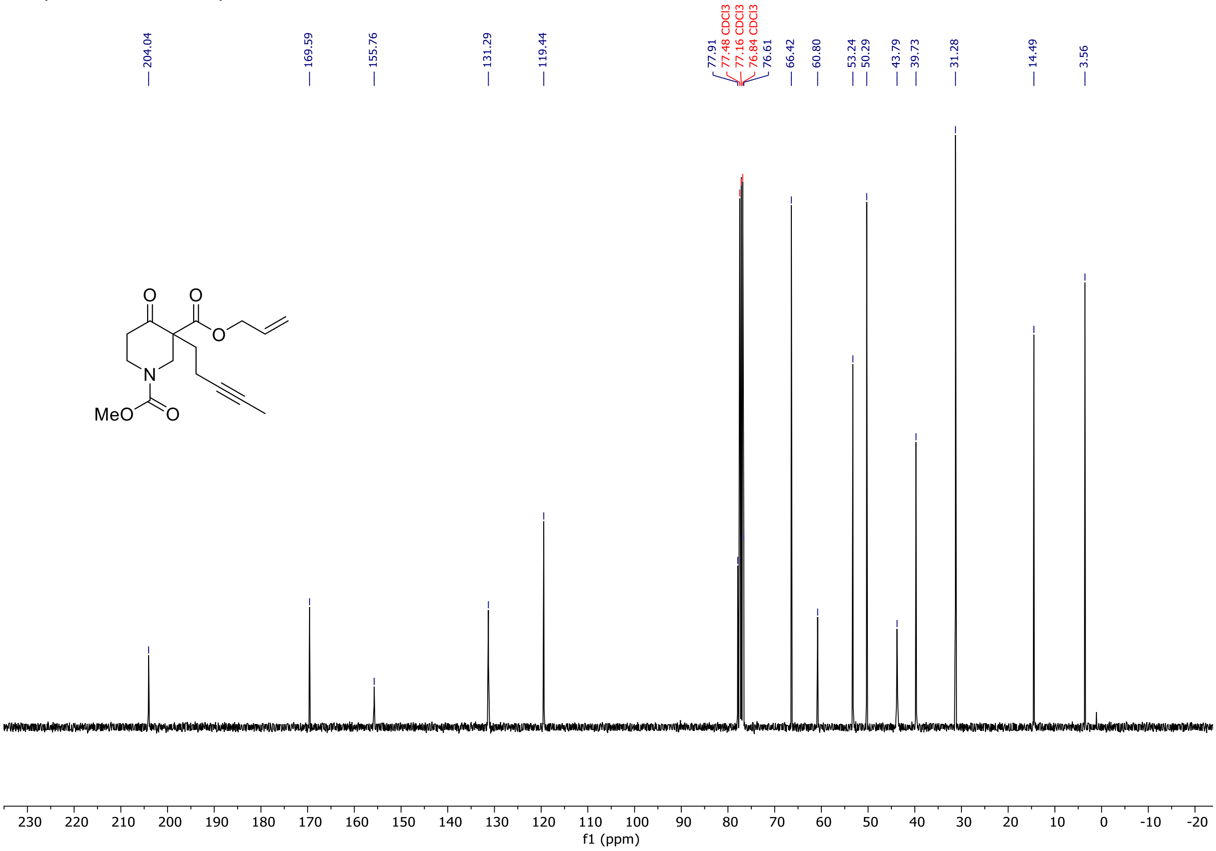

Compound 14

^{1}H ($CDCl_3$, 400 MHz)

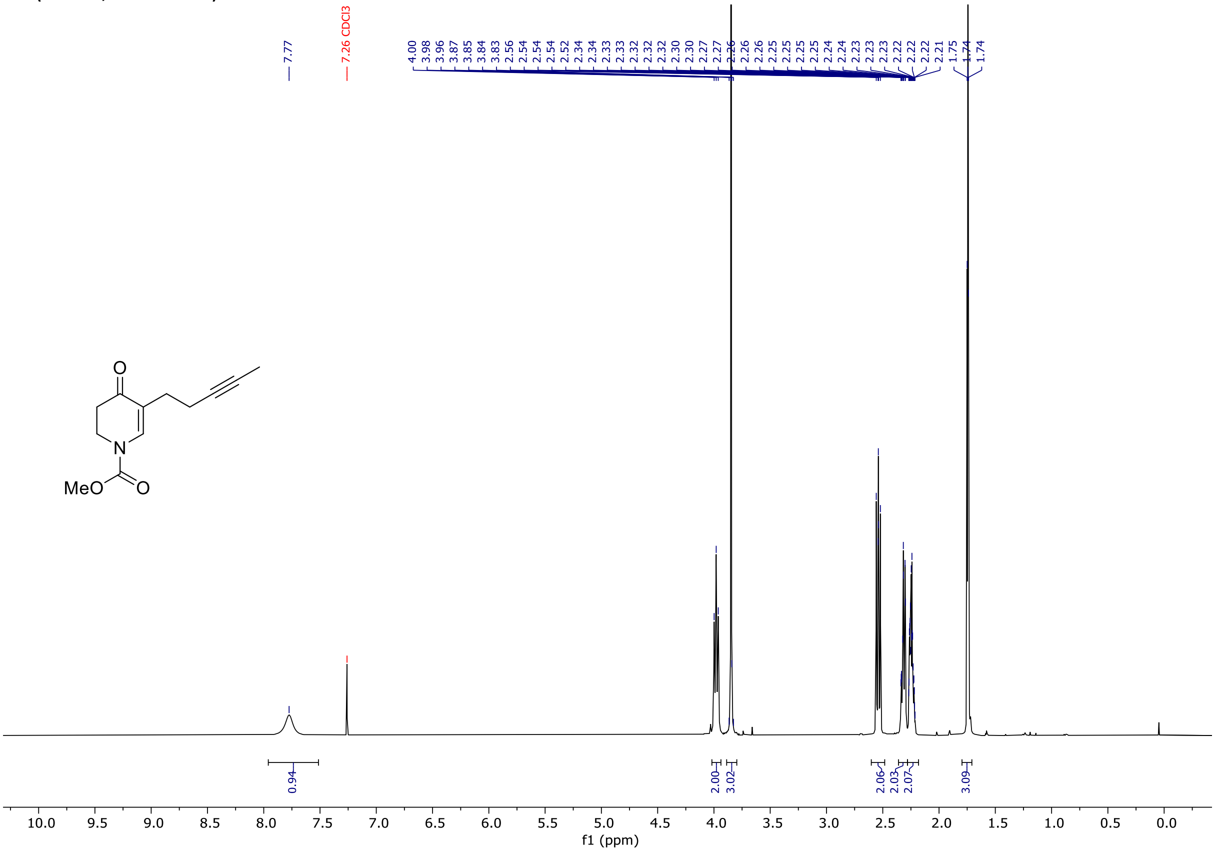

^{13}C ($CDCl_3$, 101 MHz)

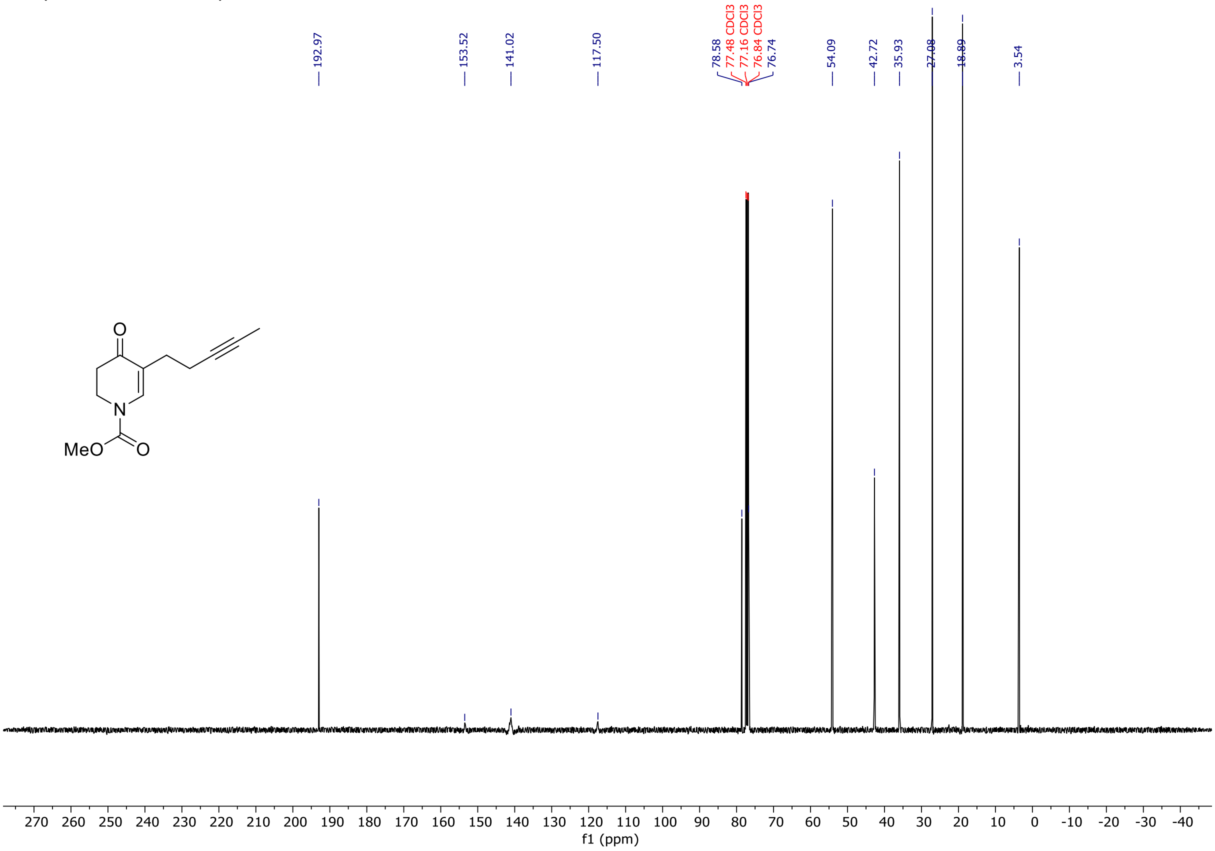

Compound 17

^1H ($CDCl_3$, 400 MHz)

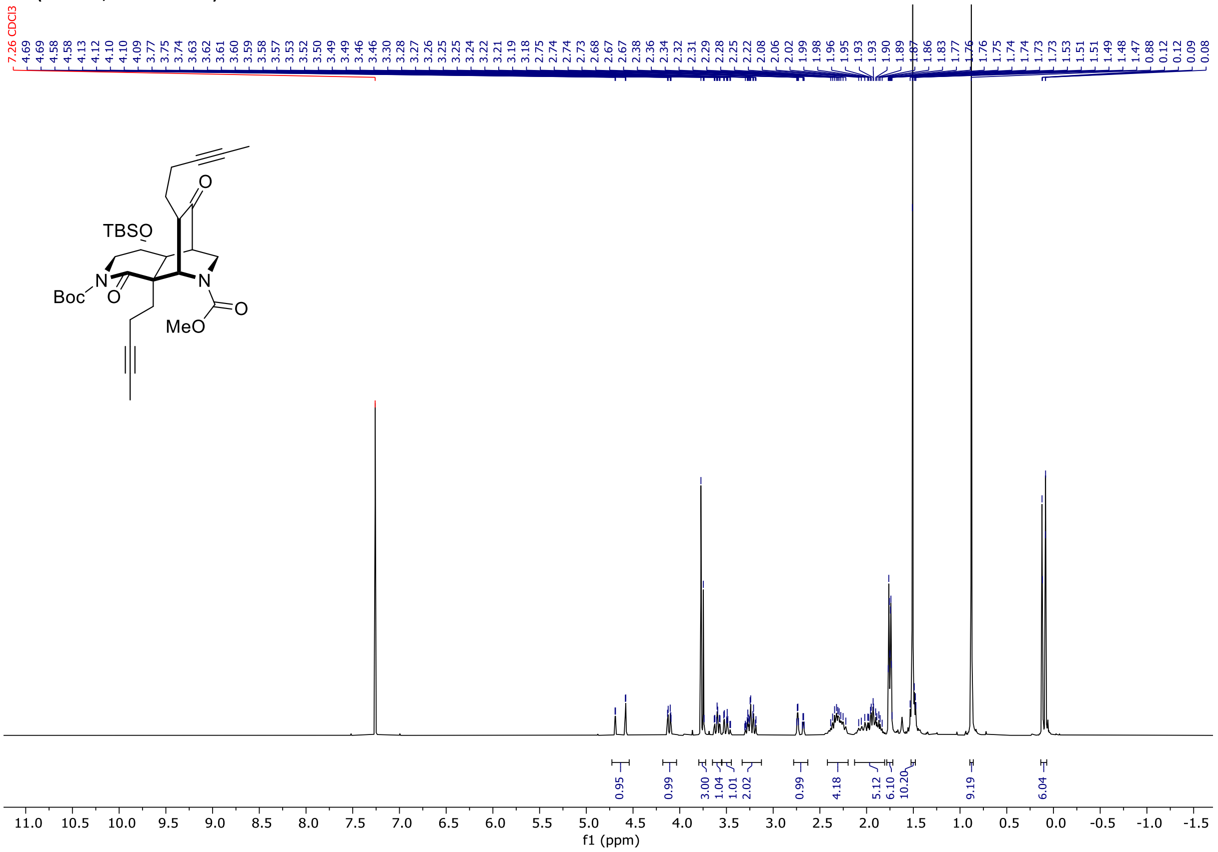

^{13}C ($CDCl_3$, 101 MHz)

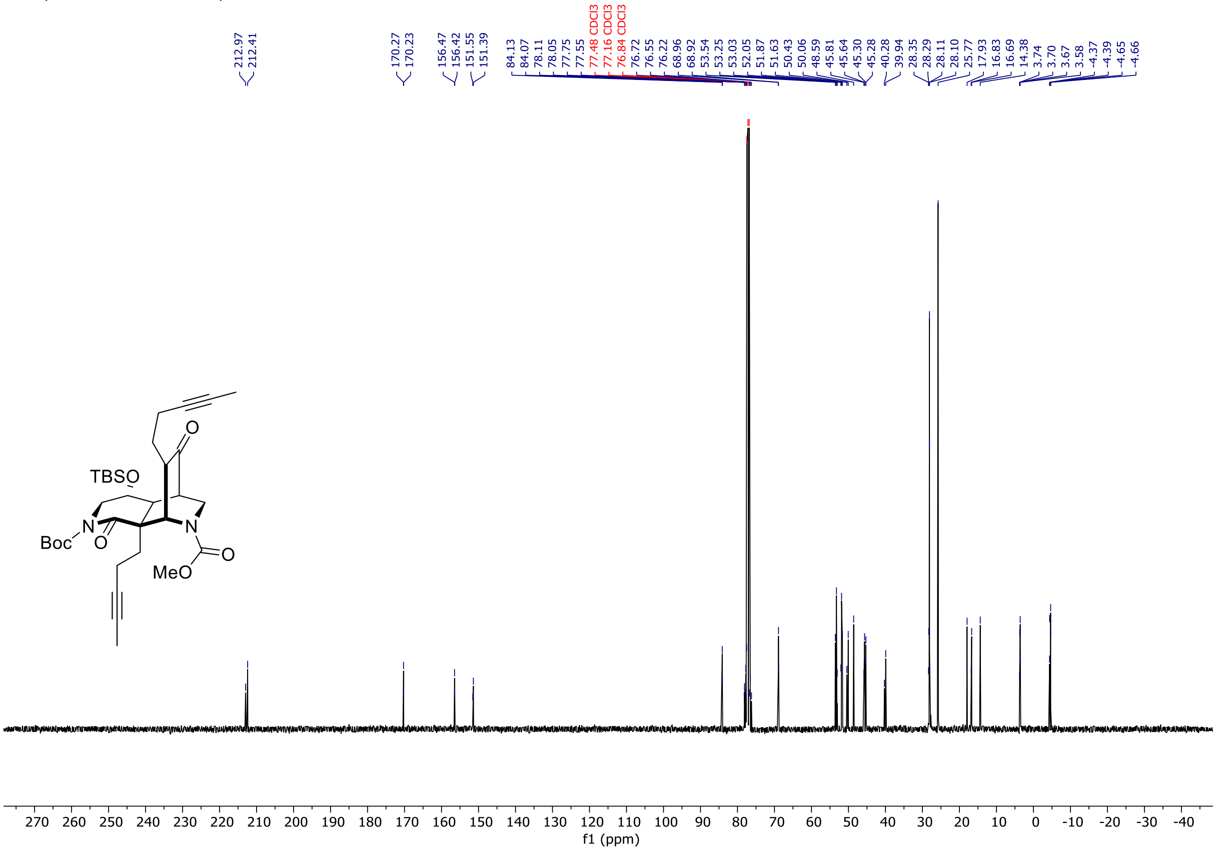

Compound S7

^{1}H ($CDCl_3$, 400 MHz)

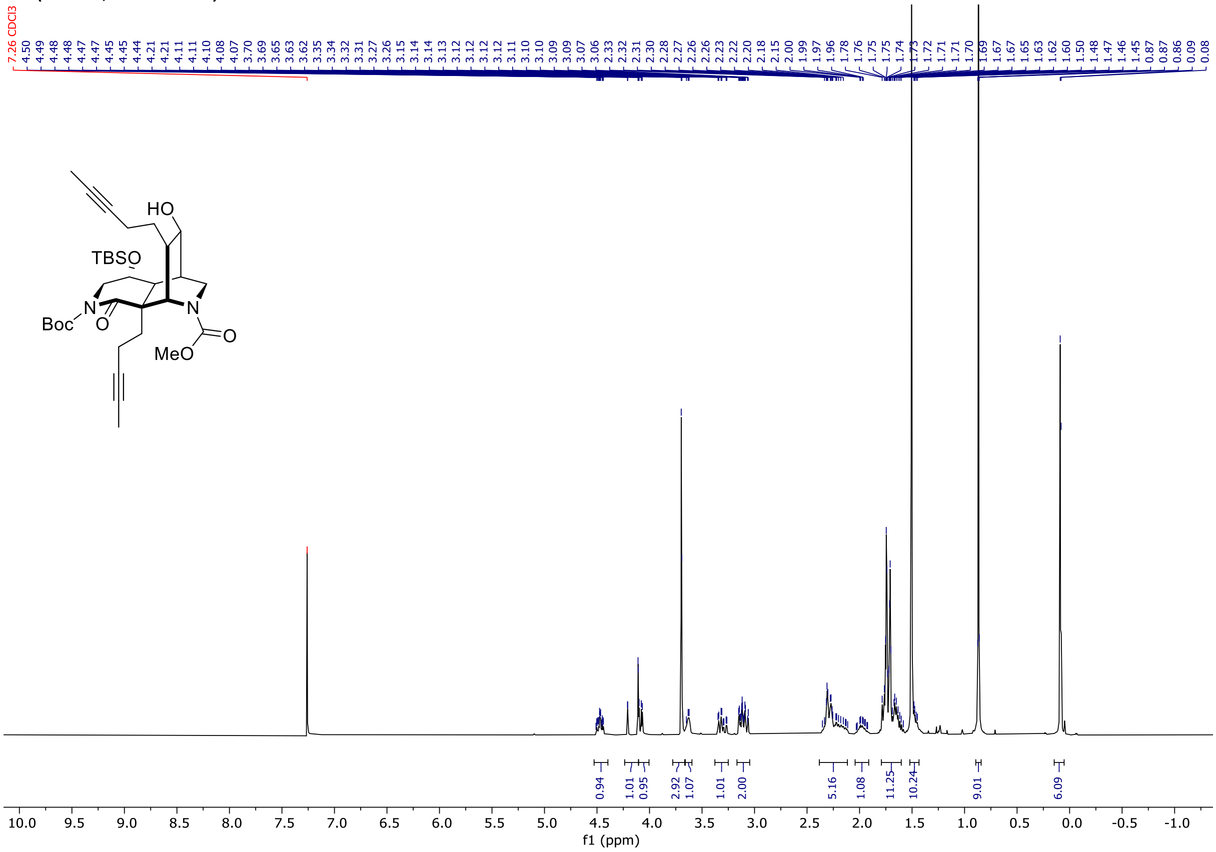

^{13}C ($CDCl_3$, 101 MHz)

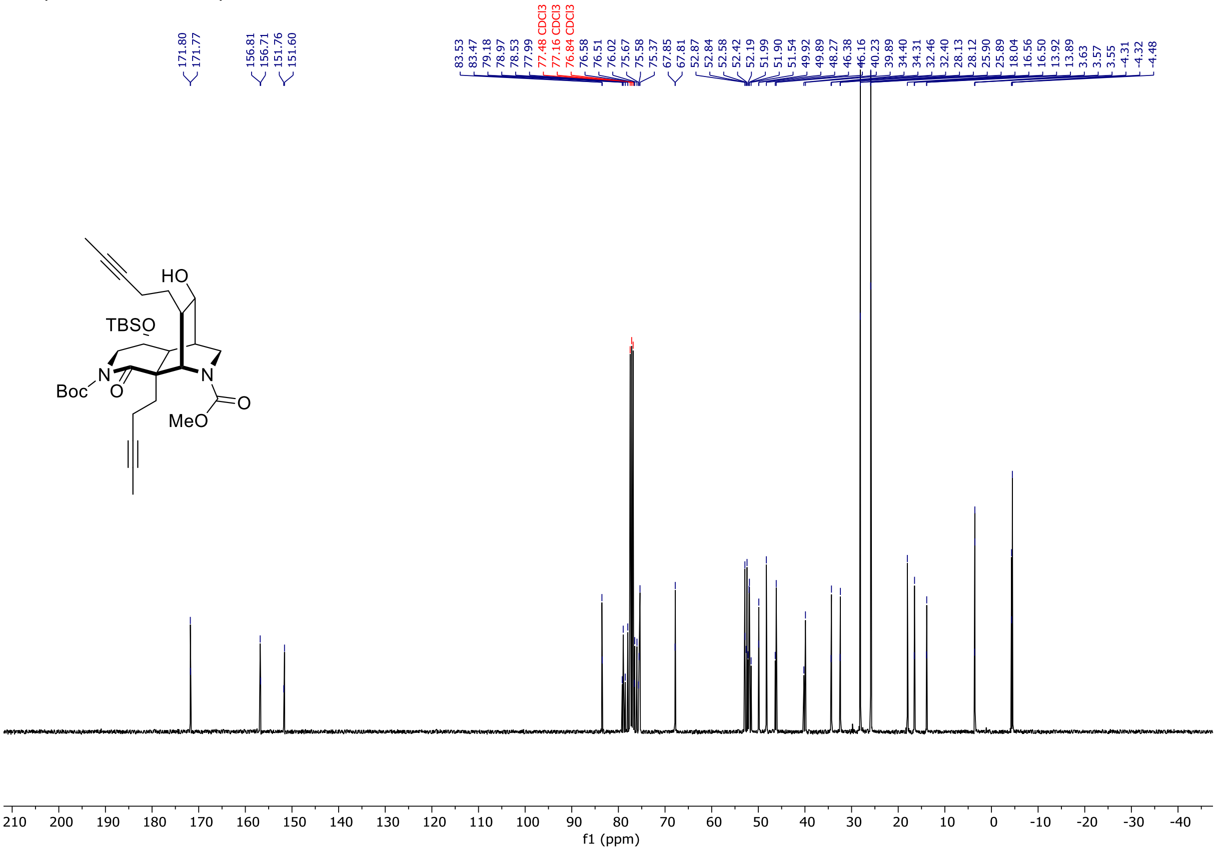

Compound S8

^1H ($CDCl_3$, 400 MHz)

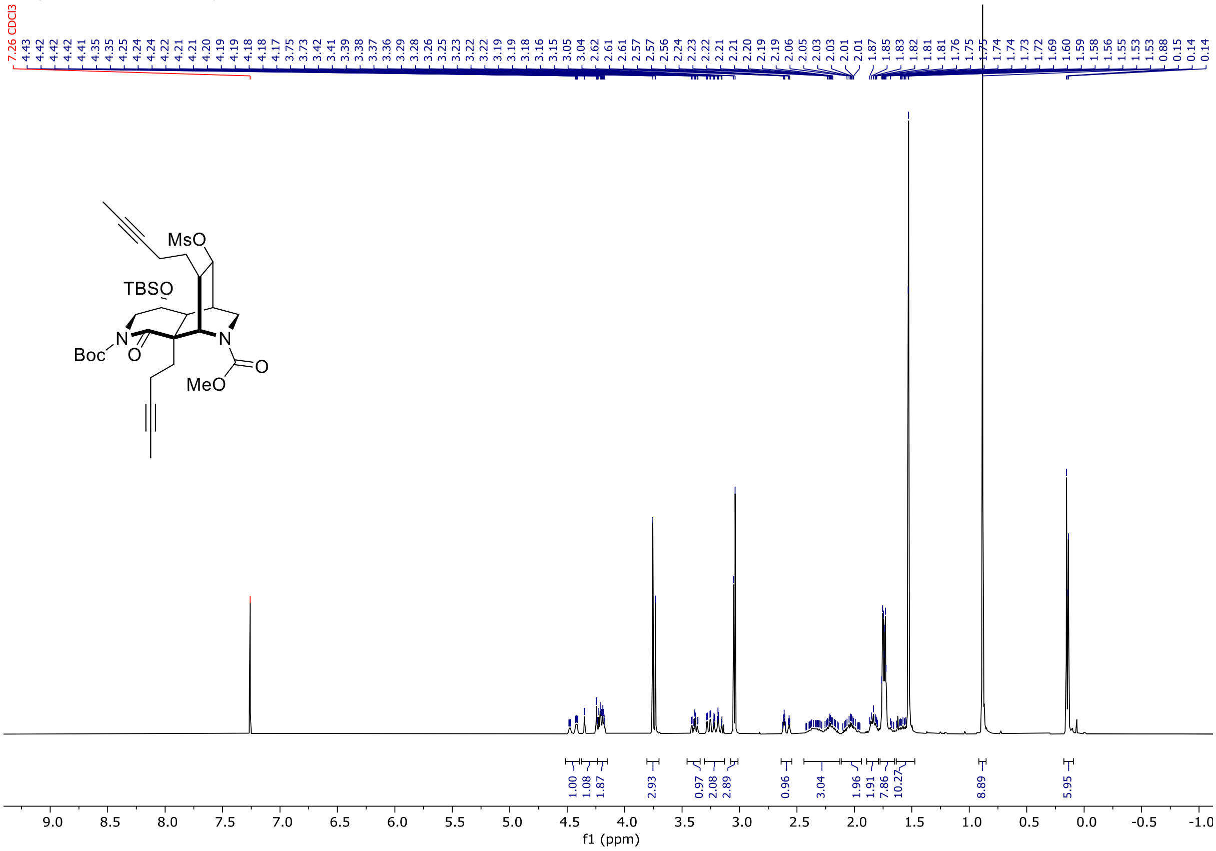

^{13}C ($CDCl_3$, 101 MHz)

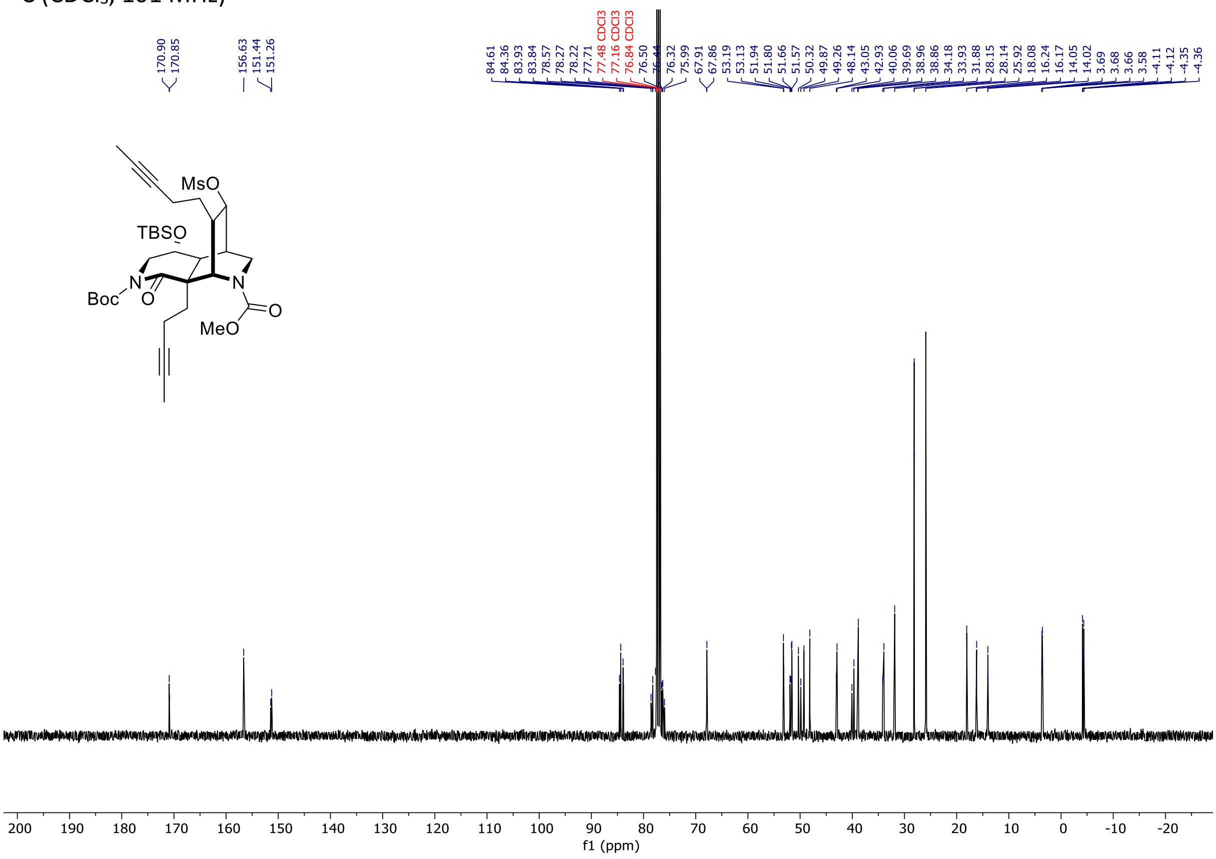

Compound 18

^{1}H ($CDCl_3$, 400 MHz)

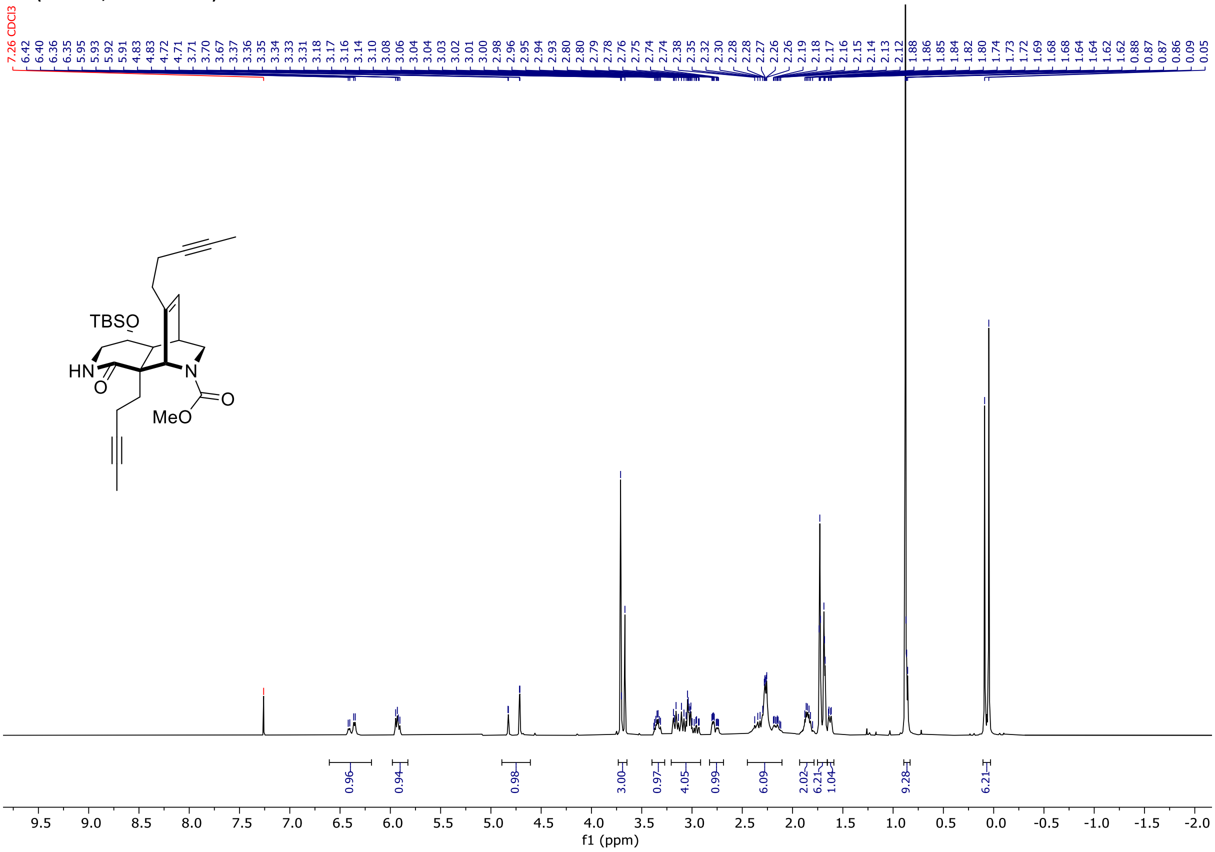

^{13}C ($CDCl_3$, 101 MHz)

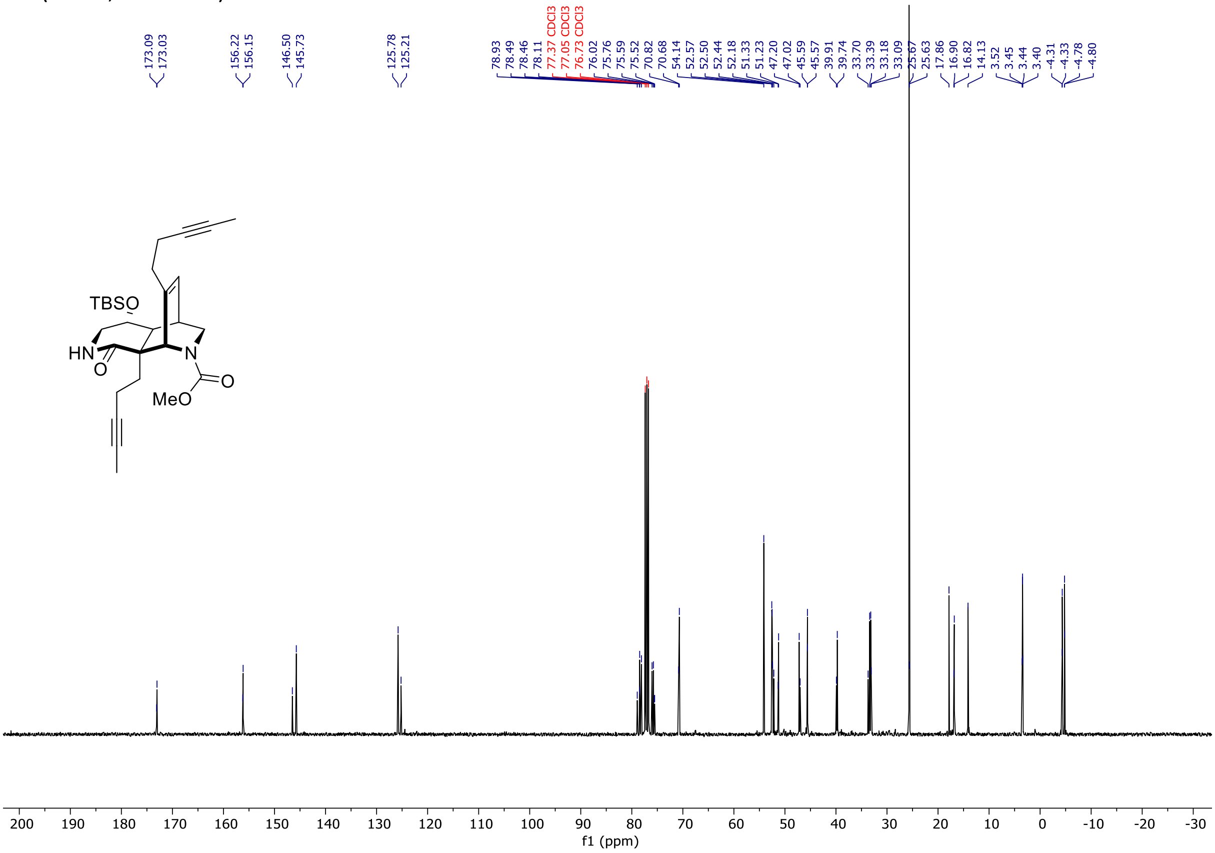

Compound S9

^{1}H ($CDCl_3$, 400 MHz)

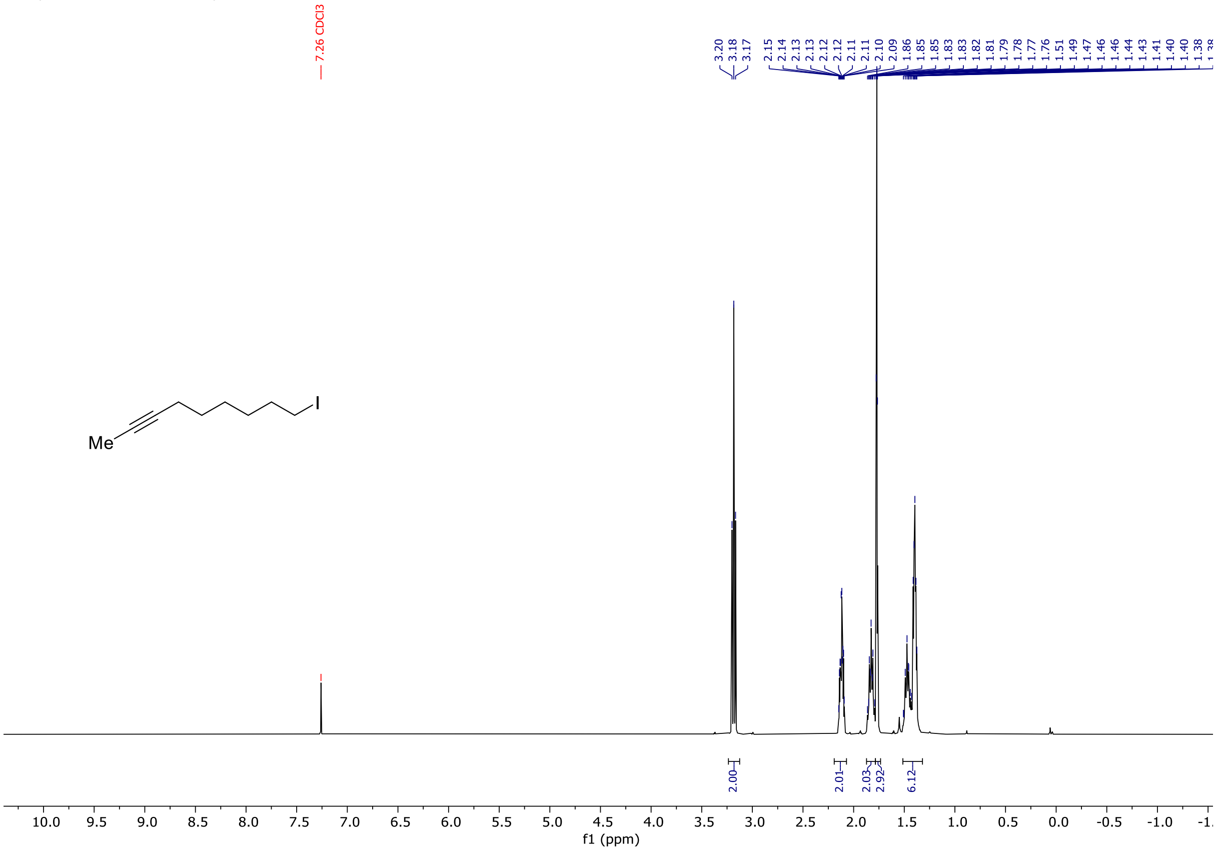

^{13}C ($CDCl_3$, 101 MHz)

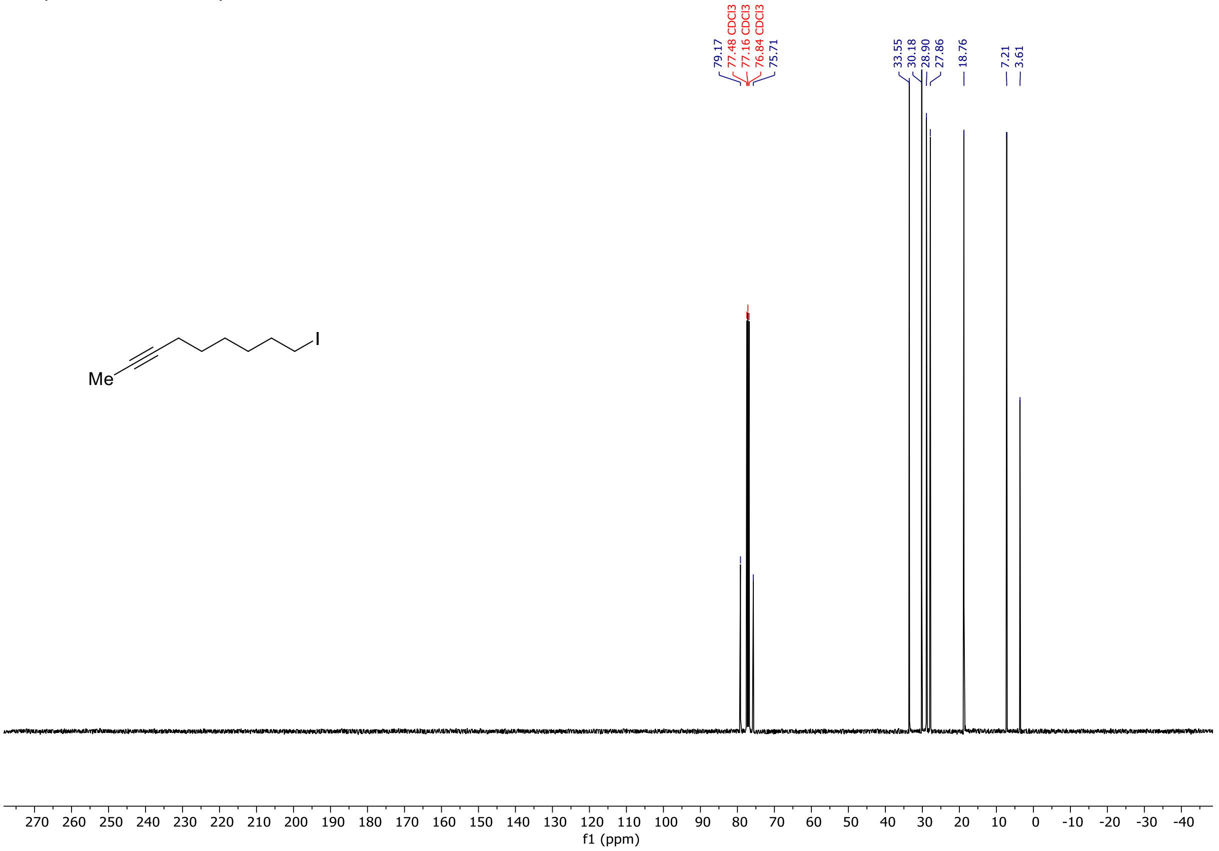

Compound S10

^{1}H ($CDCl_3$, 400 MHz)

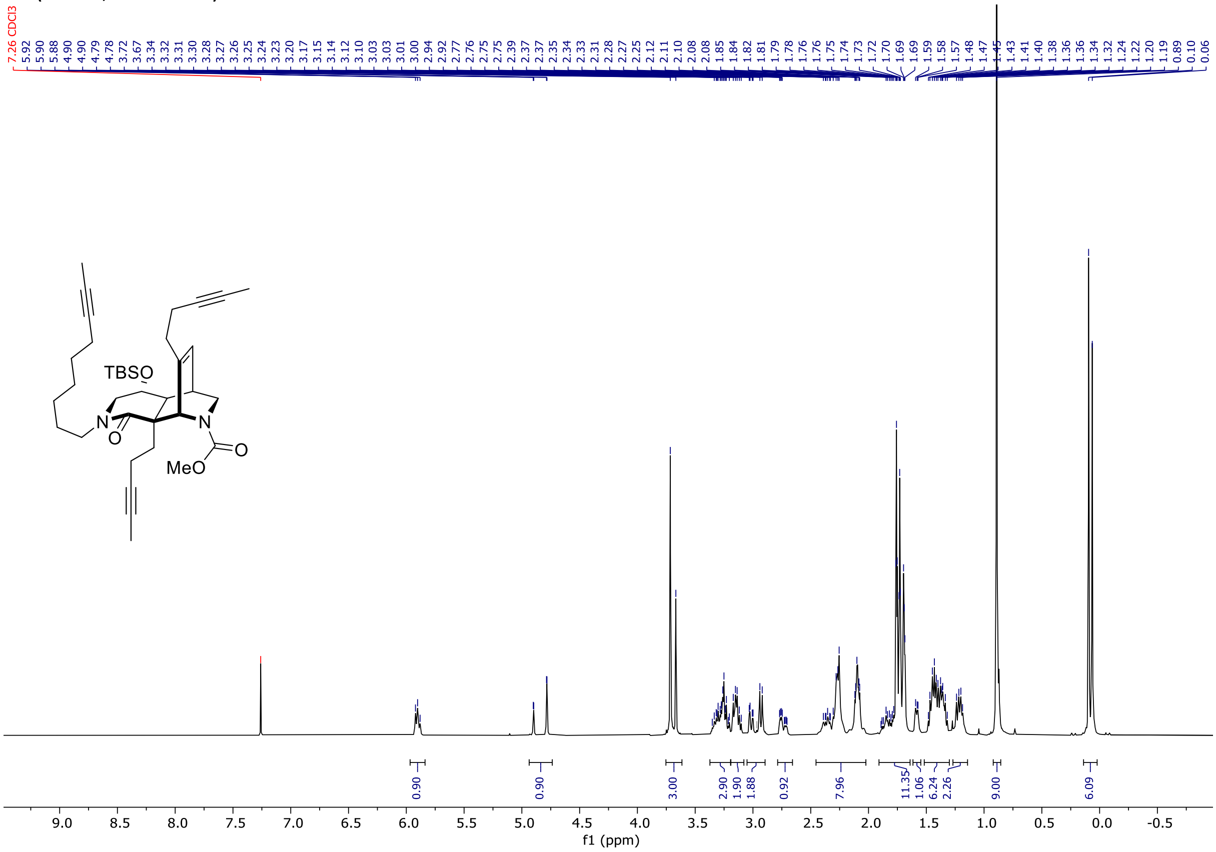

^{13}C ($CDCl_3$, 101 MHz)

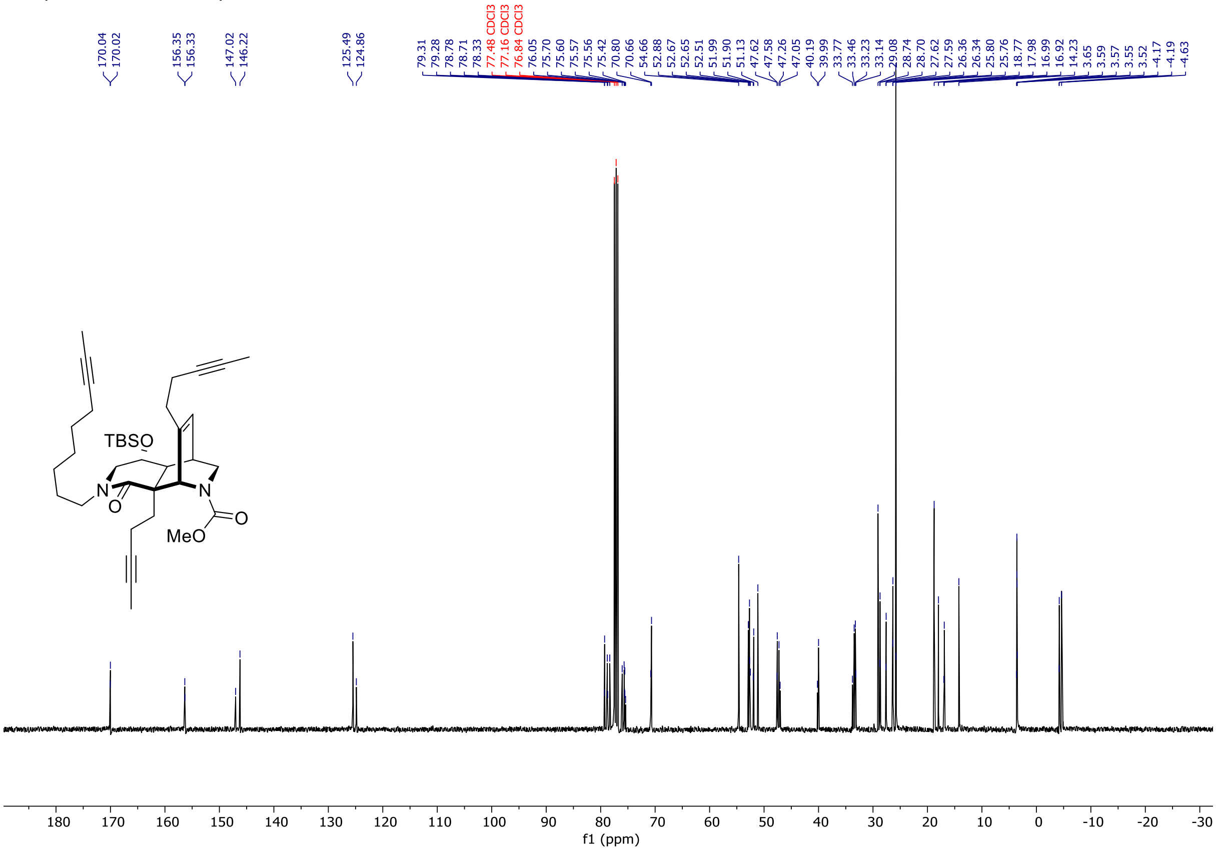

Compound 19

^{1}H ($CDCl_3$, 400 MHz)

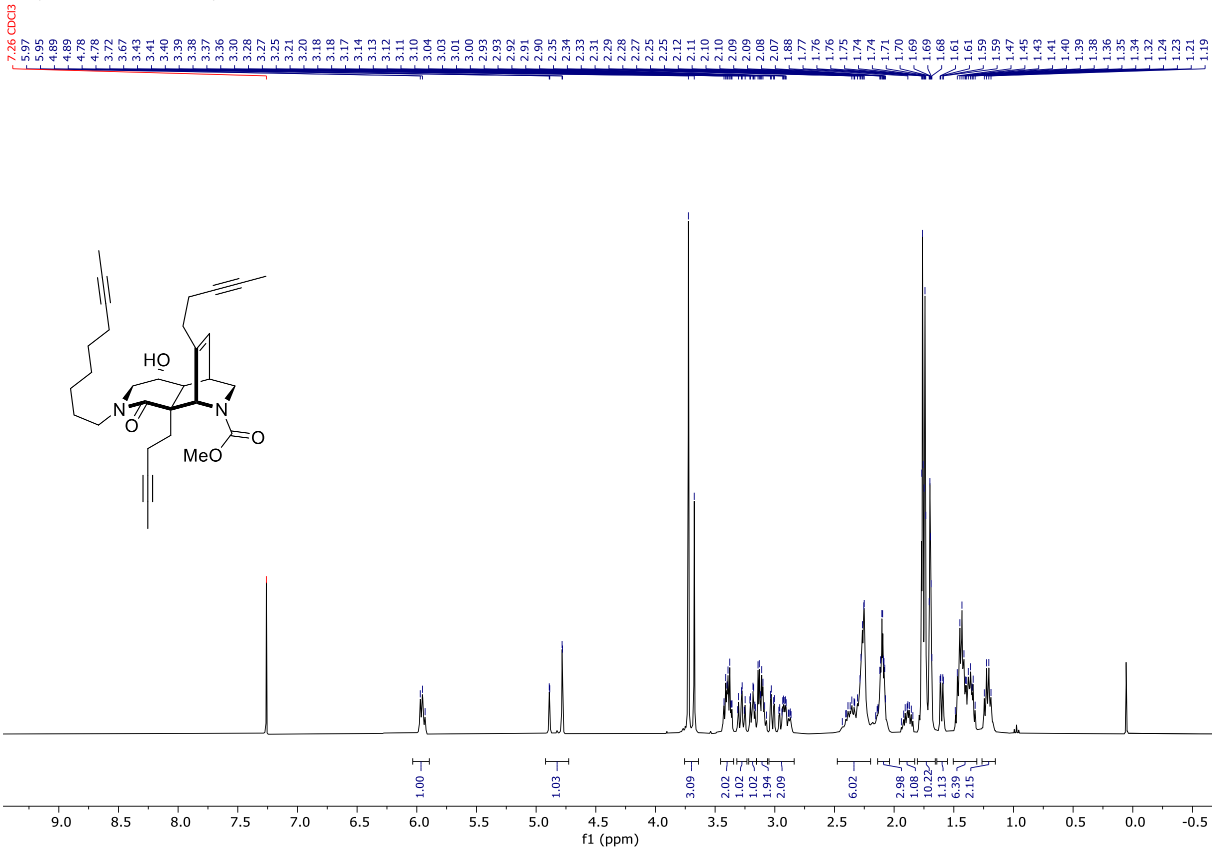

^{13}C ($CDCl_3$, 101 MHz)

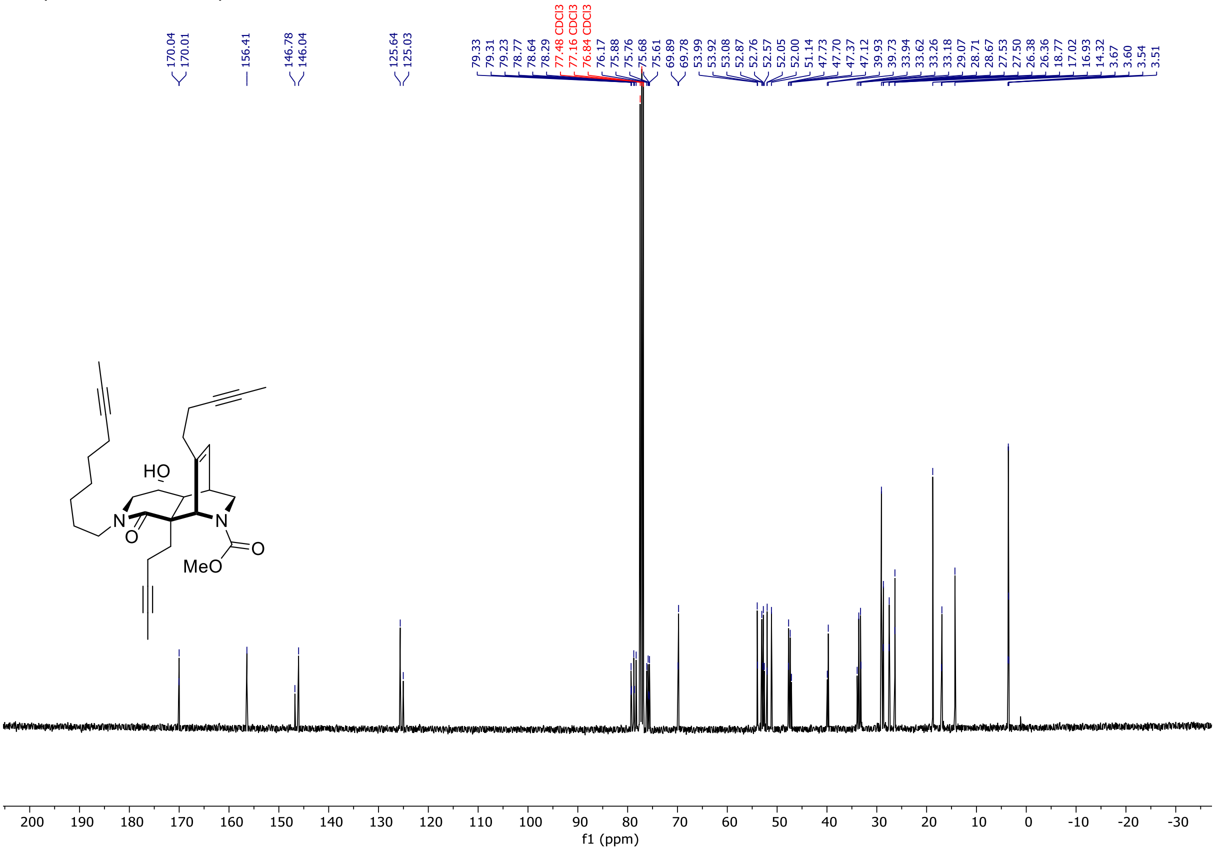

Compound S11

^{1}H ($CDCl_3$, 400 MHz)

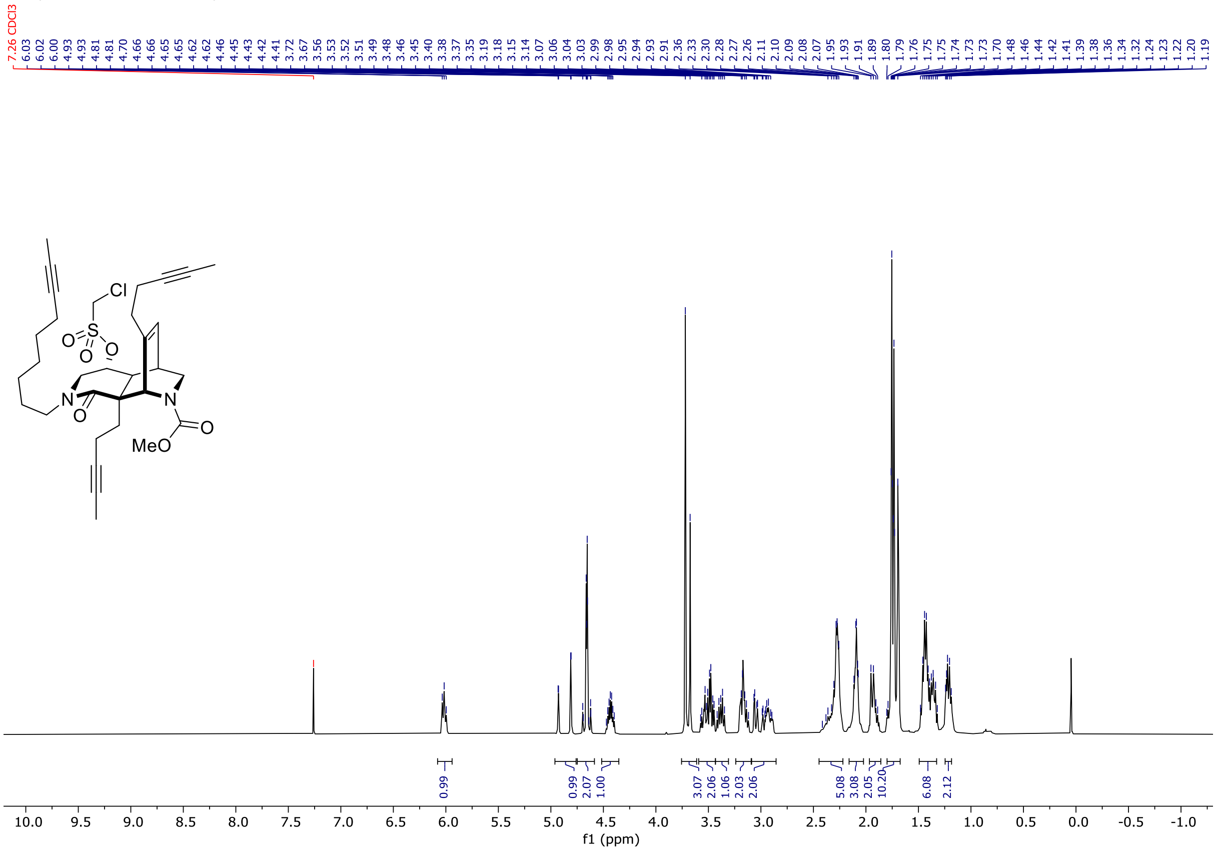

^{13}C ($CDCl_3$, 101 MHz)

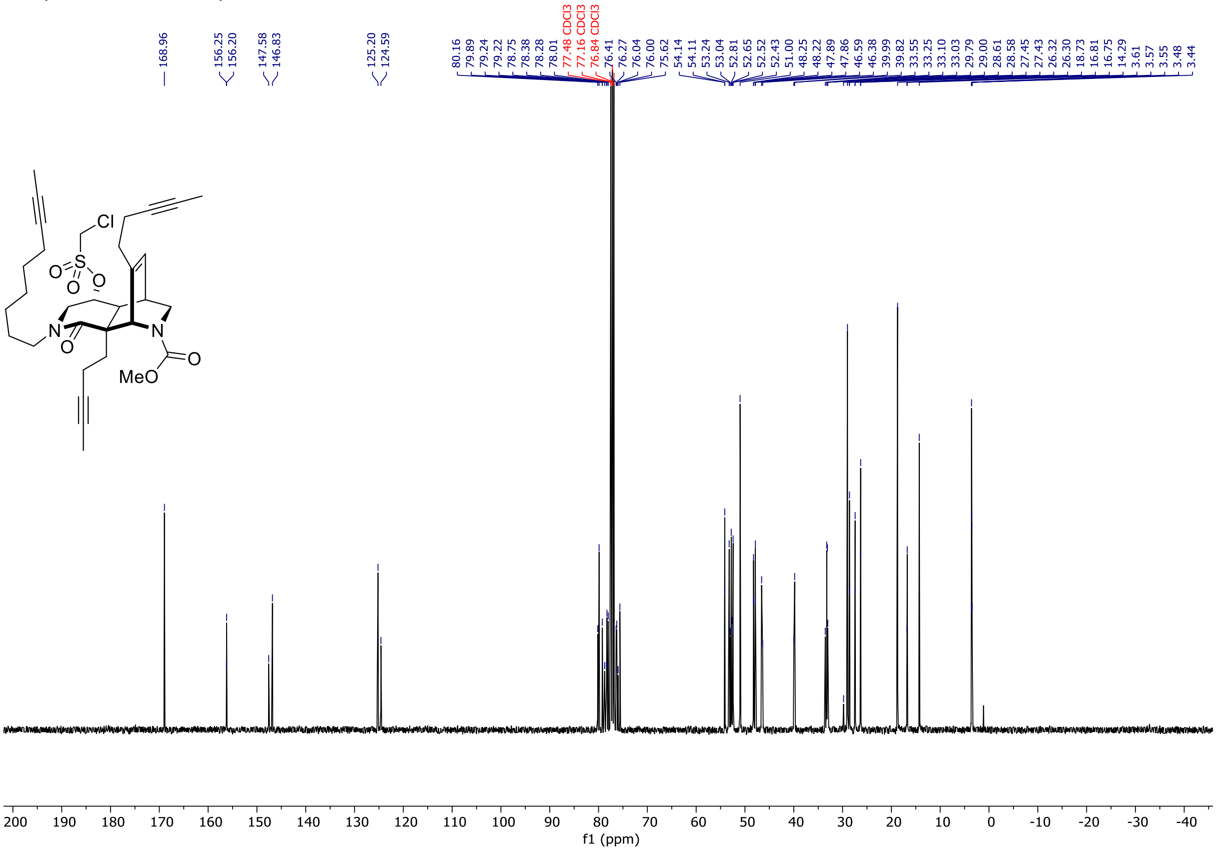

Compound 20

^{1}H (CDCl$_3$, 400 MHz)

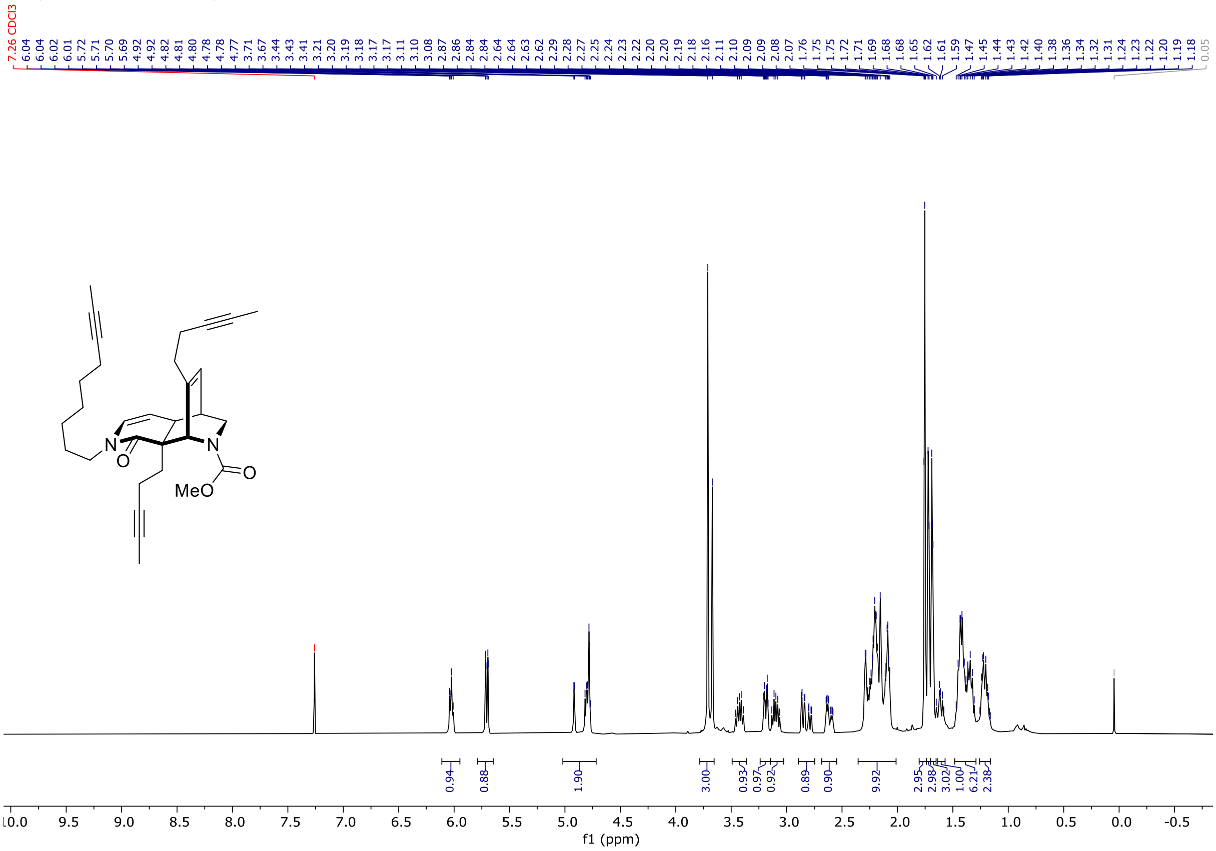

^{13}C (CDCl$_3$, 101 MHz)

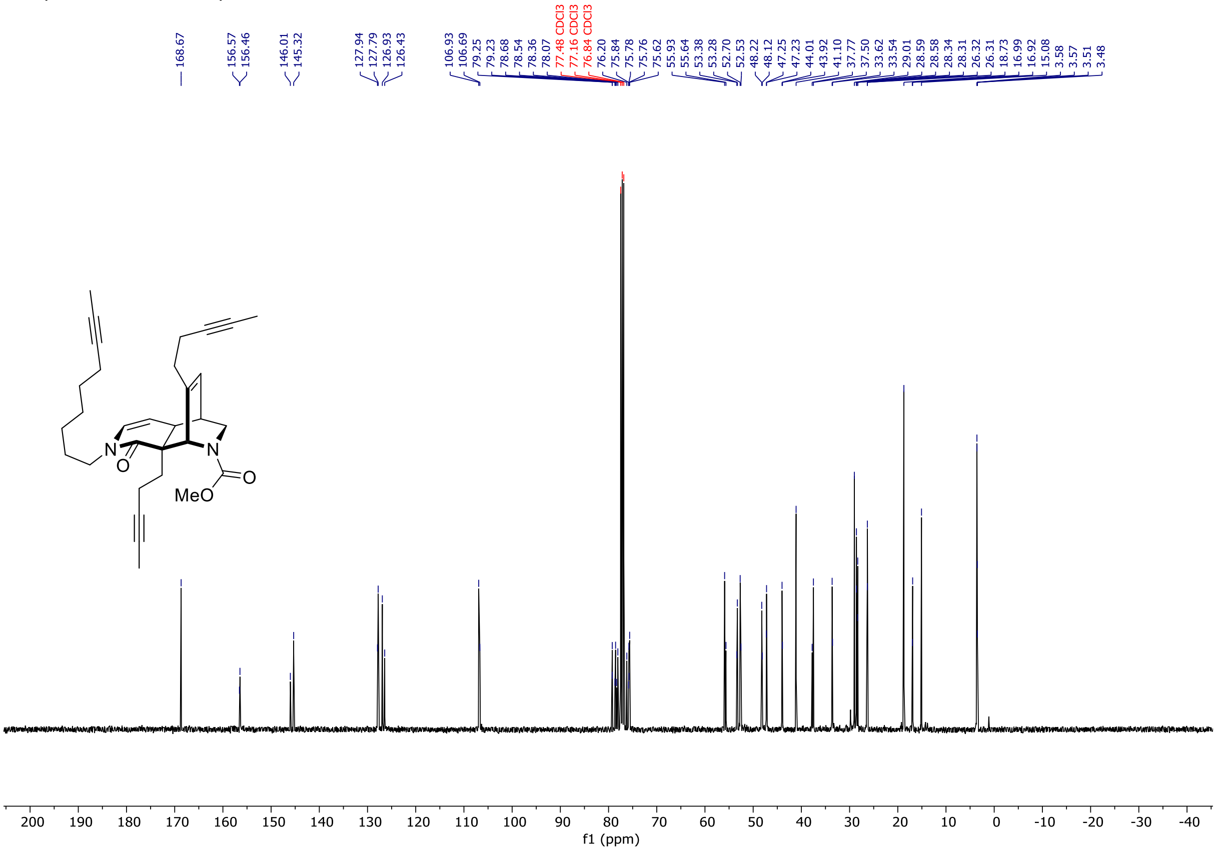

Compound 21

^{1}H ($CDCl_3$, 600 MHz)

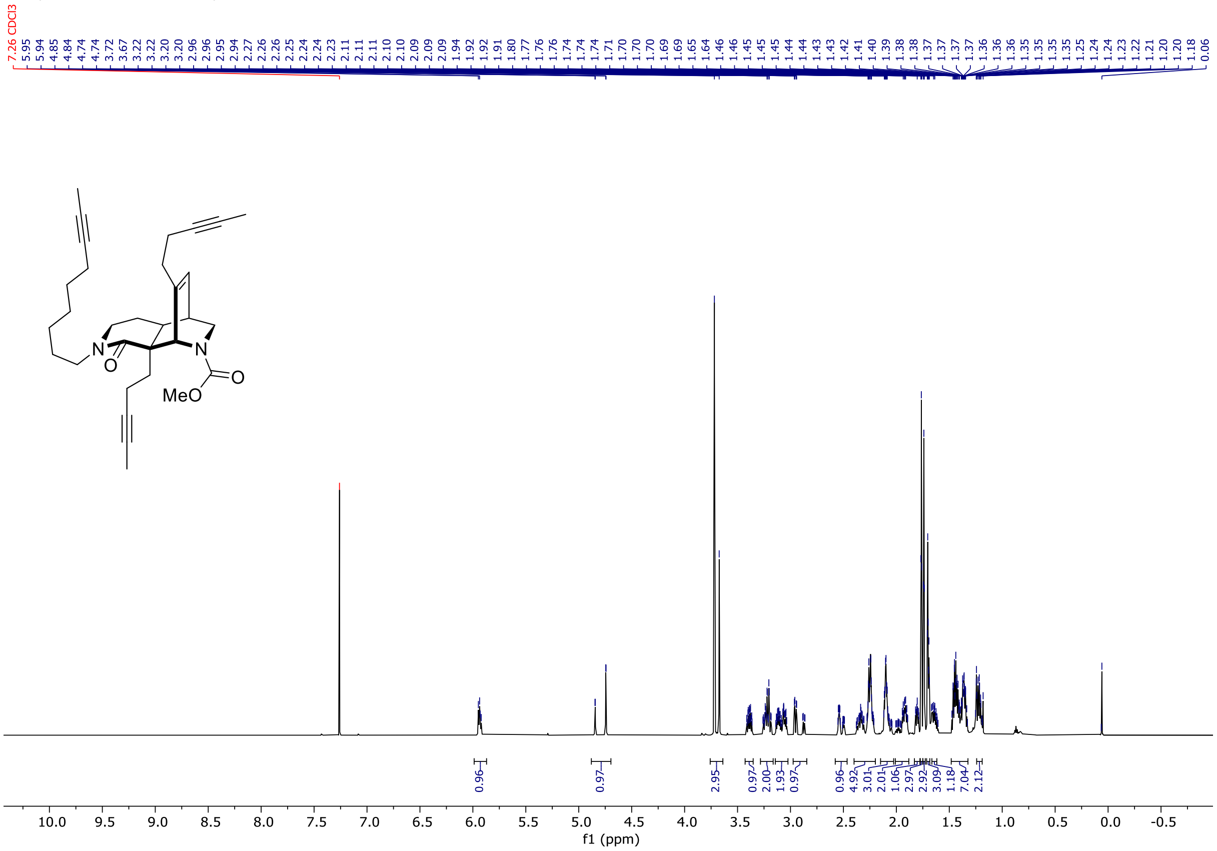

^{13}C ($CDCl_3$, 151 MHz)

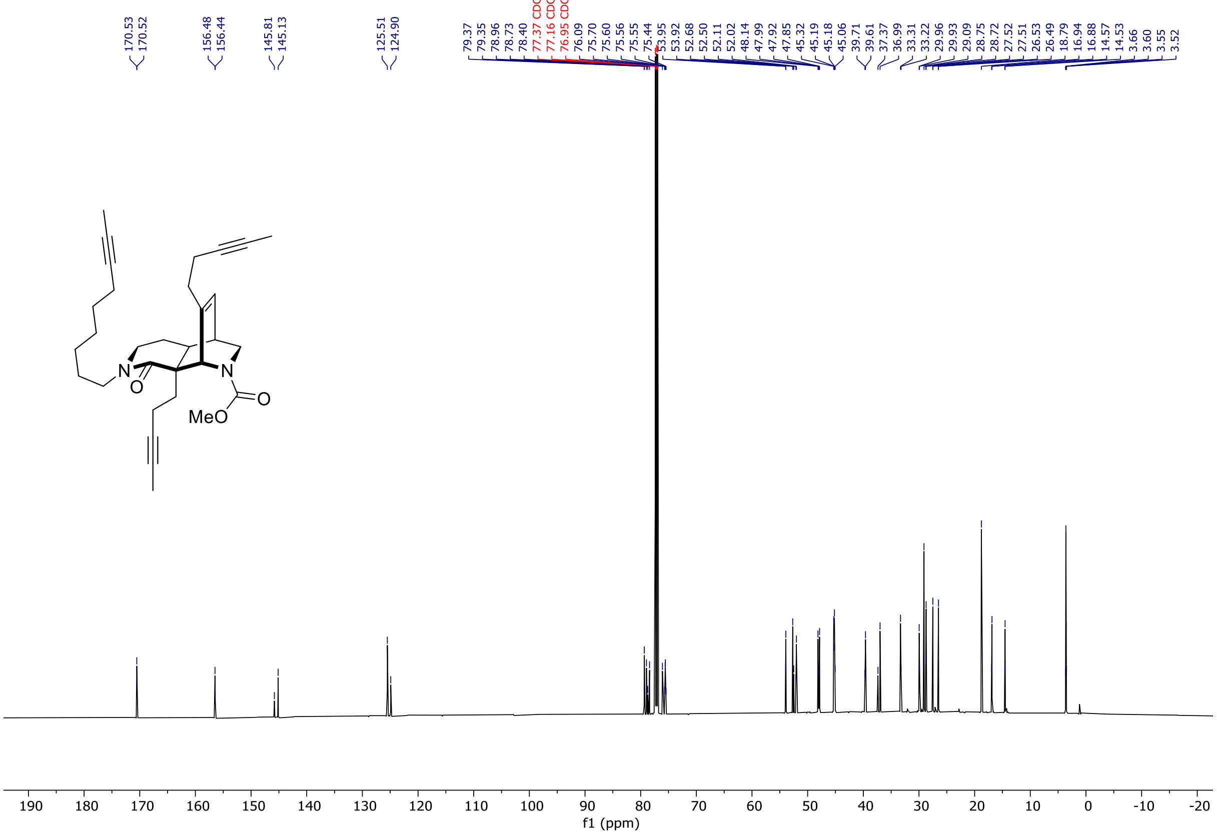

Compound 23

^{1}H ($CDCl_3$, 400 MHz)

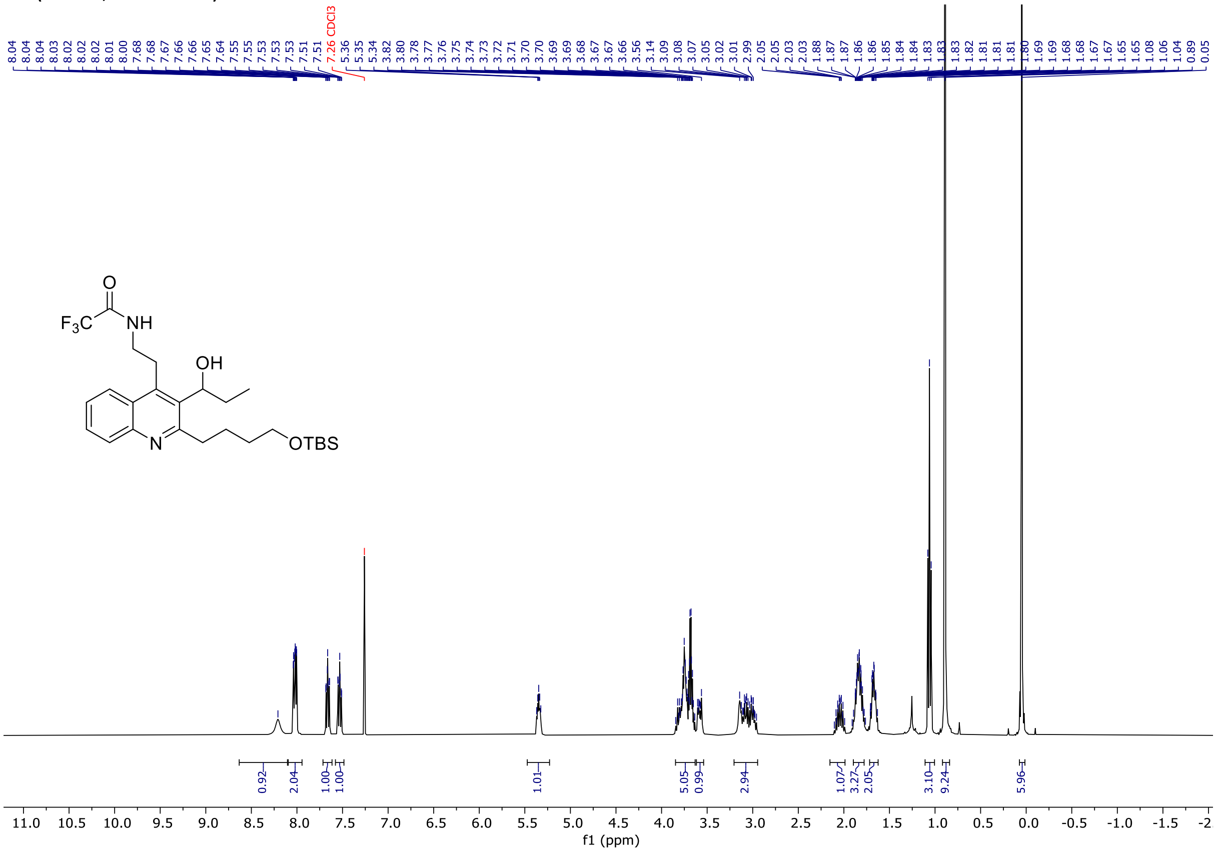

^{13}C ($CDCl_3$, 101 MHz)

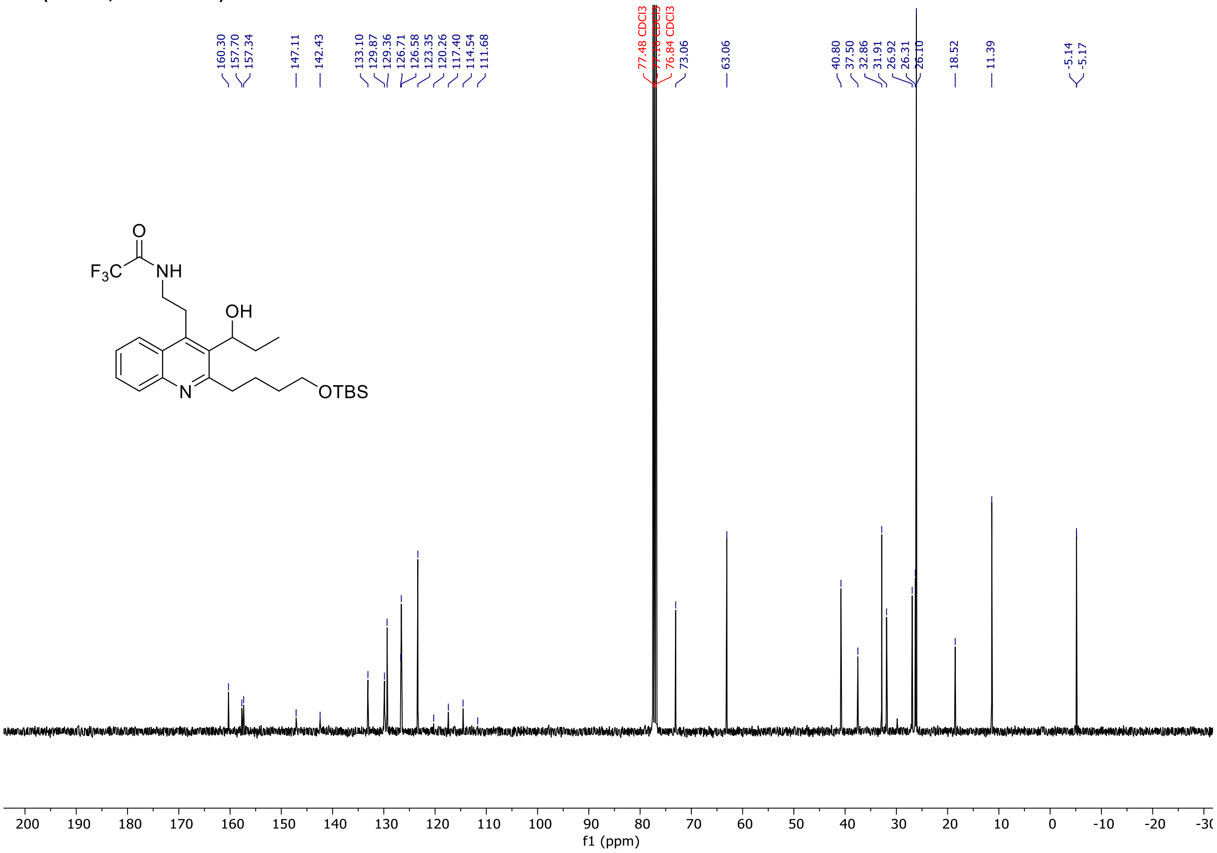

^{19}F ($CDCl_3$, 282 MHz)

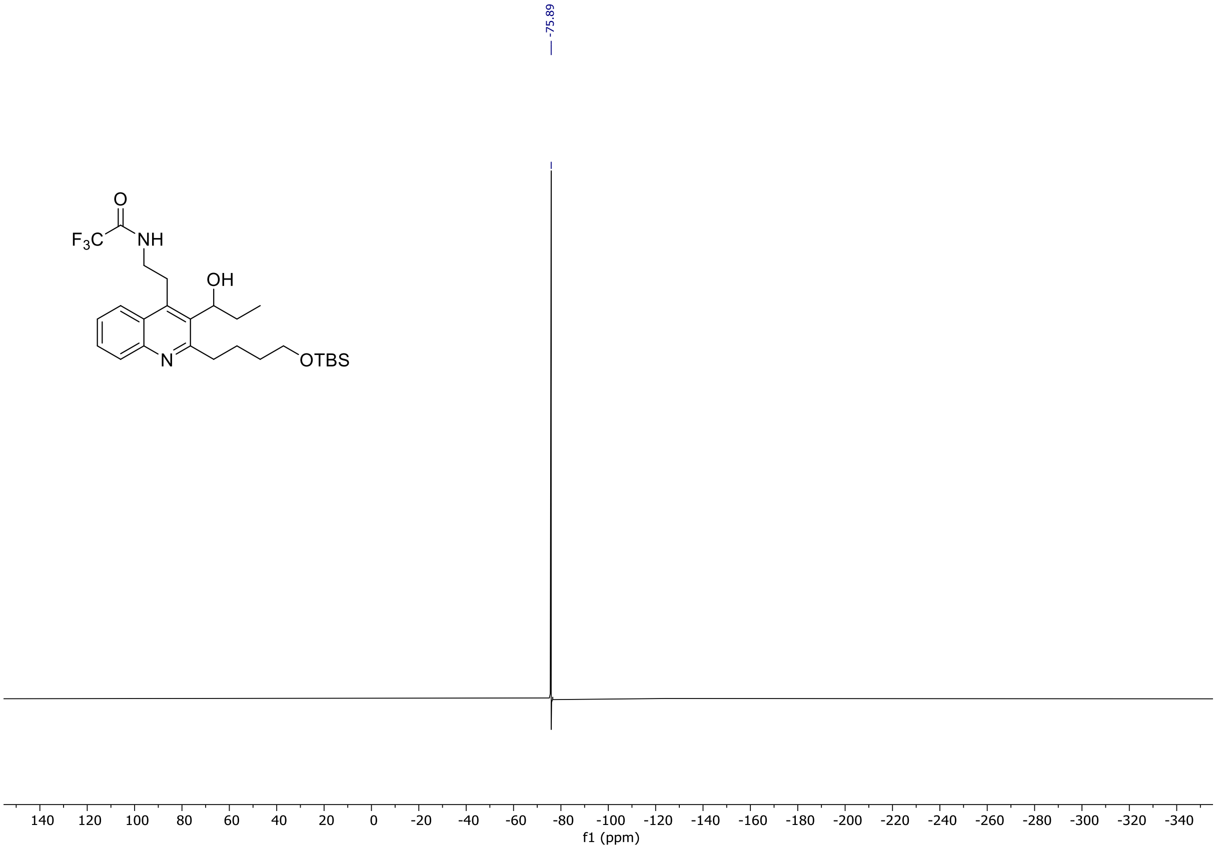

Compound S12

^{1}H ($CDCl_3$, 600 MHz)

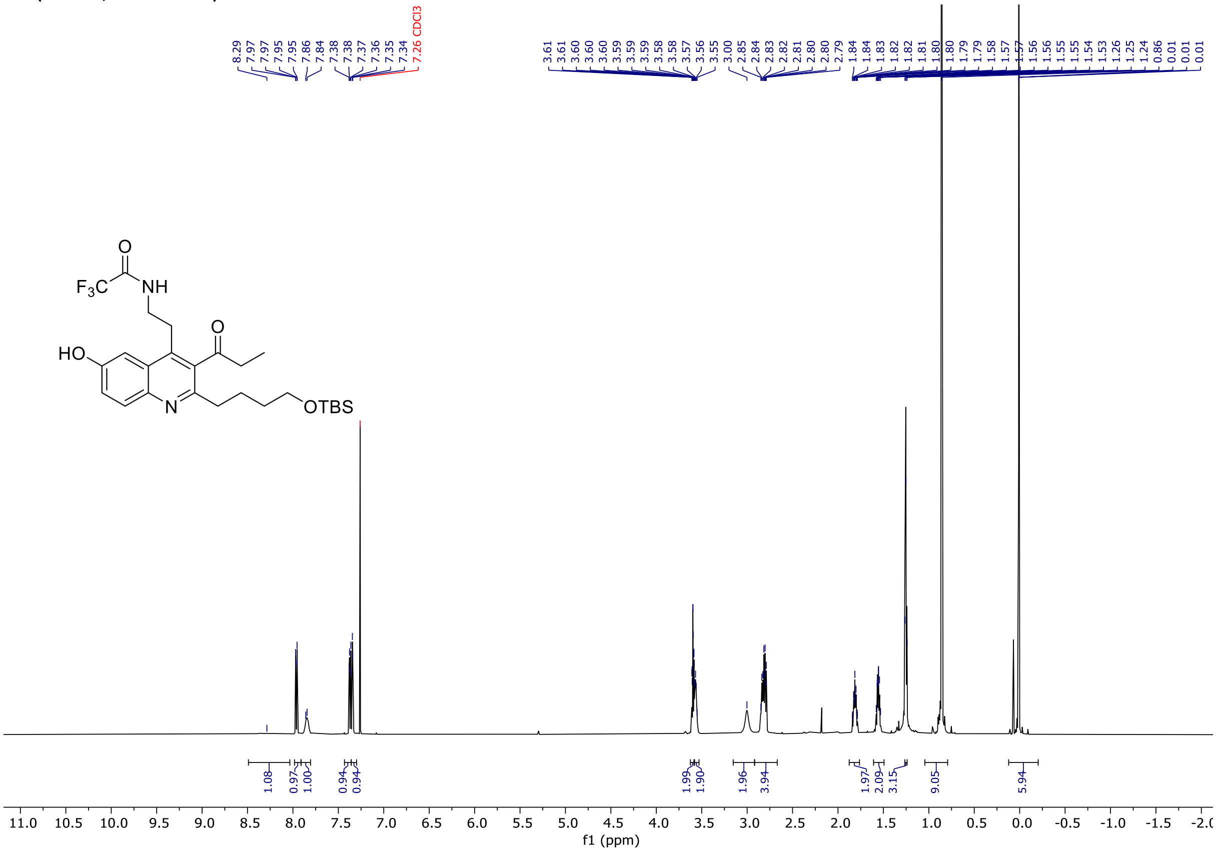

^{13}C ($CDCl_3$, 151 MHz)

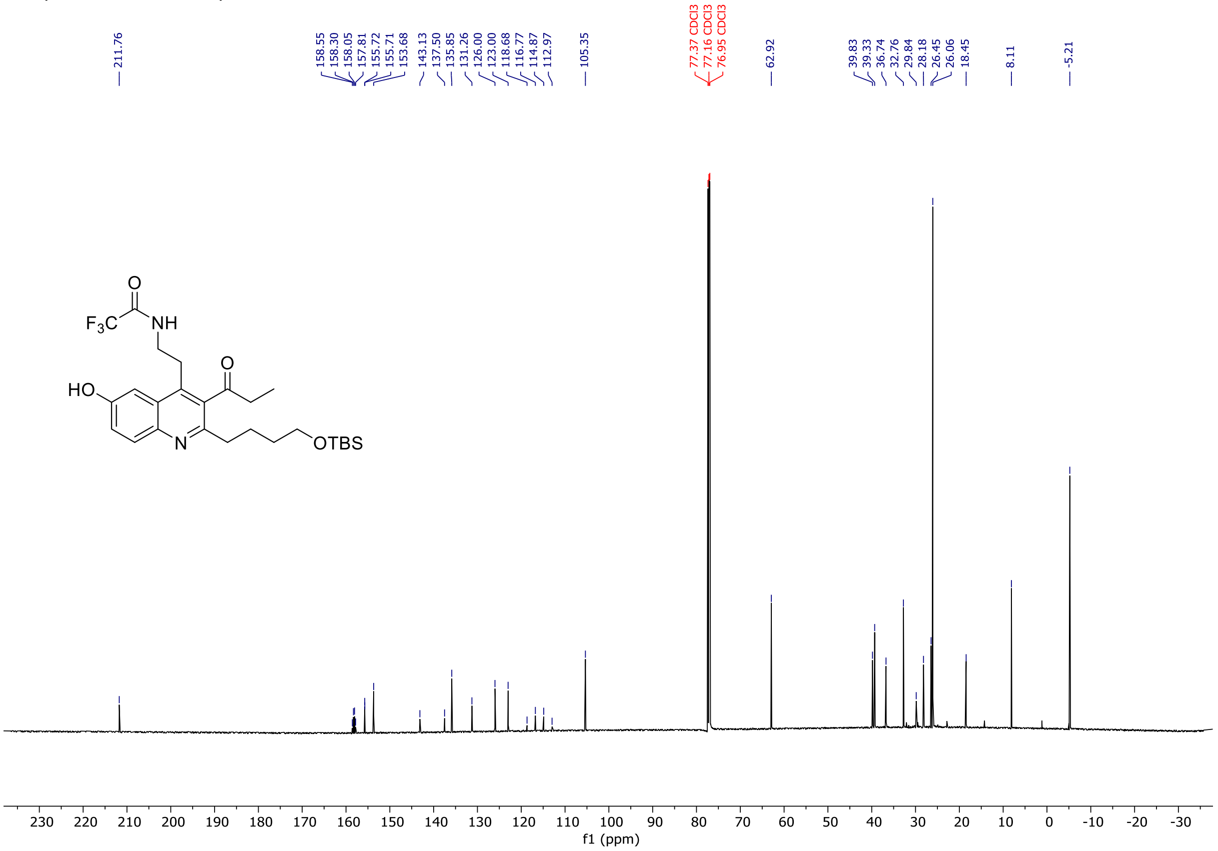

^{19}F (CDCl$_3$, 565 MHz)

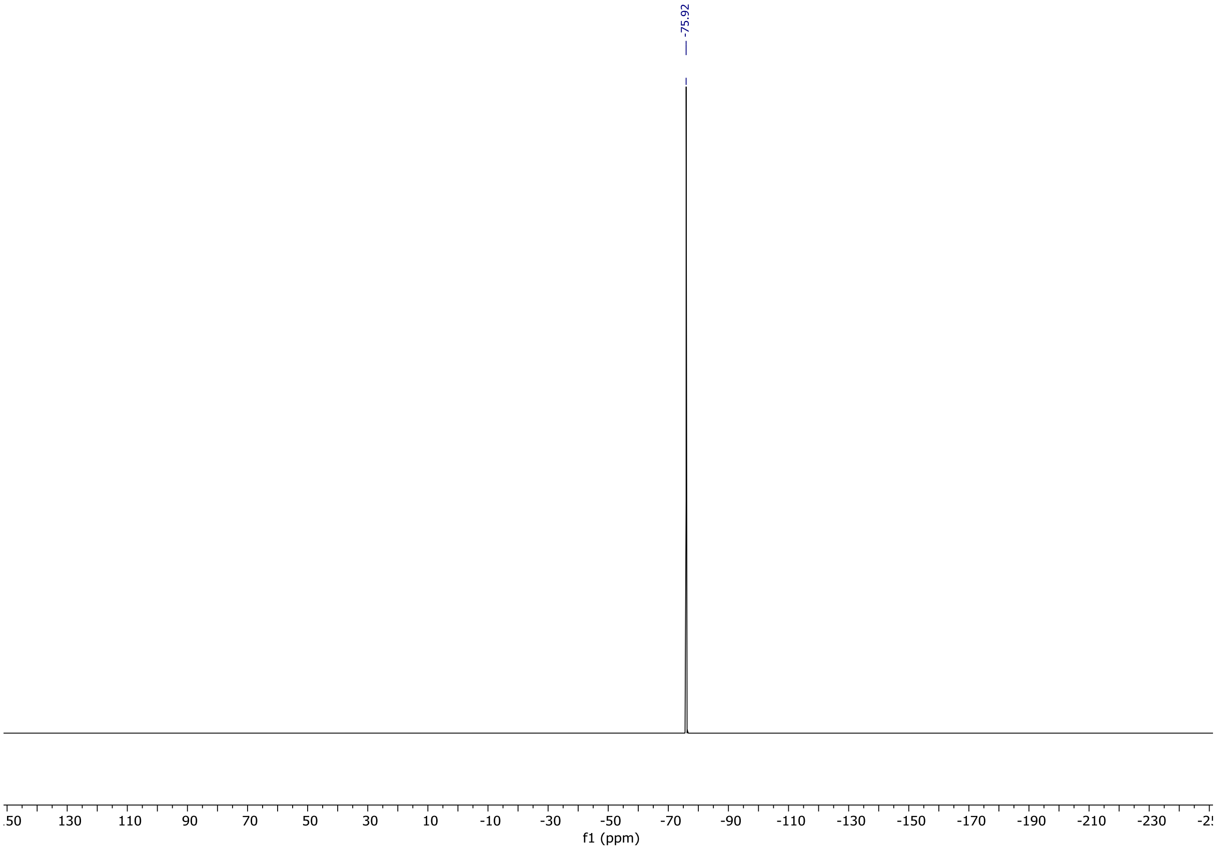

Compound 26

^{1}H ($CDCl_3$, 400 MHz)

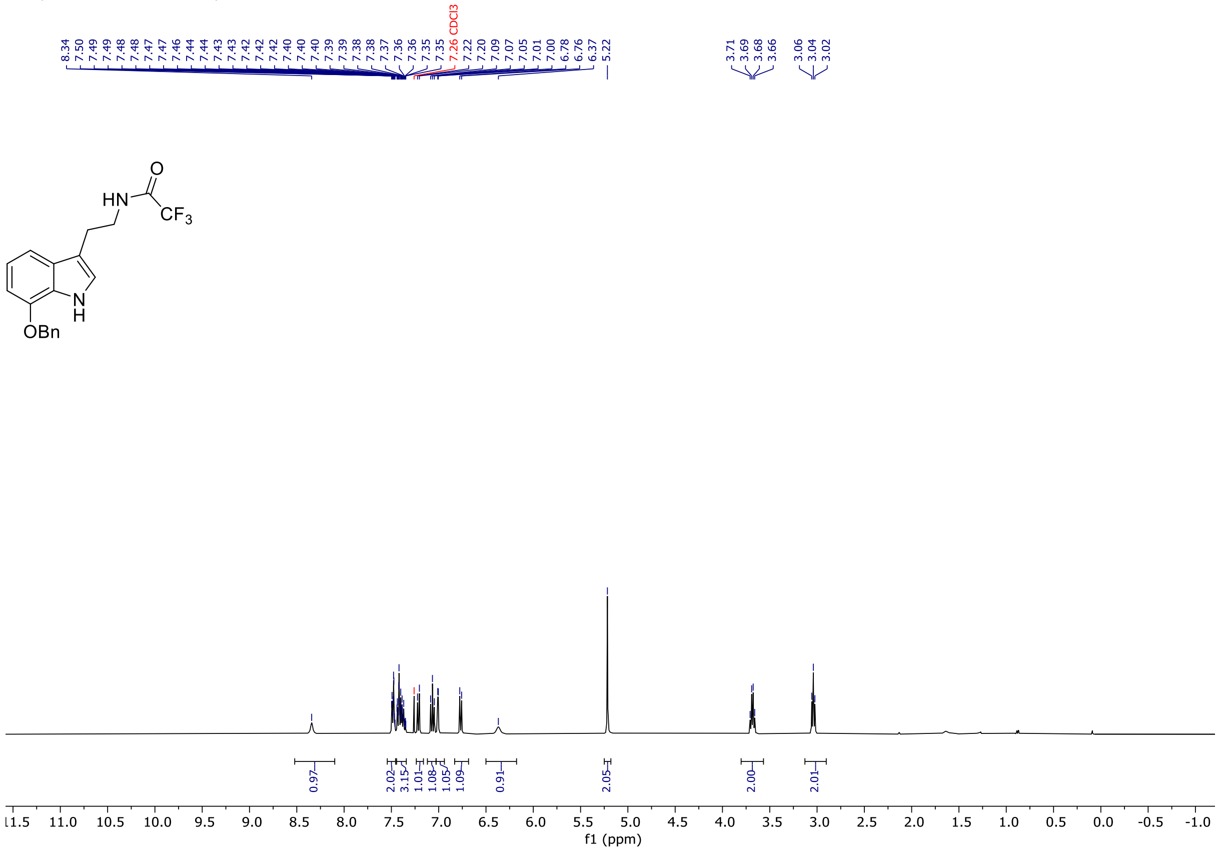

^{13}C ($CDCl_3$, 101 MHz)

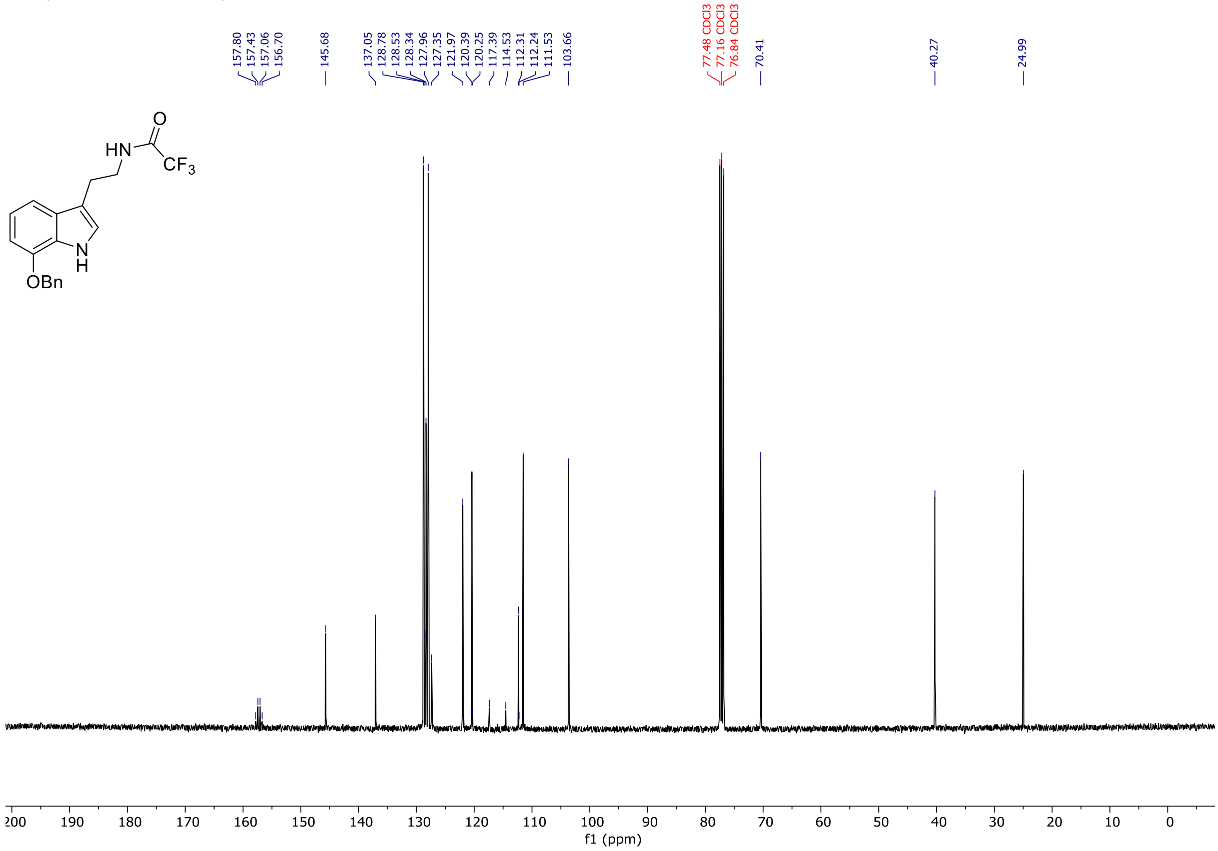

^{19}F NMR ($CDCl_3$, 565 MHz)

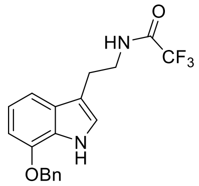

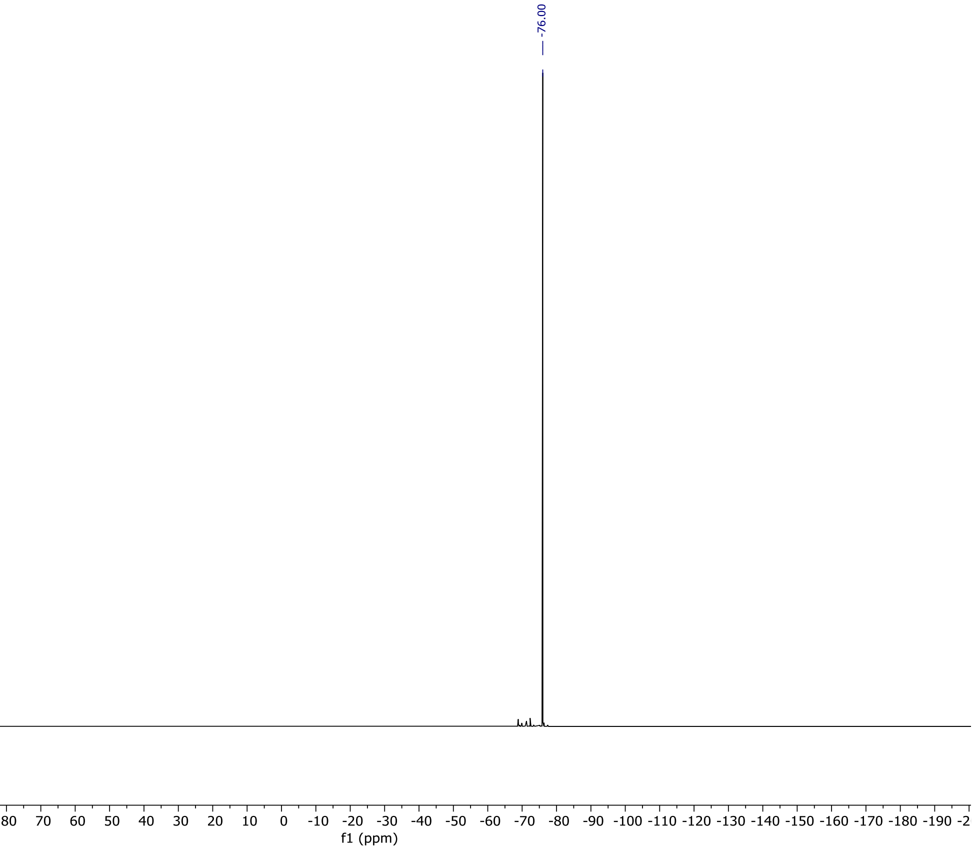

Compound 27

^{1}H ($CDCl_3$, 600 MHz)

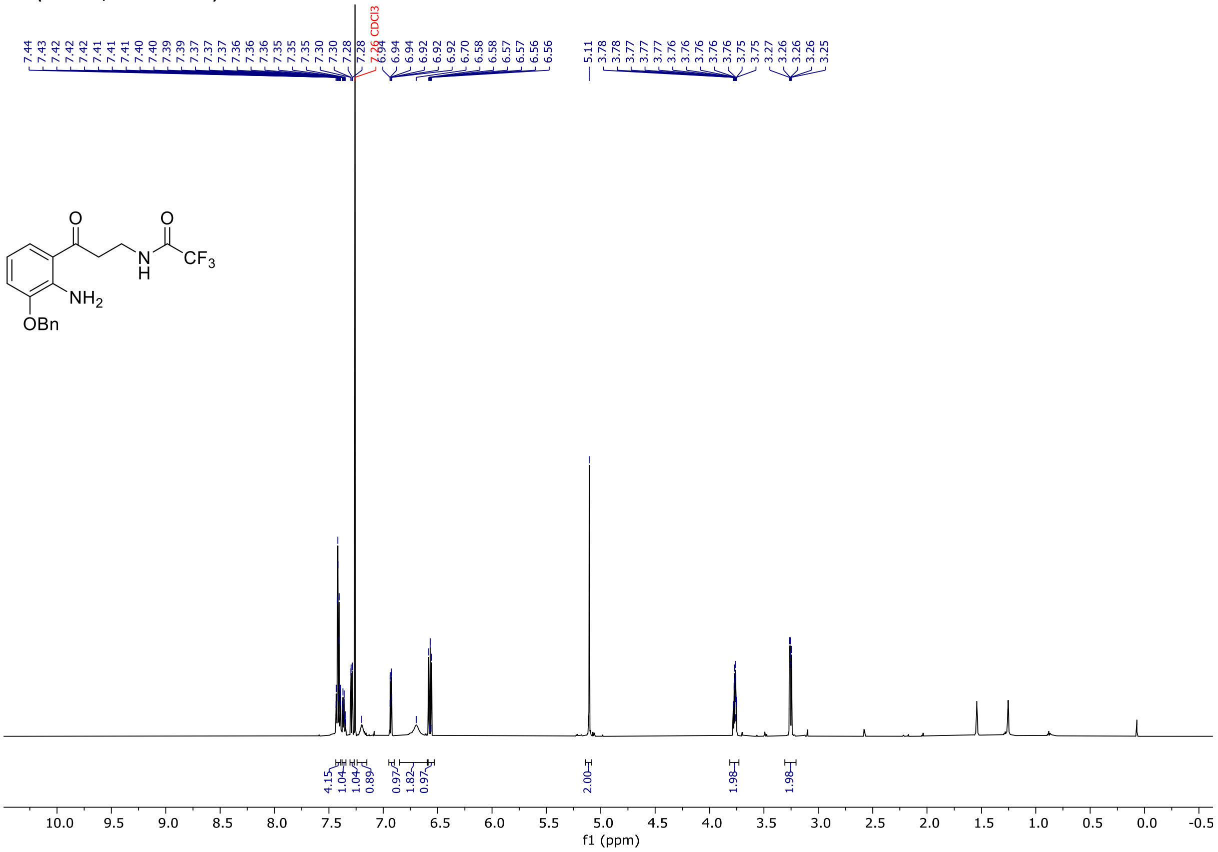

^{13}C ($CDCl_3$, 151 MHz)

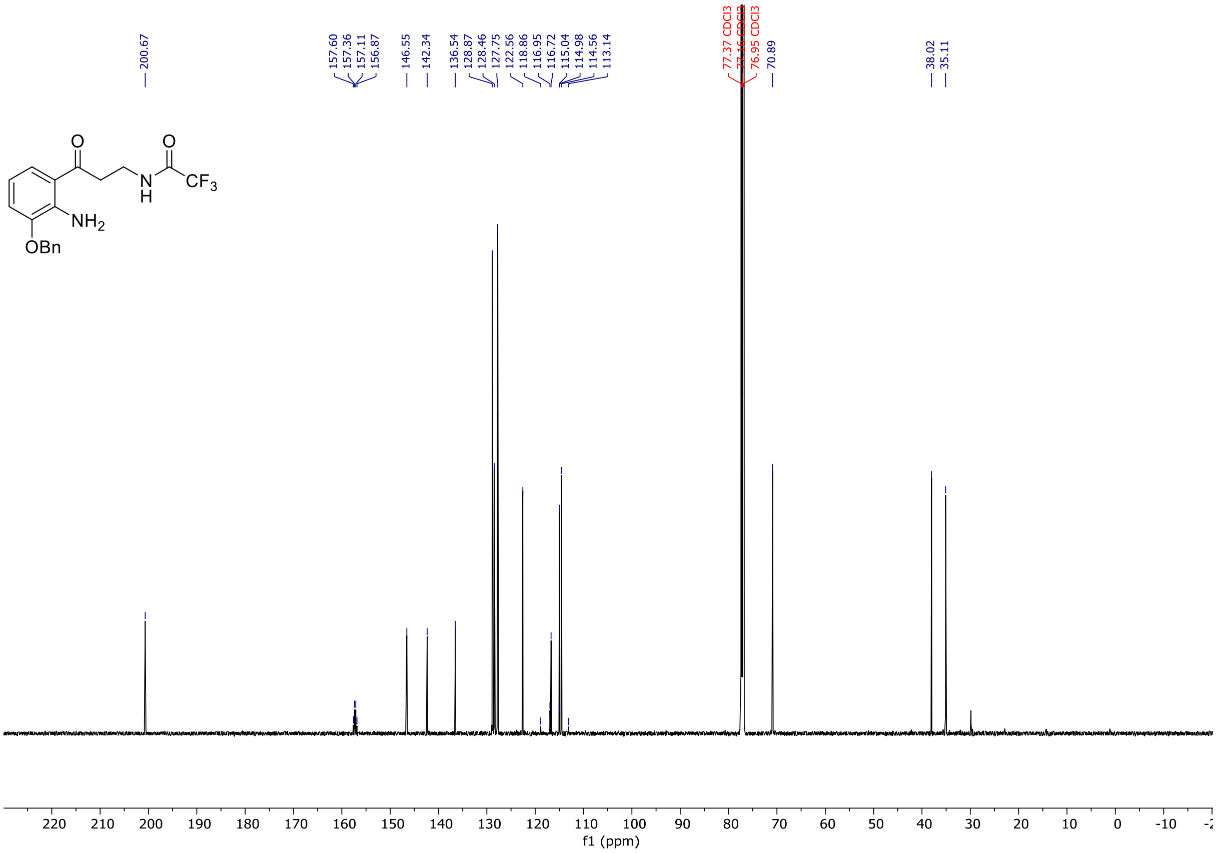

^{19}F NMR ($CDCl_3$, 565 MHz)

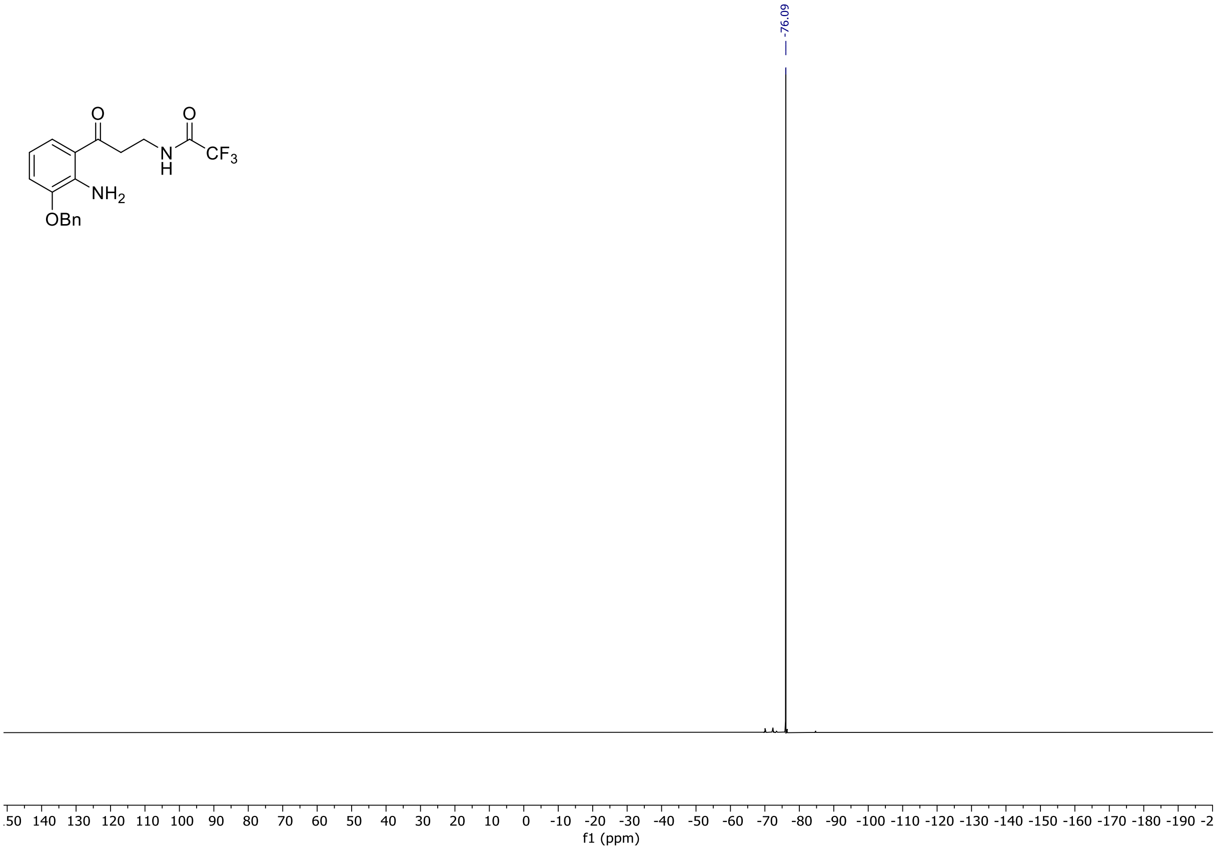

Compound 28

^{1}H ($CDCl_3$, 400 MHz)

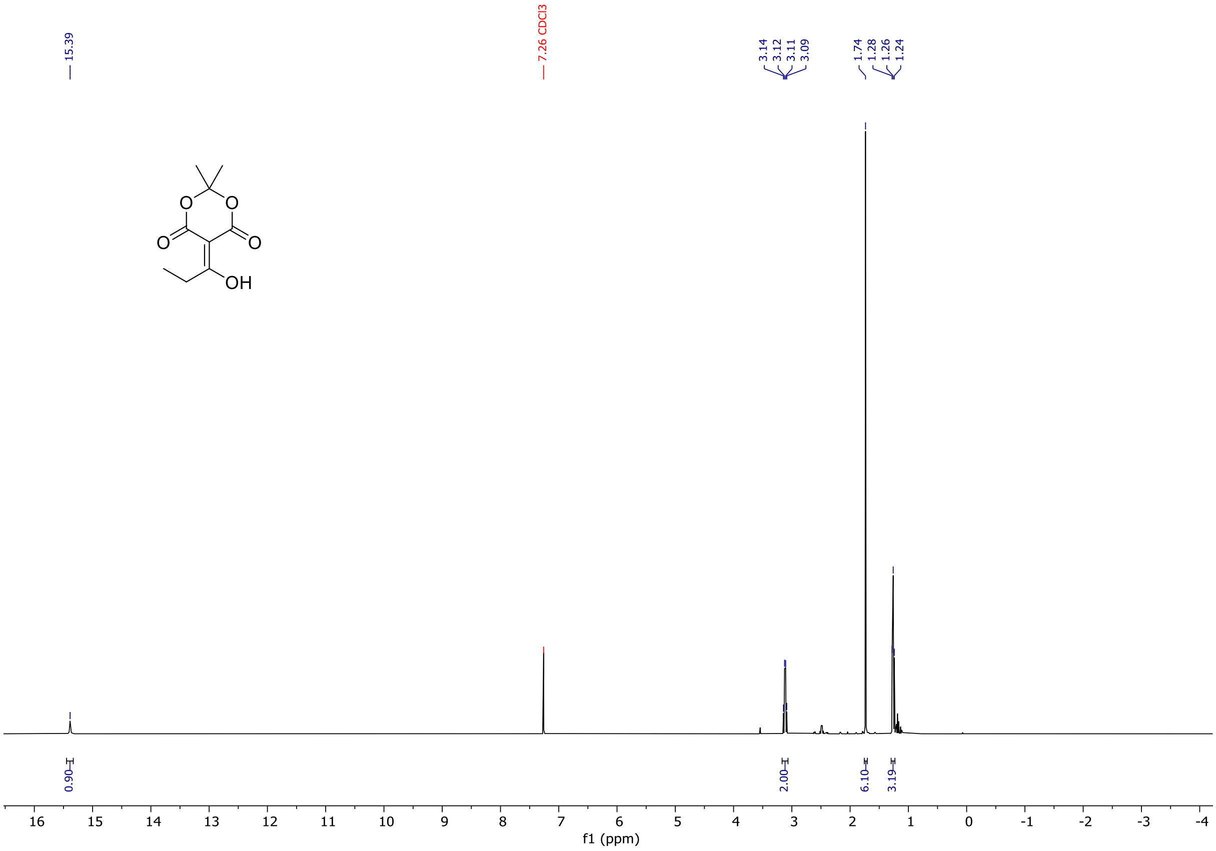

Compound 29

^{1}H (MeOD, 600 MHz)

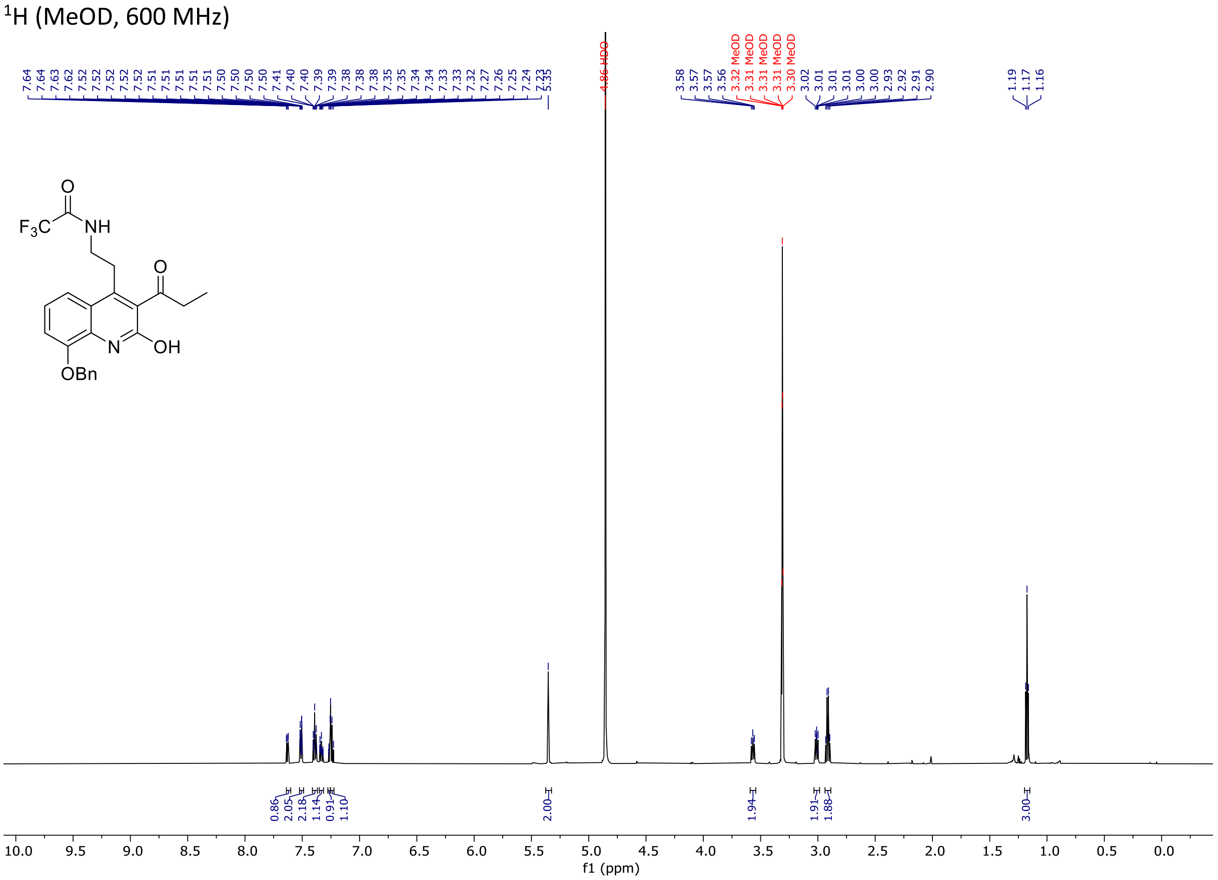

^{13}C (MeOD, 151 MHz)

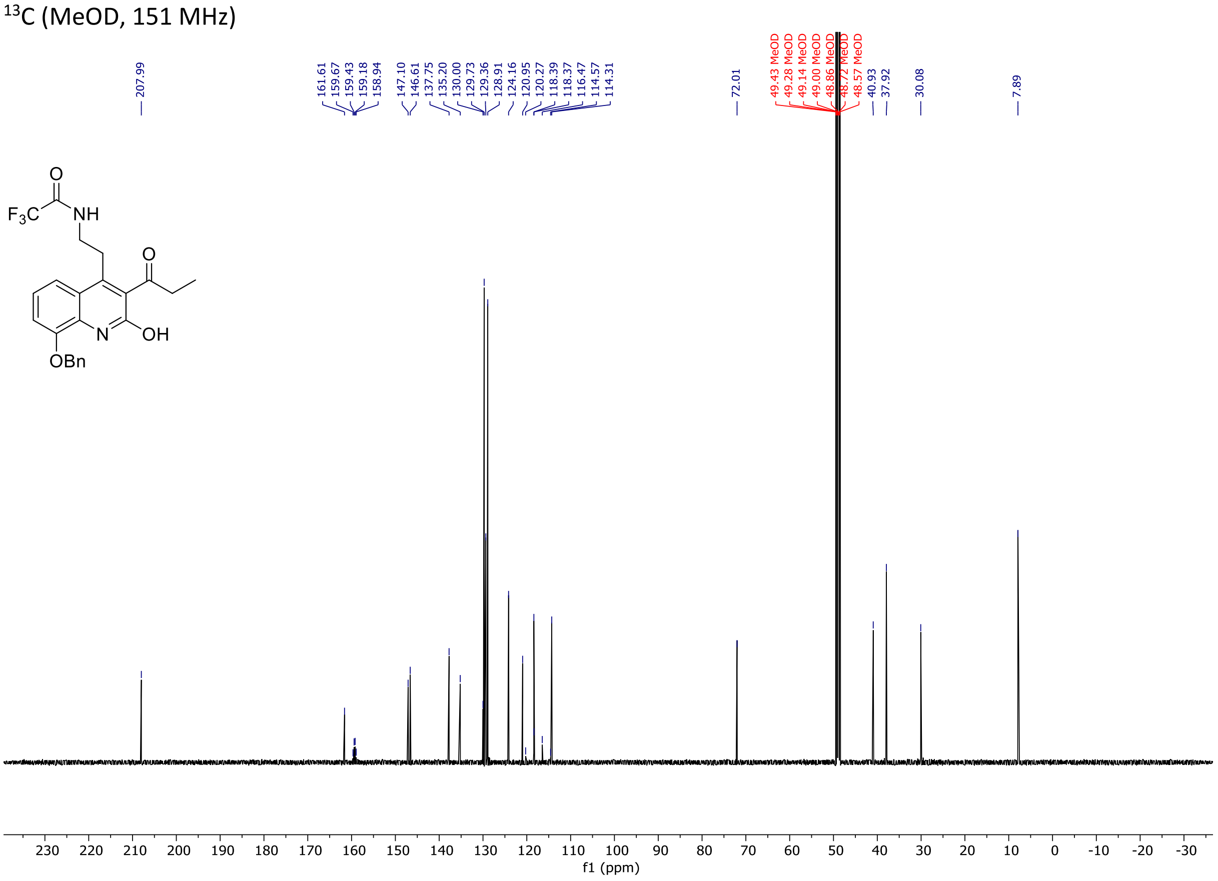

^{19}F NMR (MeOD, 565 MHz)

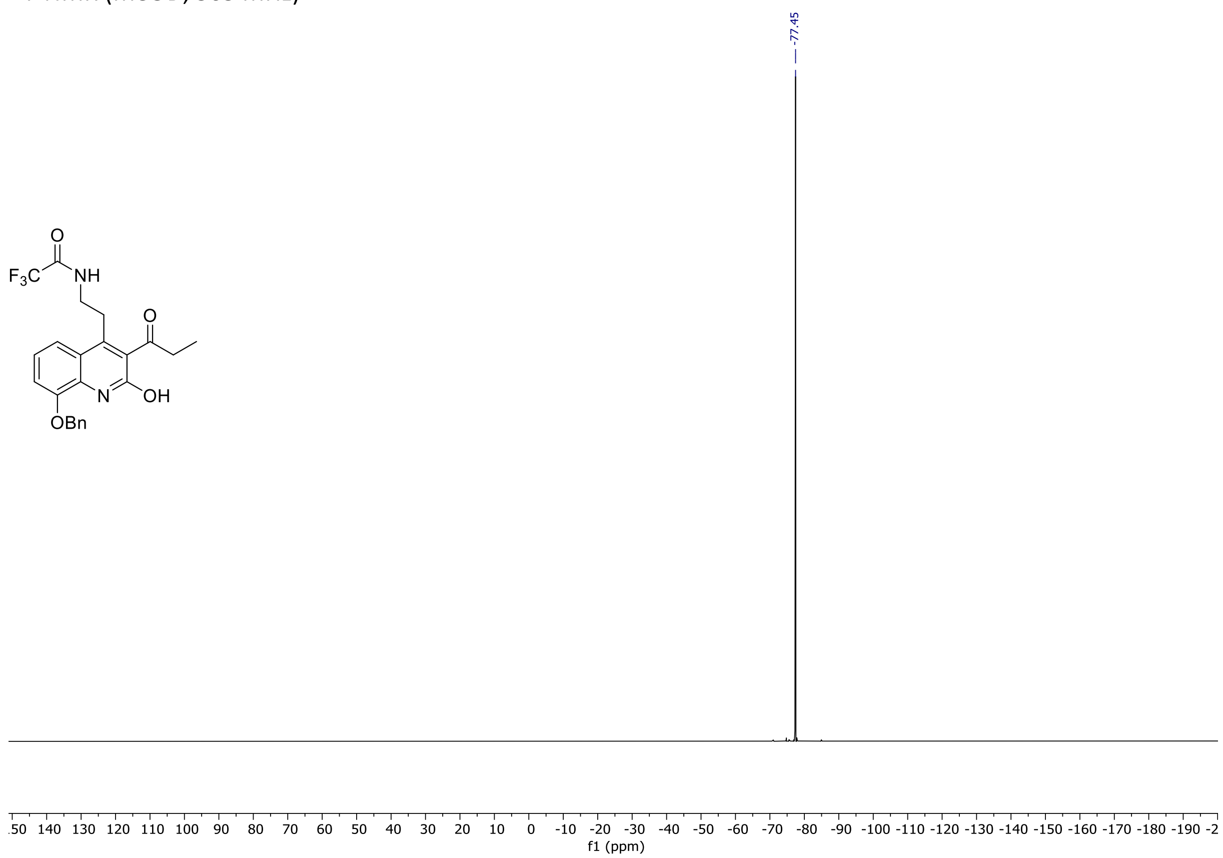

Compound 30

^{1}H ($CDCl_3$, 600 MHz)

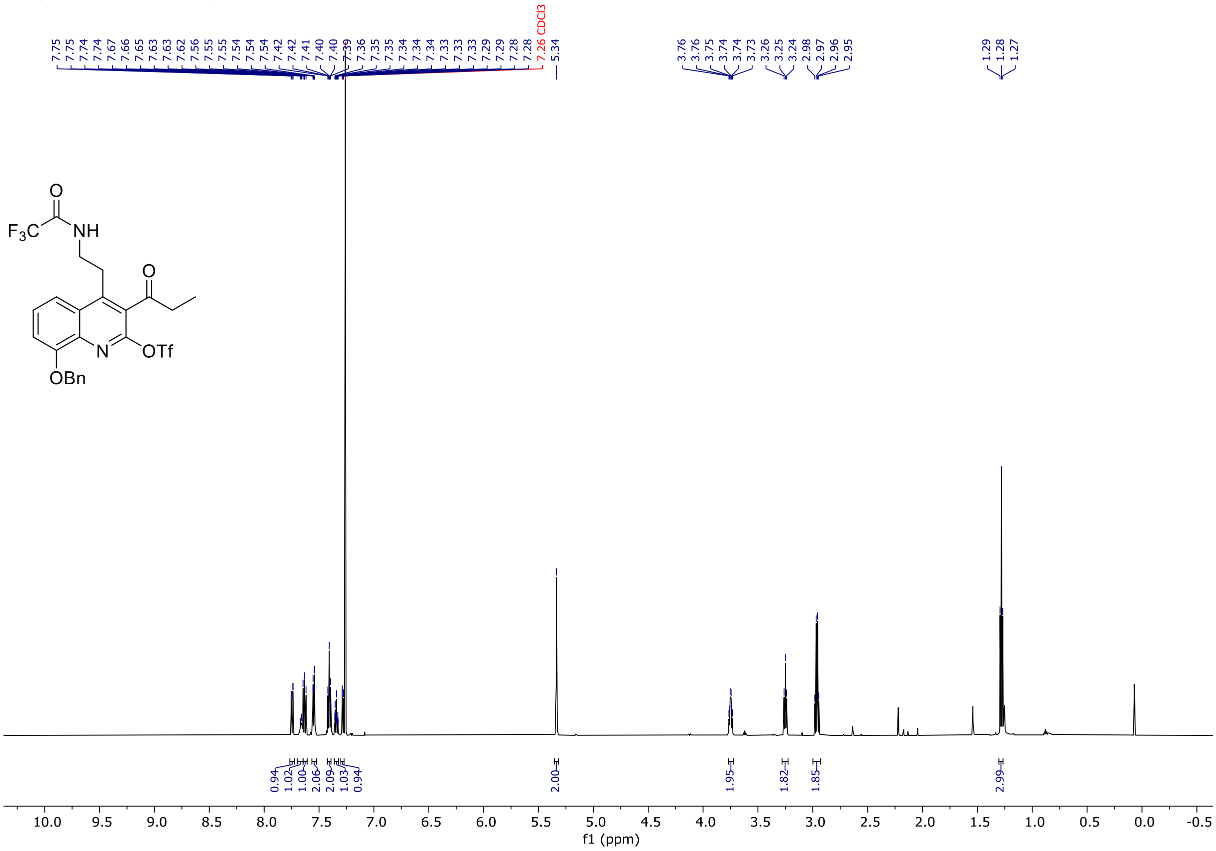

^{13}C ($CDCl_3$, 151 MHz)

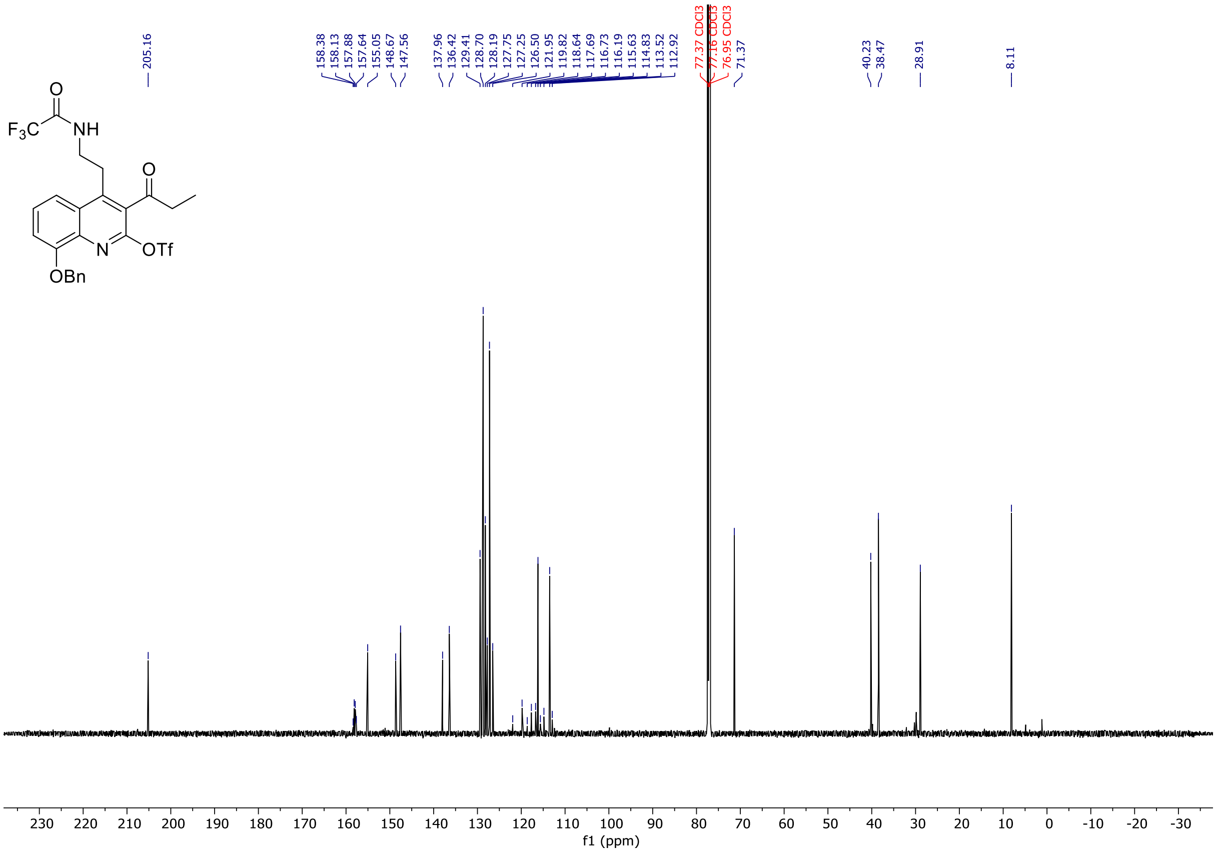

^{19}F NMR ($CDCl_3$, 565 MHz)

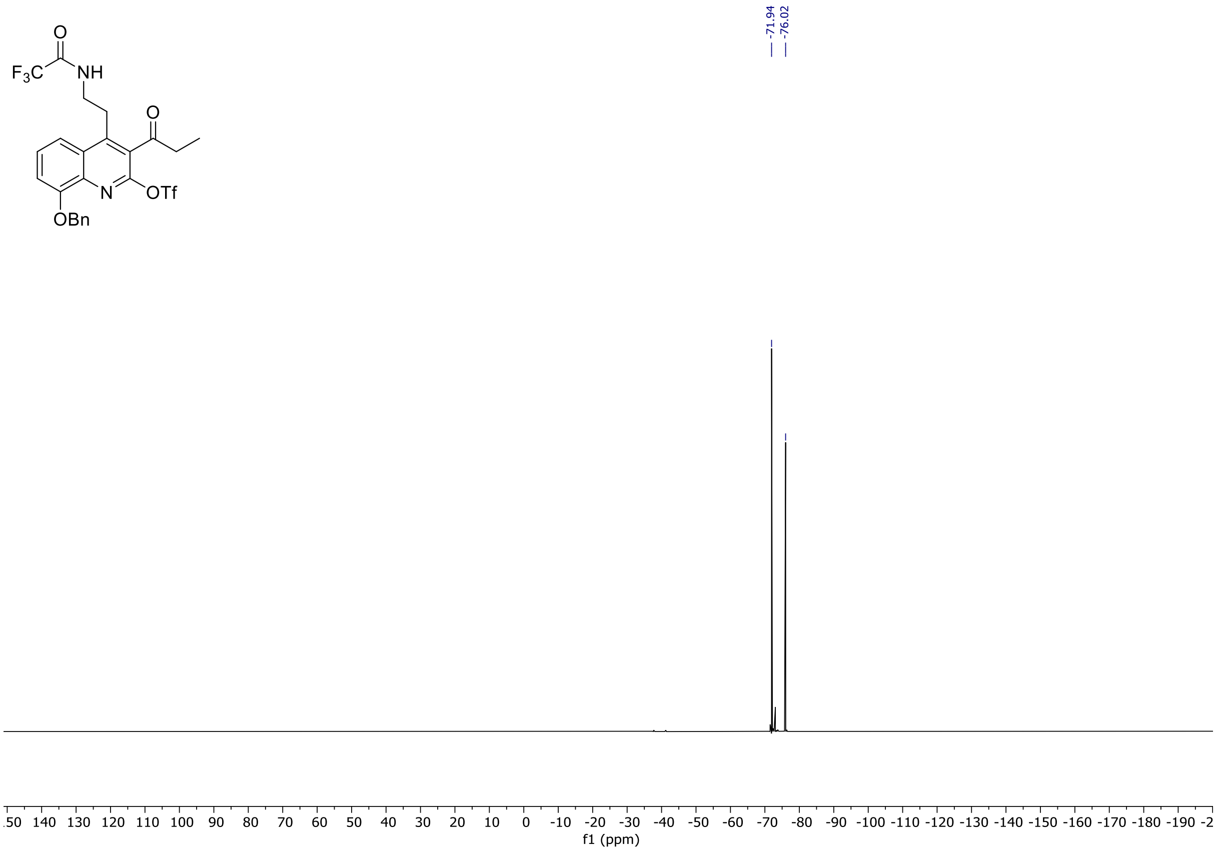

Compound 31

^{1}H ($CDCl_3$, 400 MHz)

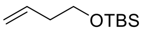

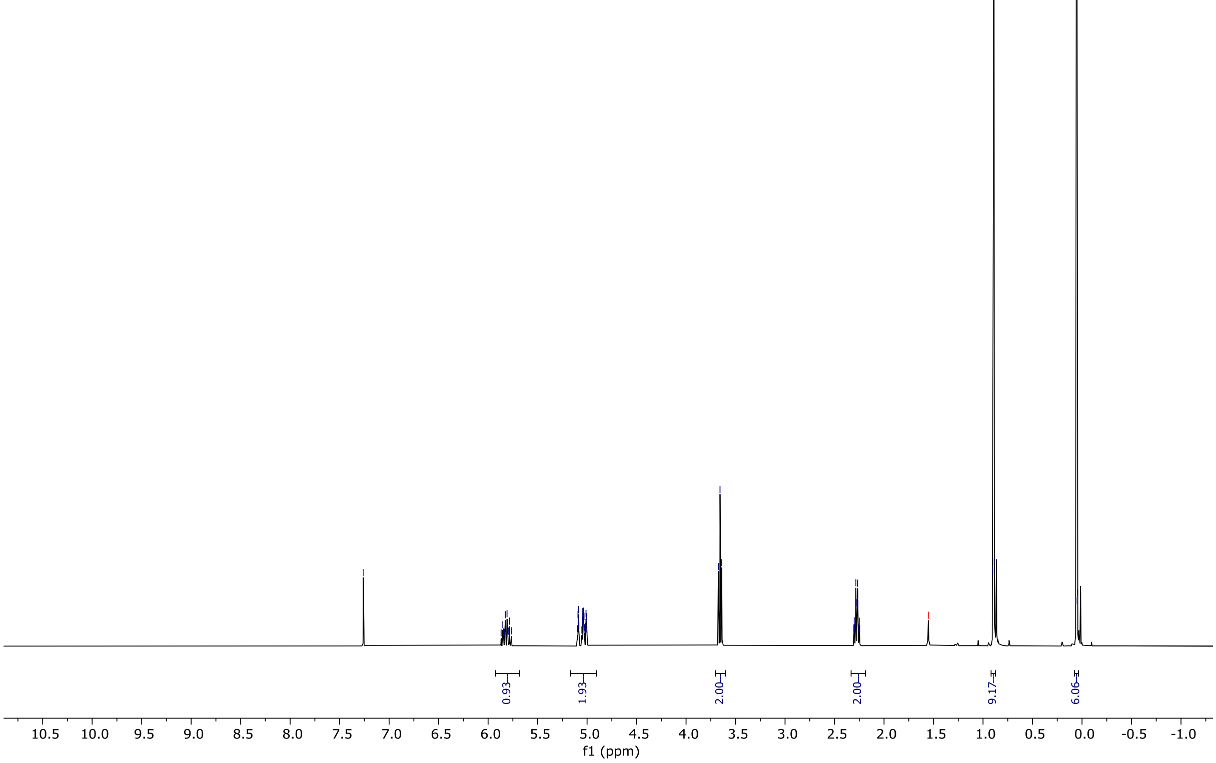

Compound 32

^{1}H ($CDCl_3$, 600 MHz)

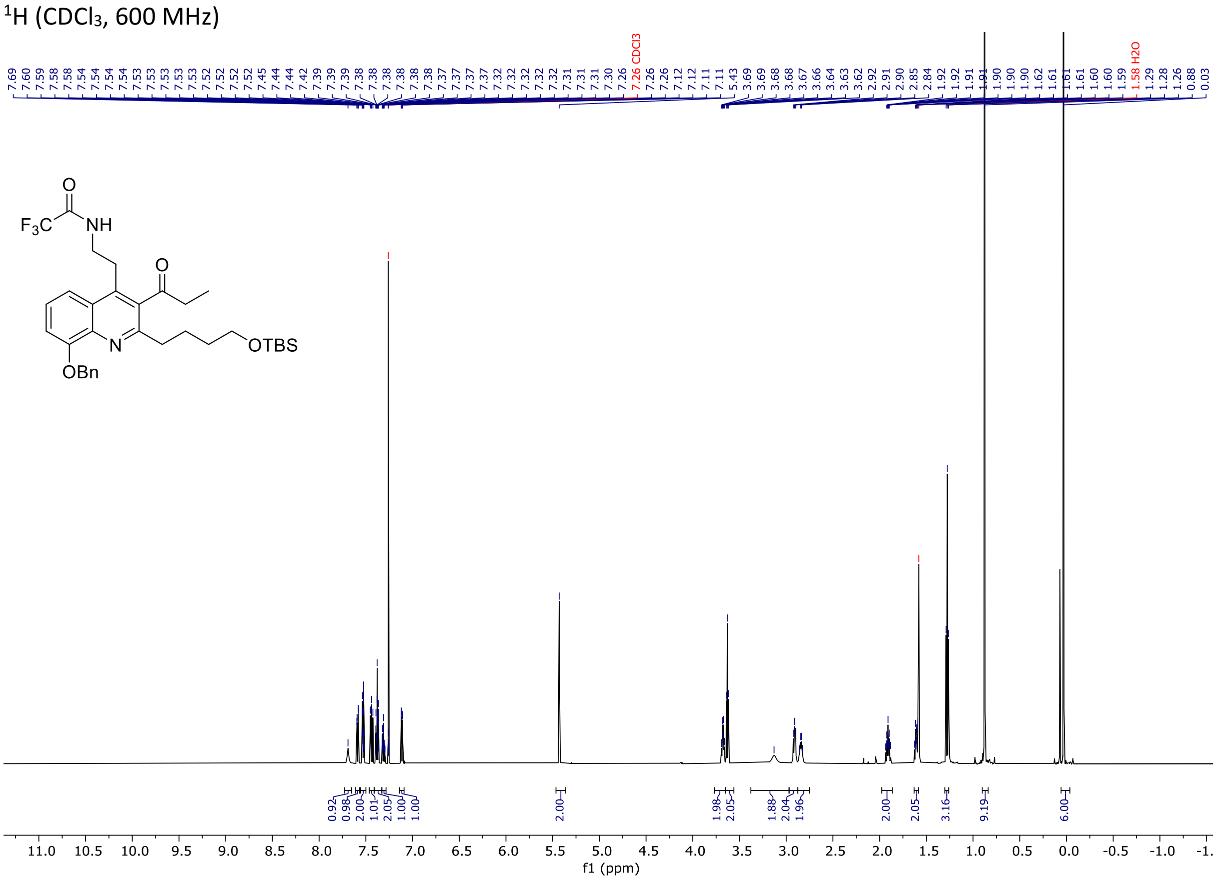

^{13}C ($CDCl_3$, 151 MHz)

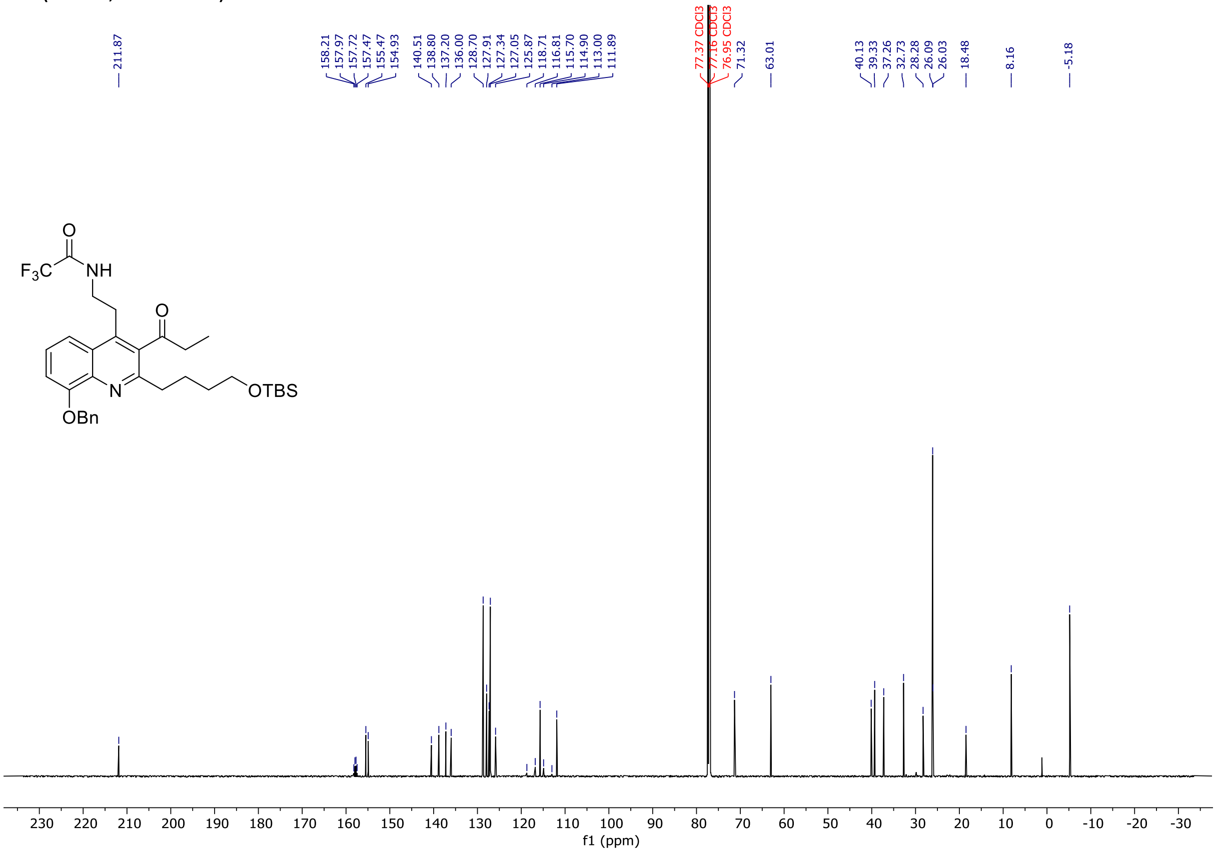

^{19}F (CDCl$_3$, 565 MHz)

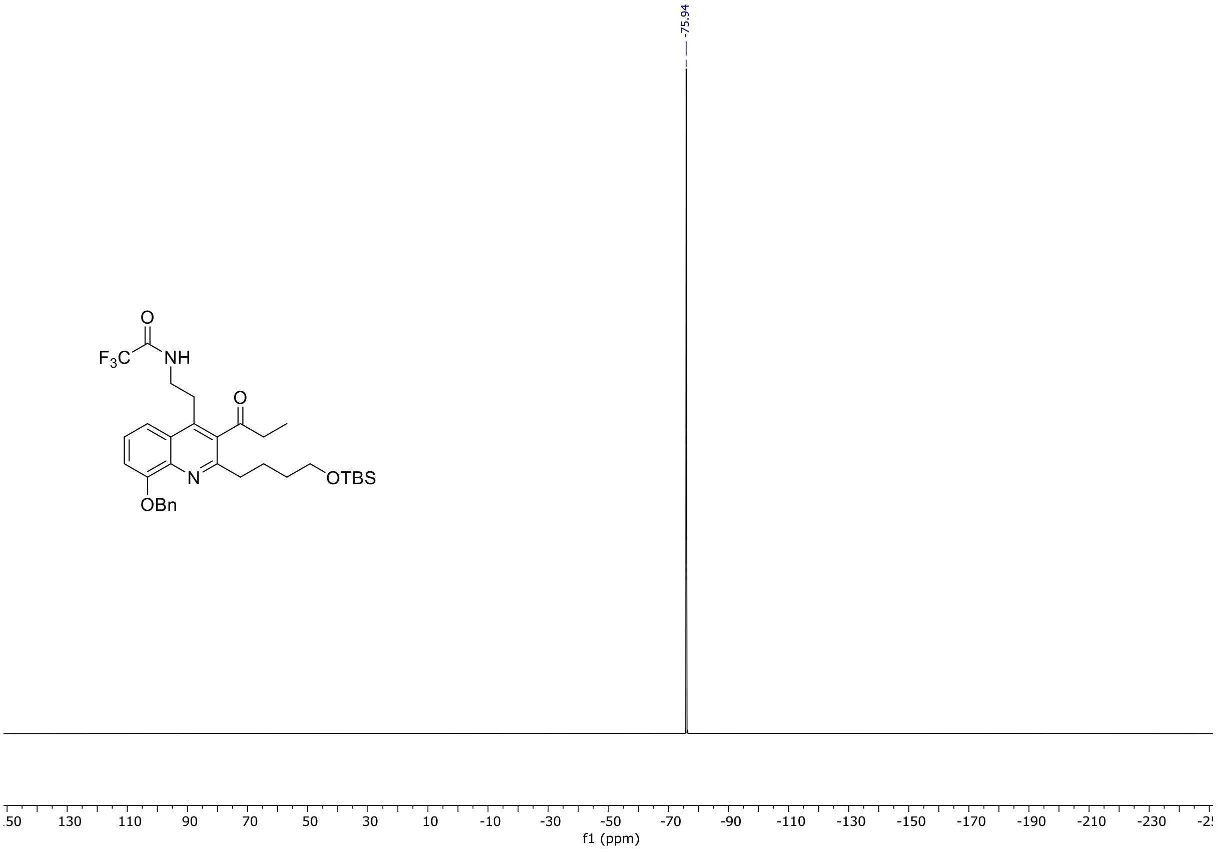

Compound S17

^{1}H ($CDCl_3$, 400 MHz)

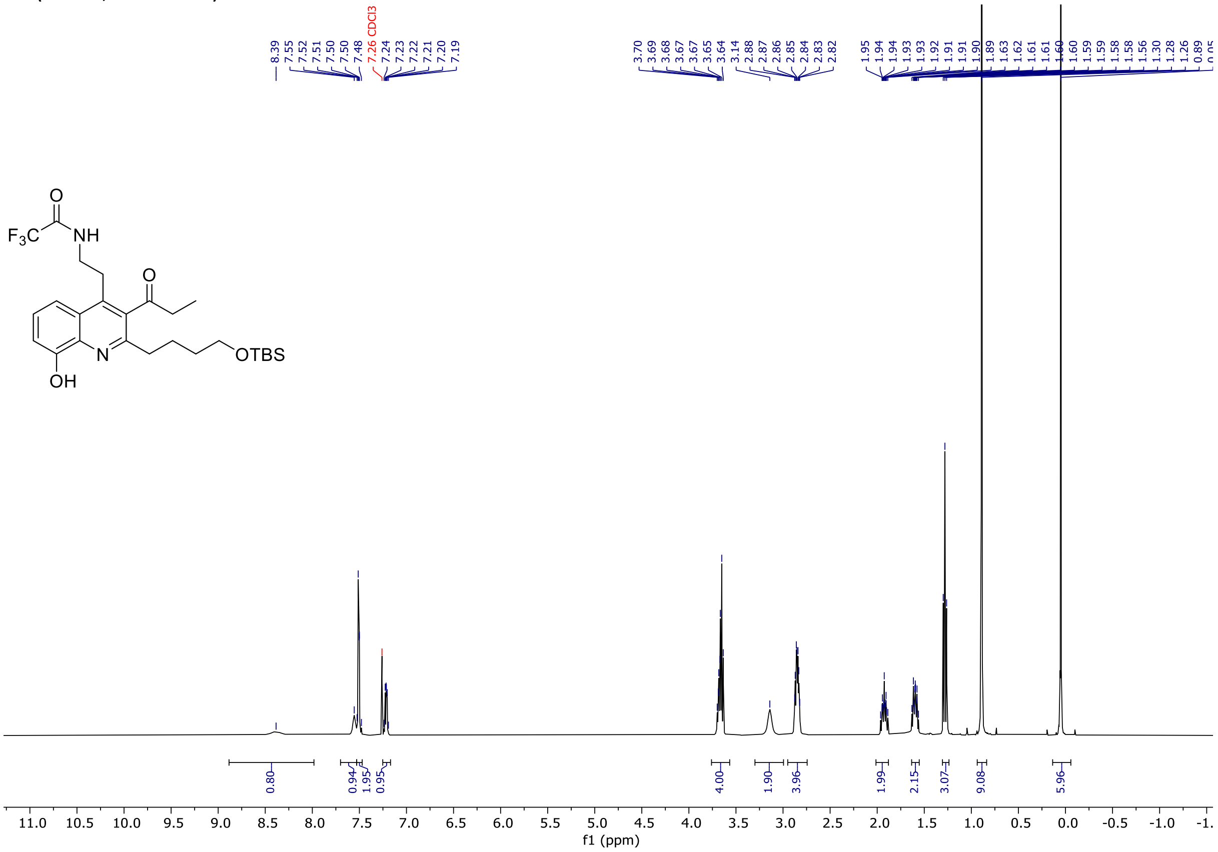

^{13}C ($CDCl_3$, 101 MHz)

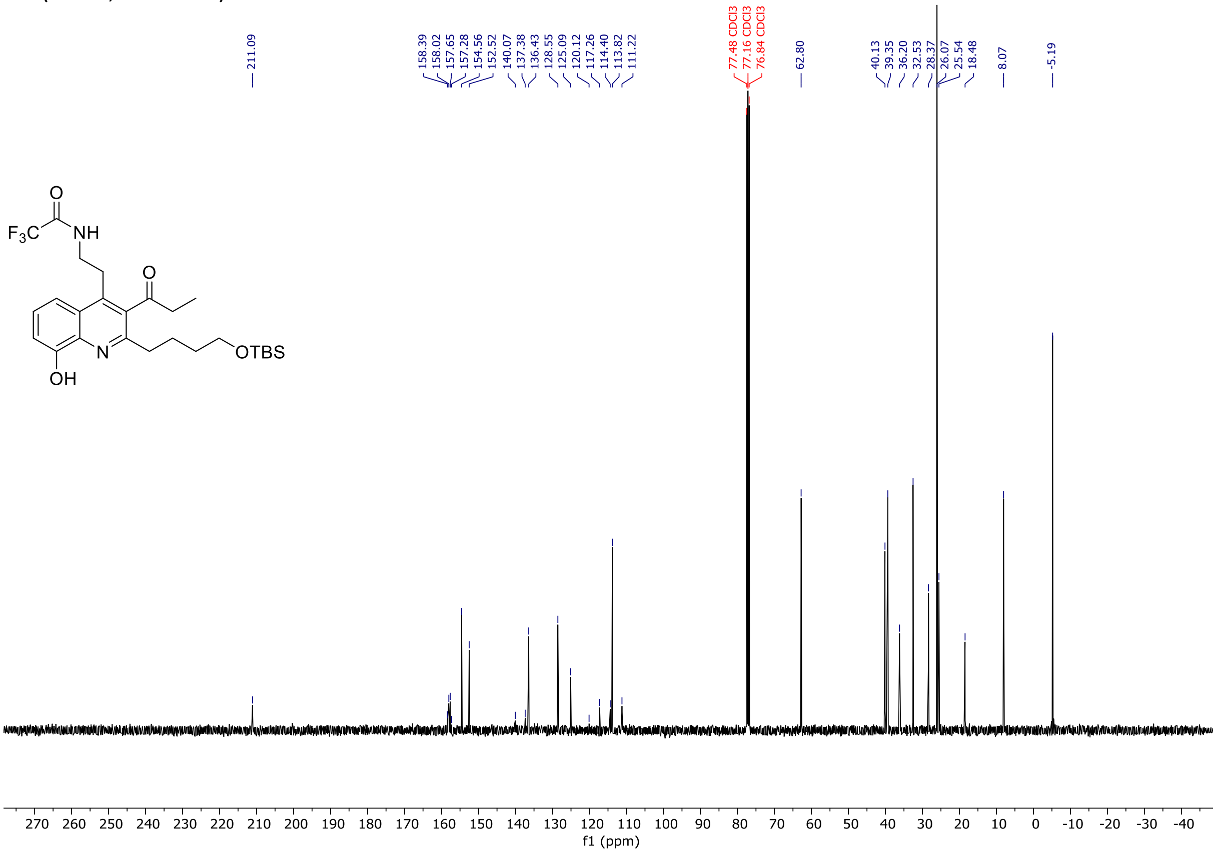

^{19}F ($CDCl_3$, 282 MHz)

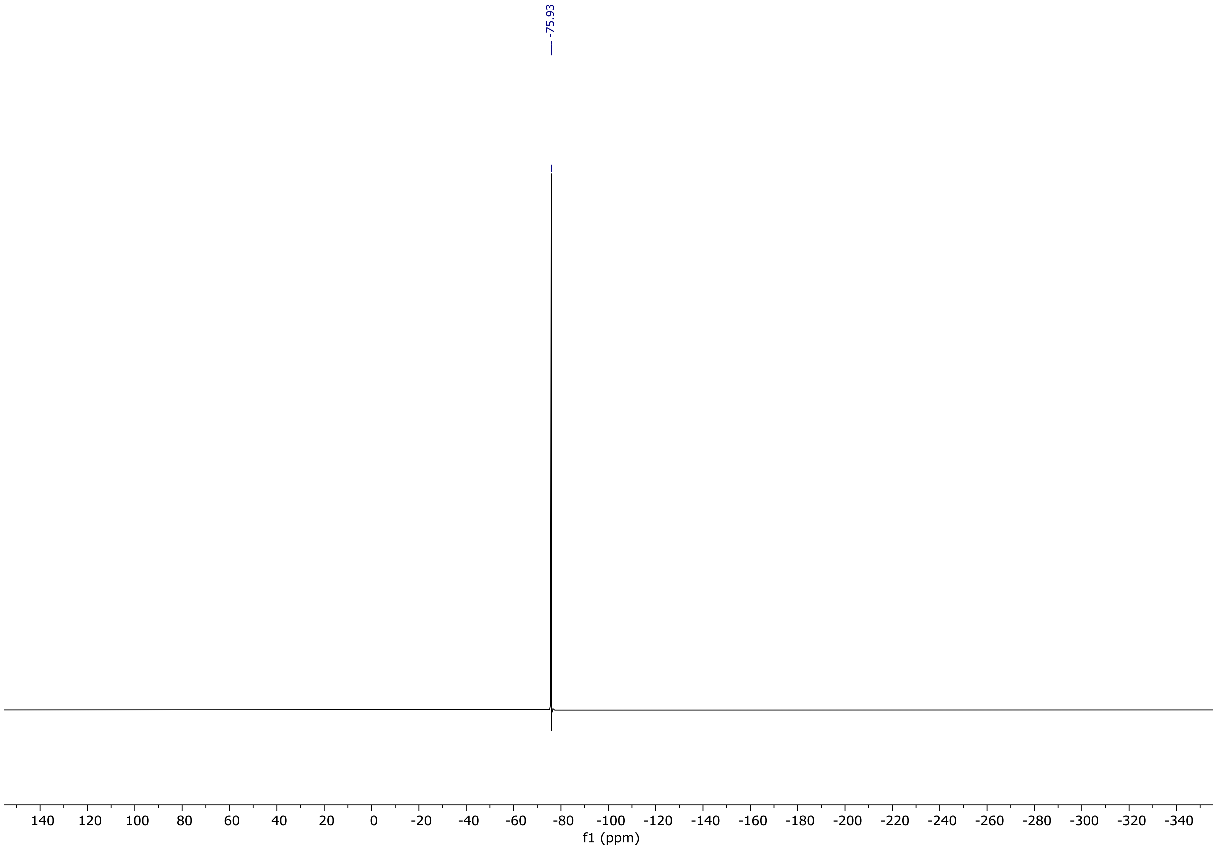

Compound S18

^{1}H ($CDCl_3$, 400 MHz)

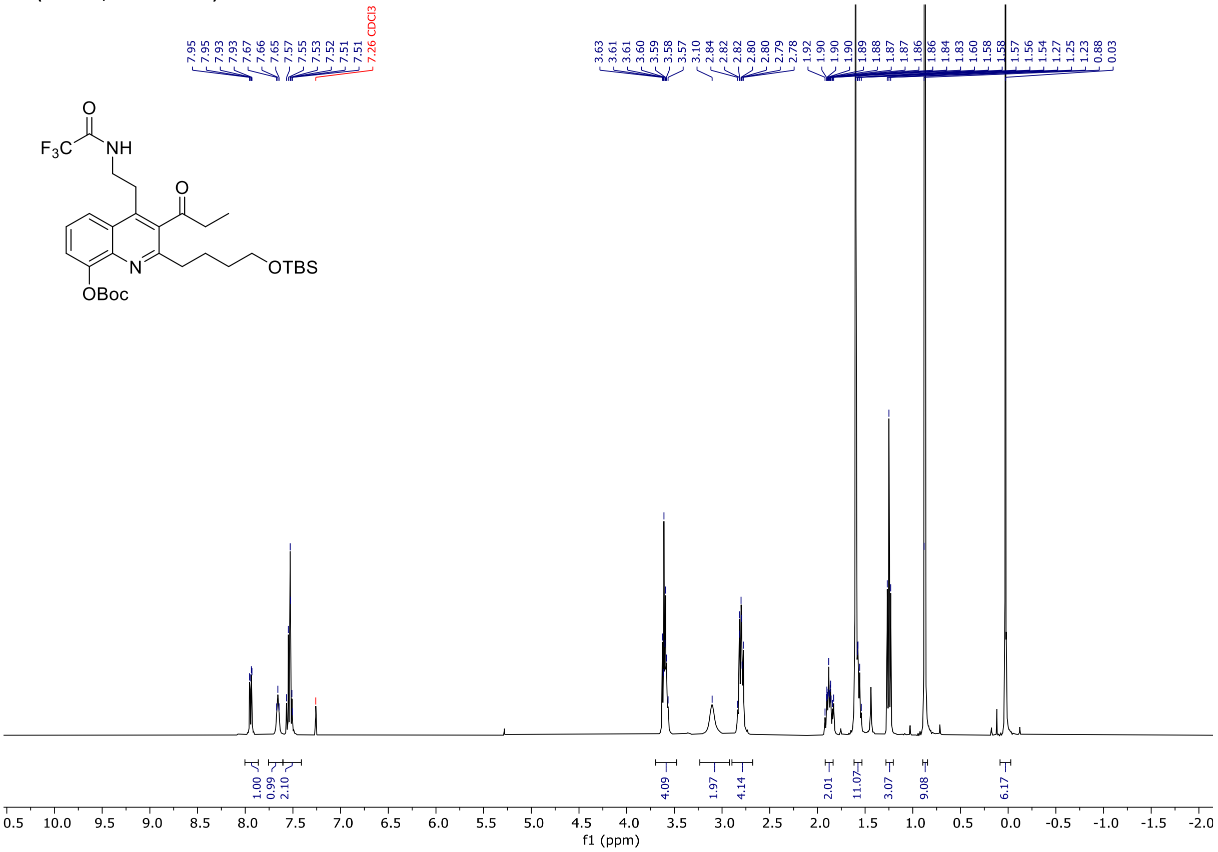

^{13}C ($CDCl_3$, 101 MHz)

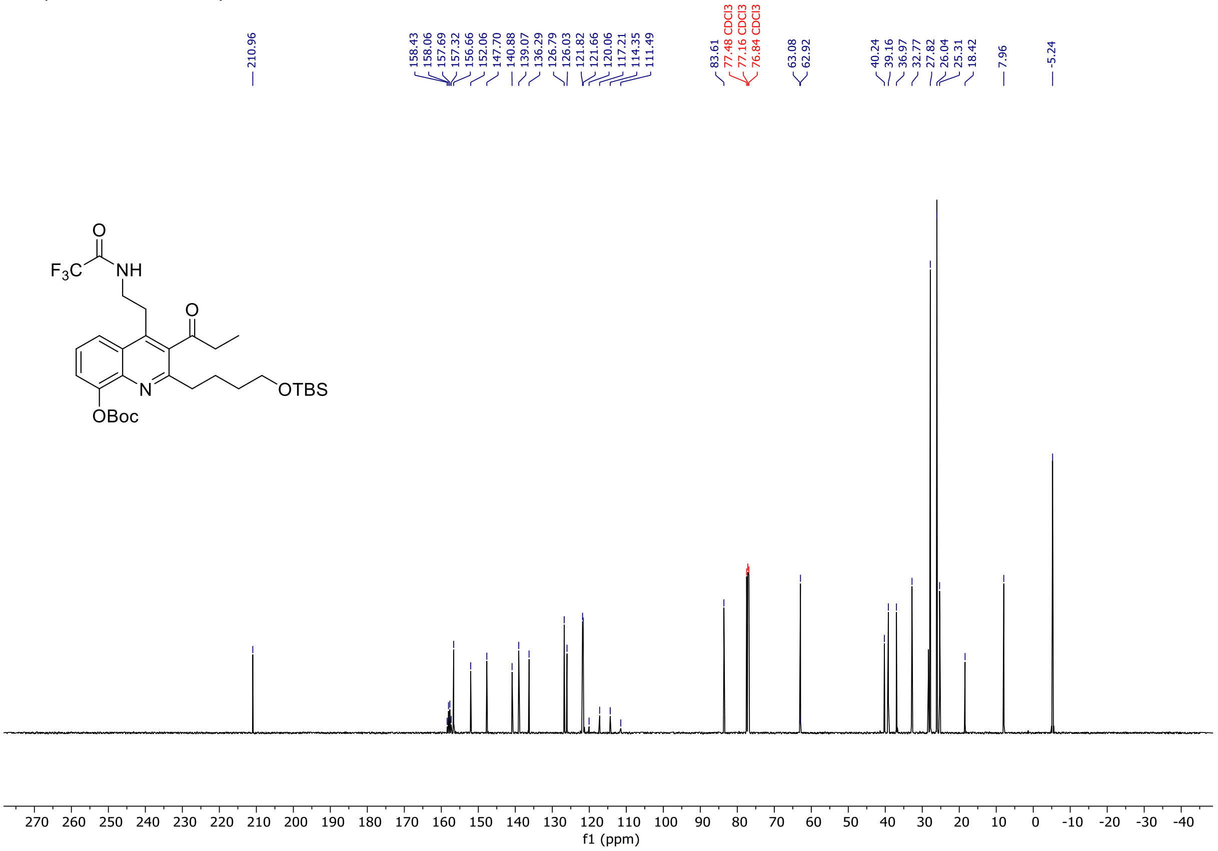

^{19}F ($CDCl_3$, 282 MHz)

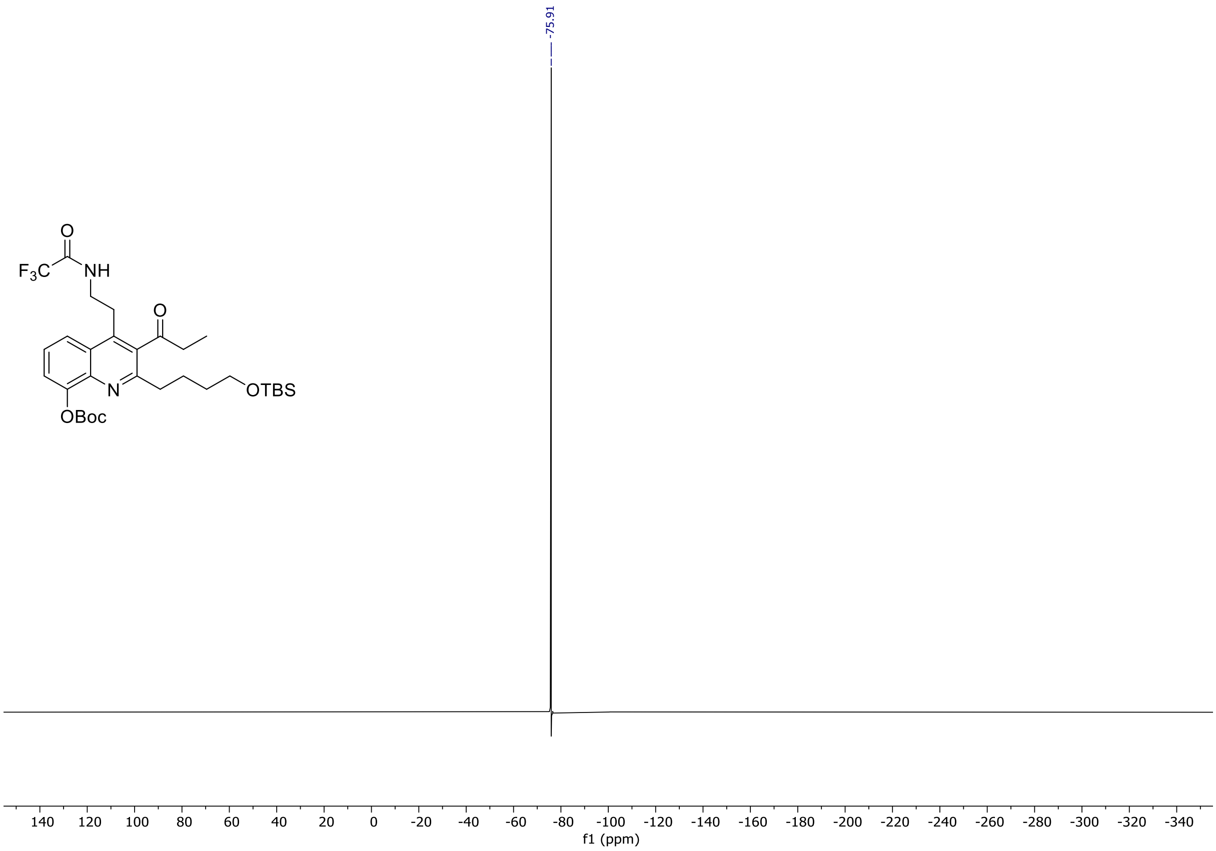

Compound 33

^{1}H ($CDCl_3$, 400 MHz)

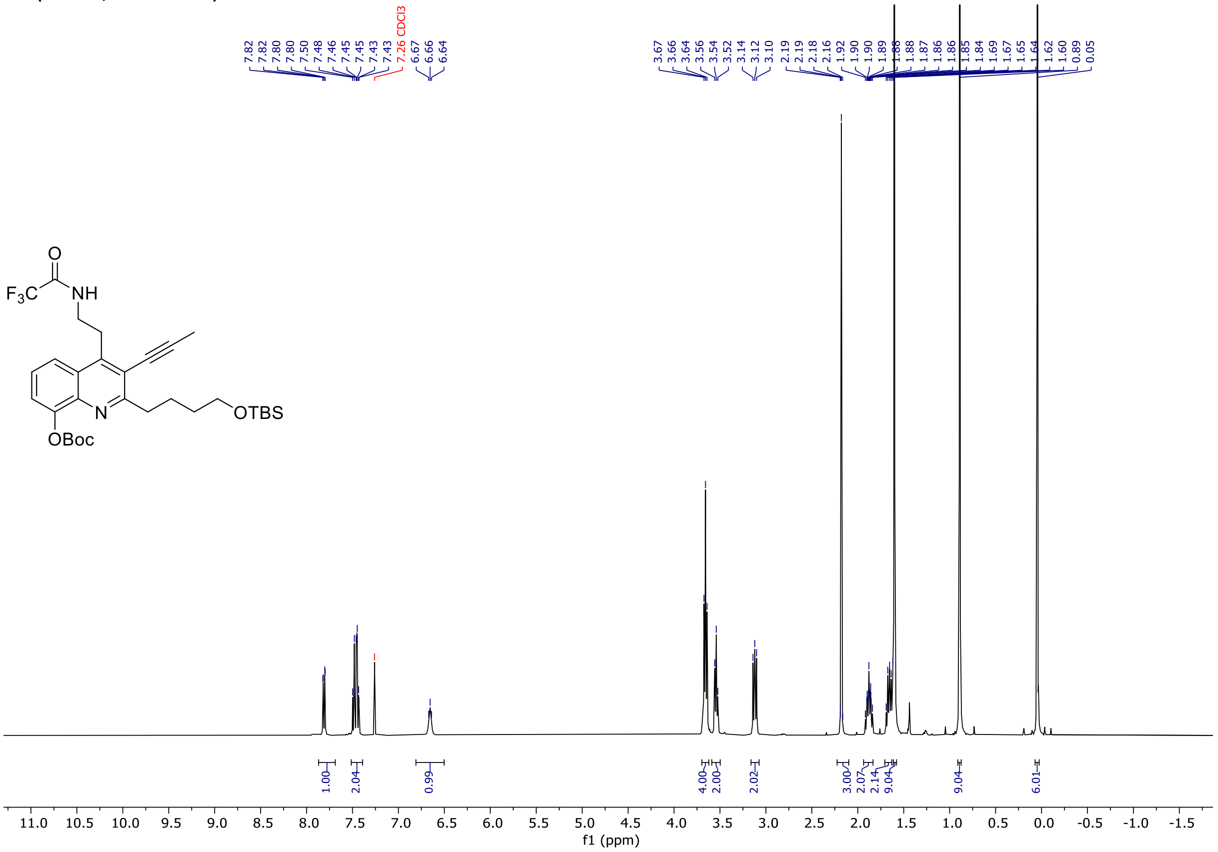

^{13}C ($CDCl_3$, 101 MHz)

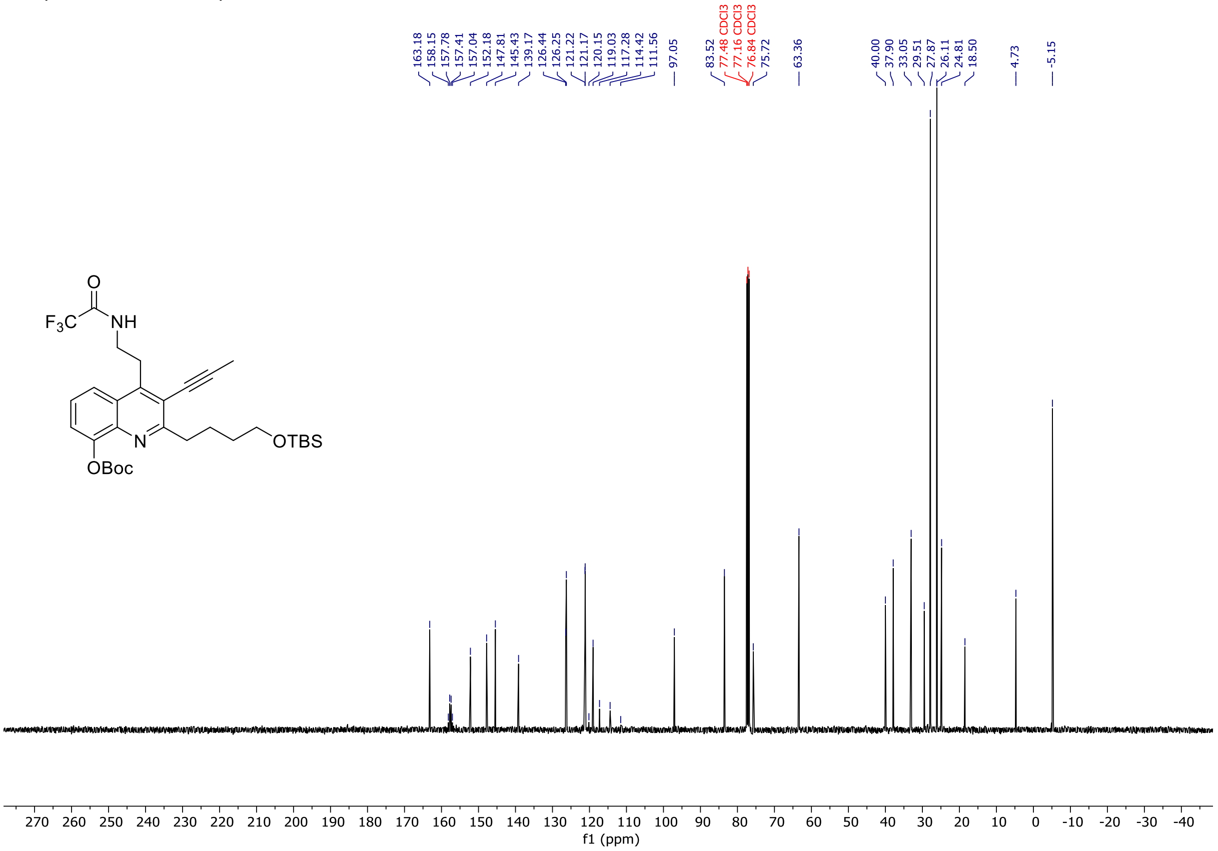

^{19}F ($CDCl_3$, 282 MHz)

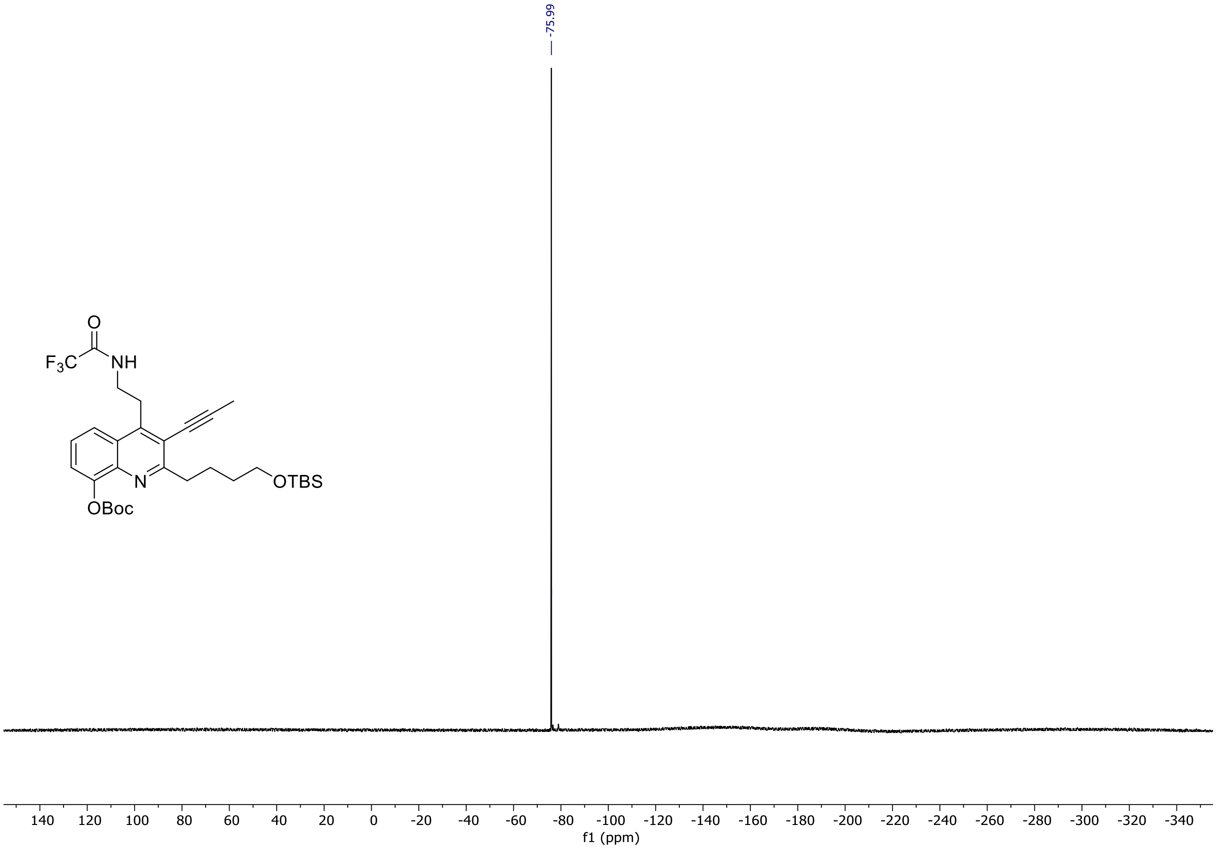

Compound S19

^{1}H ($CDCl_3$, 400 MHz)

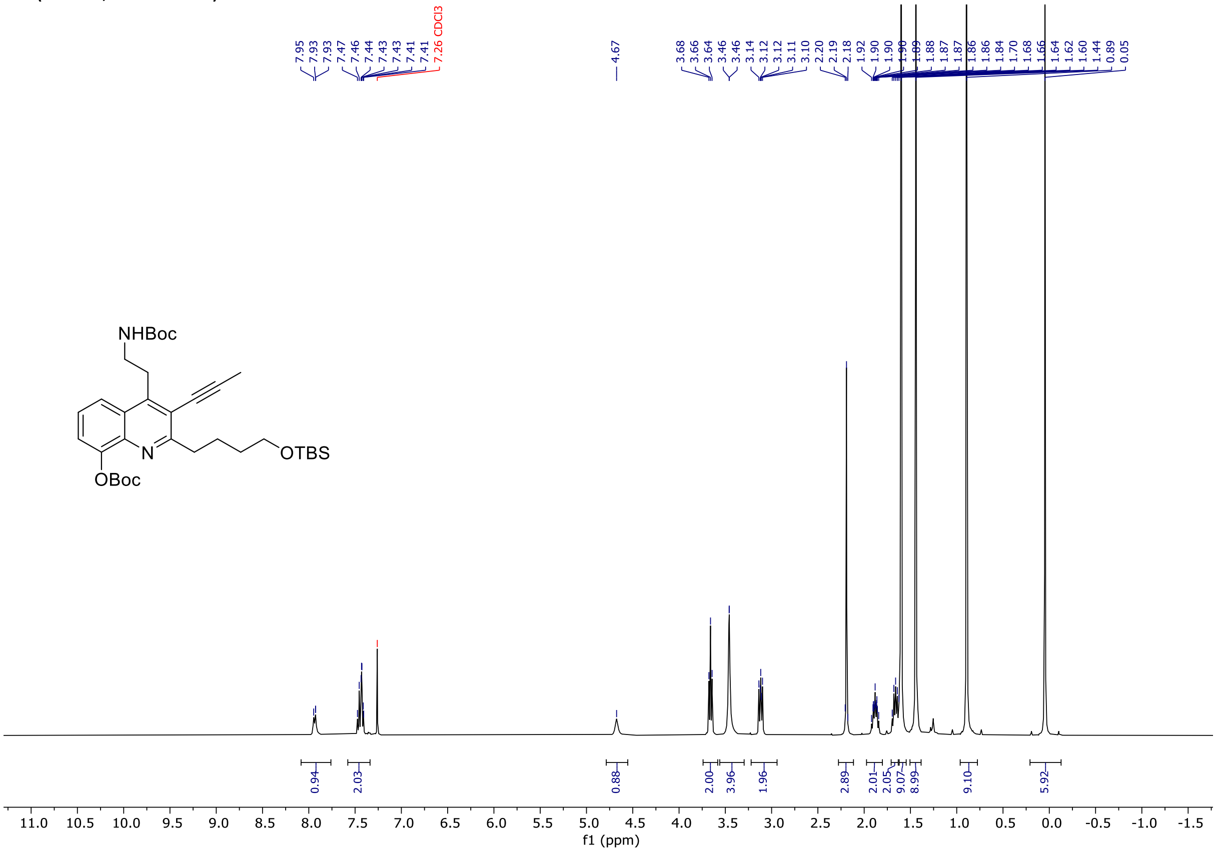

^{13}C ($CDCl_3$, 101 MHz)

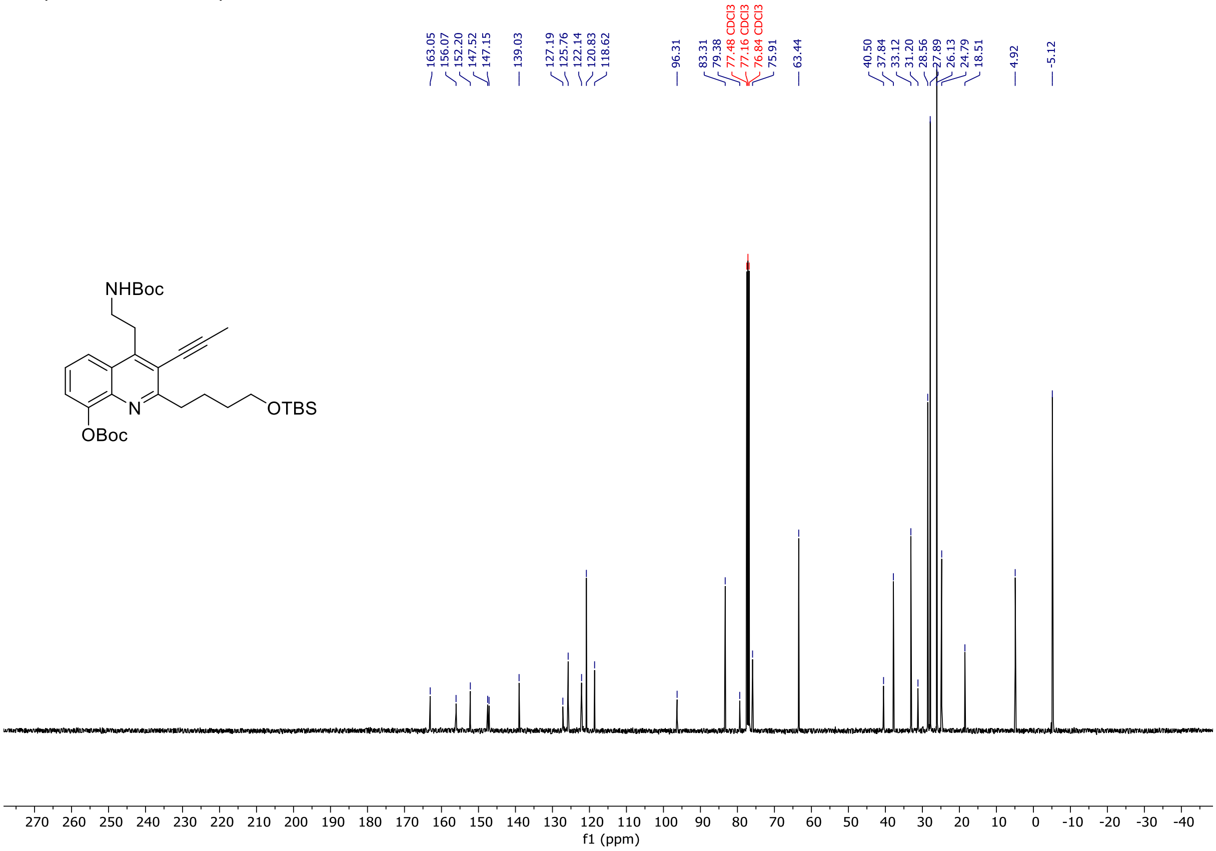

Compound S20

^{1}H ($CDCl_3$, 400 MHz)

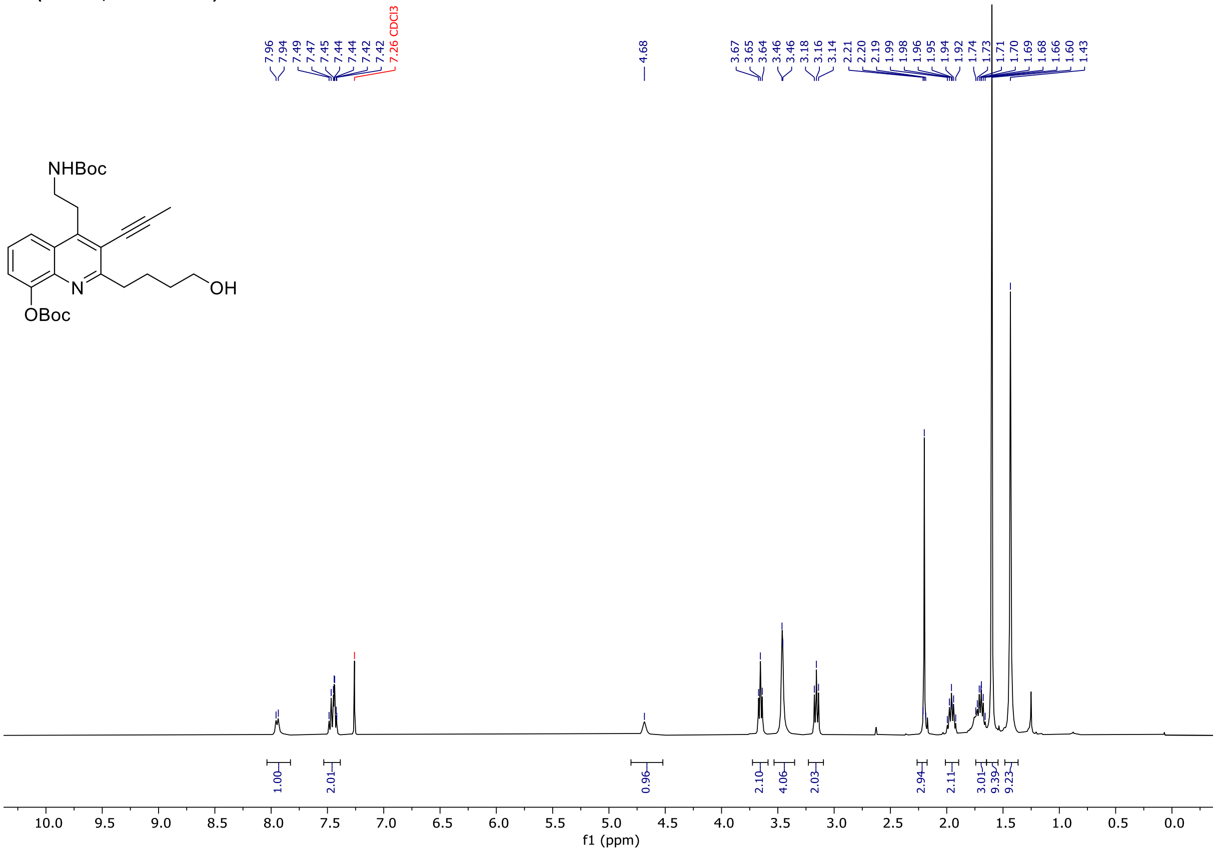

^{13}C ($CDCl_3$, 101 MHz)

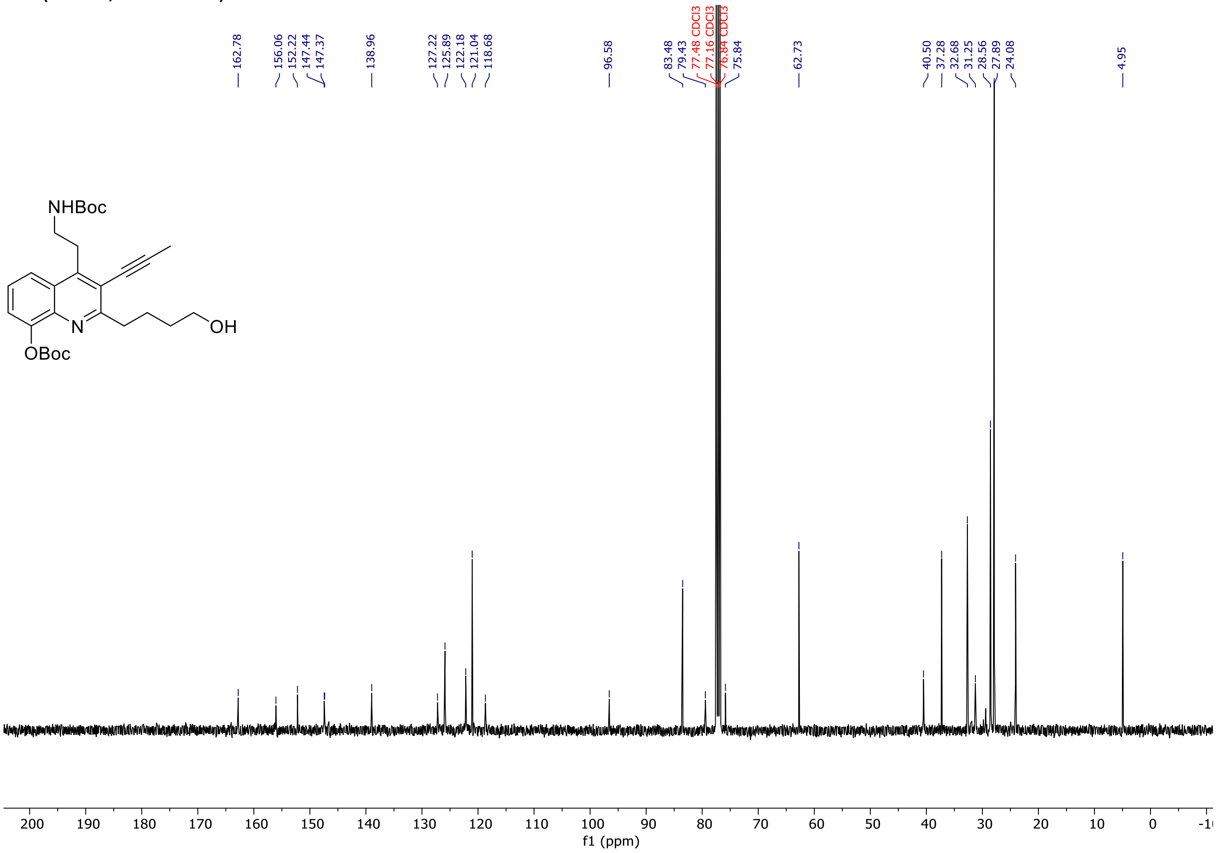

Compound 34

^{1}H (CDCl$_3$, 600 MHz)

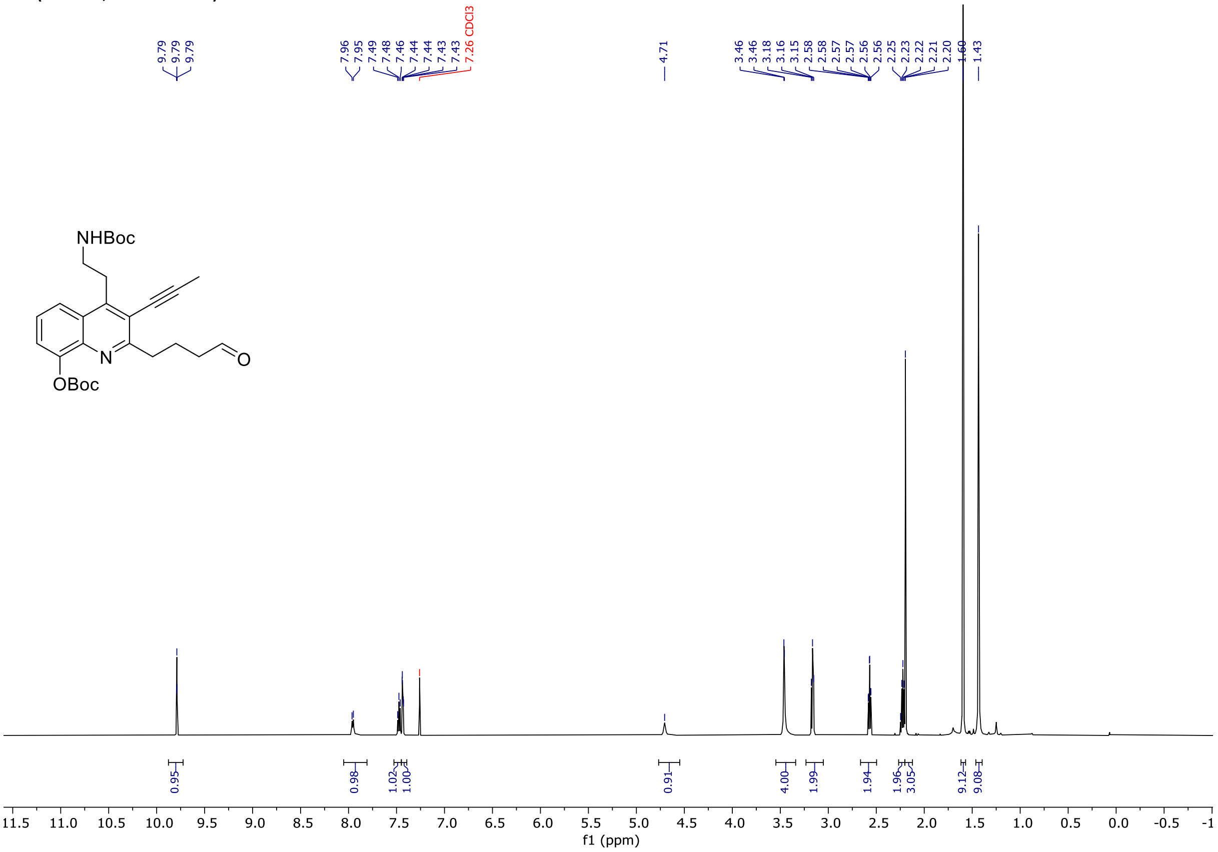

^{13}C (CDCl$_3$, 151 MHz)

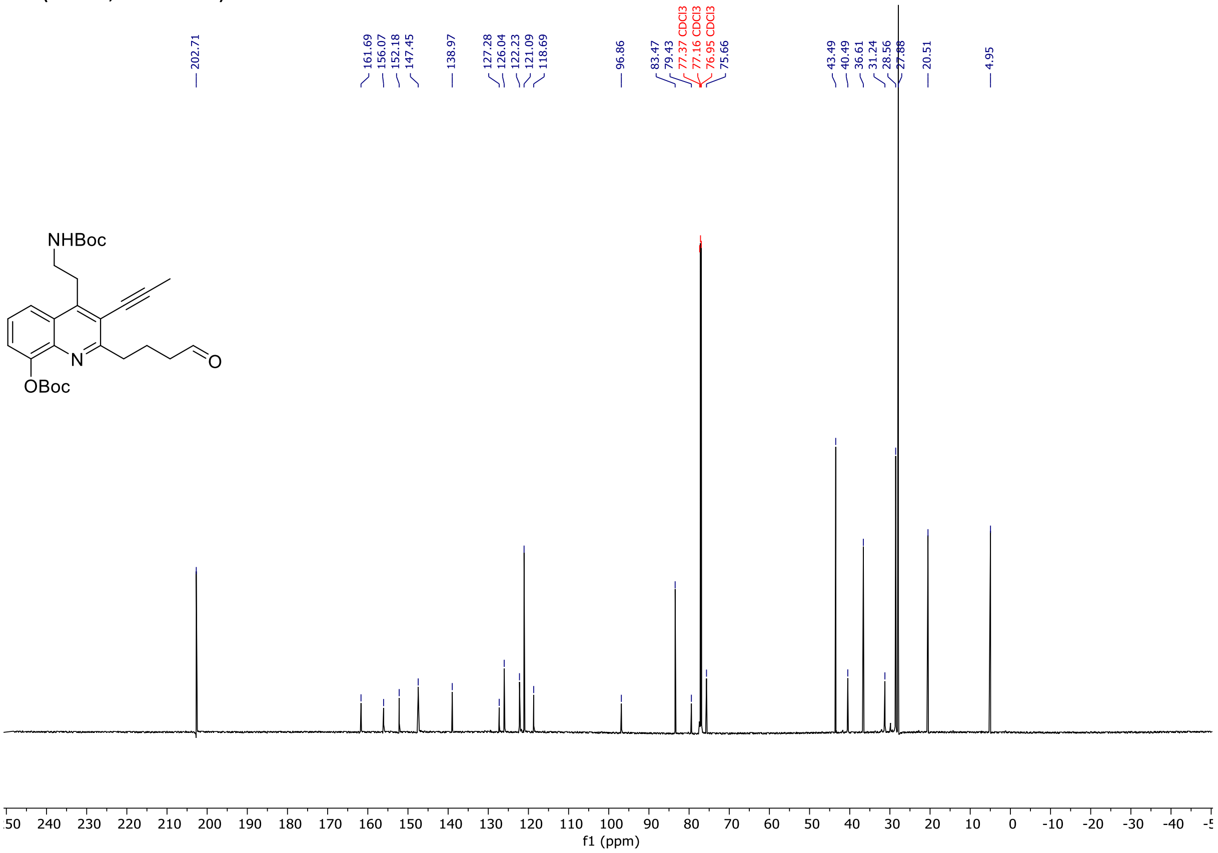

Compound 35

^{1}H (CD_2Cl_2, 600 MHz)

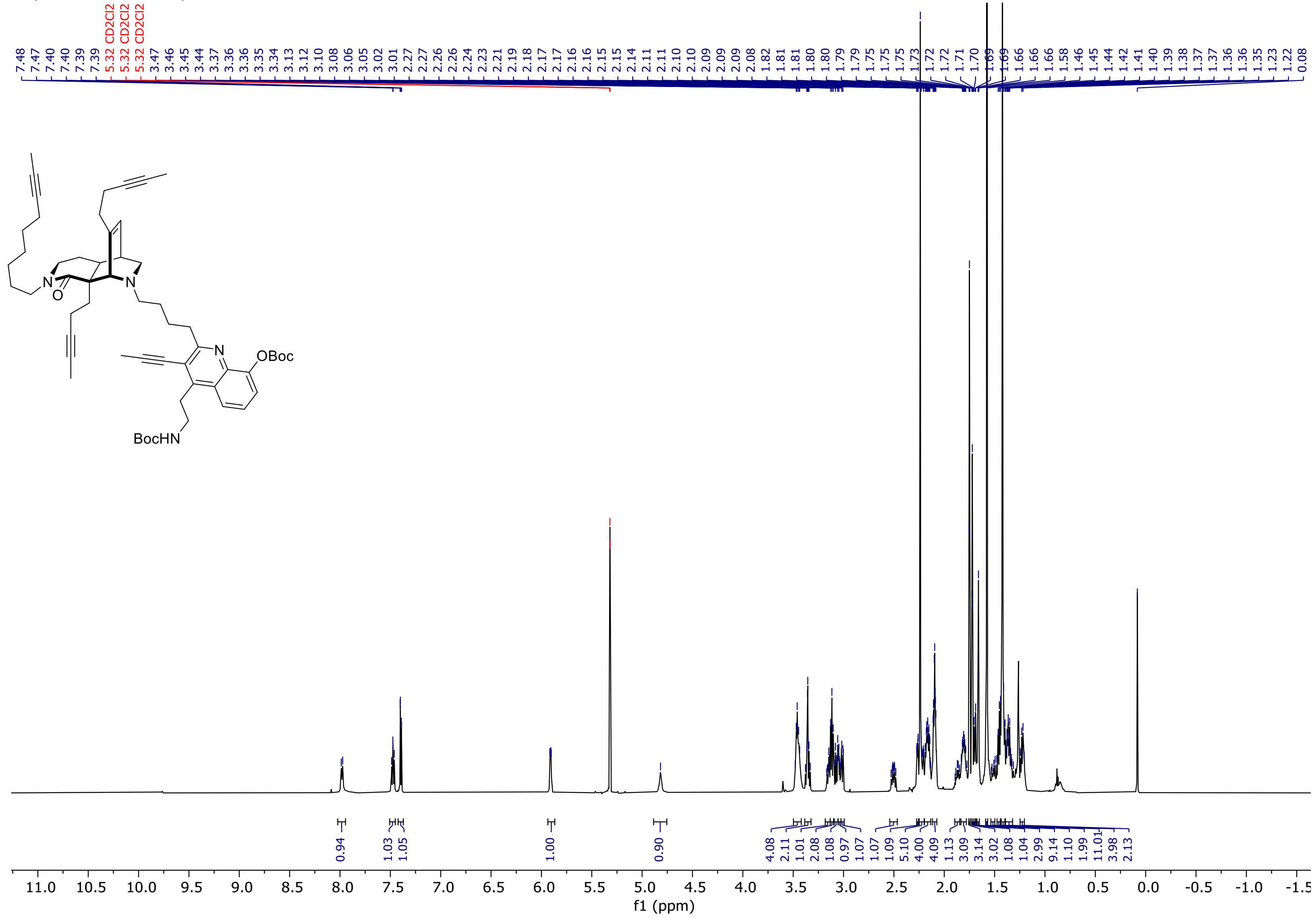

Compound 35

^{13}C (CD_2Cl_2, 151 MHz)

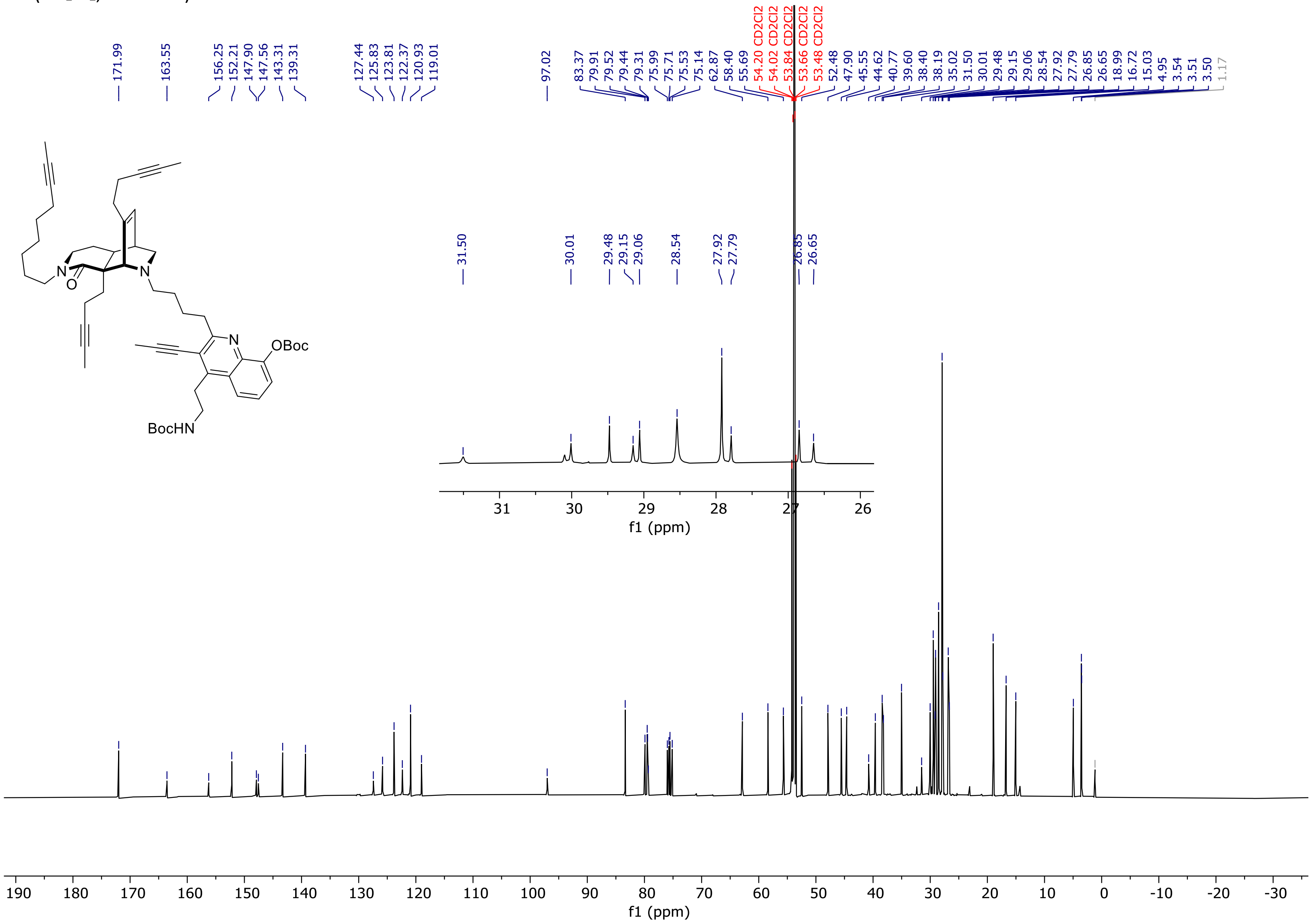

Compound 36

^1H (CD_2Cl_2, 400 MHz)

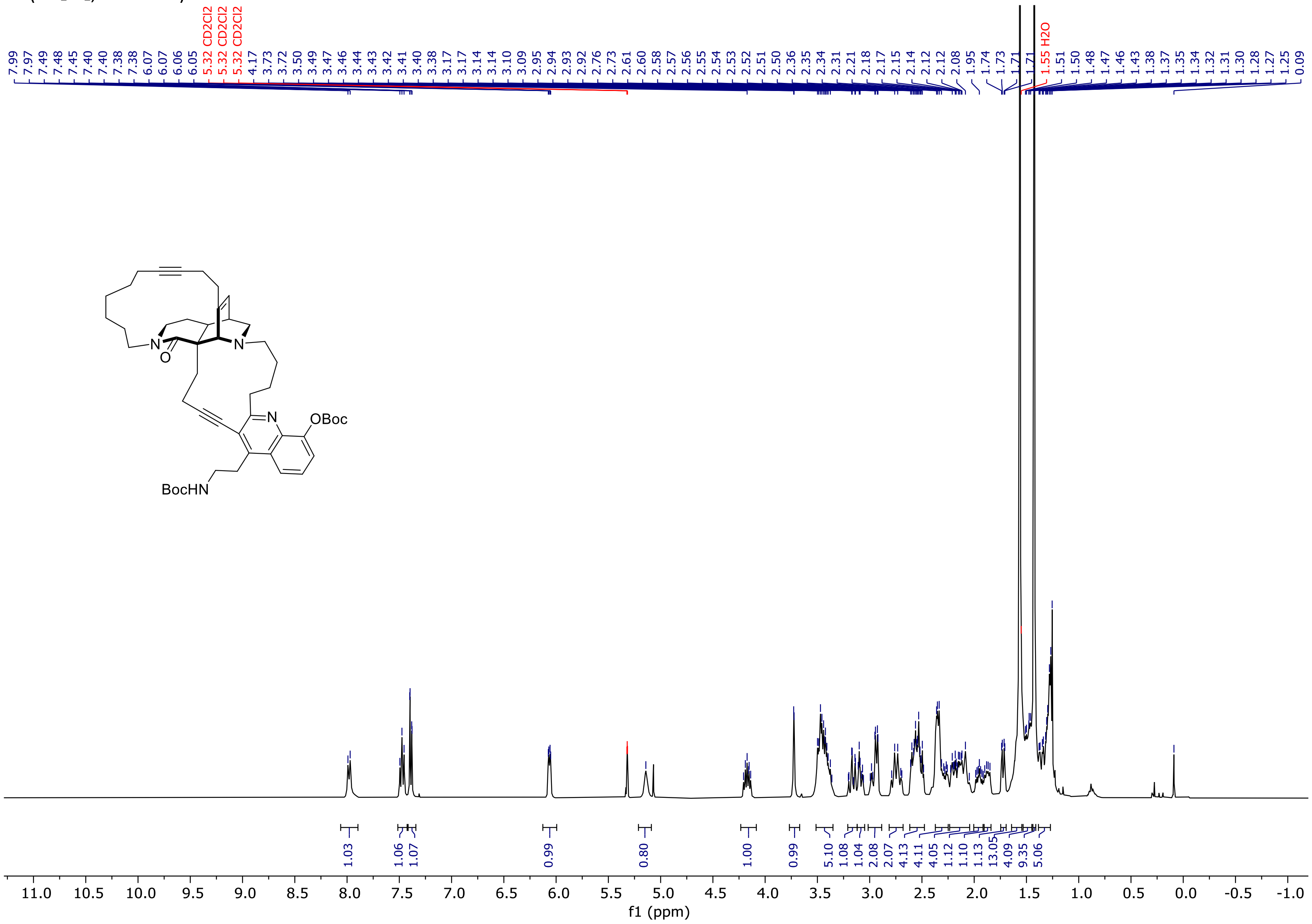

Compound 36

^{13}C (CD_2Cl_2, 101 MHz)

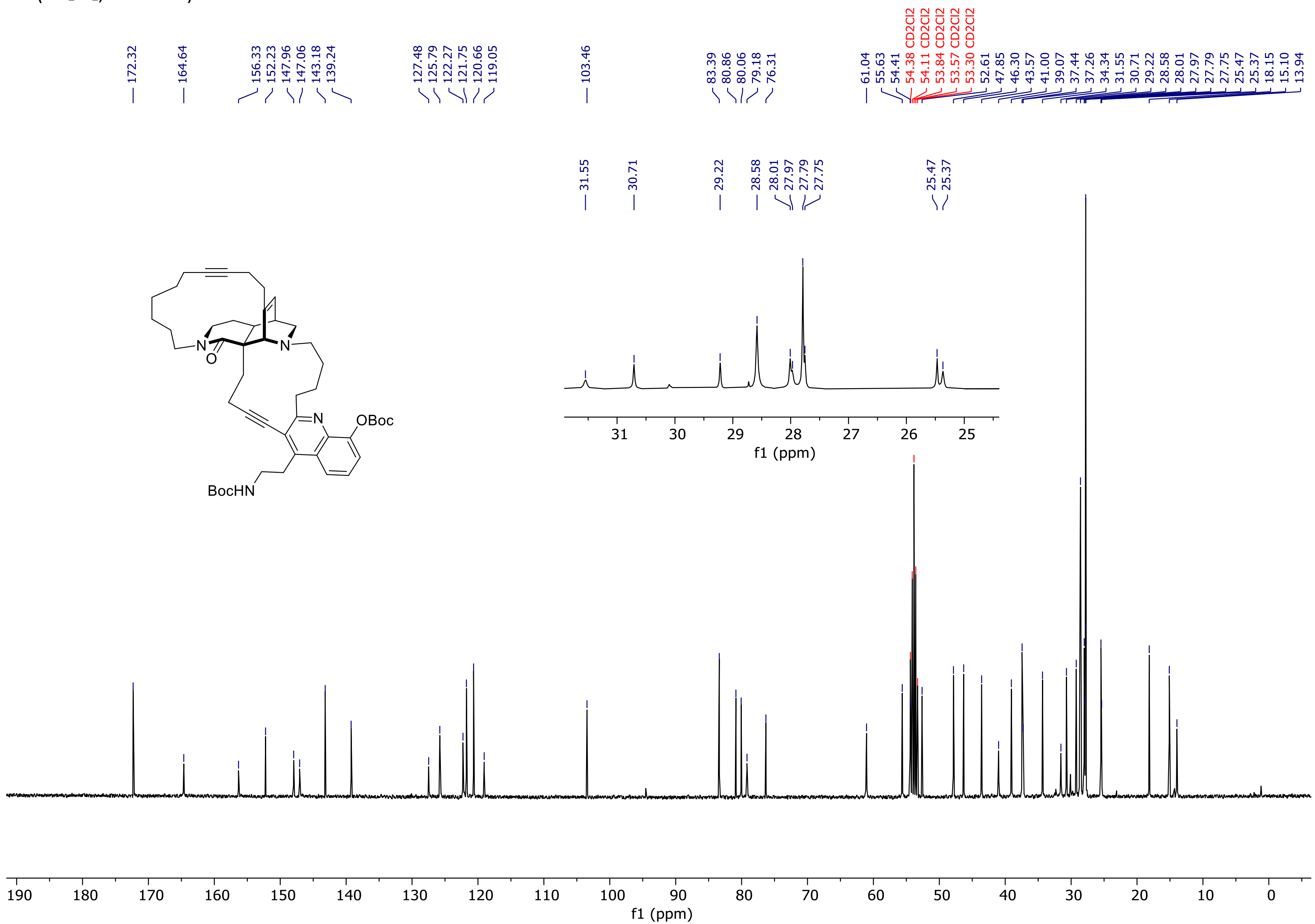

COSY Spectrum of **36**

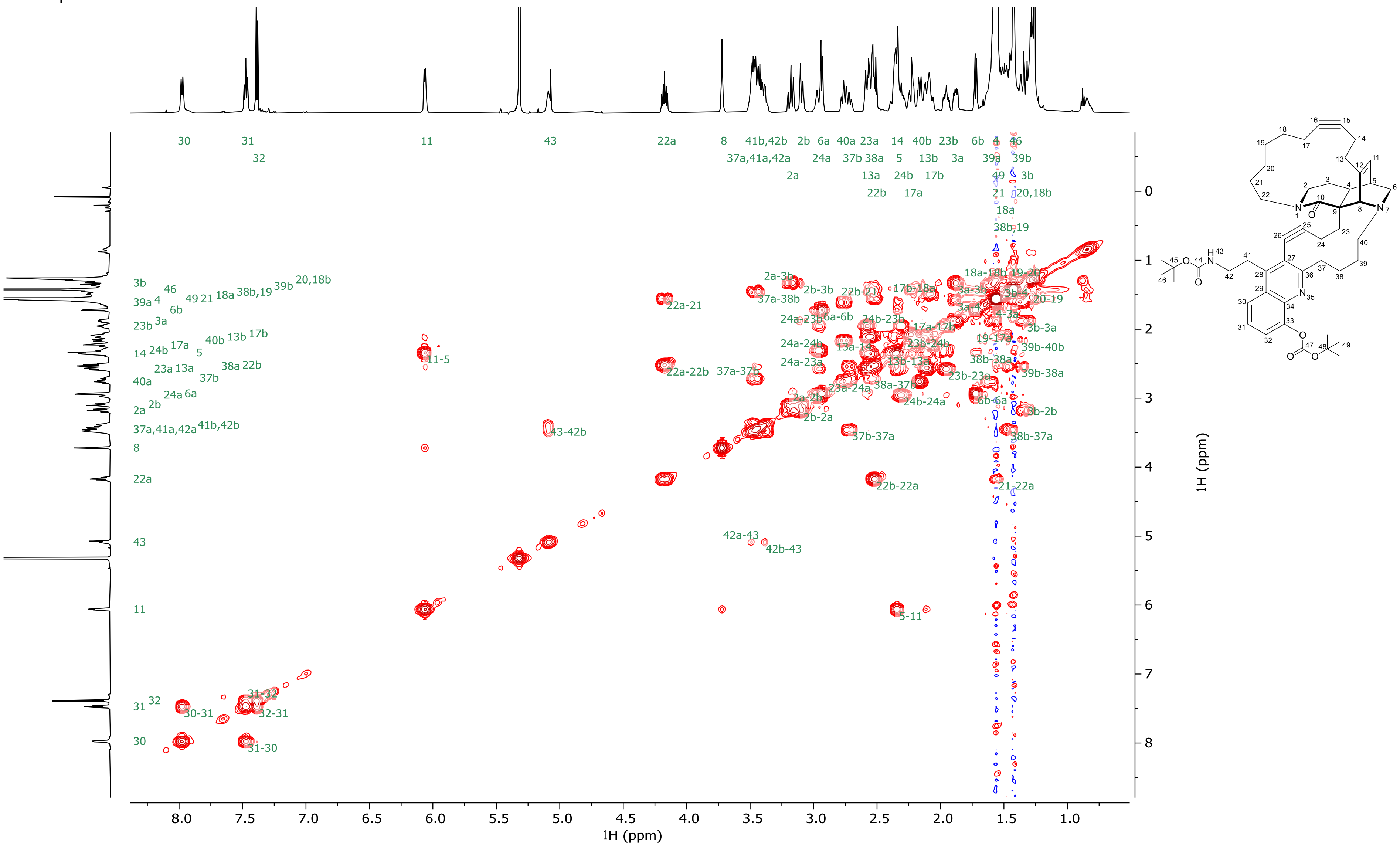

HSQC Spectrum of **36**

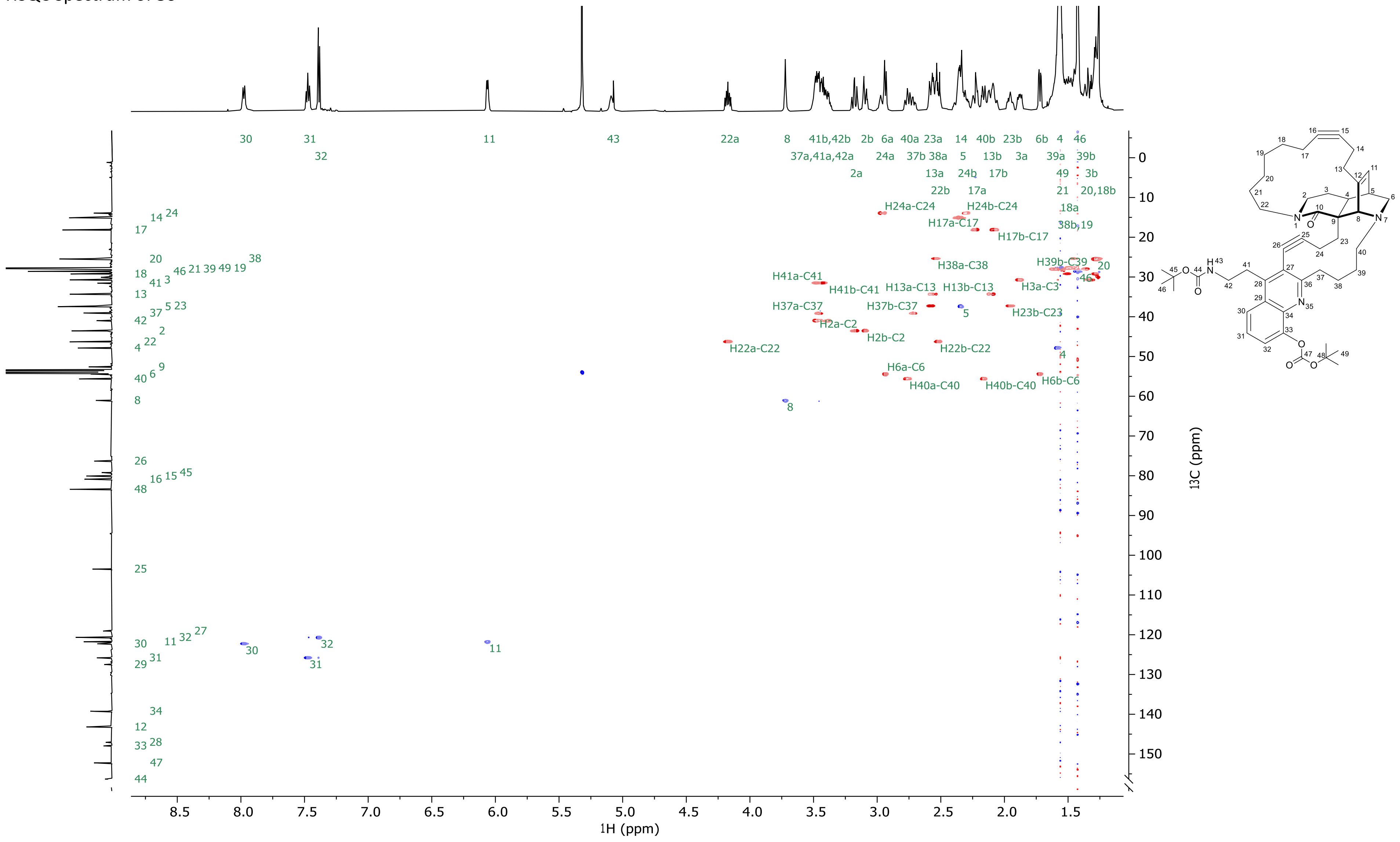

HMBC Spectrum of **36**

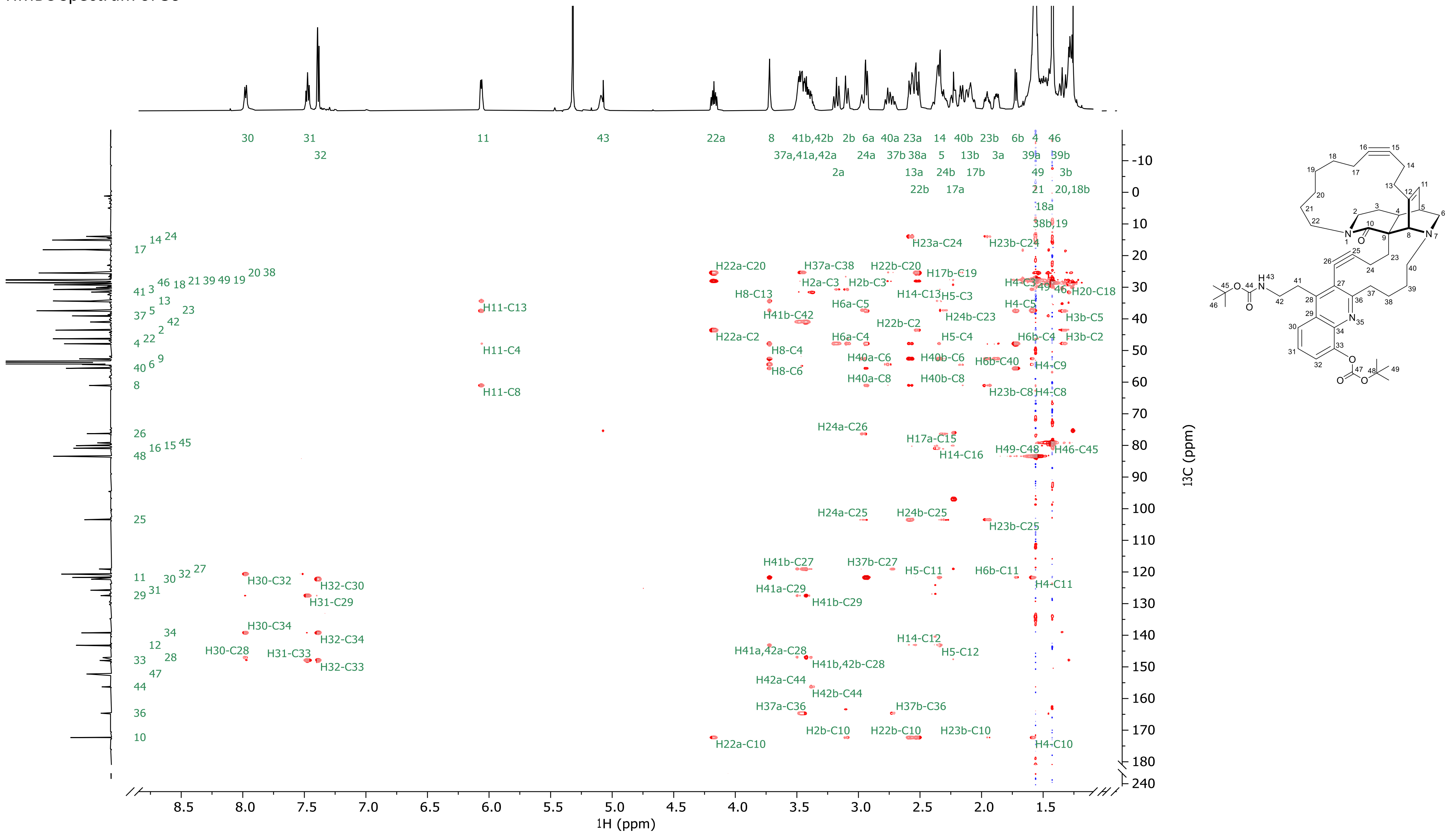

NOESY Spectrum of **36**

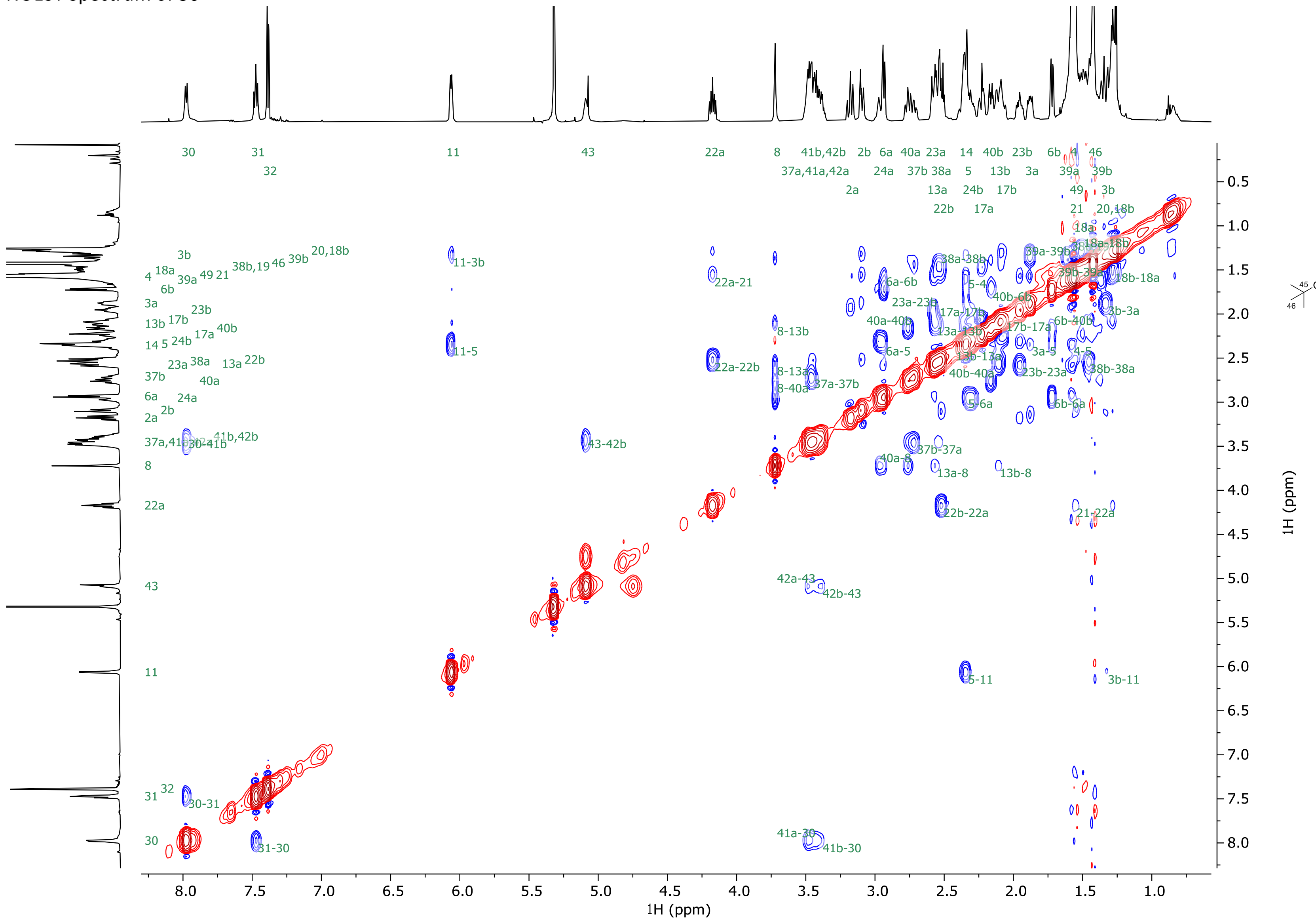

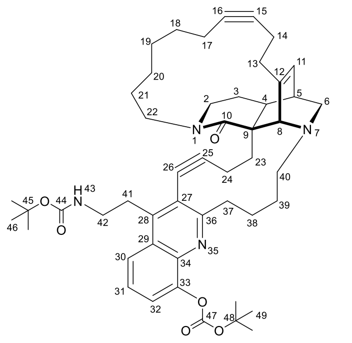

Compound 37

^{1}H (CD_2Cl_2, 600 MHz)

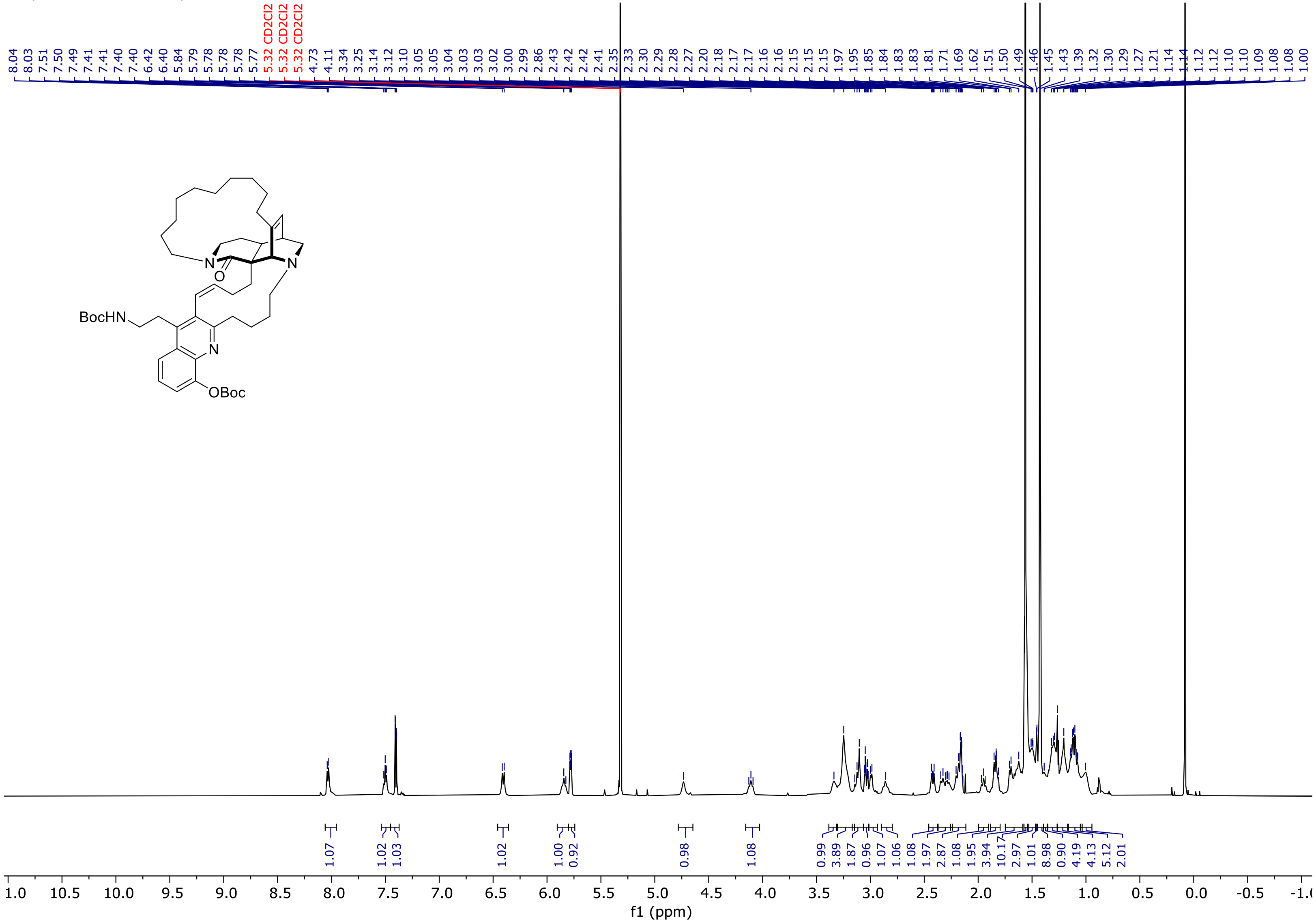

Compound 37

^{13}C (CD_2Cl_2, 151 MHz)

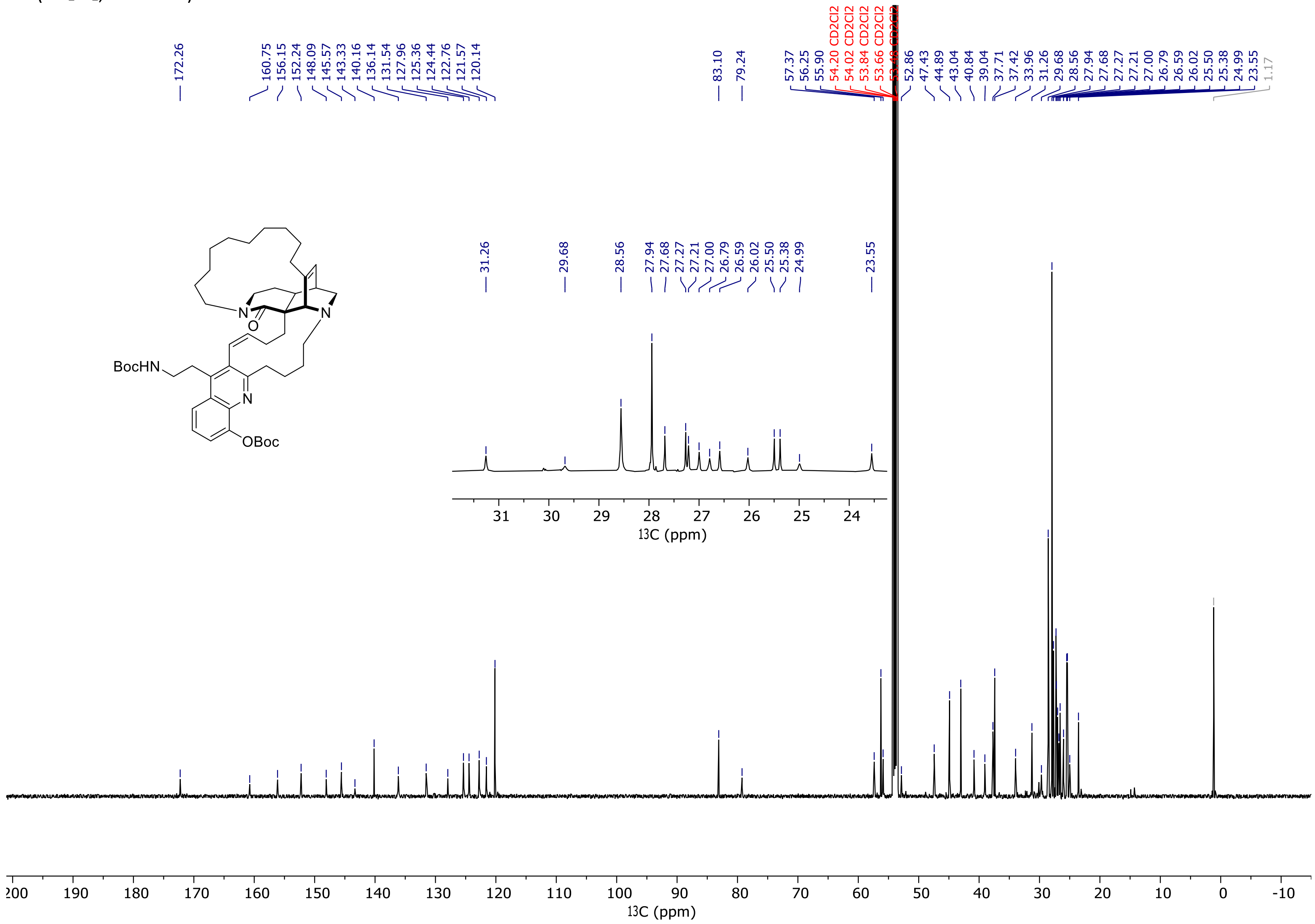

COSY Spectrum of **37**

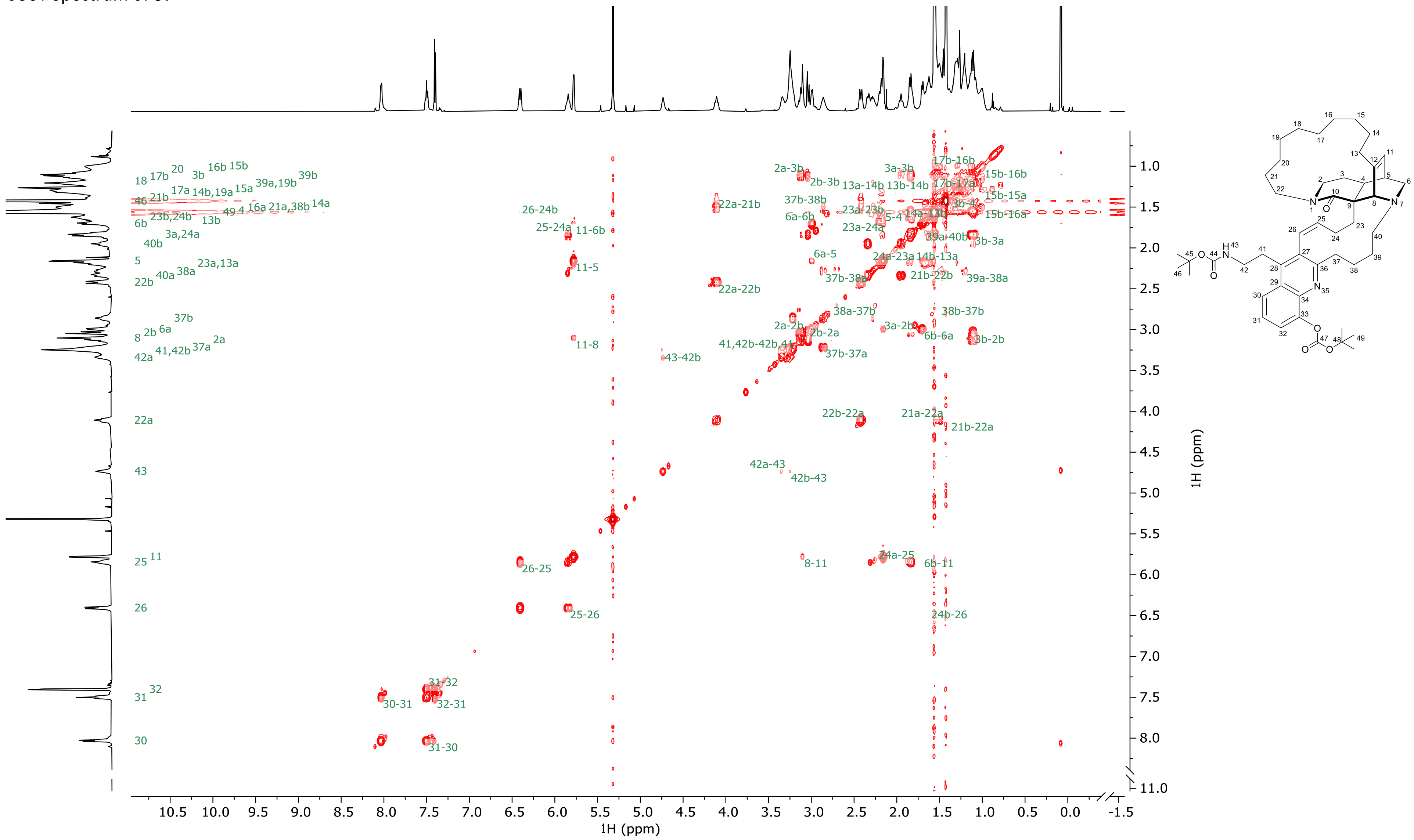

HSQC Spectrum of **37**

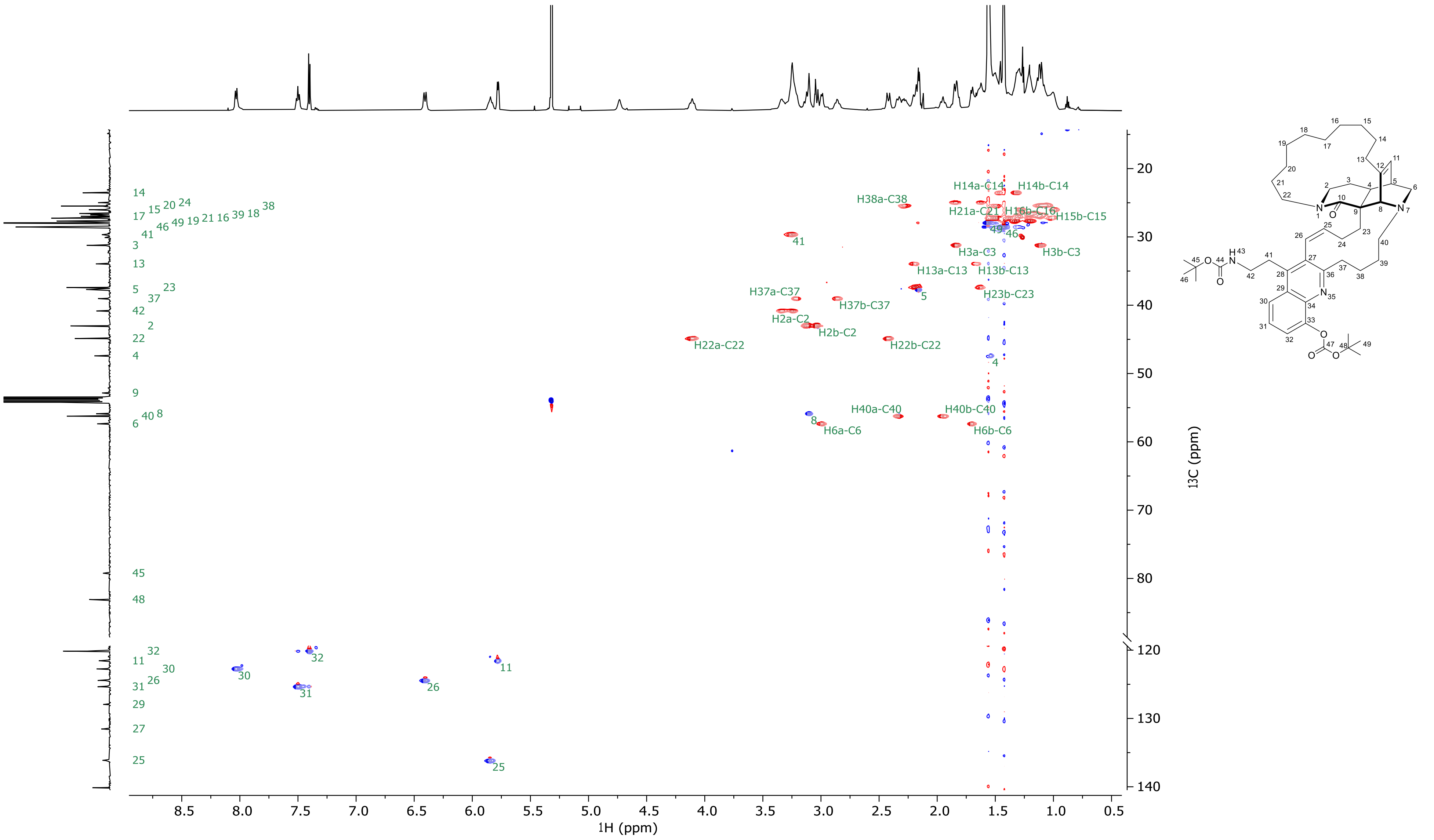
H14a-C14
H14b-C14
H38a-C38
H21a-C21
H16b-C16
H15b-C15
H3a-C3
H3b-C3
H13a-C13
H13b-C13
H23b-C23
H37a-C37
H37b-C37
H2a-C2
H2b-C2
H22a-C22
H22b-C22
H40a-C40
H40b-C40
H6a-C6
H6b-C6
1H (ppm)
13C (ppm)

HMBC Spectrum of **37**

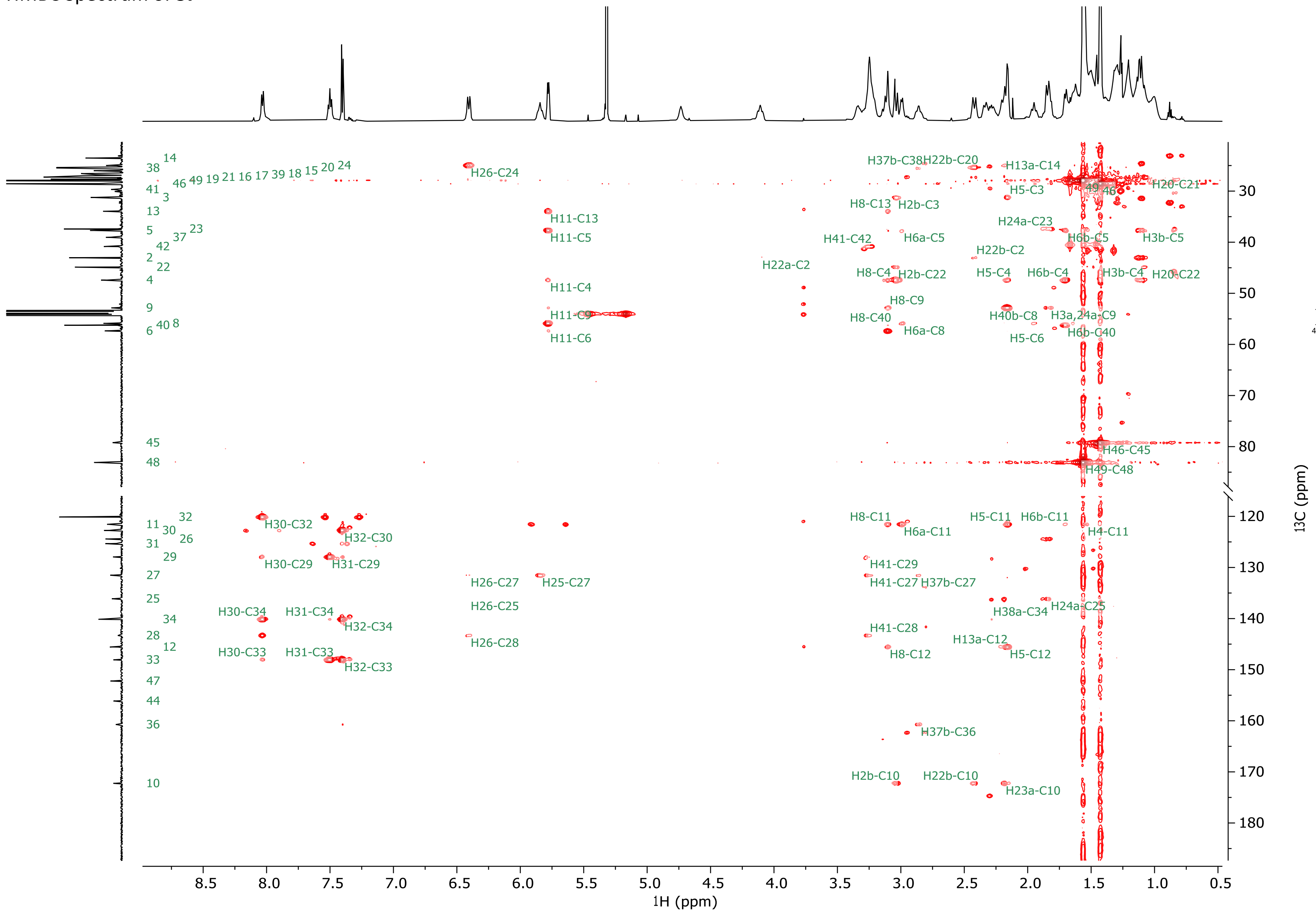

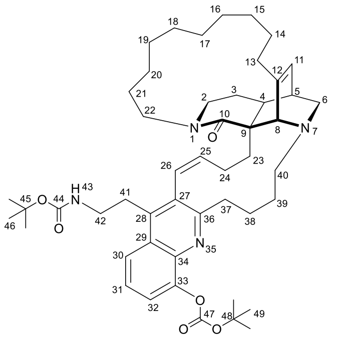

NOESY Spectrum of **37**

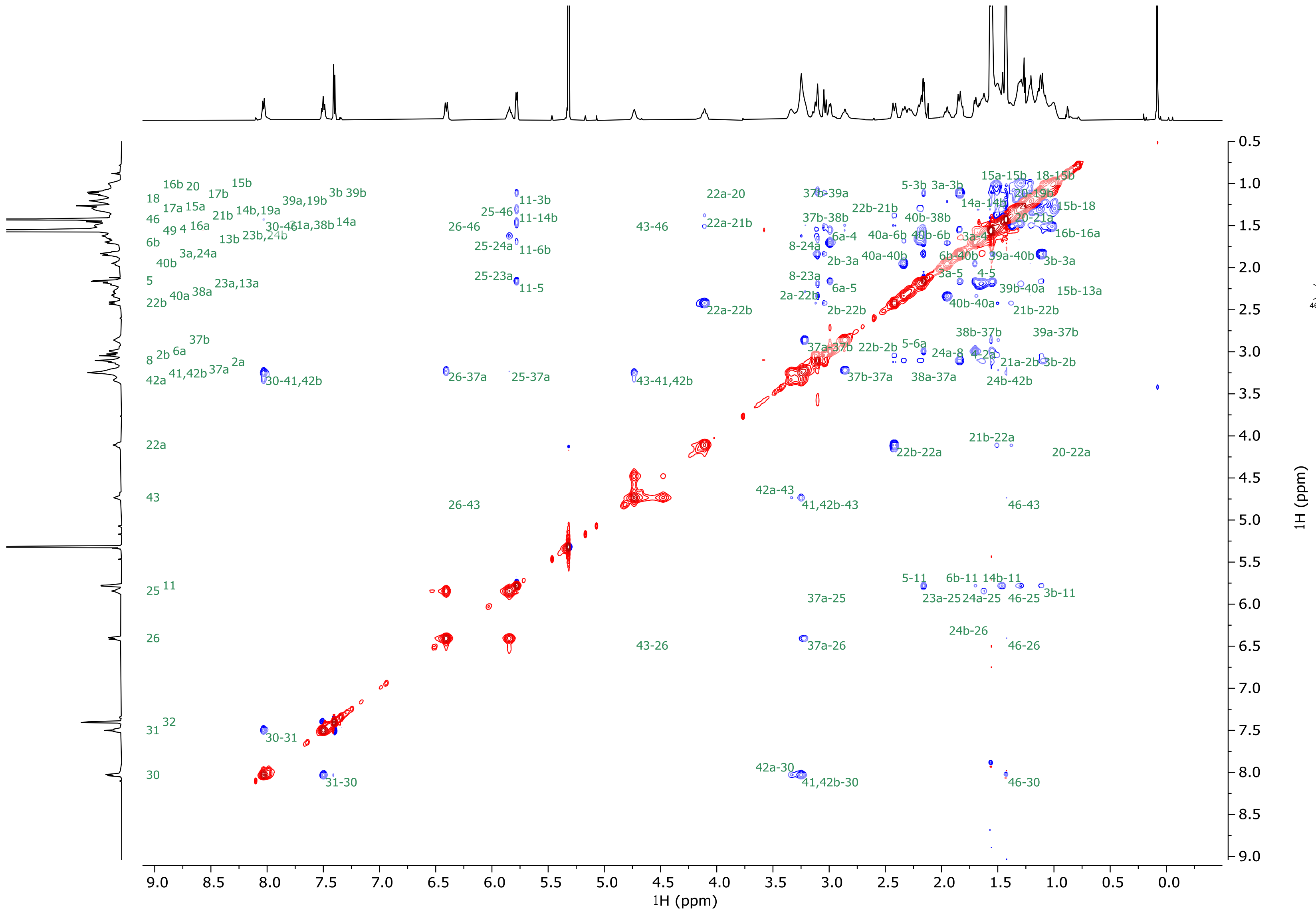
1H (ppm)
1H (ppm)
9.0 8.5 8.0 7.5 7.0 6.5 6.0 5.5 5.0 4.5 4.0 3.5 3.0 2.5 2.0 1.5 1.0 0.5 0.0
0.5 1.0 1.5 2.0 2.5 3.0 3.5 4.0 4.5 5.0 5.5 6.0 6.5 7.0 7.5 8.0 8.5 9.0

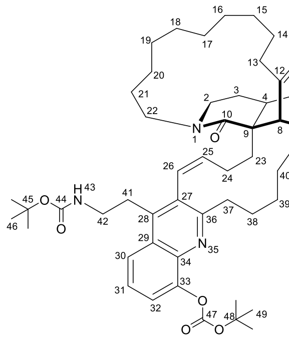

Compound 38

^{1}H (CD_2Cl_2, 400 MHz)

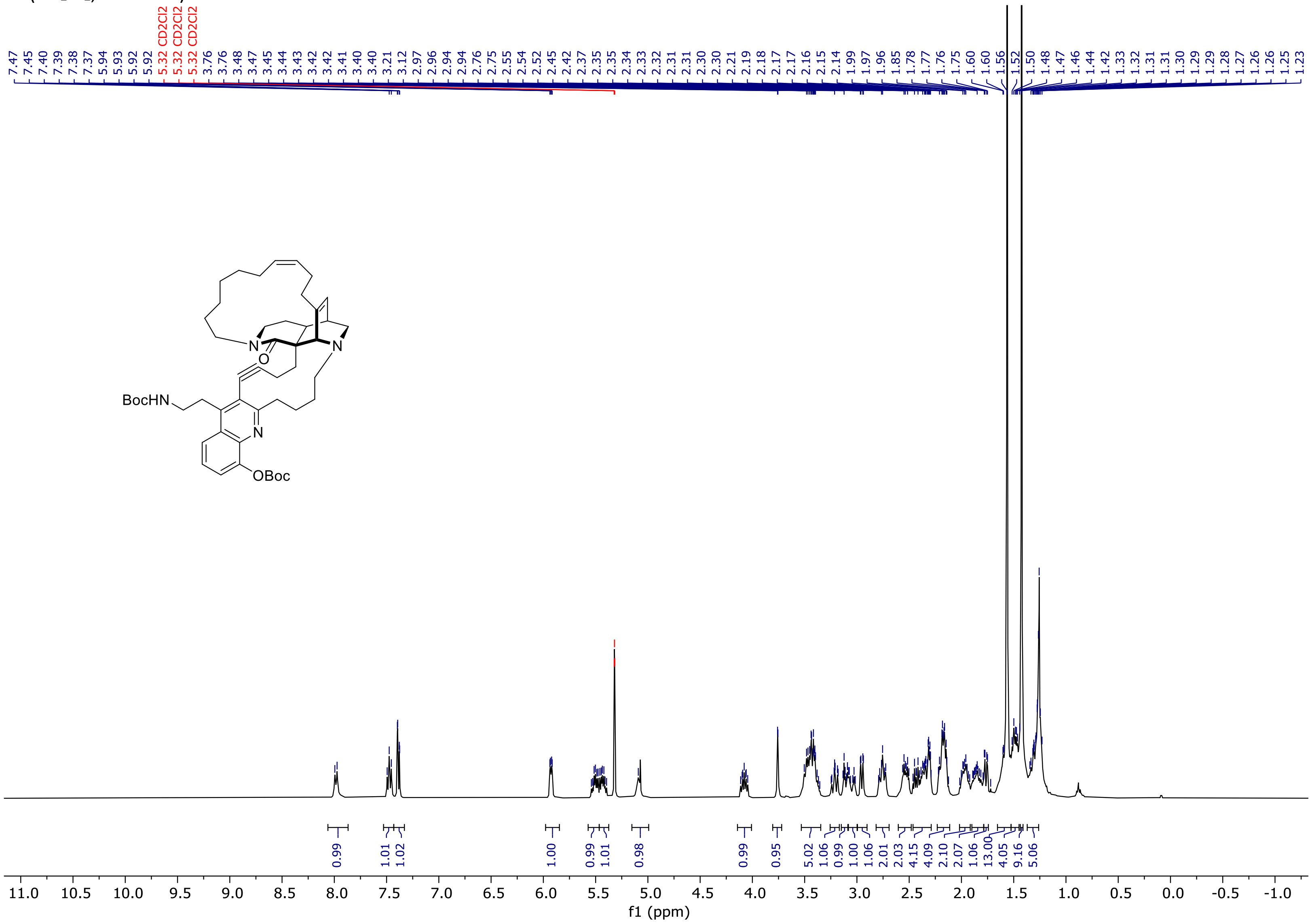

Compound 38

^{13}C (CD_2Cl_2, 101 MHz)

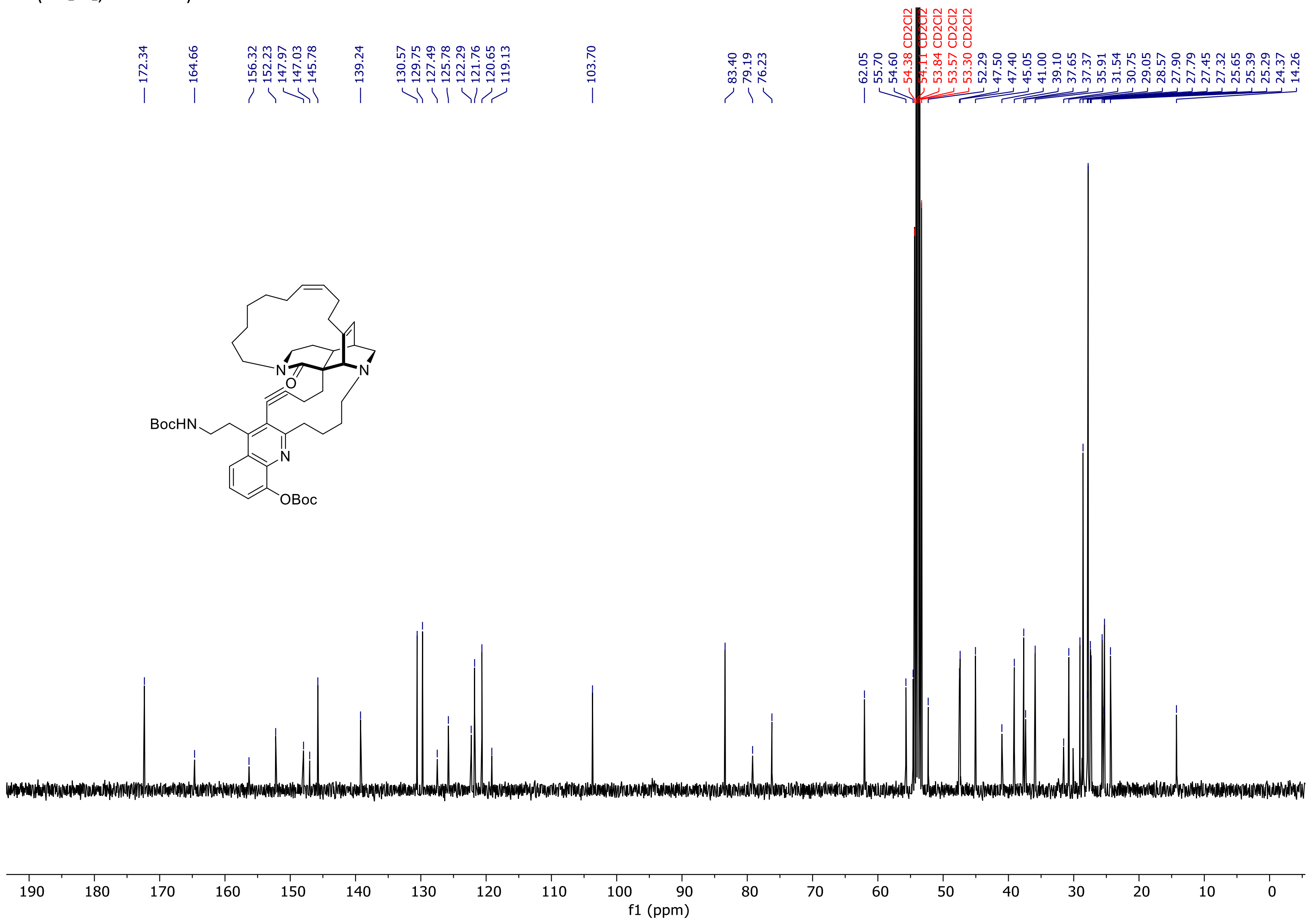

COSY Spectrum of **38**

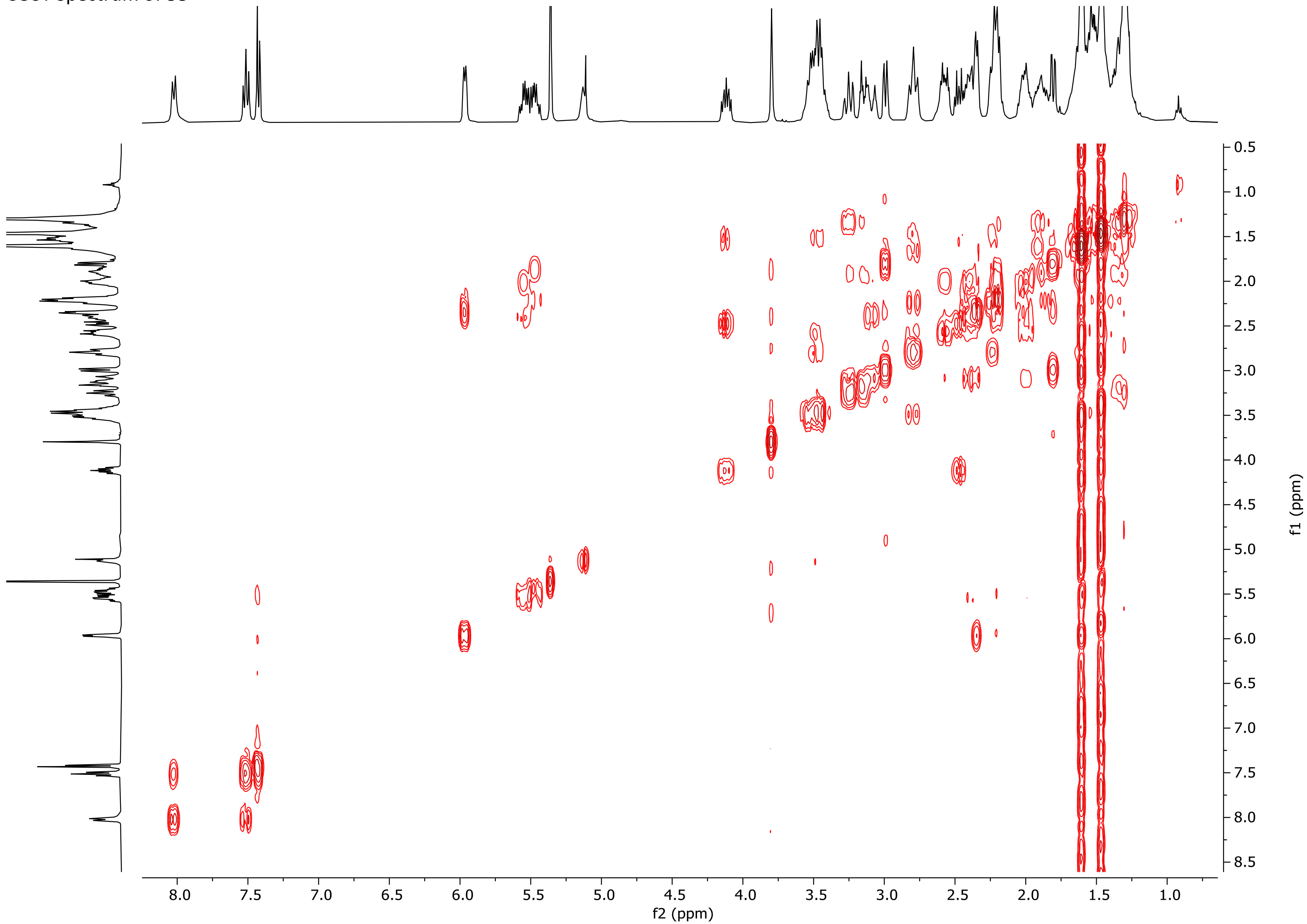

HSQC Spectrum of **38**

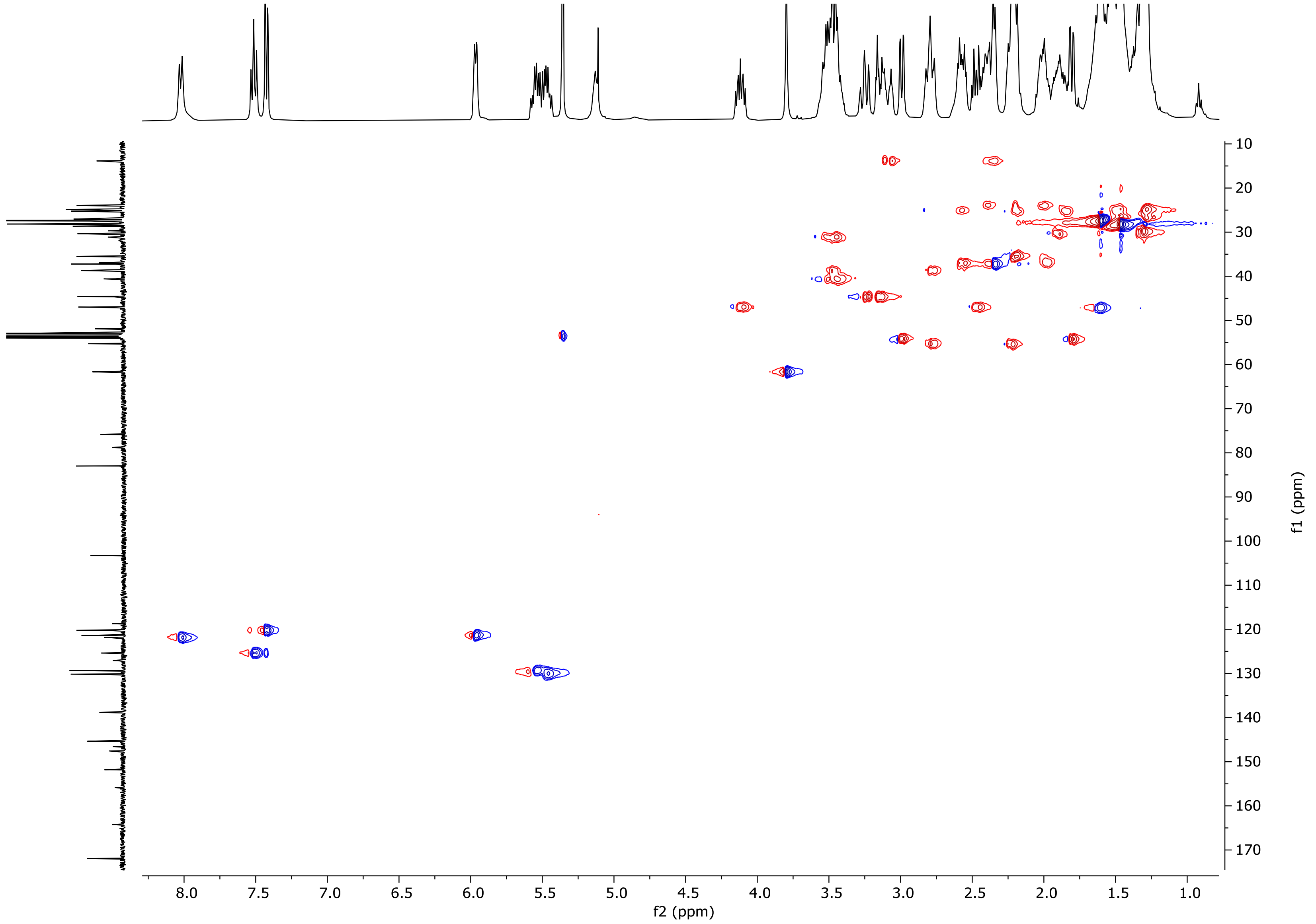

HMBC Spectrum of **38**

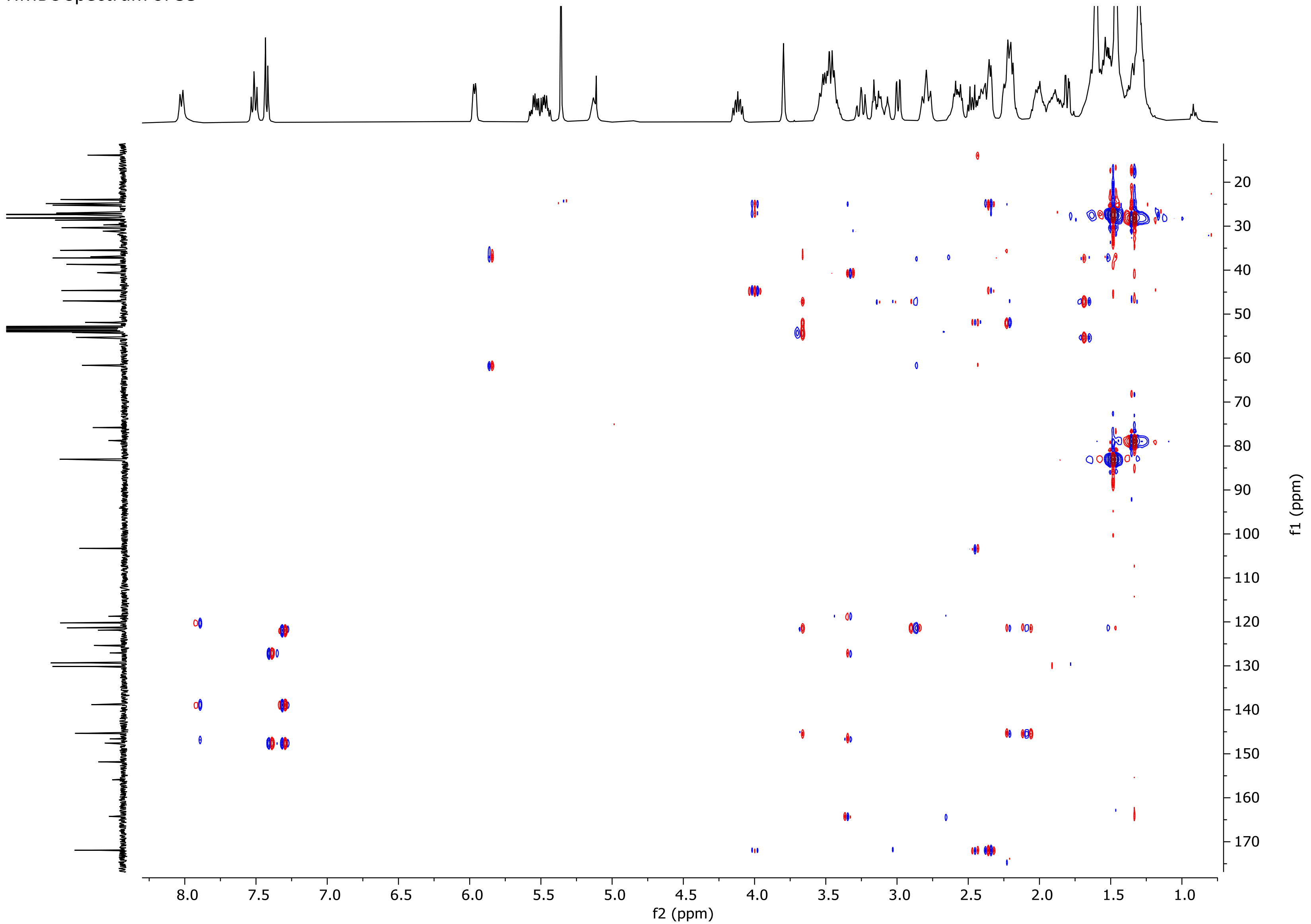

Compound 39

^{1}H (CD_2Cl_2, 600 MHz)

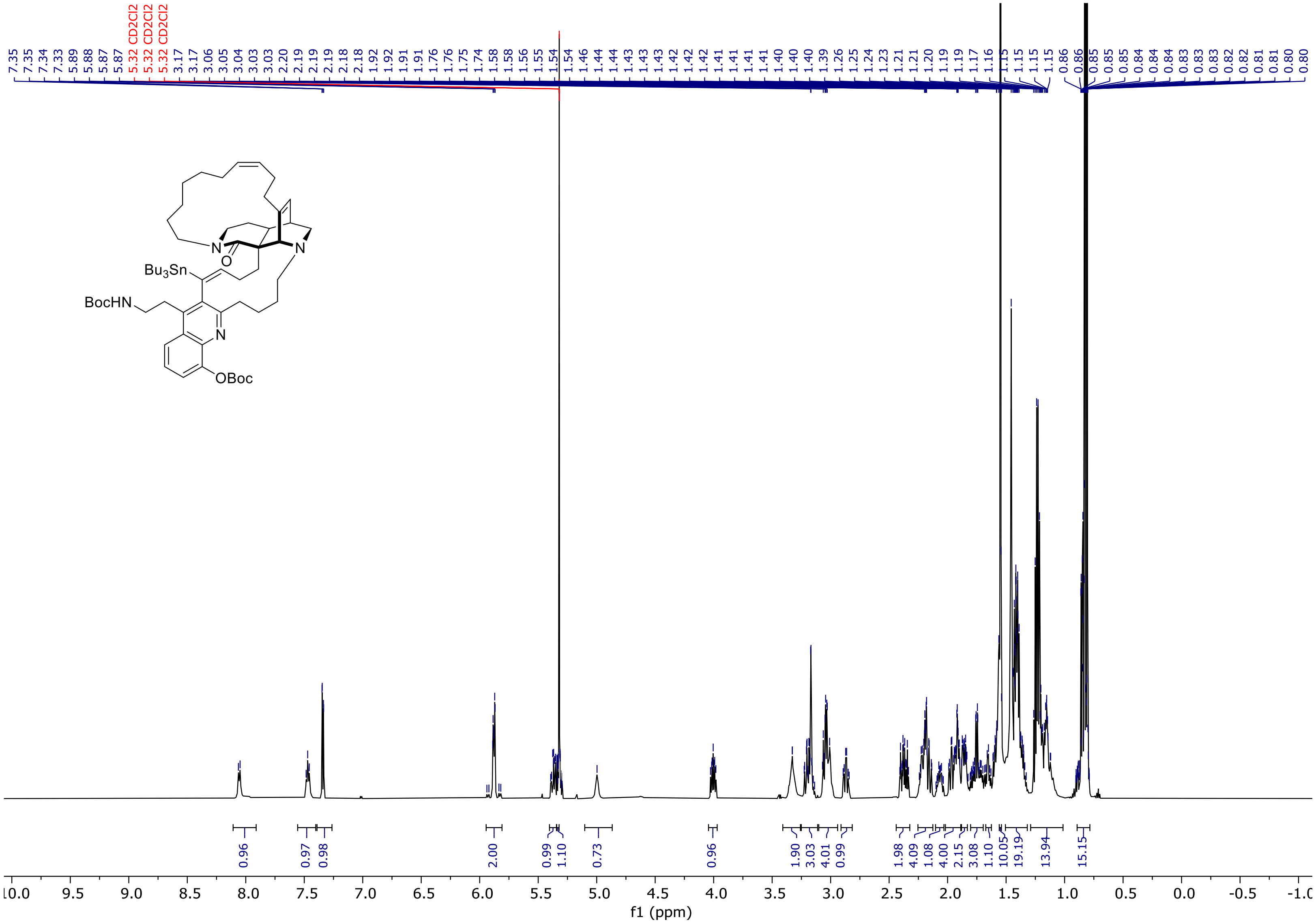

Compound 39

^{13}C (CD_2Cl_2, 151 MHz)

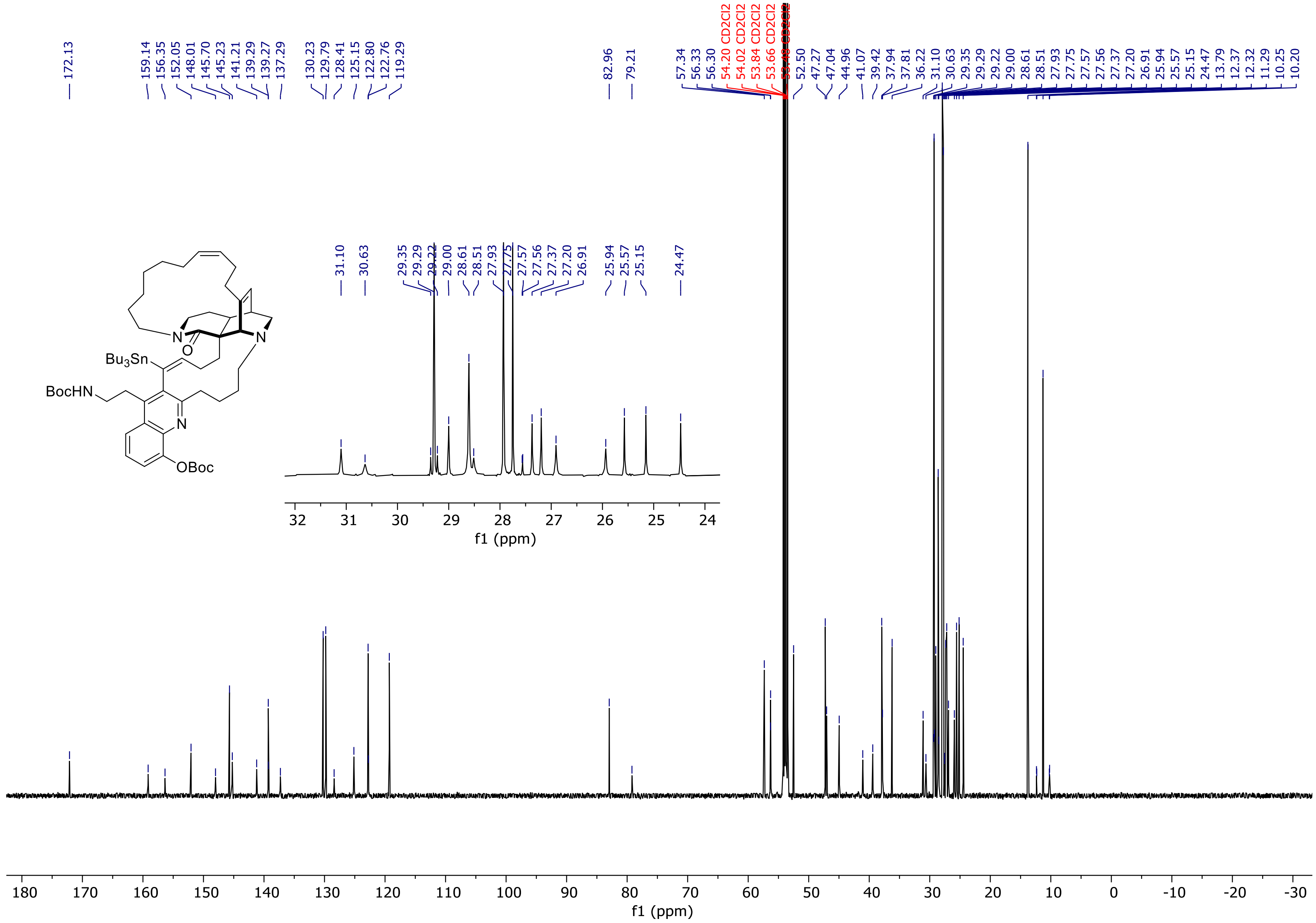

Compound 39

^{119}Sn (CD_2Cl_2, 149 MHz)

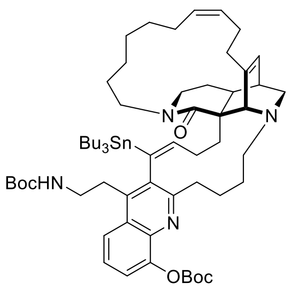

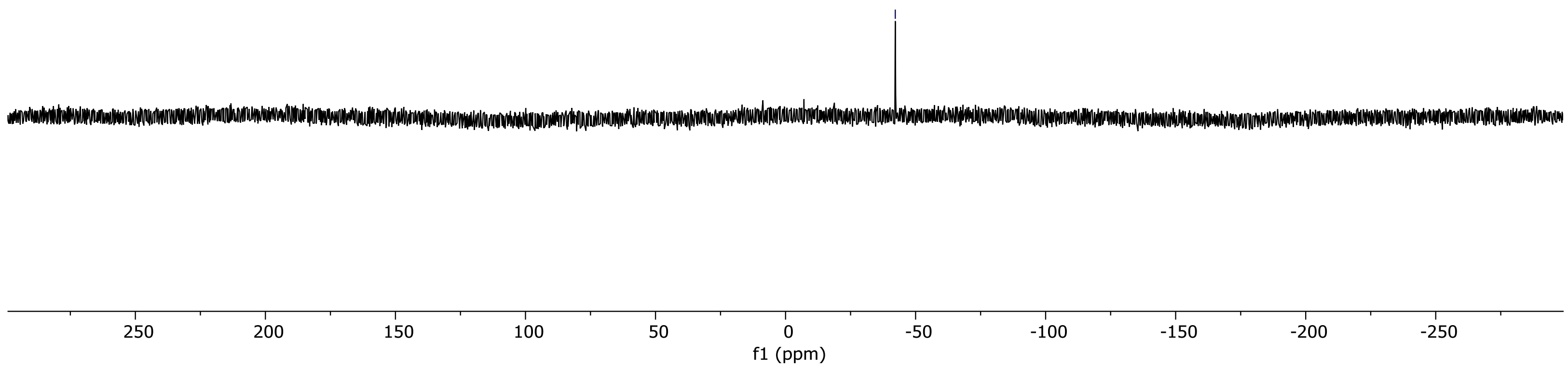

COSY Spectrum of **39**

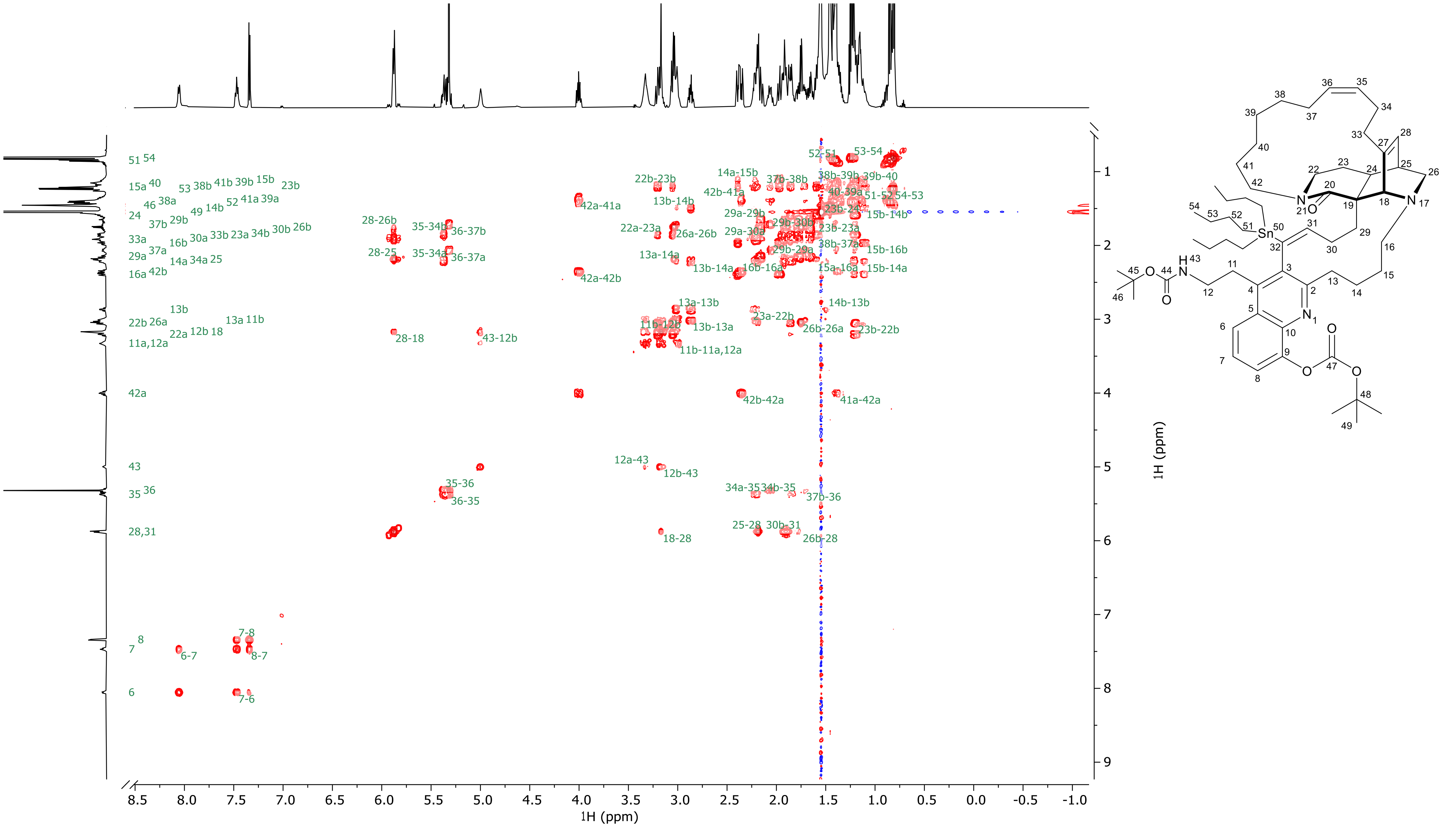

HSQC Spectrum of **39**

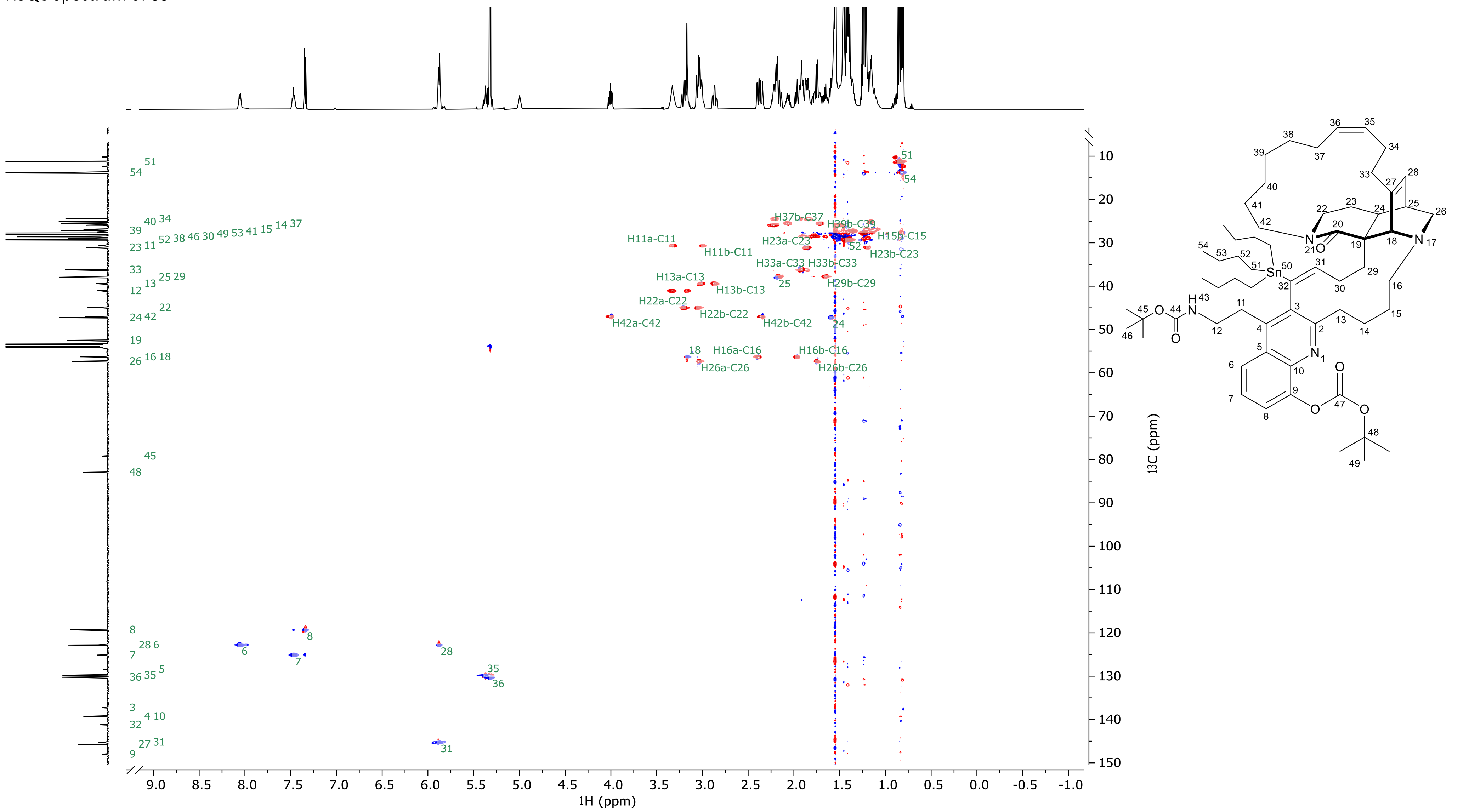

HMBC Spectrum of **39**

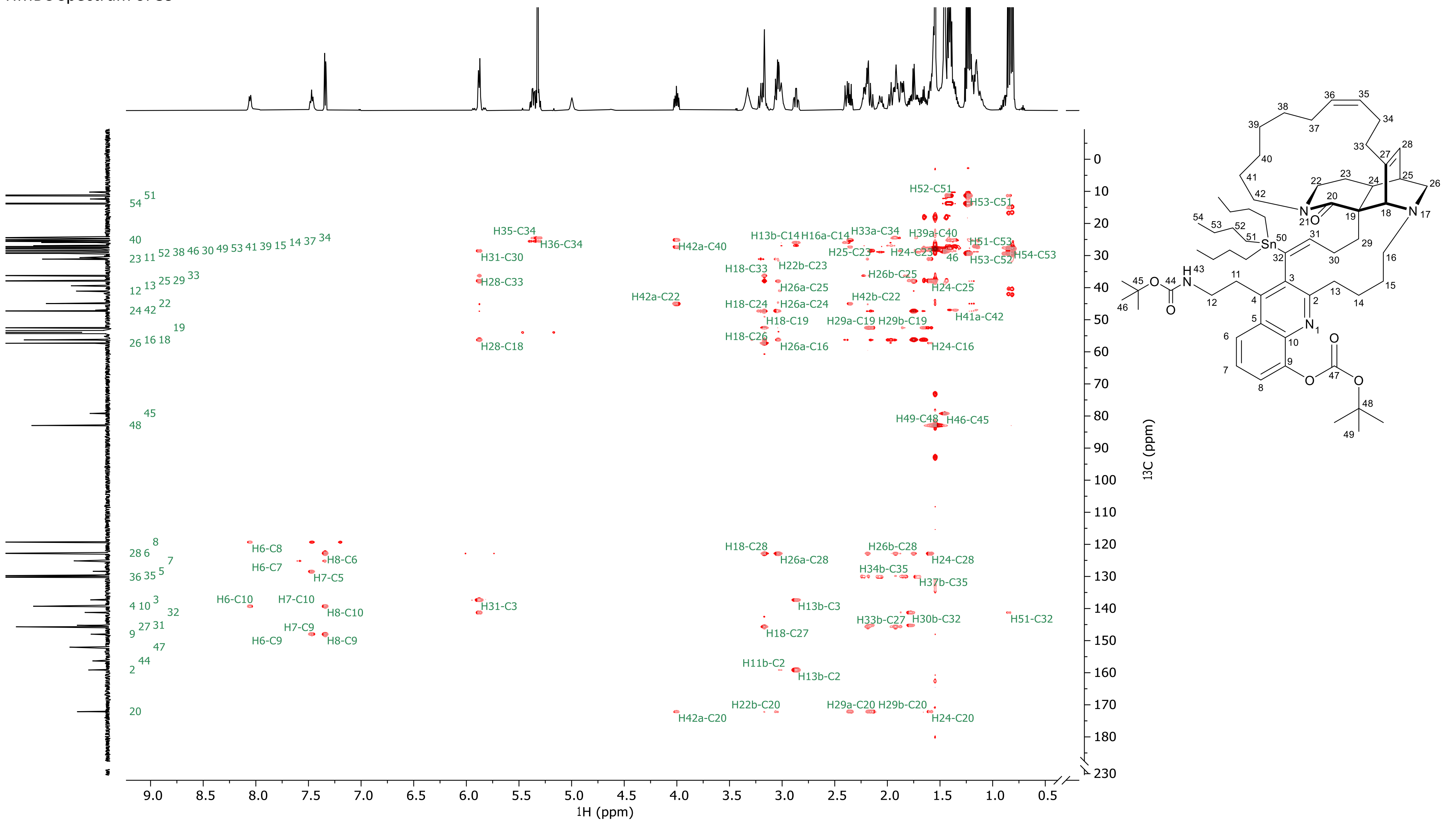

NOESY Spectrum of **39**

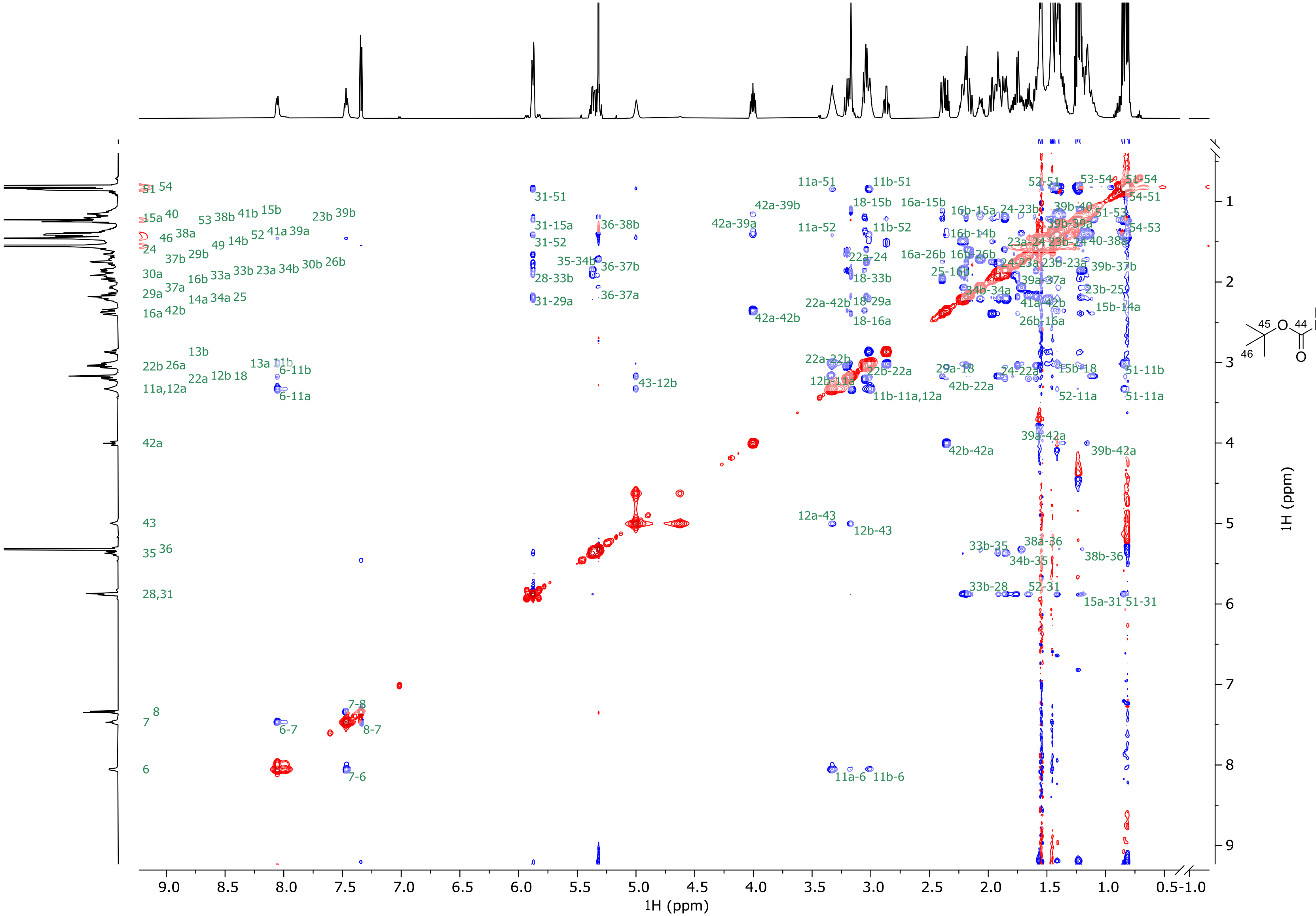

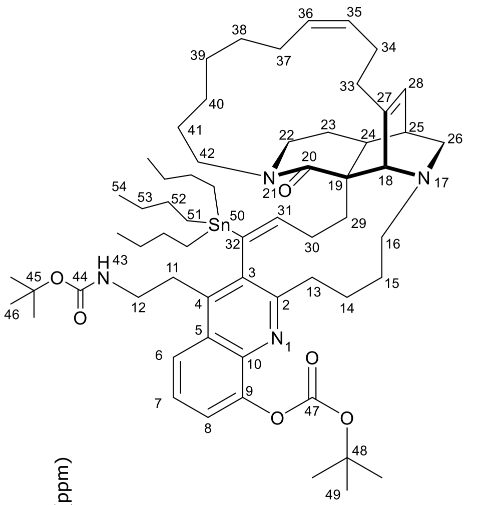

Compound S25

^{1}H (CD_2Cl_2, 600 MHz)

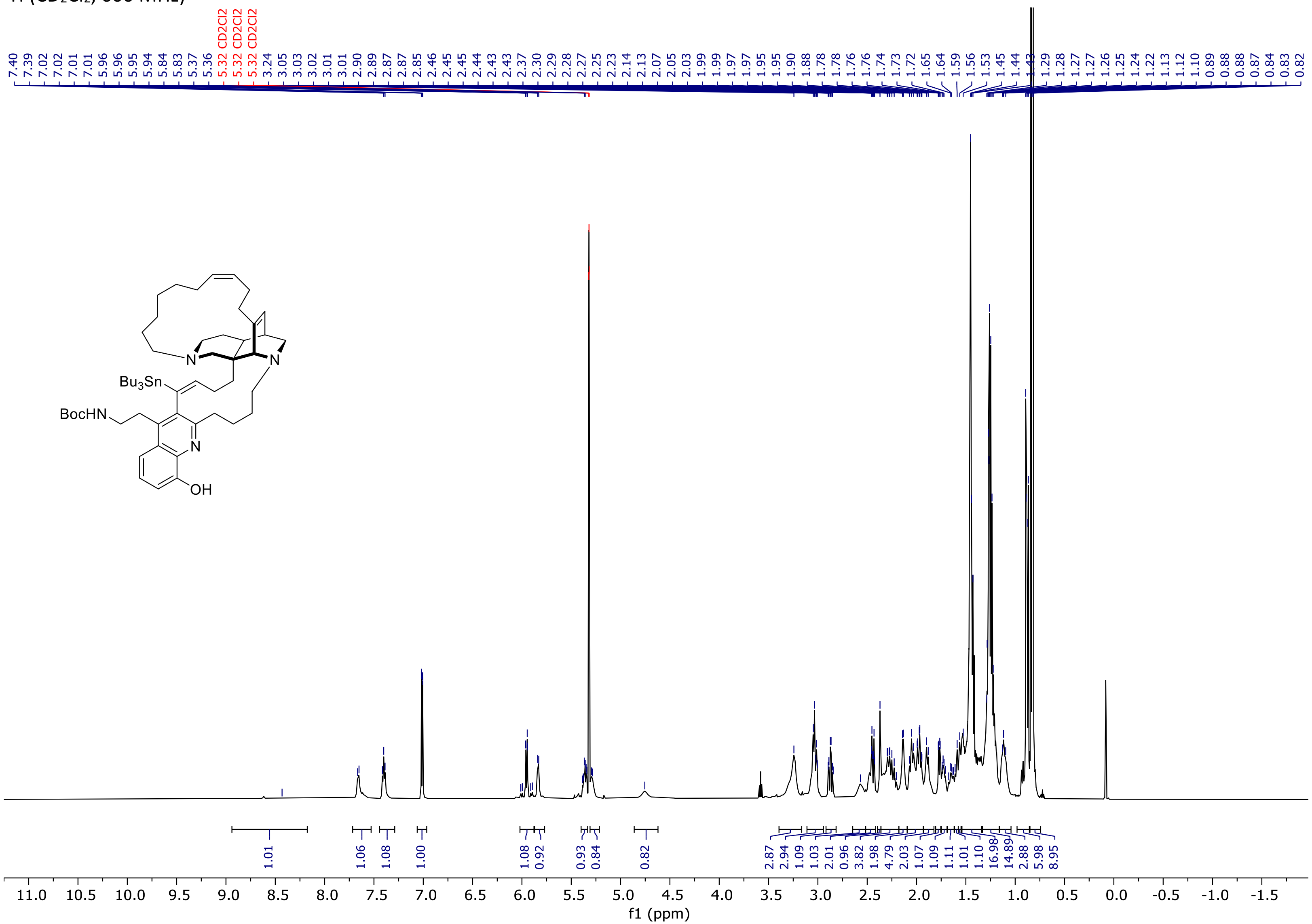

Compound S25

^{13}C (CD_2Cl_2, 151 MHz)

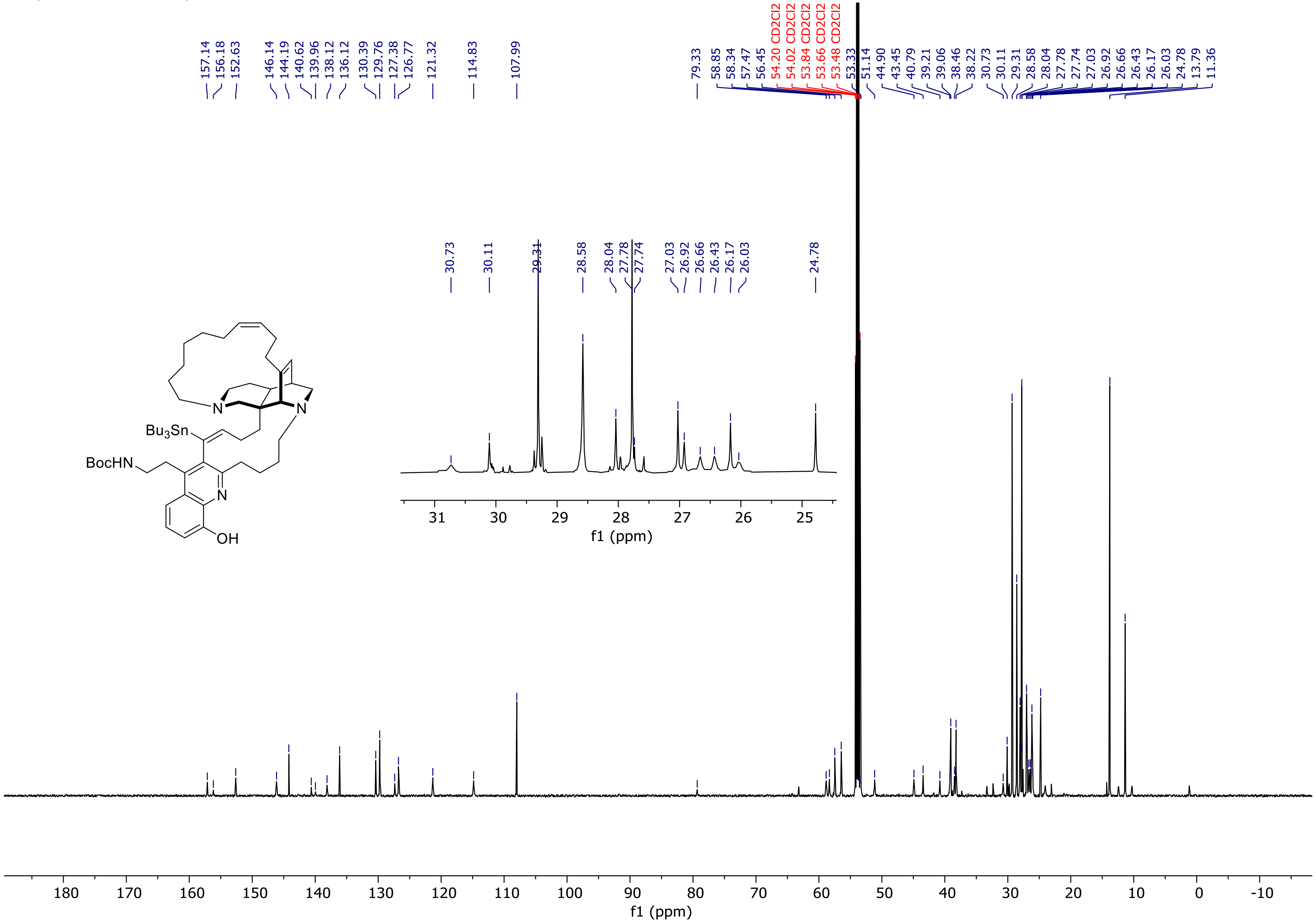

Compound S25

^{119}Sn-^{1}H HMBC (CD_2Cl_2, 400 MHz)

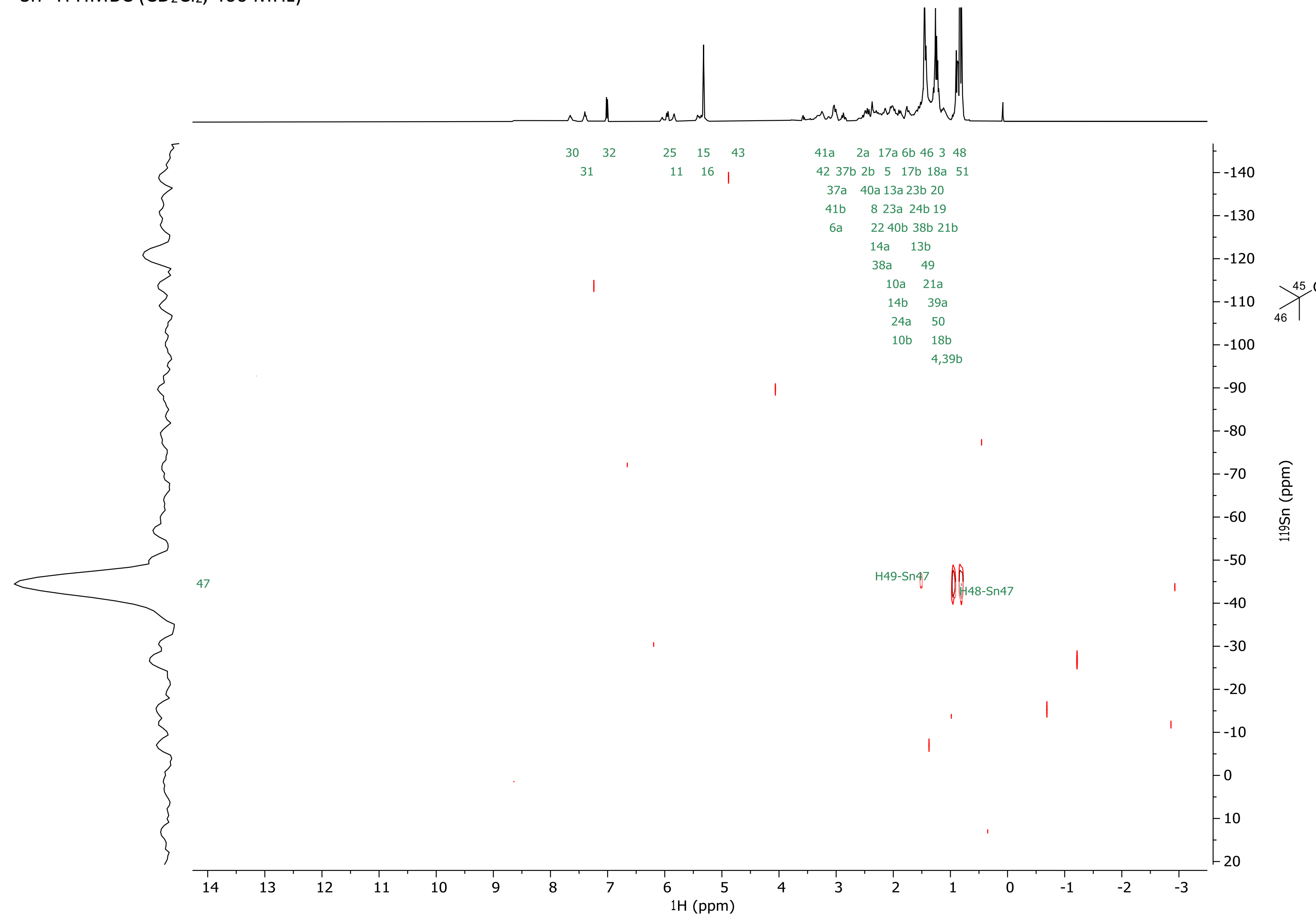

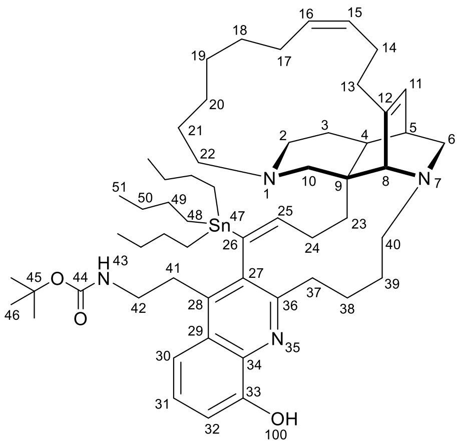

Compound (–)-3·Tfa

^{1}H (600 MHz, 3.3 mg in ca. 300 µL of Pyridine-d_5)

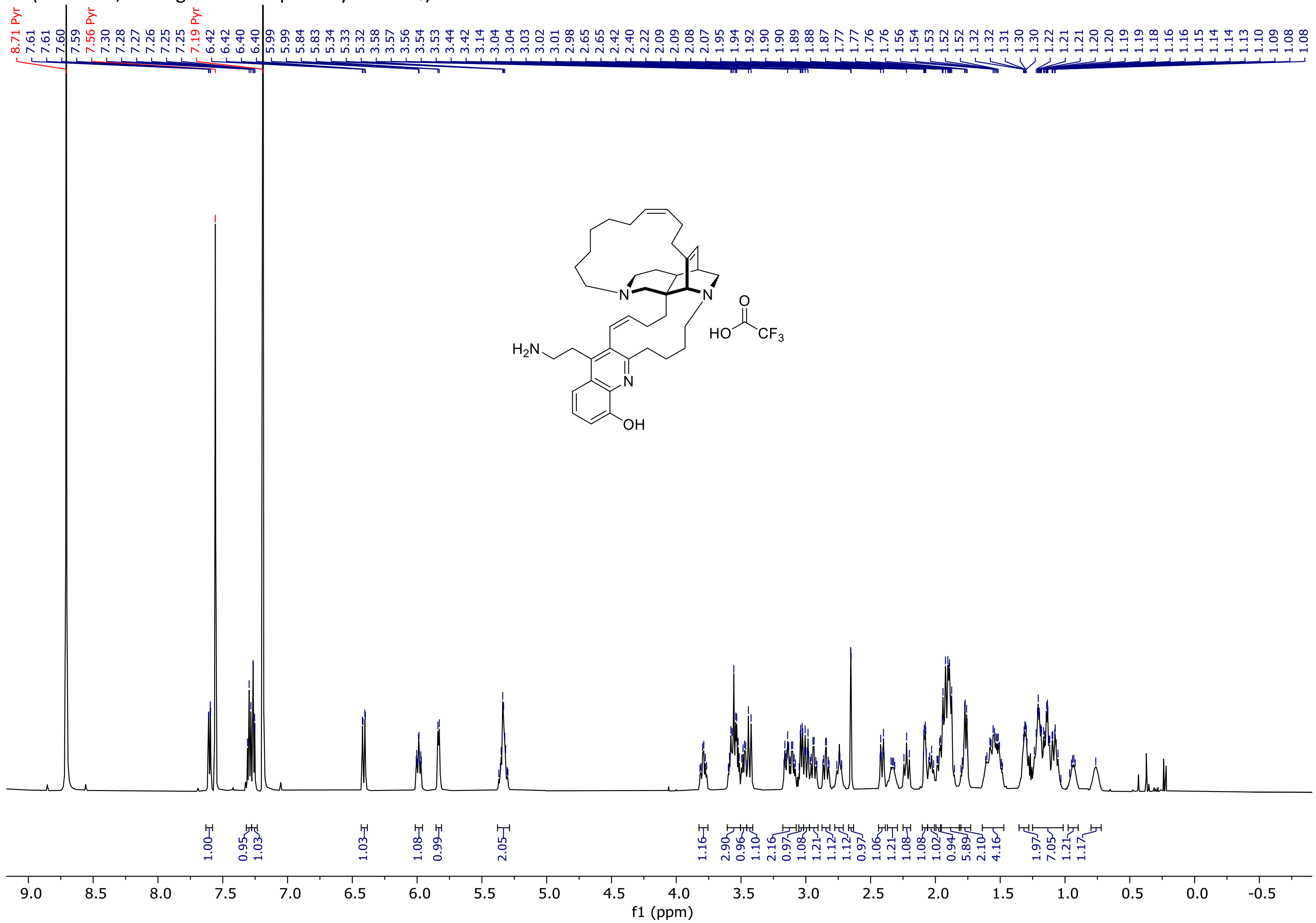

Compound (–)-3·Tfa

^{13}C (151 MHz, 3.3 mg in ca. 300 µL of Pyridine-d_5)

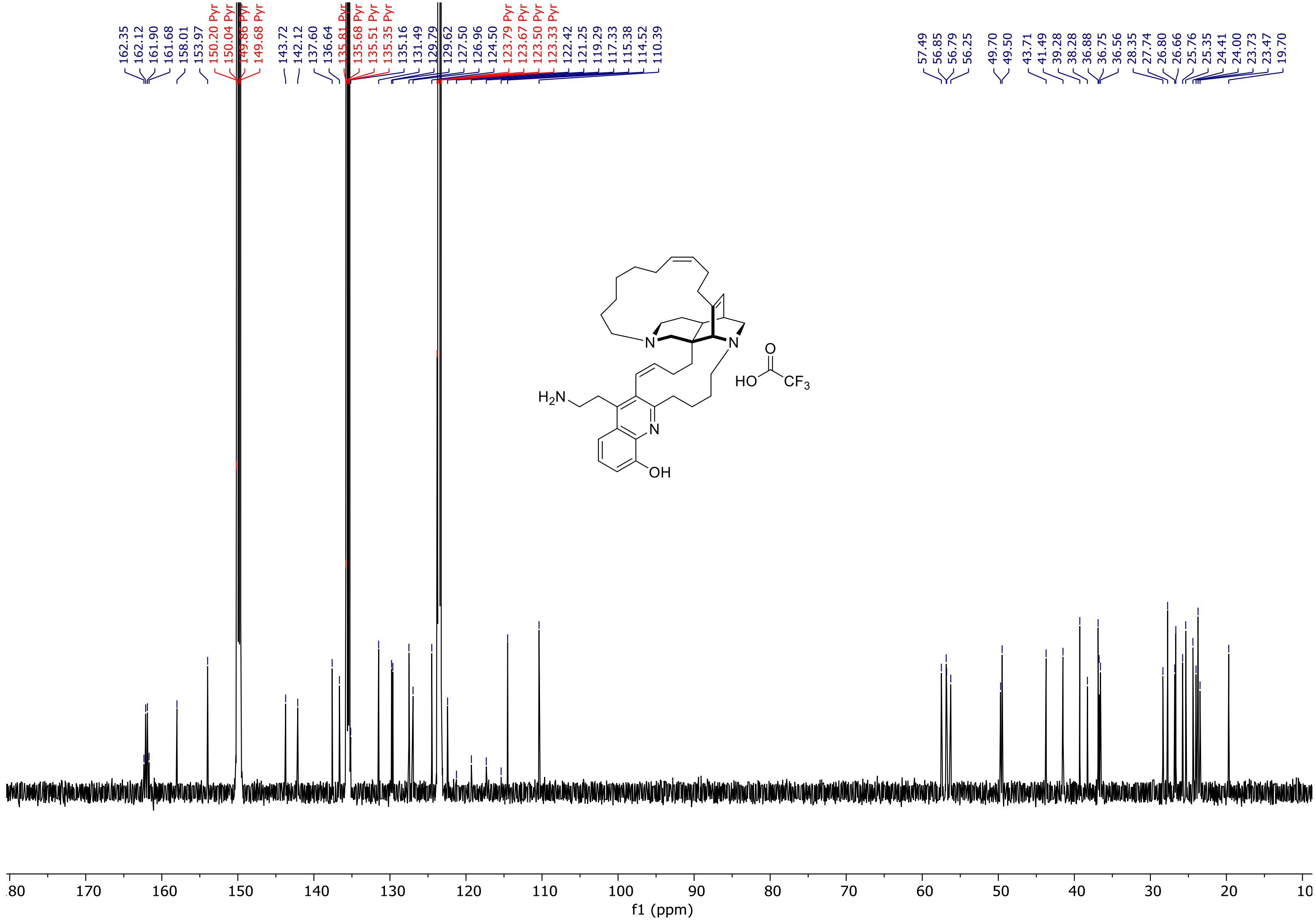

COSY Spectrum of **(–)-3·Tfa** (3.3 mg in ca. 300 µL of Pyridine-*d*5)

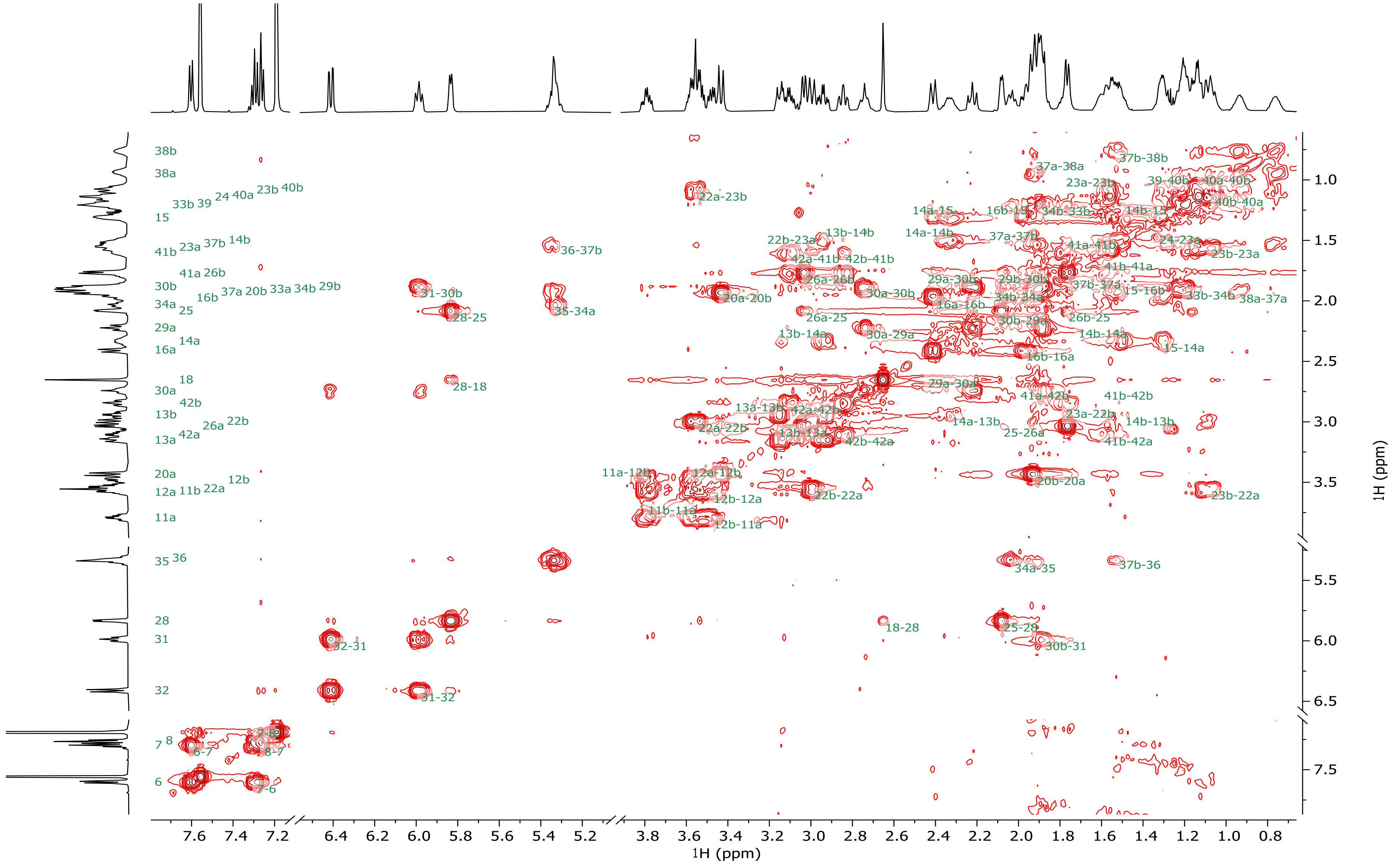

HSQC Spectrum of **(–)-3·Tfa** (3.3 mg in ca. 300 µL of Pyridine-d_5)

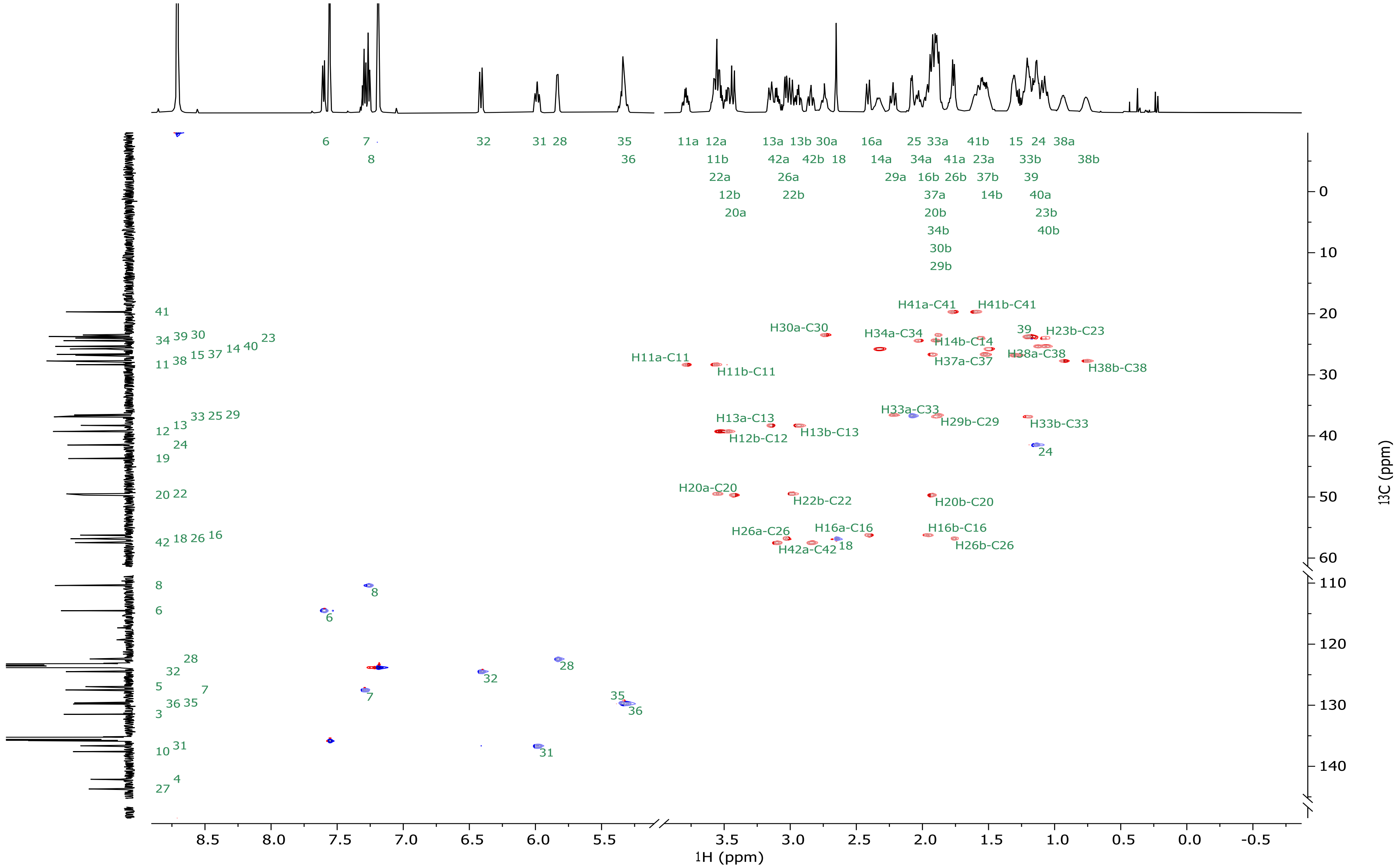

^{13}C-HMBC Spectrum of **(–)-3·Tfa** (3.3 mg in ca. 300 µL of Pyridine-d_5)

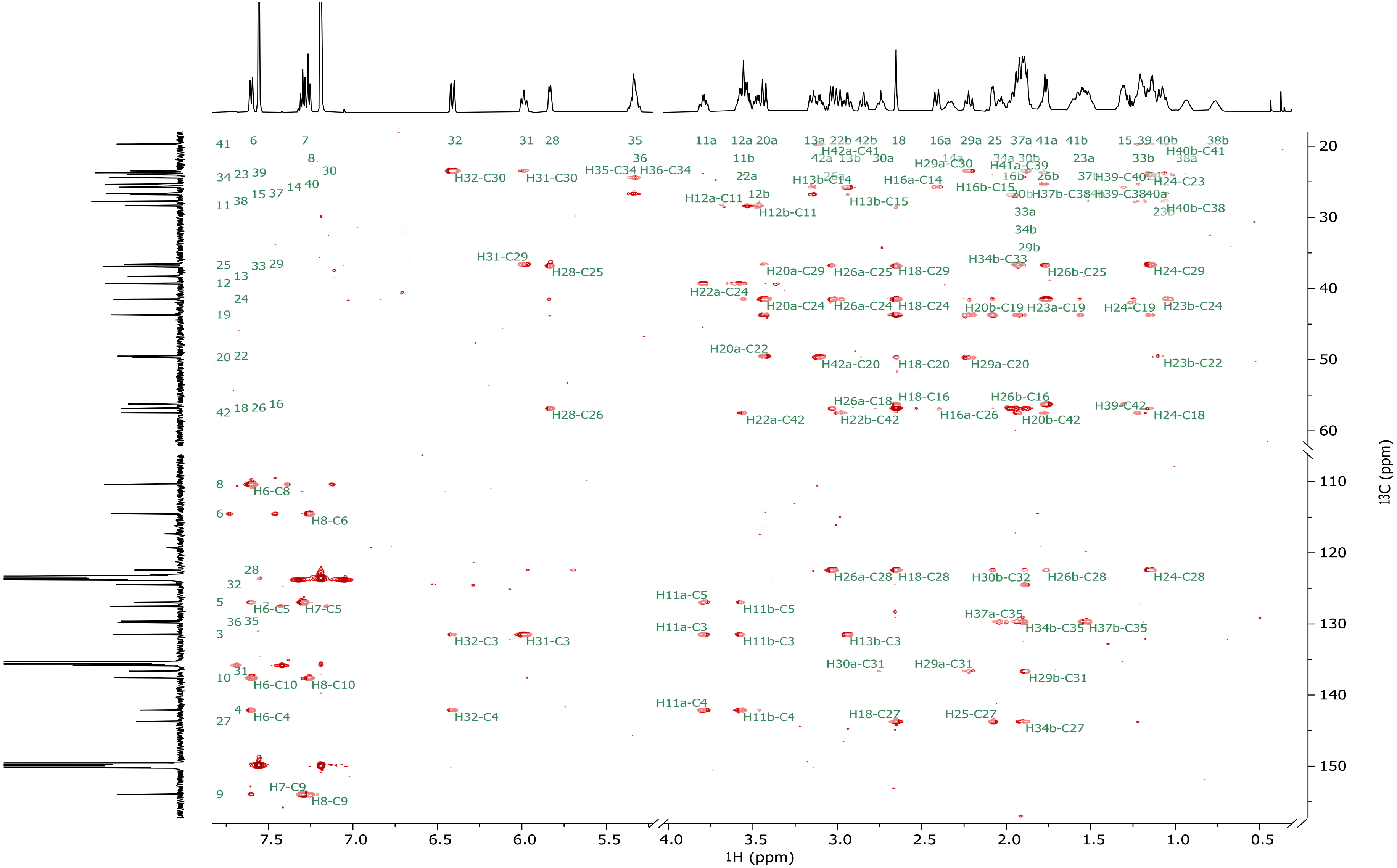

NOESY Spectrum of **(–)-3·Tfa** (3.3 mg in ca. 300 µL of Pyridine-*d*$_5$)

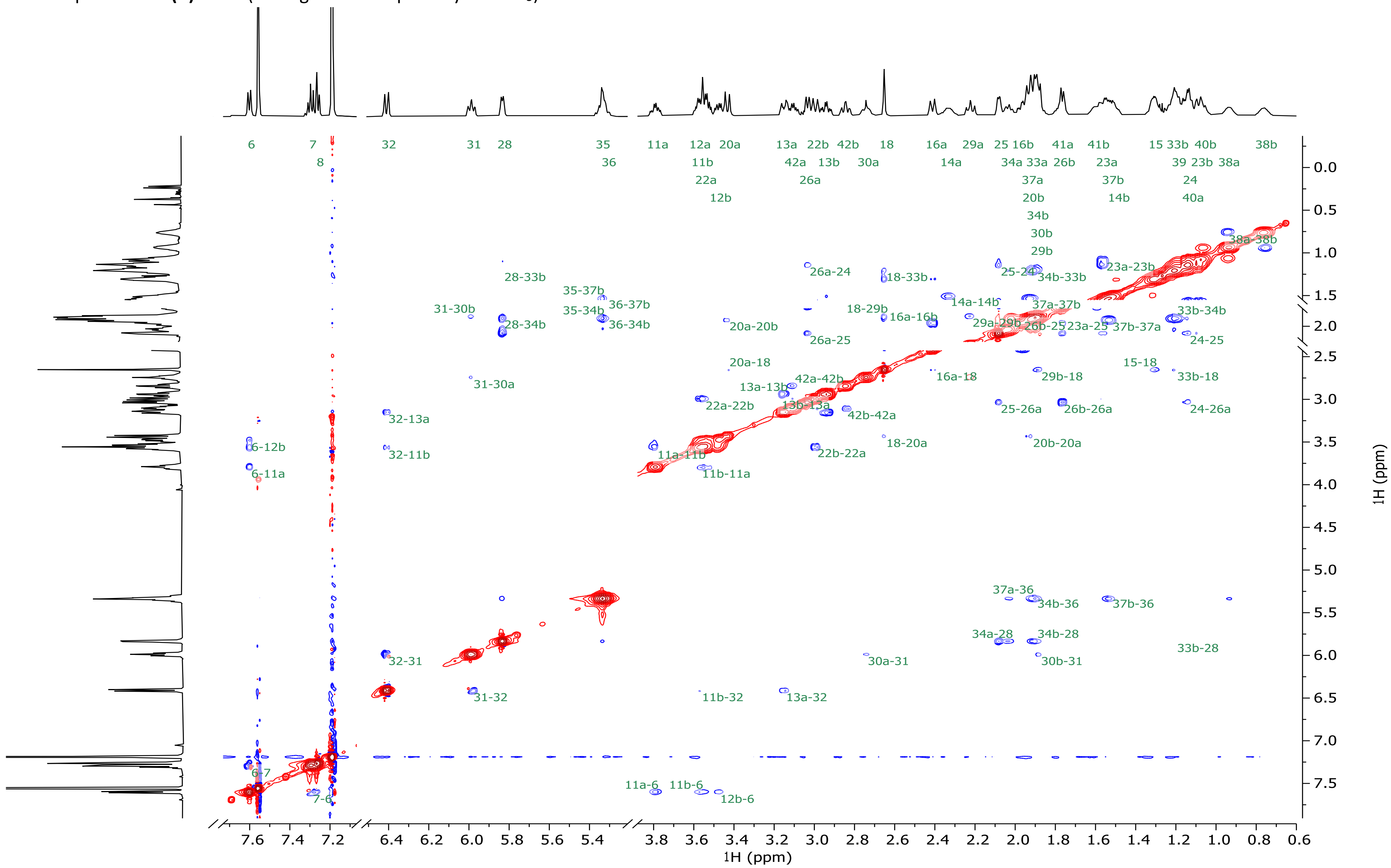

^{15}N-HMBC Spectrum of **(–)-3·Tfa** (3.3 mg in ca. 300 µL of Pyridine-d_5)

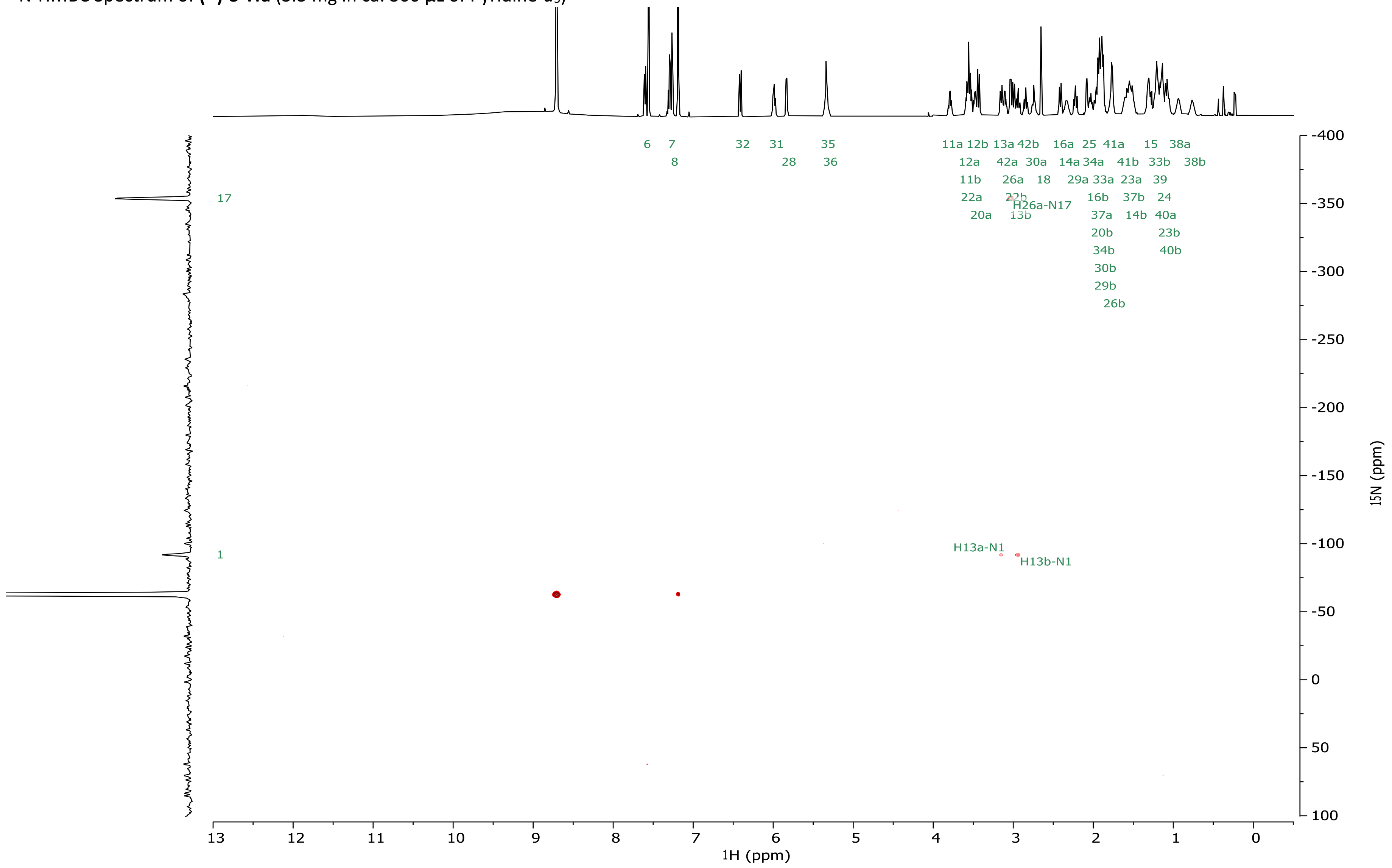

^{19}F NMR: **(–)-3·Tfa** (470 MHz, 3.3 mg in ca. 300 µL of Pyridine-d_5)

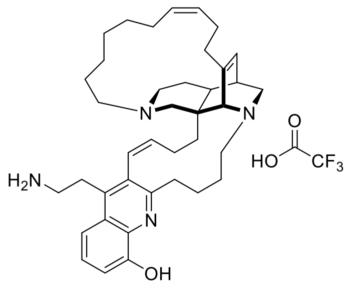

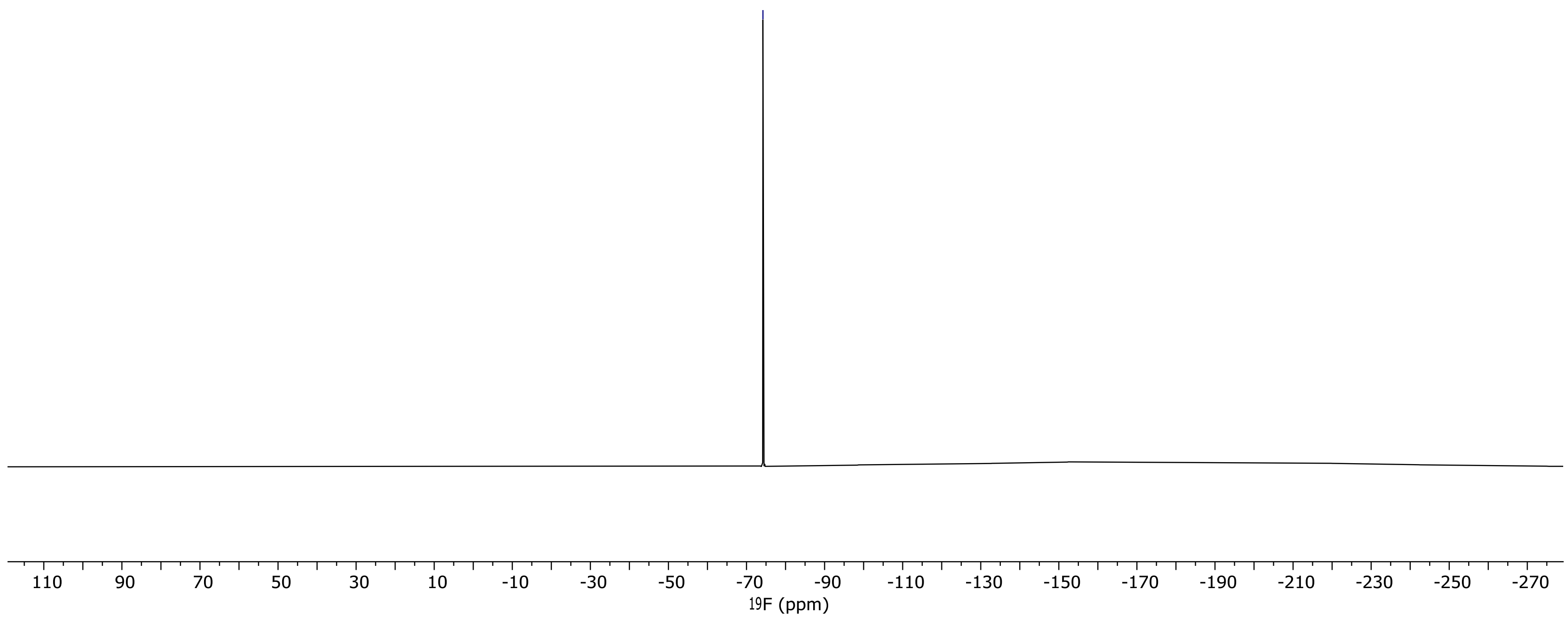

Compound 41

^{1}H (CD_2Cl_2, 600 MHz)

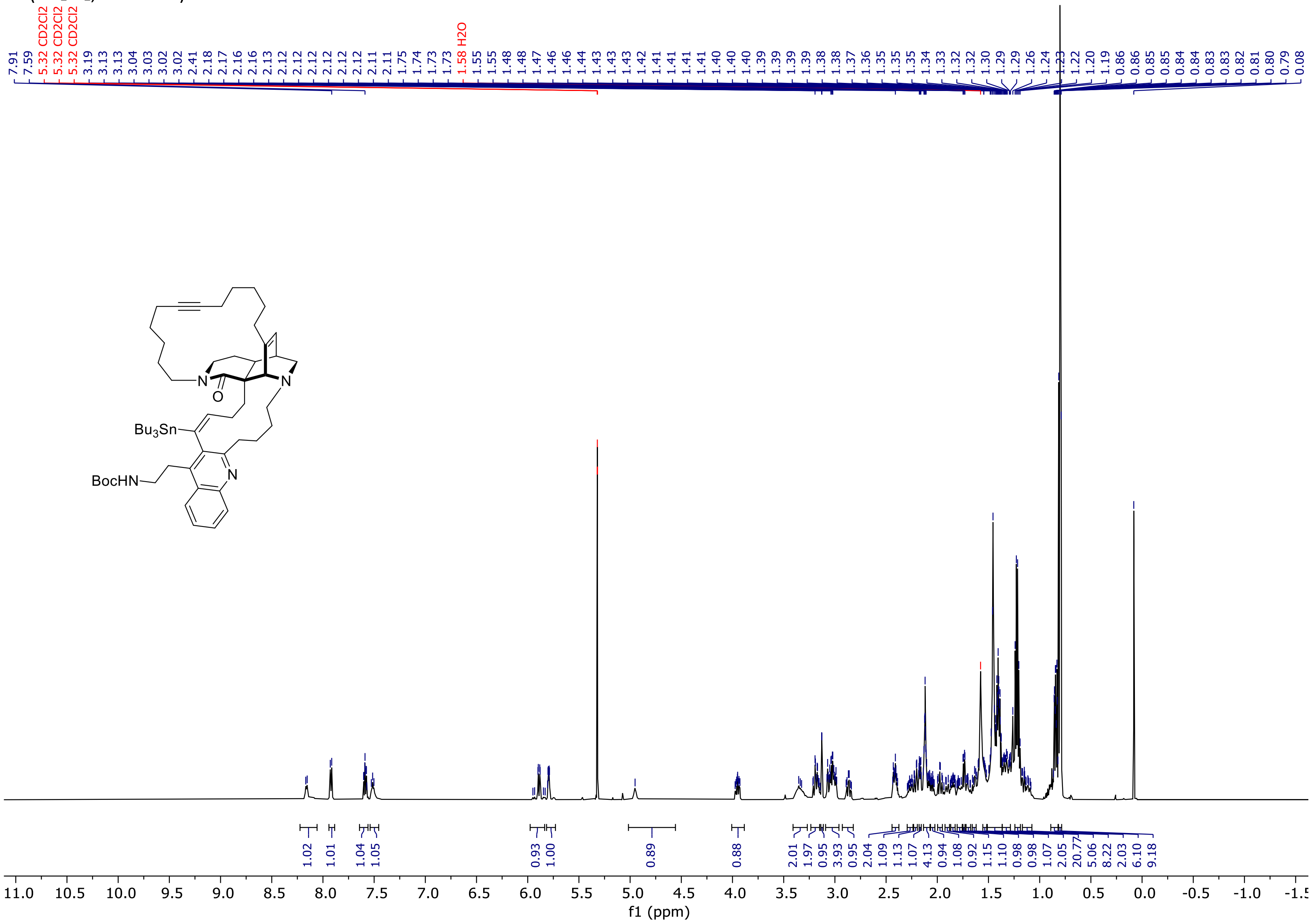

Compound 41

^{13}C (CD_2Cl_2, 151 MHz)

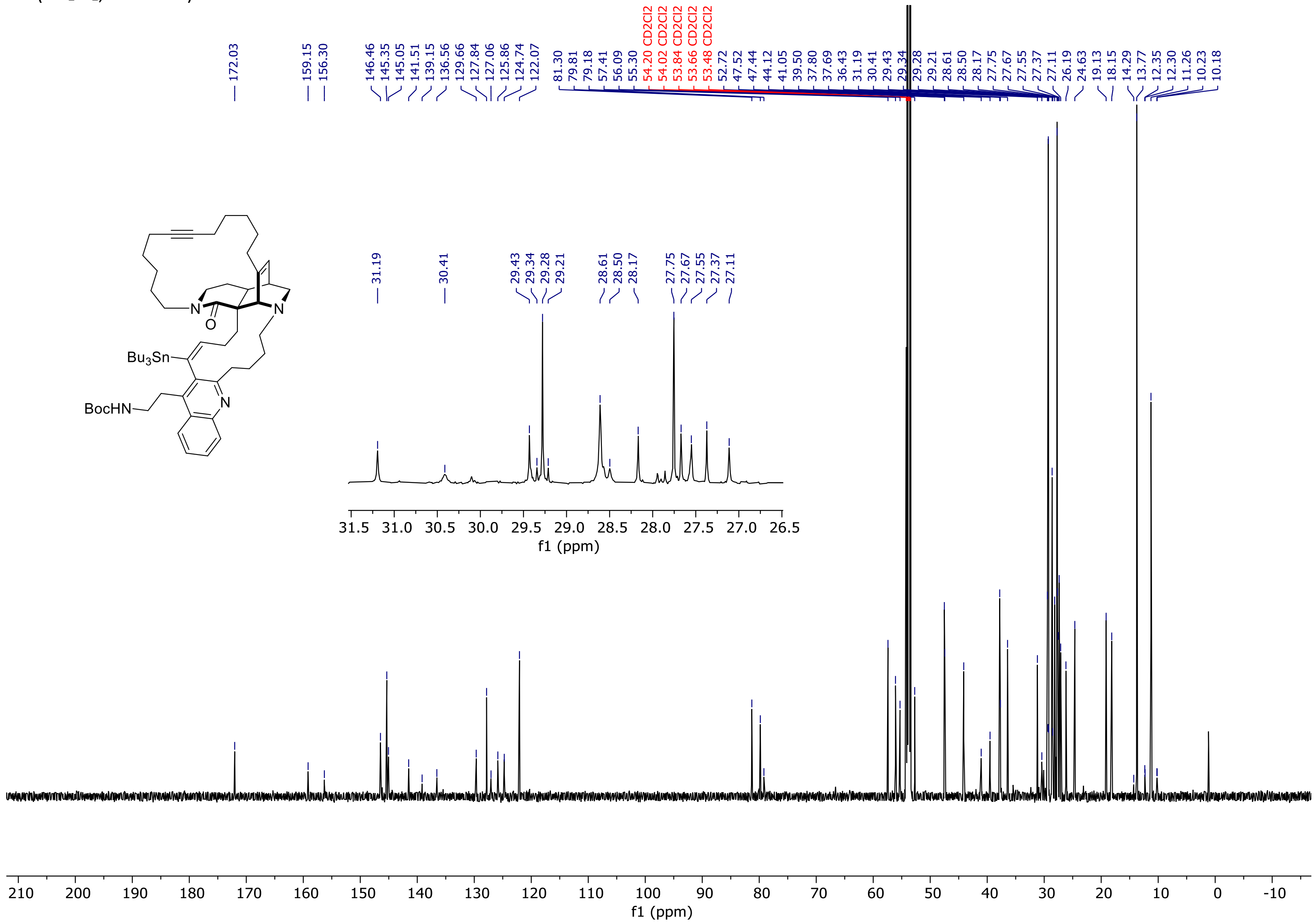

Compound 41

^{119}Sn (CD_2Cl_2, 149 MHz)

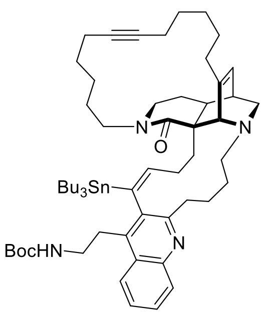

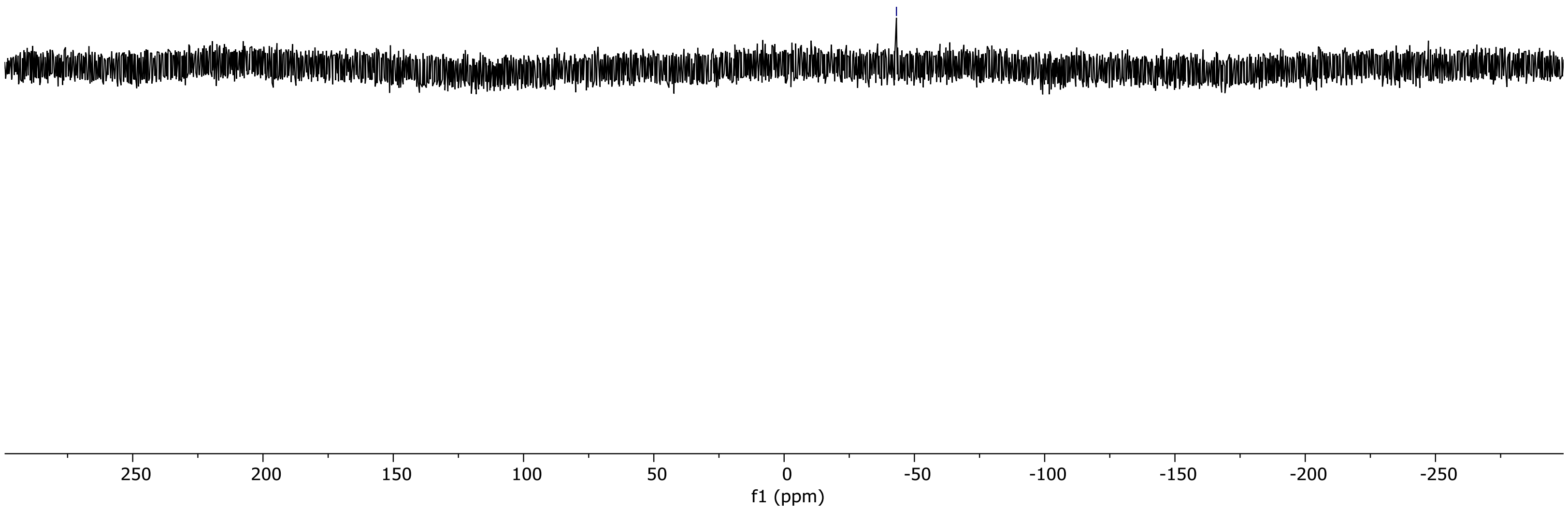

COSY Spectrum of **41**

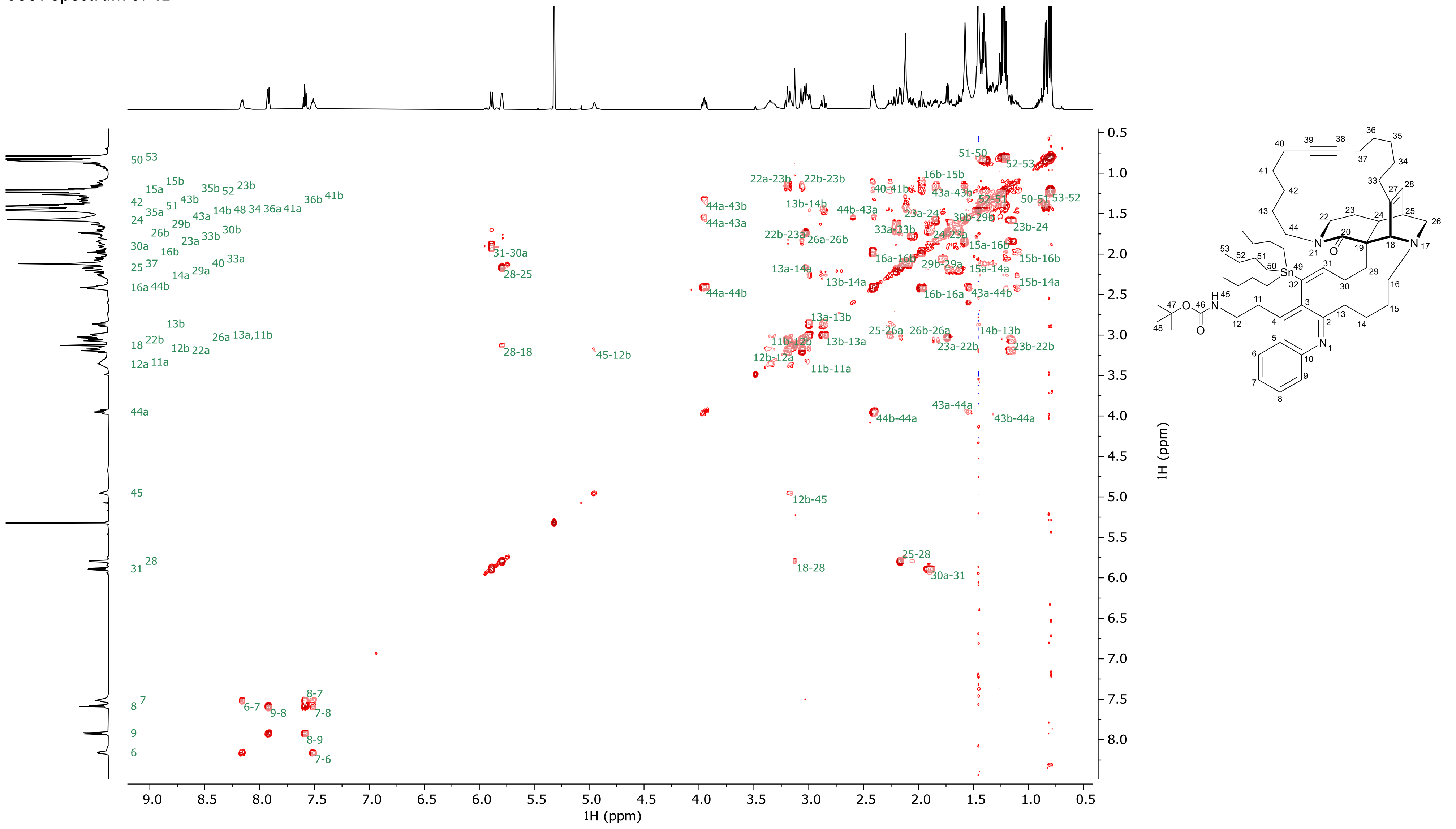

HSQC Spectrum of **41**

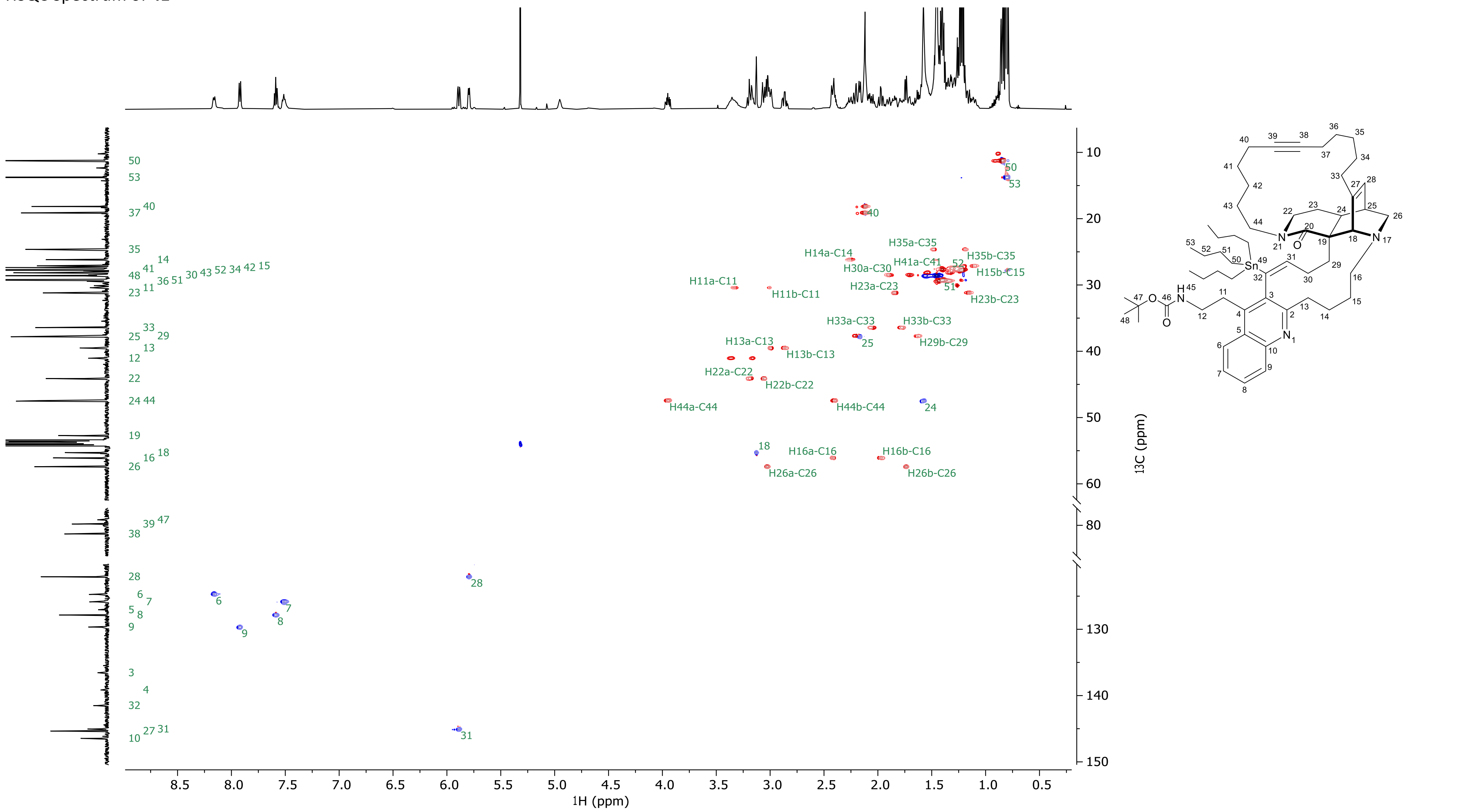

HMBC Spectrum of **41**

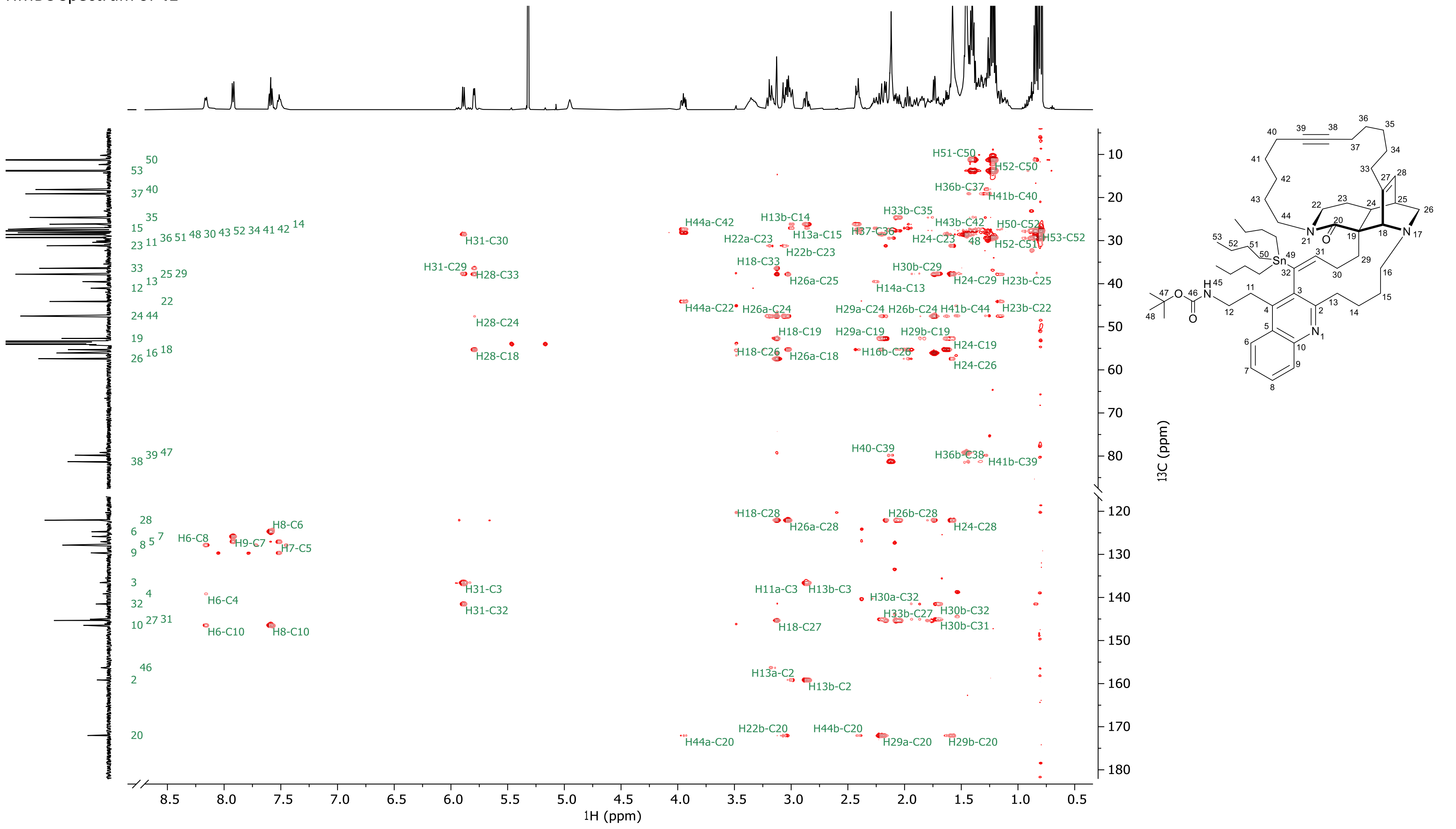

Compound 42·Tfa

^{1}H (Pyridine-d_5, 600 MHz)

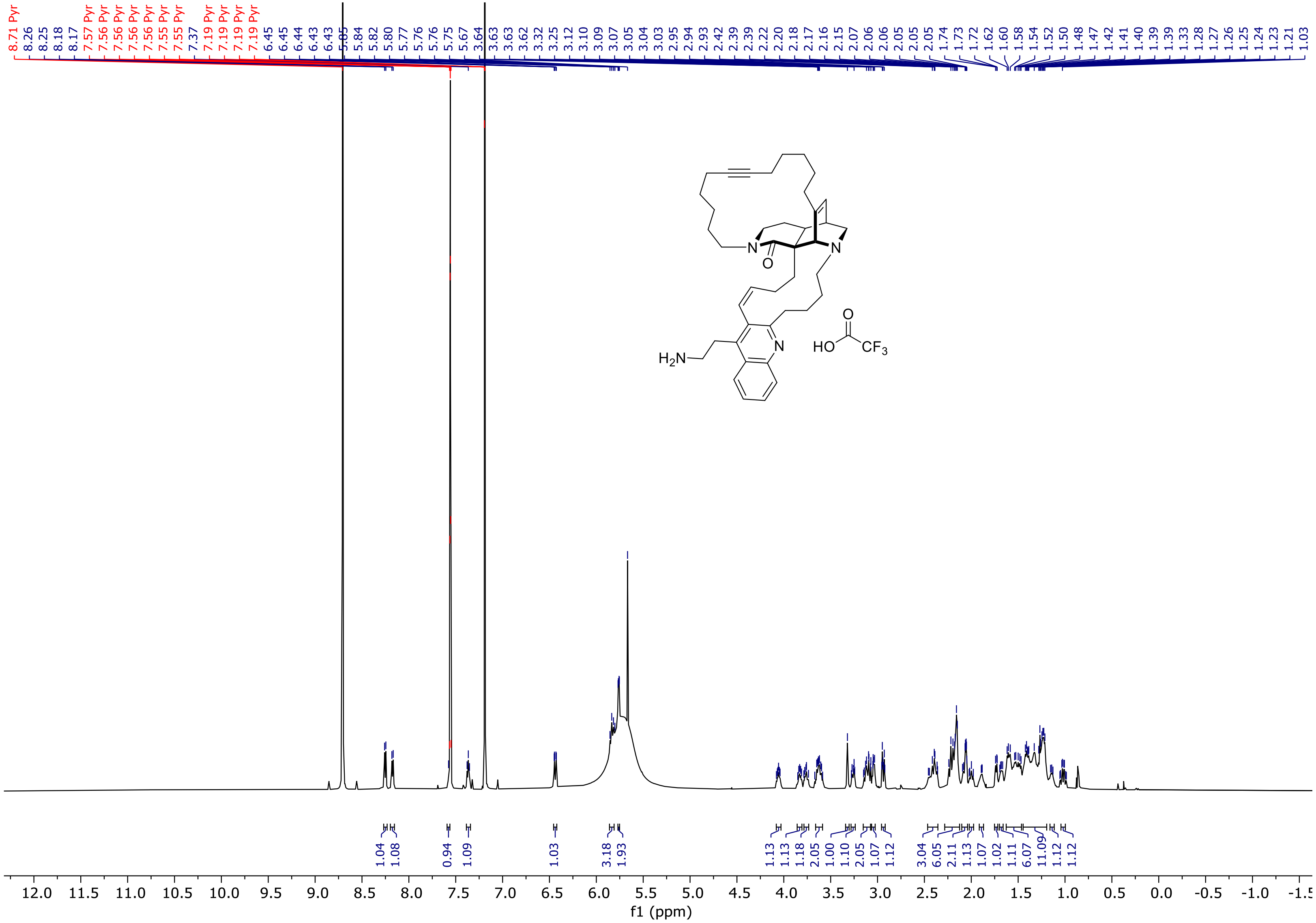

Compound 42·Tfa

^{13}C (Pyridine-d_5, 151 MHz)

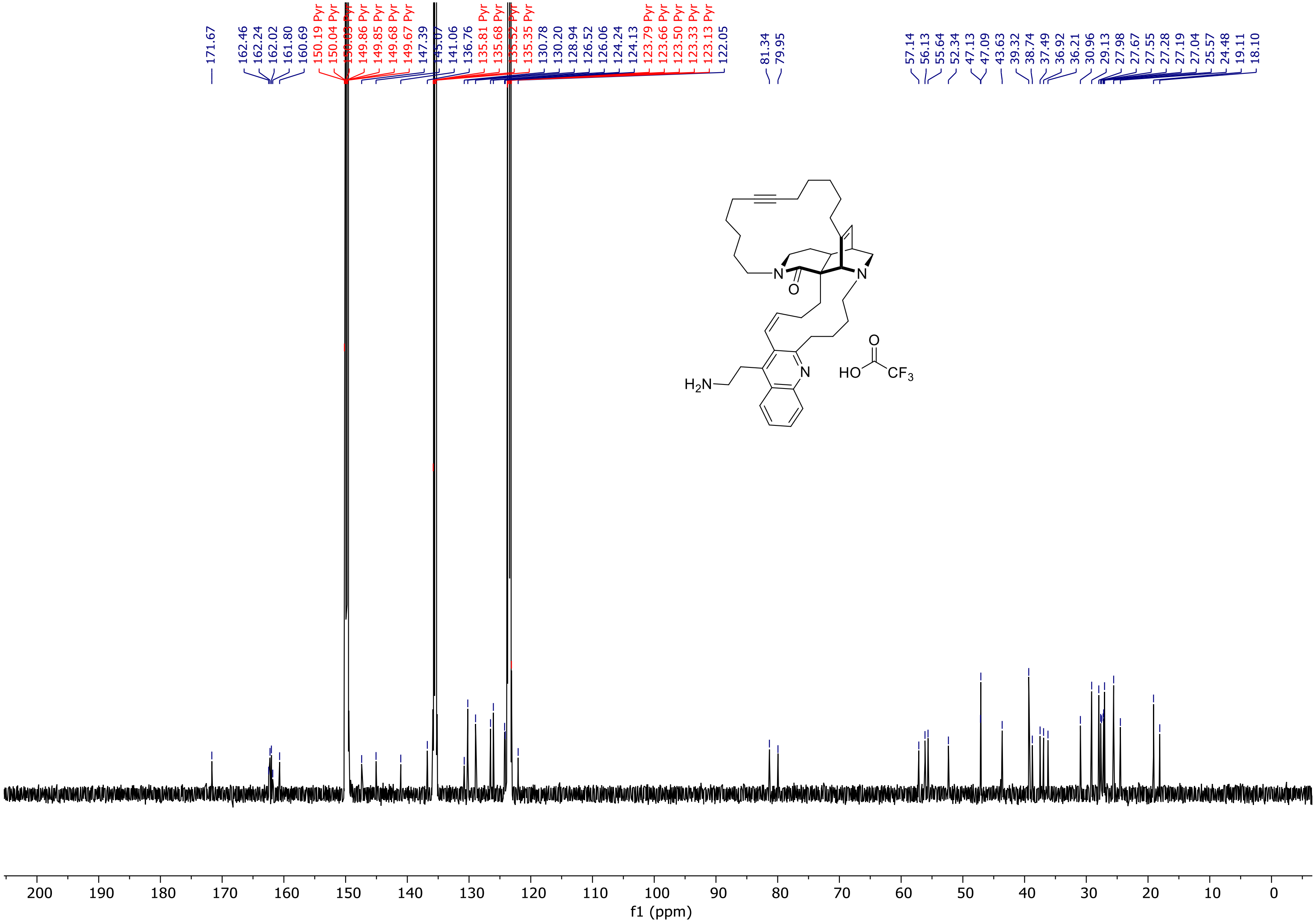

COSY Spectrum of **42·Tfa**

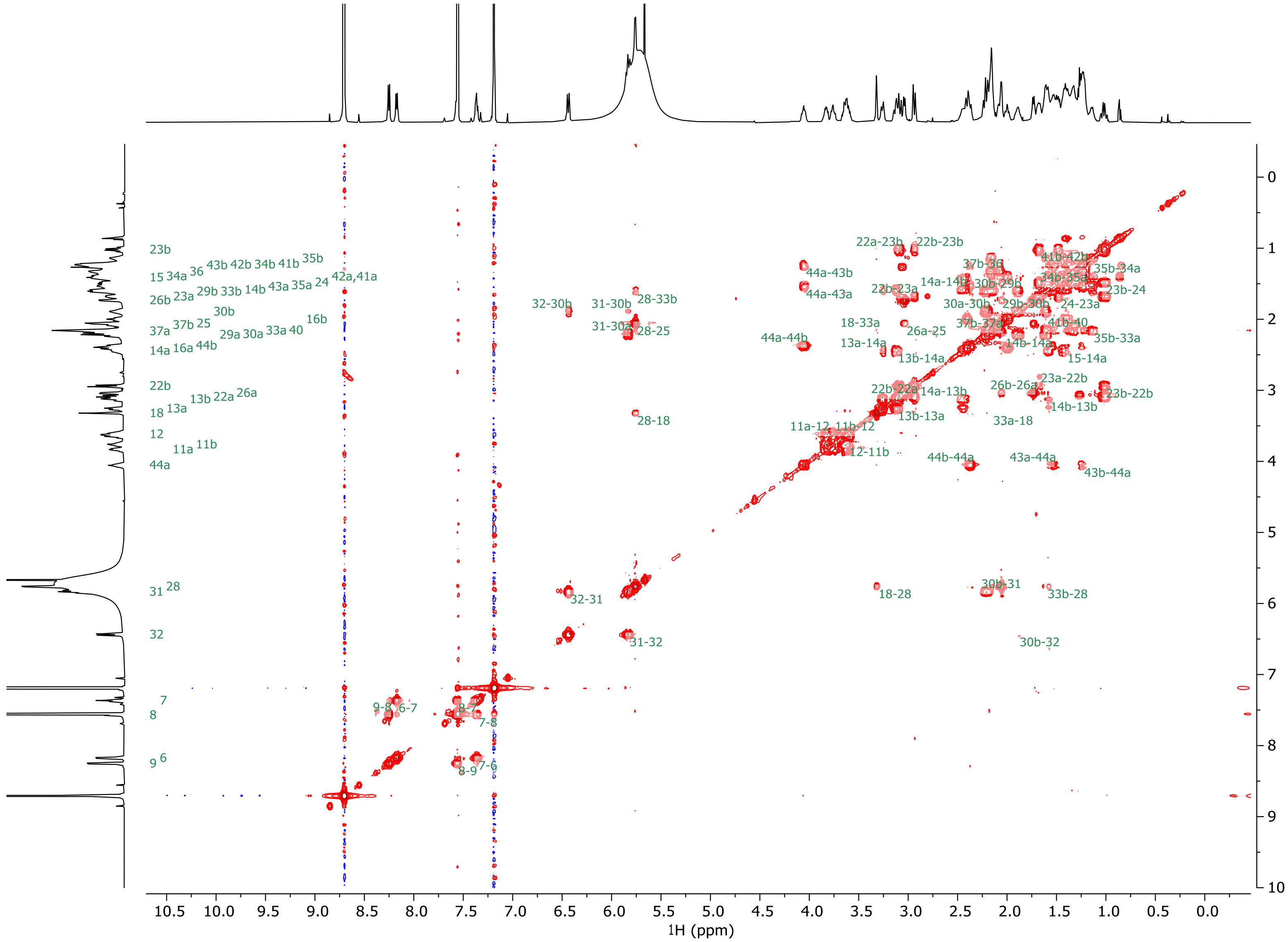

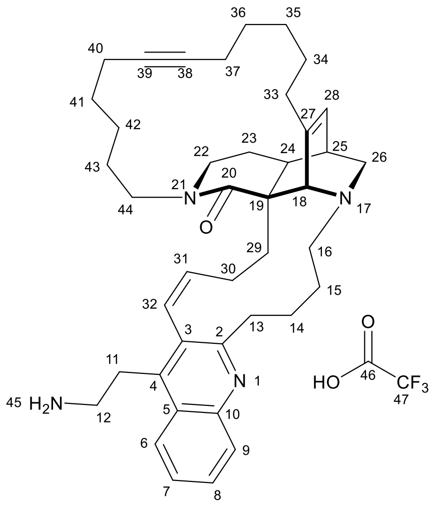

HSQC Spectrum of **42·Tfa**

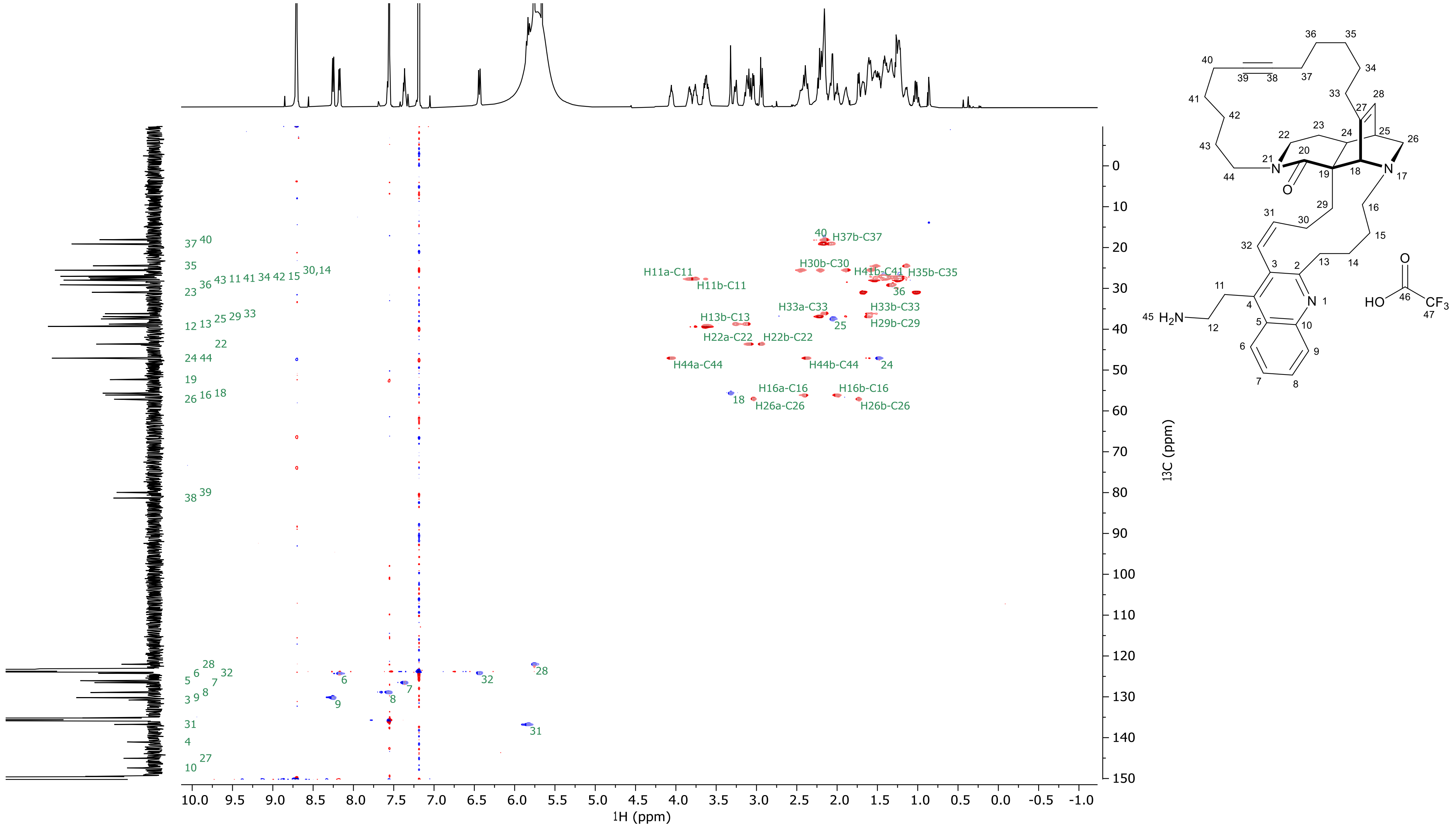

HMBC Spectrum of **42·Tfa**

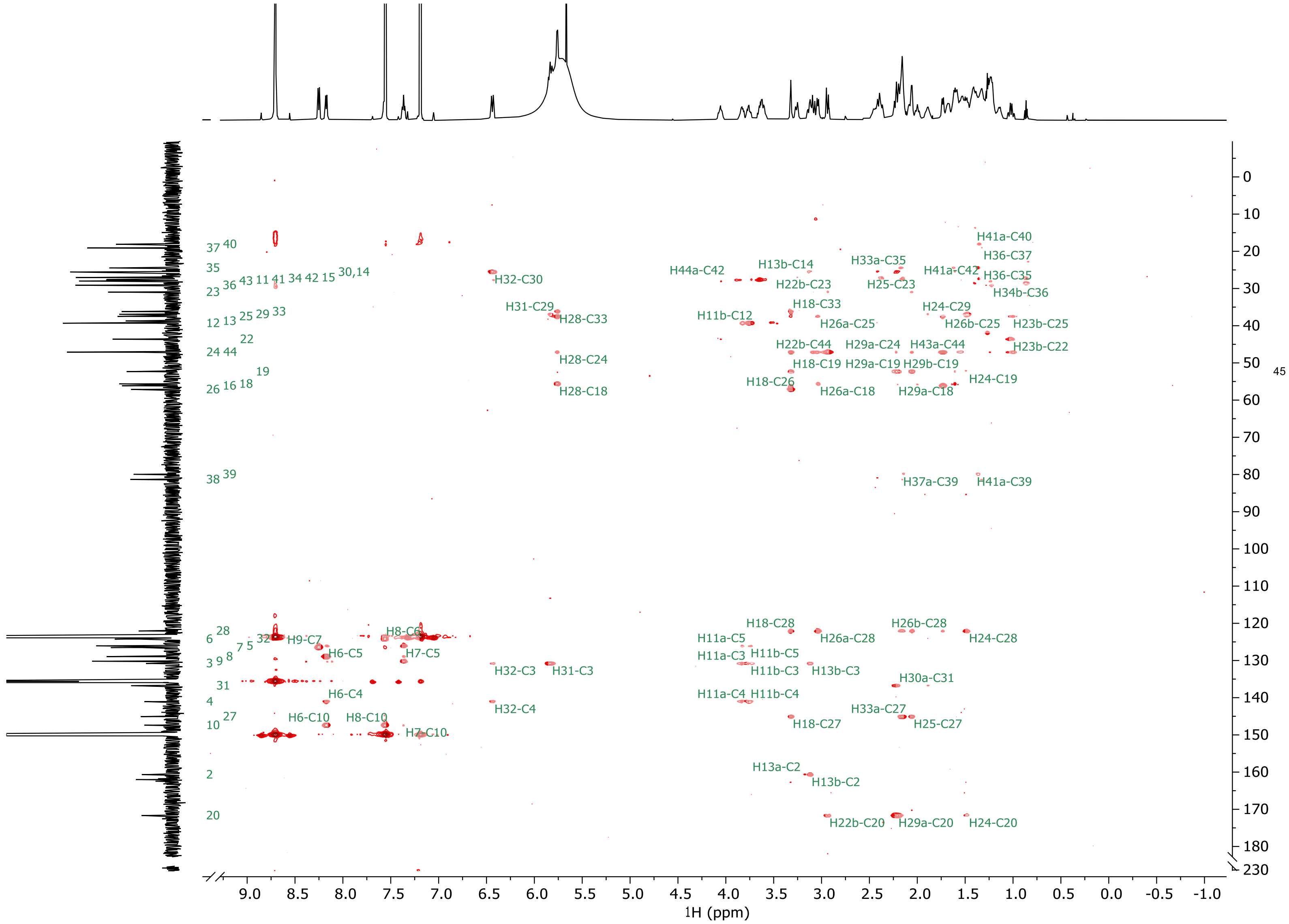

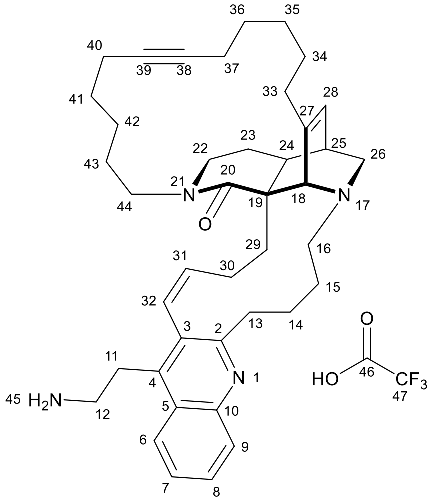

NOESY Spectrum of **42·Tfa**

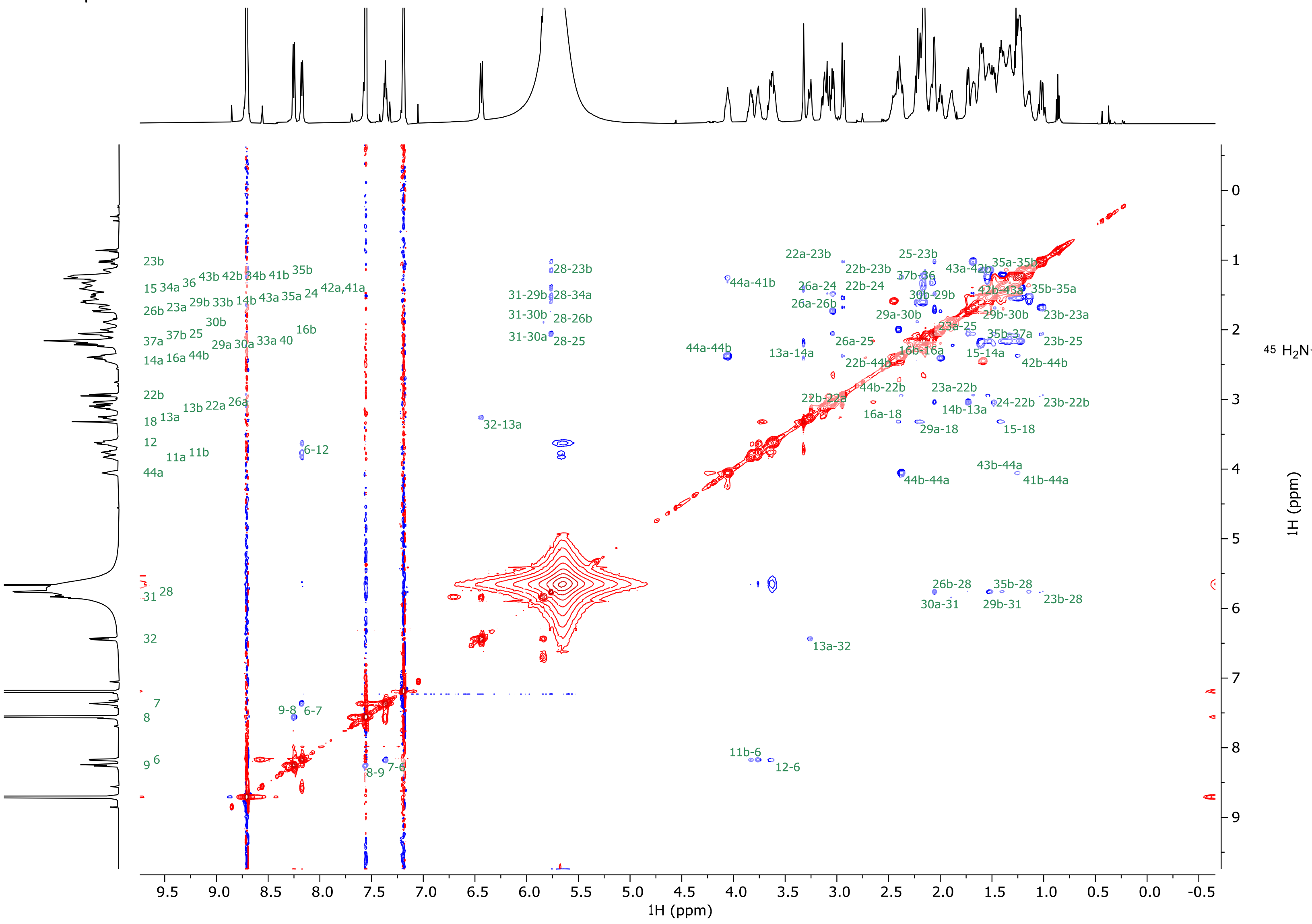

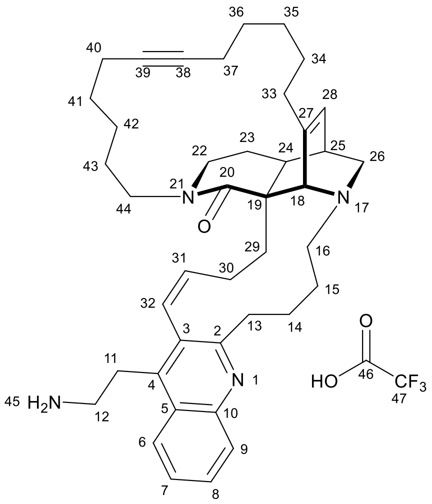

Supplement: Supplementary file 2 — ja3c08410_si_002.pdf [file ja3c08410_si_002.pdf]
